# The convergent total synthesis and antibacterial profile of the natural product streptothricin F†

**DOI:** 10.1039/d1sc06445b

**Published:** 2022-02-25

**Authors:** Matthew G. Dowgiallo, Brandon C. Miller, Mintesinot Kassu, Kenneth P. Smith, Andrew D. Fetigan, Jason J. Guo, James E. Kirby, Roman Manetsch

**Affiliations:** Department of Chemistry and Chemical Biology, Northeastern University Boston MA USA r.manetsch@northeastern.edu; Department of Pharmaceutical Sciences, Northeastern University Boston MA USA; Center for Drug Discovery, Northeastern University Boston MA USA; Barnett Institute for Chemical and Biological Analysis, Northeastern University Boston MA USA; Department of Pathology, Beth Israel Deaconess Medical Center Boston MA USA; Harvard Medical School Boston MA USA

## Abstract

A convergent, diversity-enabling total synthesis of the natural product streptothricin F has been achieved. Herein, we describe the potent antimicrobial activity of streptothricin F and highlight the importance of a total synthesis that allows for the installation of practical divergent steps for medicinal chemistry exploits. Key features of our synthesis include a Burgess reagent-mediated 1,2-*anti*-diamine installation, diastereoselective azidation of a lactam enolate, and a mercury(ii) chloride-mediated desulfurization-guanidination. The development of this chemistry enables the synthesis and structure–activity studies of streptothricin F analogs.

## Abstract 2

The second ever total synthesis of streptothricin F and the first achieved through a diversity-enabling convergent route. The synthesis is achieved in 35 total steps, with a longest linear sequence of 19 steps, and 0.40% overall yield.

## Graphical abstract

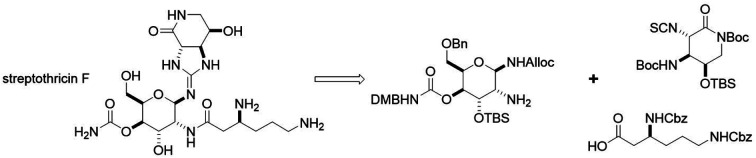

## Introduction

**Fig. 1:**
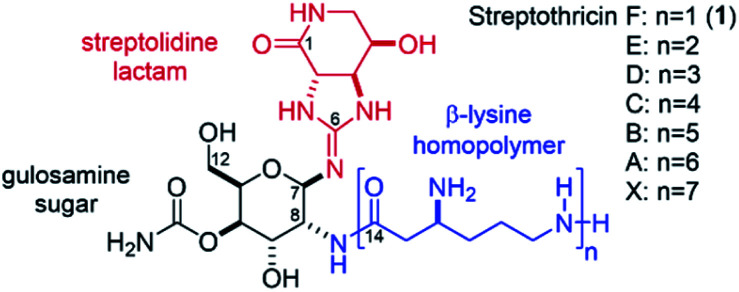
Structures of the streptothricins A–F, and X. The streptolidine lactam is shown in red, the gulosamine core is shown in black, and the β-lysine homopolymer is shown in blue.

The streptothricins are a class of natural products exhibiting potent antimicrobial activity against multidrug-resistant, Gram-negative bacteria. Streptothricins were first isolated in 1942 by Waksman and Woodruff from *Streptomyces lavendulae*^1^ and have since been identified under a variety of pseudonyms from other *Streptomyces* species.^2–9^ Isolates of streptothricin generally exist as complex mixtures of homologs A–F, and X (Fig. 1). These mixtures are typically referred to as “nourseothricin” and contain varying ratios of the component streptothricins, with streptothricin F (1) being the principal component. Nourseothricin attracted initial interest because of the impressive Gram-positive and Gram-negative antimicrobial activity^1,6,10–13^ and high water solubility^10,14–16^ of the streptothricins. However, this natural product class has not been pursued as a therapeutic due to inherent toxicity.^7,17–20^ Additionally, isolation of the individual streptothricin components of nourseothricin has proven to be challenging, with limited reports of biological characterization on demonstrated pure material.^6,19,21^ The streptothricin backbone consists of a carbamoylated gulosamine sugar core (Fig. 1, black) affixed with a streptolidine lactam moiety (red) and β-lysine homopolymer (blue) attached to the C7 and C8 amines, respectively. Streptolidine is an unusual guanidine-containing amino acid that has been isolated as a streptothricin hydrolysis product and appears to be unique to this natural product class.^22^ Additionally, the rarity of β-amino acids adds another layer of structural peculiarity, synthetic challenge, and a unique opportunity for medicinal chemistry discovery.^23^

**Scheme 1:**
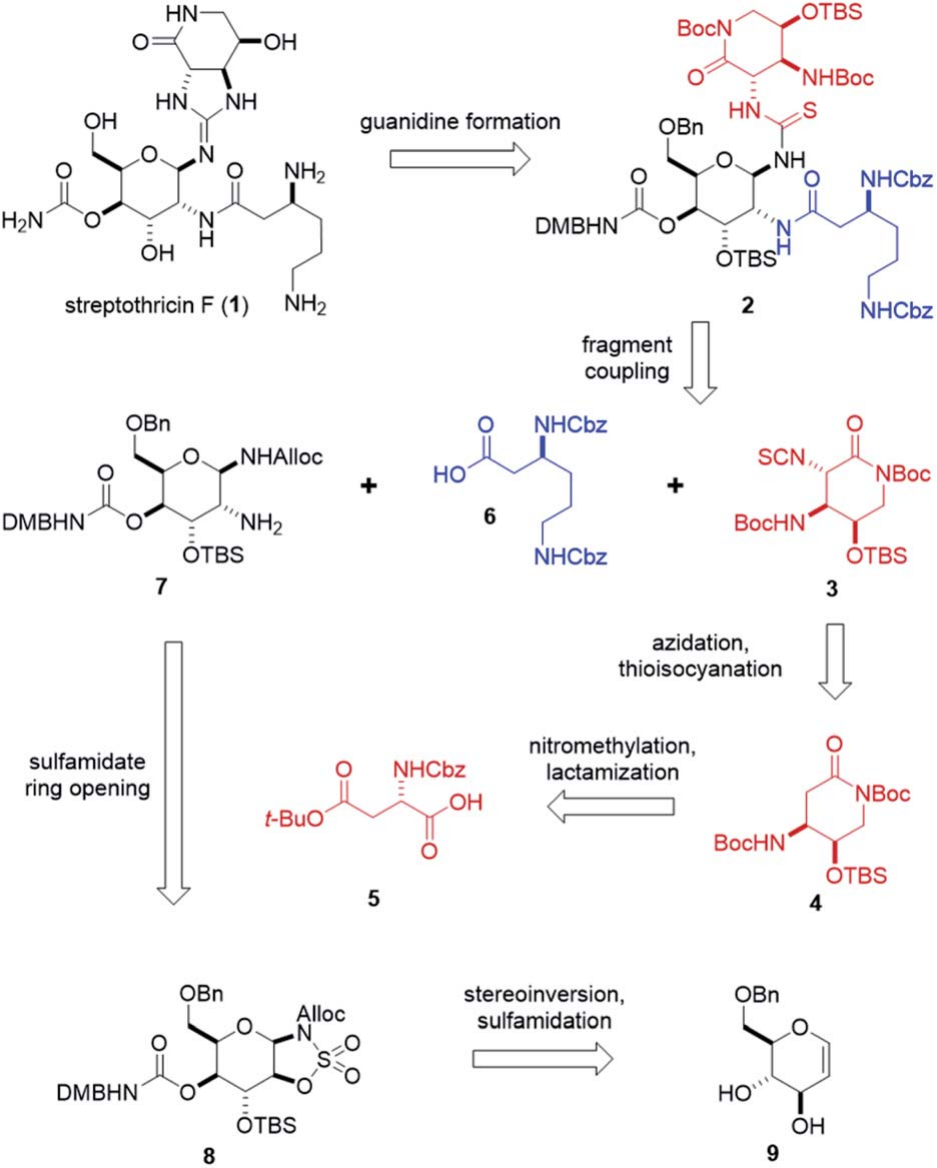
Retrosynthetic analysis of streptothricin F (1).

Streptothricins were discovered to be vulnerable to resistance through two mechanisms. The β-amine of the β-lysine moiety is susceptible to an acylation-based resistance mechanism in bacteria containing streptothricin acetyl transferases^24–30^ while enzymatic hydrolysis of the streptolidine moiety proceeds through a less-prominent resistance pathway.^31^ Streptothricin F was previously found to inhibit prokaryotic ribosomal translocation and also induce significant miscoding. That is, like aminoglycosides, they cause incorrect amino acids to be added to the growing peptide chains during protein synthesis and thereby poison the bacterial cell leading to cell death. Experimentally streptothricin F was previously found not to effect nucleic acid synthesis.^32,33^ Our main attraction to this natural product class derives from previous reports that have demonstrated streptothricin F (1) to be less toxic than other streptothricins. Studies of purified streptothricins in mice indicate that toxicity is influenced directly by the unit length (*n*) of the β-lysine homopolymer. Streptothricin F, (*n* = 1, LD_50_: 300 mg kg^−1^) shows remarkably less toxicity than streptothricin E (*n* = 2, LD_50_: 26 mg kg^−1^), streptothricin D (*n* = 3, LD_50_: ∼10 mg kg^−1^), and streptothricin C (*n* = 4, LD_50_: ∼10 mg kg^−1^).^6,15,34^ Conversely, antimicrobial activity favors longer β-lysine homopolymer chains, with nourseothricin (*K. pneumoniae* Nevada strain AR-0636 MIC: 0.15 μg mL^−1^) and streptothricin D (MIC: 0.19 μg mL^−1^) exhibiting approximately 4-fold more activity (per mol) than streptothricin F (MIC: 1 μM).^34^

**Scheme 2:**
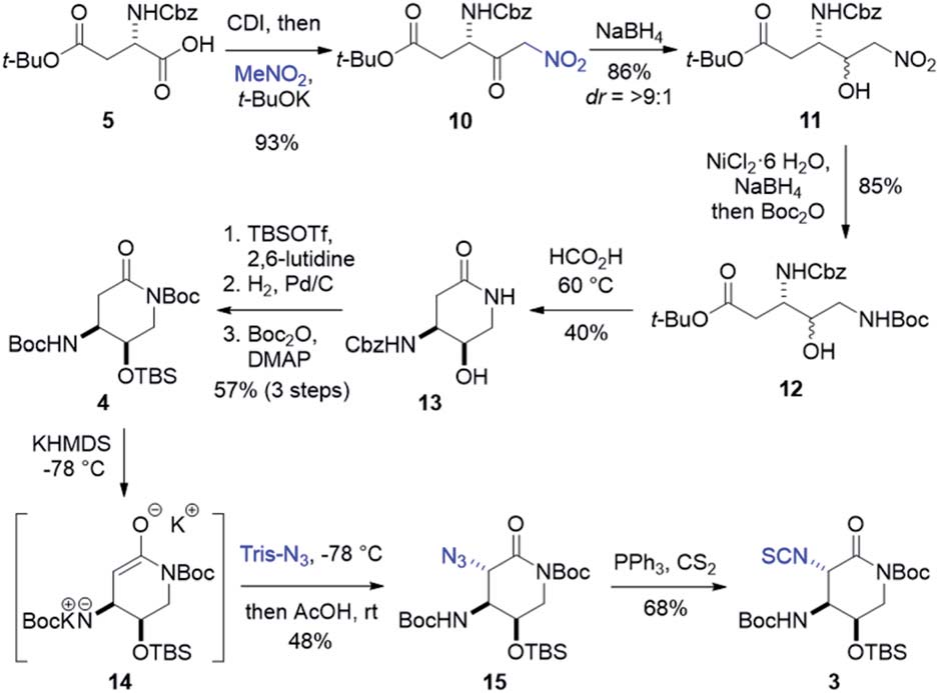
Synthesis of streptolidine isothiocyanate 3.

## Results and discussion

To fully explore the therapeutic potential of this promising scaffold, it is important to develop an efficient and robust total synthesis to produce significant quantities of streptothricins and analogs. A single total synthesis of streptothricin F has been reported in the literature by Shiba and co-workers,^35^ and no other streptothricin has been attained through synthetic means exclusively. While a landmark for its time, Shiba's synthesis contains over 46 total steps with a longest linear sequence of 25 steps and an overall yield of less than 0.28%.^36^ While Shiba's synthesis contains elements of convergence, 12 synthetic steps take place after the first fragment coupling, including the installation of a stereocenter. Drawing inspiration from Shiba's efforts and the promising attributes of streptothricin F, we have designed a total synthesis of streptothricin F that readily enables SAR exploration. Through the incorporation of late-stage fragment coupling, we believe independent modification of the three structural components of streptothricin F is possible. The intended design of our synthesis is to facilitate rapid, combinatorial-like library generation of streptothricin F analogs that are targeted to evade known resistance pathways and maintain, or further reduce, low toxicity. Herein, we report our highly convergent, diversity-enabling streptothricin F total synthesis consisting of 35 total steps, with a longest linear sequence of 19 steps and an overall yield of 0.40%.

**Scheme 3:**
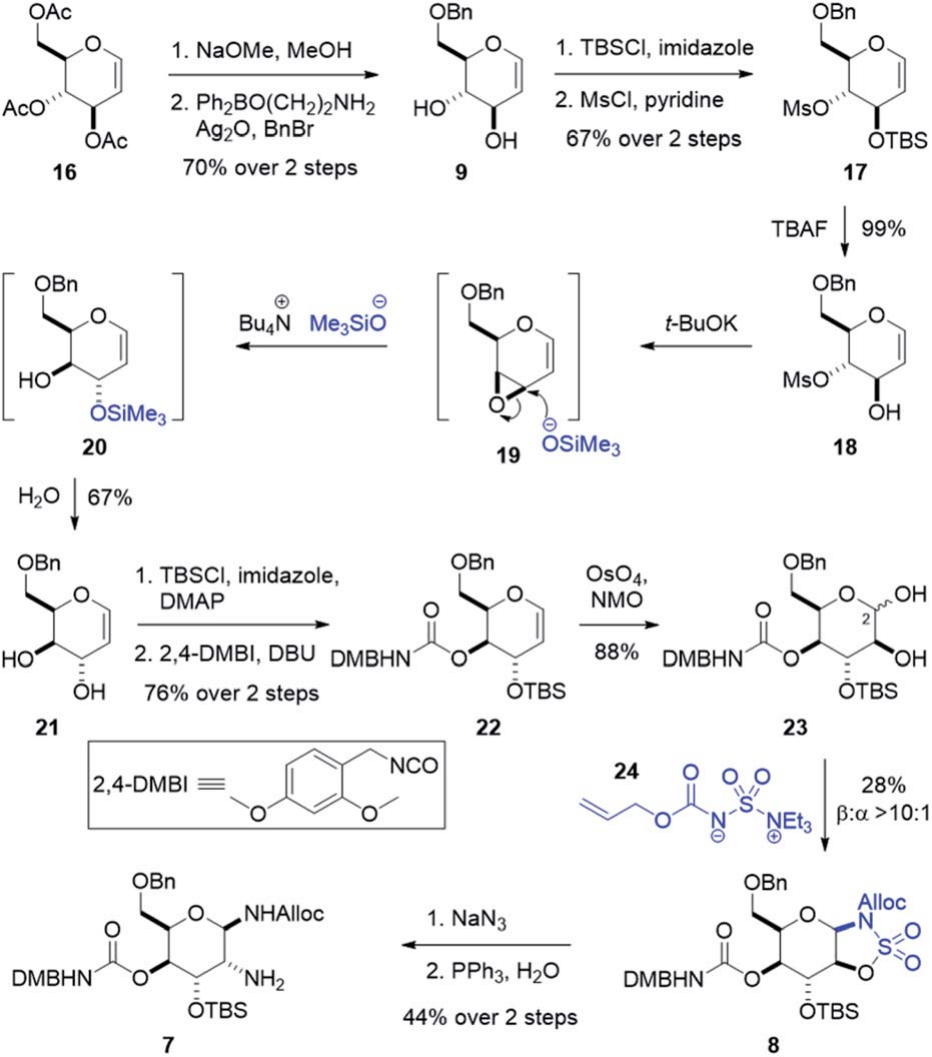
Synthesis of the gulosamine 7.

Retrosynthetically, our synthesis stems from two key disconnections at the C7 and C8 amines on the gulosamine core (Scheme 1). This approach hedged the production of streptothricin F (1) on a Lewis acid-catalyzed guanidine closure of thiourea 2, followed by global, stepwise deprotection. We envisioned thiourea 2 as the product of fragment couplings of isothiocyanate 3, partially protected β-lysine 6, and gulosamine 7. The first of these three fragments, isothiocyanate 3, can be generated through stereoselective azidation and tandem Staudinger–aza-Wittig thioisocyanation of lactam 4. Preparation of lactam 4 relied on nitromethylation of protected aspartic acid 5, followed by a formic acid-promoted deprotective-lactamization. Prior to coupling with isothiocyanate 3, partially protected β-lysine 6 and gulosamine 7 would be coupled at the C8 amine. We anticipated that access to gulosamine 7 could arise from nucleophilic ring-opening of β-sulfamidate 8. Burgess reagent-sulfamidation to obtain β-sulfamidate 8 was readily performed following stereoinversion and dihydroxylation of glucal sugar 9, which is obtained in decagram quantities from commercially available starting material.

Forward synthesis began with construction of the streptolidine moiety of streptothricin F (Scheme 2). Commercially available *N*-Cbz-l-aspartic acid 4-*tert*-butyl ester 5 was treated with carbonyl diimidazole producing an activated anhydride that reacted smoothly with a solution of excess nitromethane and stoichiometric *t*-BuOK^37^ to yield nitroketone 10.^38,39^ Diastereoselective reduction was carried out under Felkin–Ahn conditions, allowing hydride attack from the less hindered face of 10 resulting in >9 : 1 dr of the desired *erythro* (3*S*,4*R*) nitroalcohol 11.^39–41^ Nitro reduction through generation of nickel boride followed by immediate addition of di-*tert*-butyl dicarbonate in one pot produced dicarbamate 12 as a mixture of diastereomers.^42,43^ Warming in formic acid deprotected the previously installed Boc group and promoted lactamization to afford 13, where the diastereomeric mixture could be separated. Silylation of 13 with TBS triflate, followed by hydrogenation to remove benzyl carbamate and subsequent Boc protection produced dicarbamate lactam 4 in good yield over three steps. Deprotonation of 4 to lactam enolate 14 and treatment with trisyl azide provided α-azidolactam 15 as a single diastereomer.^44,45^ The complete selectivity of this reaction is likely directed through the synergistic steric effects of a congested top face of enolate 14 and the bulky nature of trisyl azide. A moderate yield of 15 was observed as a consequence of enolate formation and substrate stability. To avoid stepwise isothiocyanate formation and risk lactam hydrolysis, we looked to the tandem Staudinger–aza-Wittig method for a one pot conversion of azides to isothiocyanates.^46,47^ Adhering to this protocol, α-azidolactam 15 was treated with triphenylphosphine and excess carbon disulfide to yield isothiocyanate 3, with conservation of our lactam ring.^46,47^

**Scheme 4:**
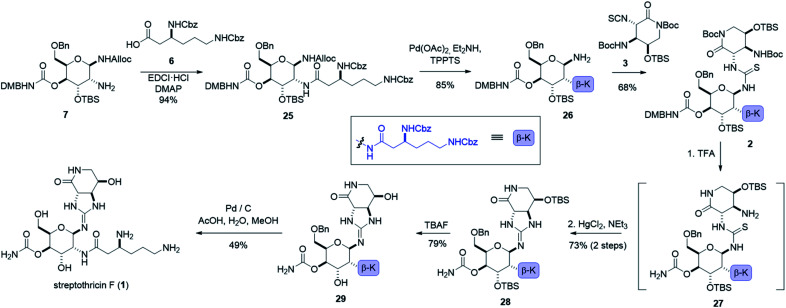
The convergent total synthesis of streptothricin F.

To assemble the gulosamine core, the first task was to generate the rare gulal sugar core from readily obtained starting material while enabling discrete alcohol functionalization (Scheme 3). Construction of gulal sugars has not been widely explored; however, two reliable methods have been reported. The first, pioneered by the Danishefsky laboratory, used a thio-phenol Ferrier-type/Mislow–Evans [2,3] sigmatropic rearrangement starting from tri-*O*-acetyl-d-galactal.^48–50^ The second method, reported by Crotti and co-workers, started from tri-*O*-acetyl-d-glucal and relied on a regioselective epoxide ring-opening to invert both the 9- and 10-position alcohols.^51,52^ Our route hewed more closely to the Crotti approach due to scalability and availability of inexpensive starting material. Deacylation of 16 and regioselective benzylation of the primary alcohol using Taylor's aminoethyl diphenylborinate catalyst^53^ and silver oxide as an activator gave benzyl glucal 9. Silylation with good regioselectivity and subsequent mesylation produced fully functionalized glucal 17. Treatment with TBAF yielded our stereoinversion precursor 18, which was treated with *t*-BuOK to form epoxide 19. When exposed to freshly prepared tetrabutylammonium trimethylsilanolate,^52^ epoxide 19 is selectively opened at the allylic position to give TMS alcohol 20 which is hydrolyzed upon work-up to give 6-*O*-benzyl-d-gulal 21. Silylation of 21 was performed with high regioselectivity and subsequent treatment with freshly prepared 2,4-dimethoxybenzyl isocyanate^49,54^ yielded the carbamoylated gulal 22.

To install our 1,2-diamine functionality on the gulal sugar, we employed Burgess reagent chemistry pioneered by Nicolaou and co-workers.^55^ Among other uses for the Burgess reagent, a method for preparation of 1,2-diamino sugars was reported, which we adopted for our synthesis. Dihydroxylation of gulal 22 gave diol 23 as an 8 : 1 mixture of anomers favoring the α-anomer, as implicated by the coupling constant of the anomeric proton (*J*_1,2_ = 11.3 Hz). Addition of the alloc-modified Burgess reagent 24 produced a disulfamate intermediate that reacts *via* C2 delivery to give β-sulfamidate 8 (>10 : 1 dr), (see ESI†).^55^ Ring opening of β-sulfamidate 8 with sodium azide followed by Staudinger reduction yielded gulosamine 7.^55,56^ To generate our C8 linkage, gulosamine 7 was coupled to partially protected β-lysine 6 (attained through homologation of α-lysine, see the ESI†) to give protected β-lysyl-gulosamine 25 (Scheme 4). Allyl carbamate deprotection of 25 proceeded through a catalytic allyl transfer mechanism to avoid anomerization. This mild deprotection method uses the palladium–TPPTS complex in tandem with diethyl amine as an allyl acceptor to generate β-lysyl-gulosamine 26.^55,57^ Coupling of β-lysyl-gulosamine 26 with previously prepared isothiocyanate 3 yielded thiourea 2, and completed our C7 linkage. Coupling of β-lysyl-gulosamine 26 with previously prepared isothiocyanate 3 yielded thiourea 2, and completed our C7 linkage.

At this stage (four steps from the completion of the synthesis), all streptothricin F stereocenters are set, and our three synthetic routes have converged. Treatment of thiourea 2 with TFA removed both Boc groups as well as the 2,4-dimethoxybenzyl moiety to yield thiourea 27 which was unstable to purification methods. The crude reaction mixture containing 27 was therefore directly cyclized to guanidine 28 through mercury(ii) chloride mediated desulfurization with triethylamine (for detailed optimization attempts and alternative approaches to prepare guanidine 28 see the ESI†). Two consecutive deprotections from guanidine 28 would complete our total synthesis. Silyl removal with TBAF provided partially deprotected streptothricin F 29 and under conditions similar to the Shiba total synthesis, benzyl and carboxybenzyl groups were removed in a single step through hydrogenolysis in acidic solvent.^35^ This hydrogenolysis provided streptothricin F (1) as an acetate salt; however, we desired to convert streptothricin F acetate to the sulfate to compare the activity and spectral data of synthetic streptothricin F more accurately to streptothricin F sulfate isolated from commercially available nourseothricin sulfate. Purified streptothricin F acetate was acidified to a pH of 2 in H_2_SO_4_, precipitated from methanol-diethyl ether, and collected *via* centrifugation.^58^

The comparison of antimicrobial activity of synthetic streptothricin F sulfate to isolated streptothricin F sulfate purified in our laboratory is shown in Table 1. Our purification method was adopted from Taniyama *et al.* with modifications to column length and flow rate.^6^ Upon loading a ∼300 mg sample of commercially available nourseothricin sulfate onto a glass column (150 × 2.4 cm) packed with Sephadex LH-20 afforded ∼75 mg of pure streptothricin F and ∼15 mg of pure streptothricin D, as well as mixed fractions. The microbes highlighted in Table 1 represent a diverse panel of Gram-positive and Gram-negative pathogens, many of which have been designated as either urgent or serious threats by the CDC and the WHO for their resistance capabilities,^59–61^ or are surrogates for CDC category A or B biothreat pathogens. Many of these species are also members of the so-called ESKAPE pathogens^62^ for which emerging antibiotic resistance threatens to eliminate effectiveness of all currently available antibiotics. Of note, both natural and synthetic streptothricin F were found to be active against the following: vancomycin-resistant *Staphylococcus aureus*; *Bacillus anthracis* (the cause of anthrax); multi-drug-resistant species of Gram-negative pathogens including the pan-resistant *K. pneumoniae*,^63^*Escherichia coli* expressing the colistin resistance gene mcr-1, and *Acinetobacter baumannii*; *Yersinia pestis* (the cause of bubonic plague); and *Francisella tularensis* (the cause of tularemia). However, there was low activity against *Burkholderia* and *Pseudomonas* strains tested. As a useful metric of comparison, the minimum inhibitory concentration (MIC) values for ampicillin, gentamicin, and tetraycline were 2, 0.5, and 2 μg mL^−1^ for *Escherichia coli*; 16, 0.5, and 8 μg mL^−1^ for *Acinetobacter baumannii*; and 128 (defined for benzylpenicillin), 0.125, and 0.5 μg mL^−1^ for methicillin-resistant *Staphylococci aureus*, respectively.^64^ MIC values for Gram-negative multidrug-resistant pathogens are typically much higher and are dependent upon the antibiotic and speicies of interest.^65–67^ Importantly, the MIC values of synthetic streptothricin F and streptothricin F purified from nourseothricin natural product were essentially identical within the expected experimental error, providing biological confirmation of the anticipated activity for our synthetic natural product.

**Table 1:** Antimicrobial activity comparison of synthetic and isolated streptothricin F

	Organism	Phenotype	MIC values (μg mL^−1^)	
			Isolated S-F	Synthetic S-F
Gram-positive	*S. aureus* ATCC 29213	Pan-susceptible control	4	4
	*S. aureus* (VRSA) NR46422	Vancomycin-resistant	4	8
	*S. aureus* (VRSA) NR49120	Vancomycin-resistant	4	16
	*S. aureus* (VRSA) NR46420	Vancomycin-resistant	4	16
Gram-negative	*A. baumannii* ATCC 17978	Pan-susceptible control	2	4
	*A. baumannii* MSRN 1450	Extensively-drug resistant	16	8
	*B. cenocepacia* clinical isolate K56-2 (ET-12 clone)	Cystic fibrosis pathogen	>64	>64
	*B. thailandensis* NR9908	Surrogate for biothreat *B. mallei*/*pseudomallei*	>64	>64
	*B. thailandensis* NR9909	Surrogate for biothreat *B. mallei*/*pseudomallei*	>64	>64
	*E. coli* ATCC 25922	Pan-susceptible control	1	2
	*E. coli* FDA-CDC 346 (MCR-1)	Plasmid-borne colistin resistance	1	4
	*B. anthracis* Sterne 9131	Surrogate for biothreat anthrax	8	8
	*F. tularensis* LVS	Surrogate for biothreat tularemia	0.125	0.125
	*K. pneumoniae* Nevada strain AR-0636	Pan-drug resistant	1	1
	*P. aeruginosa* ATCC 27853	Pan-susceptible control	64	>64
	*Y. pestis* Yokahama NR4693	Surrogate for biothreat bubonic plague	1	4

## Conclusions

The streptothricin scaffold has been overlooked by the synthetic chemistry community until now. Our disclosed work represents the second total synthesis of streptothricin F (1), and the first through a diversity-enabling convergent route. We have prepared streptothricin F in a longest linear sequence of 19 steps, with 35 total steps, and 0.40% overall yield. Additionally, our convergent total synthesis includes the ability to install practical, divergent synthetic steps and facilitate manipulations of each of the three independent structural moieties. We have shown that streptothricin F has great promise as a broadly active antibiotic scaffold ripe for optimization. Accordingly, through the fully developed, diversity-enabling total synthesis of streptothricin F, we are pursuing medicinal chemistry-guided analog generation of streptothricin F to explore synthetic analogs for potential therapeutic development.

## Author contributions

R. M. and J. E. K. conceptualized and supervised the work as well as edited the manuscript. M. G. D., B. C. M., M. K., K. P. S., and A. D. F designed and performed experiments. J. J. G. performed some NMR spectral acquisition. The first draft of the manuscript was written by M. G. D. and B. C. M. All authors contributed to manuscript review.

## Conflicts of interest

The authors declare no competing financial interest. The HP D300 digital dispenser and TECAN M1000 used for MIC analysis in Table 1 were provided for our use by TECAN (Morrisville, NC). TECAN had no role in study design, data collection/interpretation, manuscript preparation, or decision to publish.

## Supplementary Material

SC-013-D1SC06445B-s001
