# Supplementary material for: The convergent total synthesis and antibacterial profile of the natural product streptothricin F
Source: Chem Sci. 2022 Feb 25;13(12):3447–53. doi: 10.1039/d1sc06445b (PMC8943883; doi:10.1039/d1sc06445b)
Supplement: SC-013-D1SC06445B-s001 [file SC-013-D1SC06445B-s001.pdf]

Electronic Supplementary Material (ESI) for Chemical Science.
This journal is © The Royal Society of Chemistry 2022

The convergent total synthesis and antibacterial profile of the natural product streptothricin F

Matthew G. Dowgiallo[1], Brandon C. Miller[1], Mintesinot Kassu[1], Kenneth P. Smith[5,6], Andrew D. Fetigan[1], Jason J. Guo[1,3,4], James E. Kirby[5,6], Roman Manetsch[1,2,3]*

[1]Department of Chemistry and Chemical Biology, Northeastern University, Boston, MA, USA.

[2]Department of Pharmaceutical Sciences, Northeastern University, Boston, MA, USA.

[3]Center for Drug Discovery, Northeastern University, Boston, MA, USA.

[4]Barnett Institute for Chemical and Biological Analysis, Northeastern University, Boston, MA, USA.

[5]Department of Pathology, Beth Israel Deaconess Medical Center, Boston, MA, USA.

[6]Harvard Medical School, Boston, MA, USA

Supplementary Information

Table of Contents:

Supplementary figures………………………………………………………………….. S2

Streptothricin F model system synthesis ………………………………………………. S6

General experimental procedures………………………………………………………. S7

Synthetic procedures……………………………………………………………………. S9

Streptothricin isolation………………………………………………………………….. S45

Streptothricin F elemental analysis results……………………………………………… S46

Streptothricin F analytical data comparison: previously reported, synthetic, isolated…. S47

Antibacterial procedures………………………………………………………………... S50

Catalog of nuclear magnetic resonance spectra………………………………………… S51

References………………………………………………………………………………. S135

Supplementary figures:

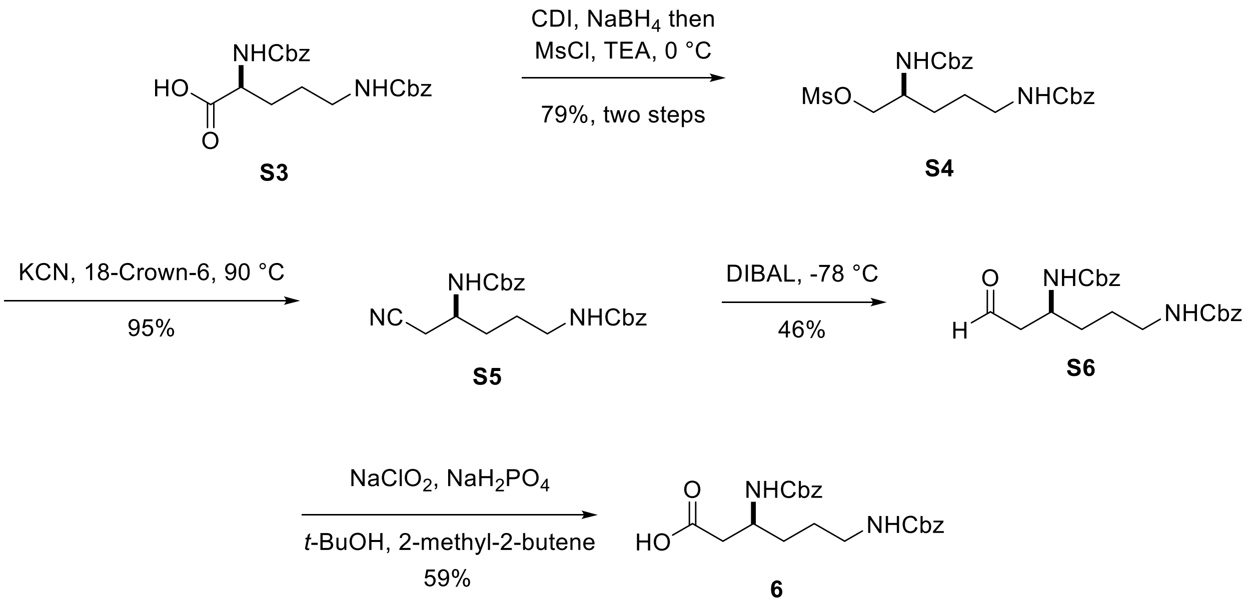

Figure S1: Synthesis of partially protected β-lysine **6**.

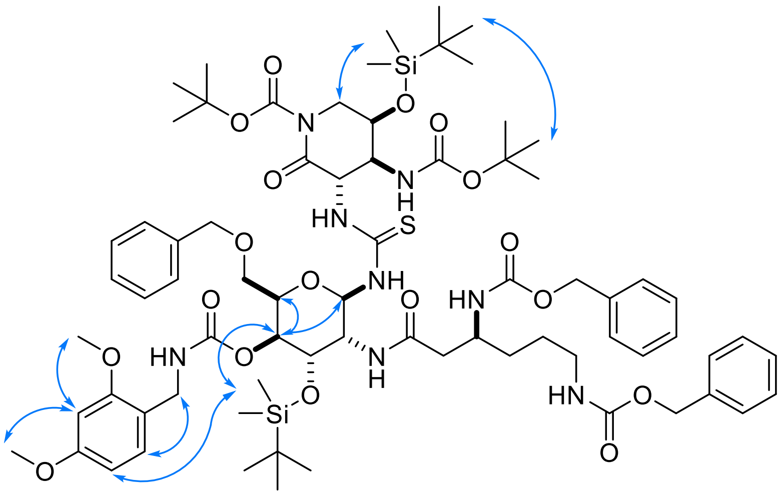

Figure S2: Key ROESY interactions of thiourea **2**.

Supplementary figures:

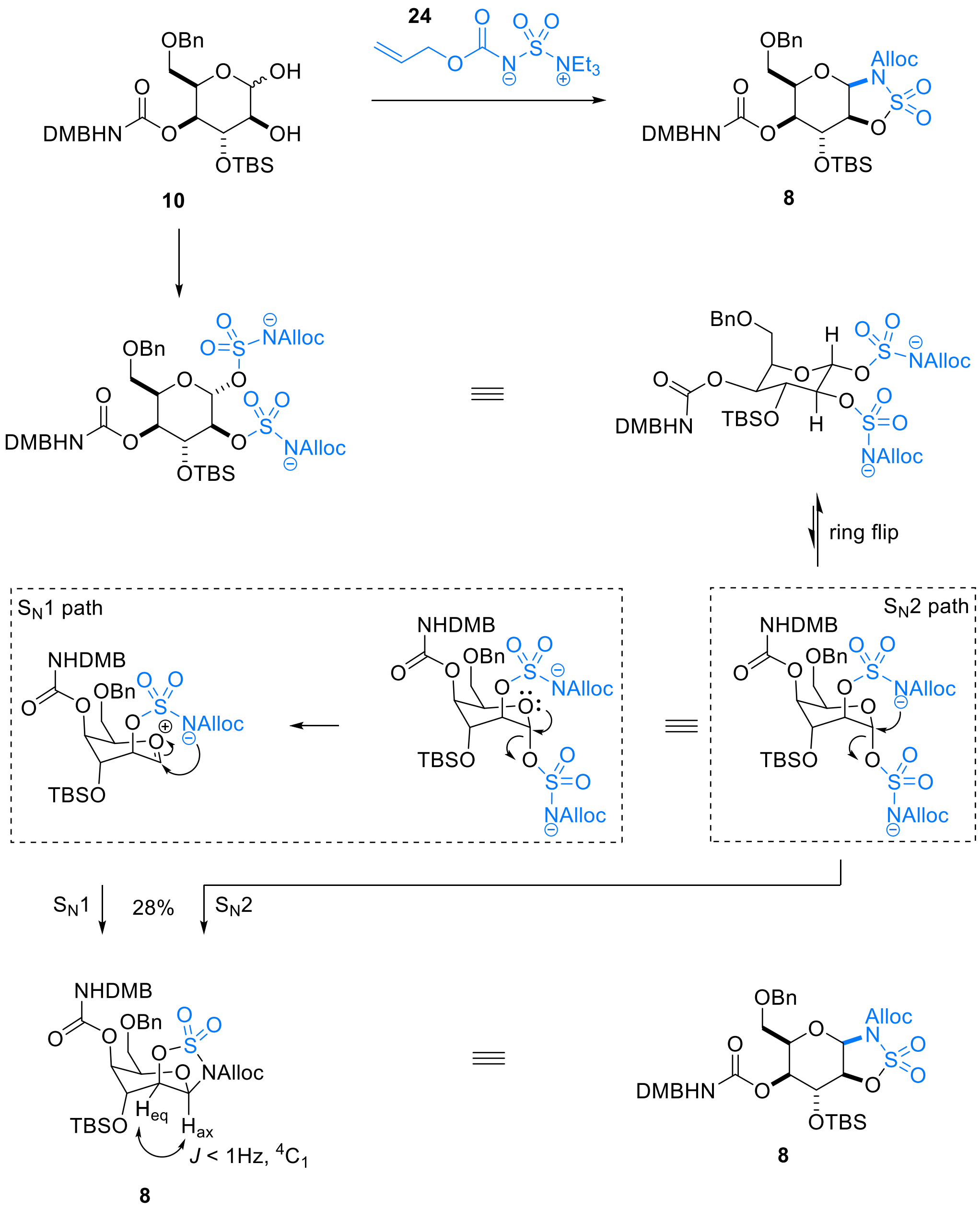

Figure S3. Burgess reagent sulfamidate reaction mechanism. Diol **10** is comprised of an 8:1 mixture of anomers with the equilibrium favoring the α-anomer suggested by the anomeric proton coupling constant (J = 11.3Hz). This also suggests a $^1C_4$ gulose configuration. The resulting sulfamidate **8** possesses a β-$^4C_1$ conformation where a $^1C_4$-$^4C_1$ transition is likely required to progress towards product formation. Reactivity analysis assumes the addition of Burgess reagent occurs first followed by ring flip. The remaining portion of the mechanism can occur either through an S_N2 route or S_N1 route. Through either path, a $^1C_4$-$^4C_1$ transition is energetically disfavored, resulting in 1,3-diaxial strain between most substituents.

Supplementary figures:

Table S1. Attempts towards guanidine **28** formation from gulosamine **27**. Each case the result of the attempt was concluded based off of TLC and LCMS analysis.

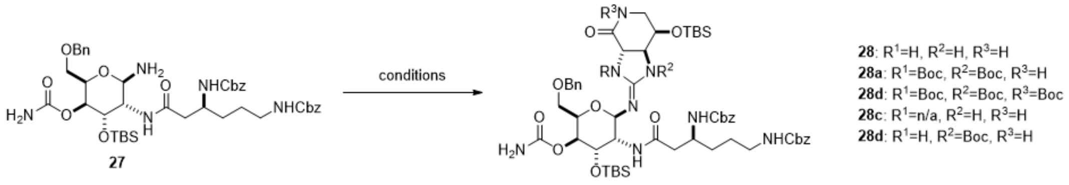

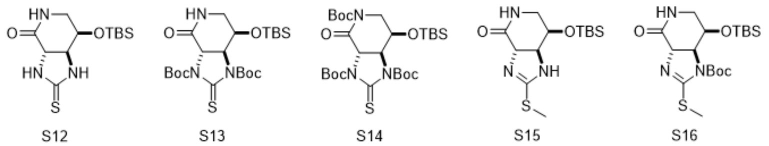

Trial	Thiourea	Solvent	Additive	Temperature	Time	Result
1	S12	DMF	$HgCl_2$, TEA	25 °C	1 d	NR
2	S13	DMF	$HgCl_2$, TEA	25 °C	1 d	NR
3	S14	DMF	$HgCl_2$, TEA	25 °C	1 d	NR
4	S15	DMF	-	80 °C	2 h	decomp
5	S16	DMF	-	25, 40, 60 °C	2 d – 2 h	NR - decomp
6	S16	MeOH	-	25, 40, 60 °C	2 d – 2 h	NR - decomp
7	S16	THF	-	25, 40, 60 °C	2 d – 2 h	NR - decomp
8	S16	DMF	DIPEA	25 °C	1 d	NR
9	S16	DMF	$HgCl_2$, TEA	25 °C	1 d	NR
10	S17	DMF	$HgCl_2$, TEA	25 °C	1 d	NR
11	S17	THF	-	25 °C	1 d	NR
12	S17	EtOH	AcOH	25 °C	1 d	NR

Supplementary figures:

Table S2. Reaction optimization attempts towards the formation of guanidine 28. DMC (dimethyl carbonate), EDCI, (1-Ethyl-3-(3-dimethylaminopropyl)carbodiimide). [α]The best yield was achieved for the overall transformation of thiourea **2** to protected guanidine **28** without the isolation of intermediate **27**.

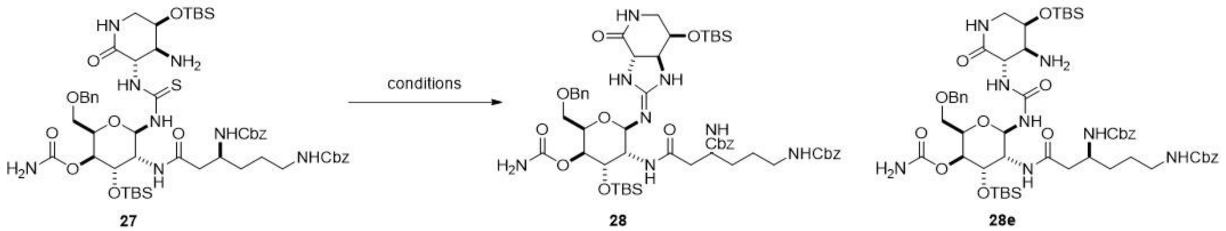

Entry	E⁺ / LA	Base (eq)	Solvent	Temperature	Time	Result (yield)
1	DMC	TEA (3)	MeCN	85 °C	12 h	28e
2	DMC	TEA (8)	MeCN	85 °C	12 h	28e
3	DMC	TEA (3)	THF	70 °C	12 h	28e
4	DMC	DBU (3)	MeCN	85 °C	12 h	28e
5	DMC	LiHMDS (3)	MeCN	0 °C - rt	2 h	28 (trace)
6	DMC	LiHMDS (2.5)	MeCN	0 °C - rt	12 h	28e
7	DMC	LiHMDS (3.5)	MeCN	0 °C - rt	2 h	Decomposition
8	EDCI	LiHMDS (3)	THF	-78 °C - rt	2 h	28 (trace)
9	$HgCl_2$	LiHMDS (3)	DMF	0 °C - rt	2 h	28 (trace)
10	$HgCl_2$	NEt_3 (3)	DMF	0 °C - rt	2 h	28 (57%)[α]

Streptothricin F model system synthesis:

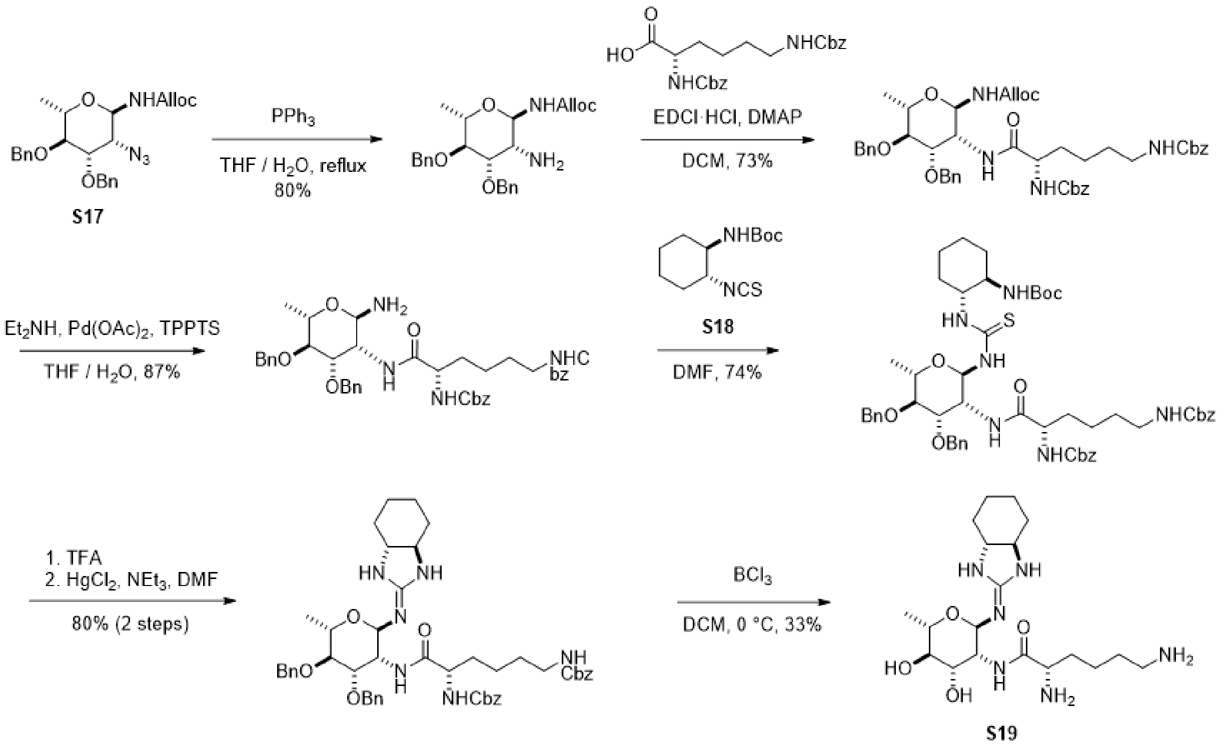

Figure S4: Reaction scheme for the synthesis of the streptothricin F model system. Full characterization data including ^{1}H NMR and ^{13}C NMR for **S17**, **S18** and **S19** is included in this supporting information document. Compound S19 was screened for antimicrobial activity and found to be inactive.

General experimental procedures:

Unless otherwise noted, all reactions were performed in flame-dried round bottom flasks under an argon atmosphere. Air and moisture sensitive liquids were transferred using stainless steel syringe or cannula. Unless otherwise noted, all commercial solvents and reagents were used as received. Tetrahydrofuran (THF) was distilled from benzophenone and sodium metal under a positive pressure argon atmosphere immediately before use. All other anhydrous solvents were purchased from VWR and stored under argon atmosphere. Sorbtec silica gel 60Å (particle size 40-63 μm) mesh was used for all flash column chromatography. Analytical thin layer chromatography (TLC) was performed on 0.25 mm silica gel 60 F_{254} precoated plates from EMD Millipore. Plates were visualized with ultraviolet light (254 nm) and/or treatment with aqueous solutions of cerium ammonium molybdate (CAM), potassium permanganate ($KMnO_4$) or ninhydrin stain followed by brief heating with a heat gun. Sephadex LH-20 size exclusion gel was purchased from GE Healthcare. Nourseothricin sulfate was purchased from Jena Bioscience. Polarimeter analysis was performed on a Jasco P-2000 Polarimeter using Spectra Manager 2.13.00 software. IR was recorded on a FT-IR spectrometer; thin film was formed in chloroform ($CHCl_3$) solution. Proton nuclear magnetic resonance (^{1}H NMR) spectra were recorded at ambient temperature on a 400 or 500 megahertz (MHz) Varian NMR spectrometer or a 600 MHz Bruker NMR spectrometer. Additionally, 2D experiments for some late-stage intermediates were recorded on a 700 MHz Bruker NMR spectrometer. All ^{1}H NMR experiments are reported in δ units, parts per million (ppm) downfield of trimethyl silane (TMS) and were measured relative to the residual proton signals of chloroform (δ 7.26), methanol (δ 3.31), acetone (δ 2.05), dimethylsulfoxide (δ 2.50), and water (δ 4.79). Data for ^{1}H NMR are reported as follows: chemicals shift (δ ppm), multiplicity (s = singlet, bs = broad singlet, d = doublet, dd = doublet of doublets, dt = doublet of triplets, t =

triplet, m = multiplet), integration, and coupling constant (Hz). Proton decoupled carbon nuclear magnetic resonance (^{13}C NMR) spectra were recorded at ambient temperature on a Varian NMR spectrometer operating at 100 or 125 MHz or a Bruker NMR spectrometer operating at 150 MHz. All ^{13}C NMR experiments are reported in δ units, ppm downfield from TMS and were measured relative to the residual carbon signals of chloroform (δ 77.1), methanol (δ 49.0), acetone (δ 29.8), and dimethylsulfoxide (δ 39.5). NMR data was analyzed by using MestReNova Software version 12.0.1. Low-resolution mass spectra were performed on an Agilent 6120 LC/MSD with electrospray ionization. High-resolution mass spectra were performed on a LTQ Orbitrap XL via loop injection with an RSLC nano pump. Elemental analysis was performed by Intertek Pharmaceutical Services in Whitehouse, New Jersey, United States.

Synthetic Procedures:

Gulosamine mimic **S17**

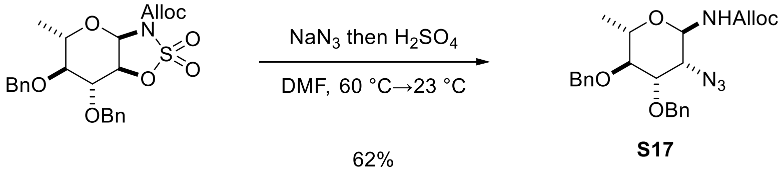

S17

To a stirring solution of the sulfamidate (3.44 g, 7.03 mmol) in anhydrous DMF (70 mL) was added sodium azide (2.28 g, 35.14 mmol) at rt under a stream of argon. The reaction was warmed to 60 °C and stirred for 5 h. LCMS analysis was performed to determine reaction progress and upon completion, the reaction mixture was cooled to rt and quenched with 10% sulfuric acid (70 mL). The reaction mixture was stirred for an additional 30 mins and then poured into brine (50 mL). The brine layer was extracted with ether (3 x 100 mL) and the combined organic extracts were washed with water (2 x 100 mL). The organic layer was dried over magnesium sulfate, filtered, and concentrated to a residue. The crude product was purified by silica gel chromatography with 10:1 toluene:EtOAc to yield 1.97 g (4.34 mmol, 62%) of **S17** as a white solid: mp 77-82 °C. R_f (7:1 toluene/EtOAc) = 0.43; ^{1}H NMR (500 MHz, Chloroform-*d*) δ 7.37 – 7.27 (m, 10H), 5.90 (ddt, *J* = 17.2, 10.4, 5.6 Hz, 1H), 5.44 – 5.41 (m, 1H), 5.31 (dq, *J* = 17.2, 1.5 Hz, 1H), 5.23 (dd, *J* = 10.6, 1.6 Hz, 1H), 4.77 (d, *J* = 11.2 Hz, 1H), 4.73 (d, *J* = 11.5 Hz, 1H), 4.66 (d, *J* = 11.7 Hz, 1H), 4.62 – 4.59 (m, 3H), 3.88 – 3.83 (m, 1H), 3.83 – 3.79 (m, 1H), 3.75 (p, *J* = 6.6 Hz, 1H), 3.48 (t, *J* = 7.3 Hz, 1H), 1.34 (d, *J* = 6.4 Hz, 3H); ^{13}C NMR (100 MHz, $CDCl_3$) δ 155.03, 137.92, 137.36, 132.24, 128.73, 128.65, 128.61, 128.27, 128.08, 118.43, 78.77, 78.70, 77.16, 74.67, 72.85, 70.13, 66.36, 60.64, 17.69. LCMS-ESI (*m* / *z*): $[M + Na]^+$ calcd for $C_{24}H_{28}N_4NaO_5$, 475.20 Da; found 475.1 Da.

Streptolidine mimic **S18**

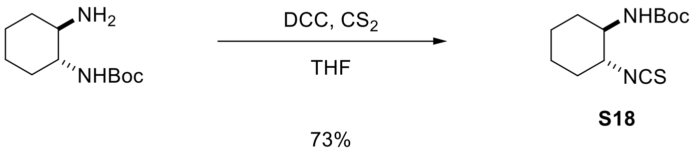

S18

To a stirring solution of *N*,*N′*-dicyclohexylcarbodiimide (0.439 g, 2.13 mmol) and carbon disulfide (0.918 mL, 15.19 mmol) in freshly distilled THF (10 mL) was added tert-butyl ((1R,2R)-2-aminocyclohexyl)carbamate (0.456 g, 2.13 mmol) in freshly distilled THF (14 mL) at 0 °C under a stream of argon over 30 mins. After the addition was complete, the reaction mixture was warmed to rt and stirred for 12 h until consumption of starting material was observed through TLC analysis. The reaction mixture was concentrated, diluted in ether (50 mL), filtered and the filtrate was concentrated to a residue. The crude product was purified by silica gel chromatography with 5:1 hexanes:EtOAc to yield 0.399 g (1.56 mmol, 73%) of **S18** as a white solid. R_f (2:1 hexanes/EtOAc) = 0.52; ^{1}H NMR (400 MHz, Chloroform-*d*) δ 4.55 (bs, 1H), 3.66 – 3.54 (m, 1H), 3.54 – 3.41 (m, 1H), 2.11 (d, J = 13.3 Hz, 1H), 2.06 – 1.97 (m, 1H), 1.75 – 1.64 (m, 2H), 1.63 – 1.57 (m, 1H), 1.47 (s, 9H), 1.39 – 1.33 (m, 1H), 1.30 – 1.22 (m, 2H). The ^{1}HNMR data of **S18** are in accordance with those reported previously.[S1] ^{13}C NMR (100 MHz, $CDCl_3$) δ 155.17, 132.06, 80.12, 60.46, 53.85, 32.35, 31.60, 28.56, 24.06, 23.63. LCMS-ESI (*m / z*): $[M + Na]^+$ calcd for $C_{12}H_{20}N_2O_2SNa$, 279.11 Da; found 279.3 Da.

Streptothricin mimic **S19**

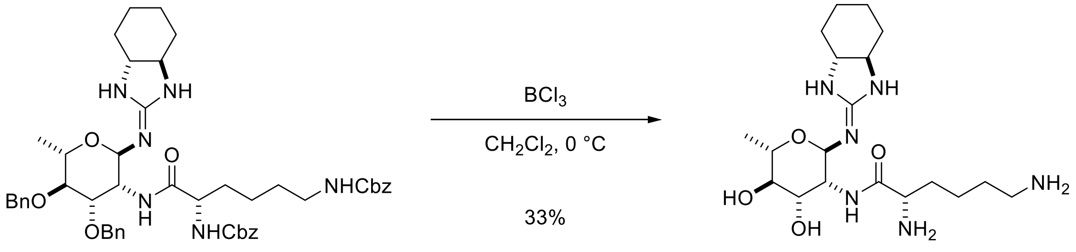

S19

To a stirring solution of protected S19 (0.111 g, 0.129 mmol) in anhydrous DCM (1.3 mL) was added a solution of boron trichloride (1M in DCM, 4.56 mmol) dropwise over 30 mins at -78 °C under a stream of argon. The reaction mixture was stirred at -78 °C for 1 h, warmed to 0 °C over 1 h, stirred at 0 °C and then warmed to rt. The reaction mixture was stirred at rt for 12 h and the consumption of starting material was observed through TLC analysis. The reaction mixture was cooled to 0 °C, quenched with MeOH (3 mL) warmed to rt and concentrated to a residue. The crude product was purified by reverse phase chromatography 100%-2% water/MeCN over 32 mins and pure fractions were freeze dried to yield 0.018 g (0.042 mmol, 33%) of **S19** as a white foam. ^{1}H NMR (500 MHz, Methanol-d_4) δ 5.01 (d, J = 1.8 Hz, 1H), 4.58 (dd, J = 4.7, 1.8 Hz, 1H), 4.15 – 4.09 (m, 1H), 3.72 (dd, J = 9.7, 4.7 Hz, 1H), 3.46 (dq, J = 9.4, 6.1 Hz, 1H), 3.31 – 3.28 (m, 2H), 3.26 (t, J = 9.6 Hz, 1H), 2.95 (t, J = 7.6 Hz, 2H), 2.16 (d, J = 11.5 Hz, 2H), 1.91 – 1.86 (m, 4H), 1.76 – 1.63 (m, 3H), 1.59 – 1.52 (m, 3H), 1.44 – 1.38 (m, 2H), 1.35 (d, J = 6.1 Hz, 3H); ^{13}C NMR (100 MHz, cd_3od) δ 172.12, 162.48, 81.03, 75.67, 73.25, 64.39, 54.57, 54.10, 40.39, 31.88, 30.70, 29.70, 28.21, 24.81, 22.69, 17.83. LCMS-ESI (m / z): $[M + H]^+$ calcd for $C_{19}H_{37}N_6O_4$, 413.29 Da; found 413.3 Da.

Nitroketone **10**:

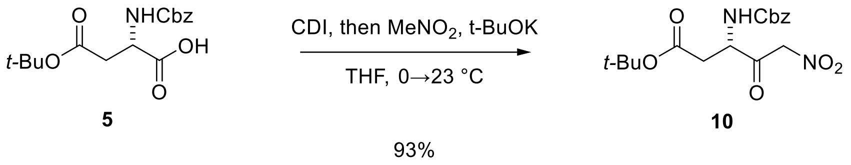

To a stirring solution of carbonyl diimidazole (2.60 g, 16.04 mmol) in freshly distilled THF (165 mL) was added a solution of *N*-Cbz-L-aspartic acid 4-*tert*-butyl ester **5** (4.94 g, 15.28 mmol) in freshly distilled THF (55 mL) at 23 °C under argon and stirred for 5 h. A solution of the nitro methane, potassium salt was prepared by diluting potassium *tert*-butoxide (1.89 g, 16.81 mmol) in freshly distilled THF (220 mL) which was placed in an ice bath at 0 °C. A solution of nitromethane (8.18 mL, 152.78 mmol) in THF (88 mL) was added to the potassium *tert*-butoxide solution at 0 °C and stirred for 30 mins at 0 °C, and then allowed to warm to 23 °C over 30 mins. The vessel containing the activated mixed anhydride was added to this solution via cannula and then stirred for 12 h at 23 °C. The reaction mixture was quenched with a 1 M HCl solution (400 mL), diluted with EtOAc (100 mL), and then extracted with EtOAc (3 x 100 mL). The combined organic layers were washed with brine (200 mL) and dried over Na_2SO_4. The solvent was removed to yield 4.6 g (12.6 mmol, 93%) of pure nitro ketone **11** as an amber solid: mp 88-91 °C. R_f (5% MeOH/CH_2Cl_2, CAM) = 0.36; $[\alpha]_D^{21}$ = -43.12 (*c* = 0.5, CH_2Cl_2); IR (thin film, cm^{-1}): 3366.61, 2979.48, 2934.84, 1717.10, 1562.06, 1510.10, 1455.75, 1369.22, 1316.74, 1243.81, 1155.68, 1053.23, 998.82, 844.13, 752.56, 698.51; ^{1}H NMR (500 MHz, $CDCl_3$) δ 7.42 – 7.33 (m, 5H), 5.85 (d, *J* = 8.6 Hz, 1H), 5.60 (d, *J* = 15.3 Hz, 1H), 5.49 (d, *J* = 15.4 Hz, 1H), 5.16 (s, 2H), 4.61 (dt, *J* = 8.4, 4.1 Hz, 1H), 3.06 (dd, *J* = 17.5, 4.2 Hz, 1H), 2.72 (dd, *J* = 17.5, 4.7 Hz, 1H), 1.43 (s, 9H); ^{13}C NMR (100 MHz, $CDCl_3$): δ 195.9, 170.4, 156.2, 135.7, 128.8, 128.7, 128.4, 82.9, 81.9, 77.5, 77.2, 76.8, 67.9, 55.7, 36.8, 28.0. HRMS-ESI (*m / z*): $[M + Na]^+$ calcd for $C_{17}H_{22}N_2O_7Na$, 389.1325 Da; found 389.1325 Da.

Nitroalcohol **11**:

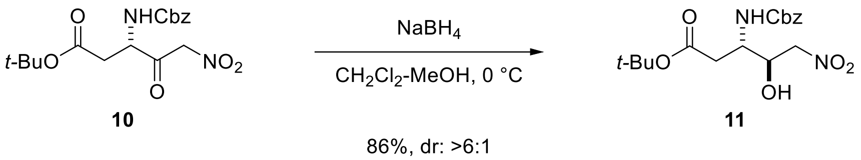

To a stirring solution of **10** (10.7 g, 29.23 mmol) in a solvent mixture of anhydrous CH_2Cl_2 (35 mL) and anhydrous MeOH (25 mL) at 0 °C was added sodium borohydride (1.11 g, 29.23 mmol) portionwise over 1 h under a stream of argon. The reaction mixture was allowed to stir for 30 mins at 0 °C following the final addition of sodium borohydride. While maintaining a temperature of 0 °C, the reaction mixture was quenched by the addition of 10% $KHSO_4$ solution, diluted with CH_2-Cl_2 (100 mL) and then extracted with CH_2Cl_2 (3 x 50 mL). The combined organic layers were washed with H_2O (100 mL), dried over sodium sulfate, and concentrated. The crude product was purified by silica gel chromatography eluting with 5:1 – 3:1 hexanes/EtOAc to yield 9.3 g (25.25 mmol, 86%) of the nitro alcohol **11** as a diasterometric mixture of a clear oil (dr: >6:1 **a**:**b**); R_f (2:1 hexanes/EtOAc) = 0.38; $[\alpha]_D^{23}$ = +1.88 (*c* = 1.03, CH_2Cl_2); IR (thin film, cm^{-1}): 3339.11, 1697.10, 1554.06, 1368.33, 1253.34, 1154.38, 1039.33, 697.86; ^1H NMR (500 MHz, CD_3OD) δ 7.38 – 7.27 (m, 5H), 5.12 (d, *J* = 12.3 Hz, 1H), 5.04 (d, *J* = 12.5 Hz, 1H), 4.62 (dd, *J* = 12.7, 2.6 Hz, 1H), 4.41 – 4.36 (m, 1H), 4.22 – 4.16 (m, 1H), 3.95 (td, *J* = 9.6, 3.9 Hz, 1H), 2.74 (dd, *J* = 15.4, 4.1 Hz, 1H), 2.38 (dd, *J* = 15.4, 9.9 Hz, 1H), 1.40 (s, 9H); ^{13}C NMR (100 MHz, CD_3OD) δ 172.1, 158.2, 138.1, 129.5, 129.0, 128.9, 82.1, 80.2, 72.2, 67.7, 52.6, 38.3, 28.2. HRMS-ESI (*m / z*): $[M + Na]^+$ calcd for $C_{17}H_{24}N_2O_7Na$, 391.1481 Da; found 391.1478 Da.

Dicarbamate **12**:

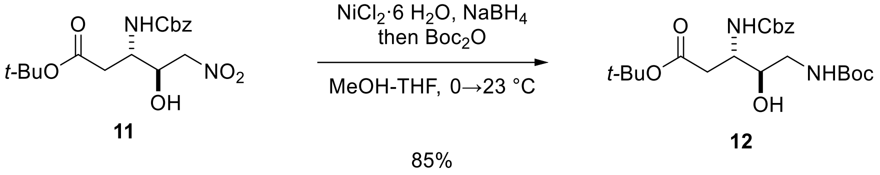

To a stirring solution of **11** (10.85 g, 29.46 mmol) and nickel (II) chloride hexahydrate (7.00 g, 29.46 mmol) in MeOH (150 mL) and THF (150 mL) was added sodium borohydride (5.57 g, 147.29 mmol) portionwise over 10 mins at 0 °C under a stream of argon. The reaction mixture was stirred for 30 mins and reaction progress was determined by TLC analysis. Upon consumption of the starting material, di-*tert*-butyl dicarbonate (19.29 g, 88.37 mmol) was added at 0 °C, stirred at 0 °C for 15 mins, warmed to rt and then stirred for 12 h. A 10% solution of sodium bicarbonate (200 mL) was added and the reaction mixture was filtered over celite, concentrated to half its volume and added to H_2O (100 mL). The aqueous layer was extracted with EtOAc (3 x 100 mL) and the combined organic extracts were washed with brine (200 mL). The organic layer was dried over sodium sulfate, filtered, and concentrated to a residue. The crude product was purified by silica gel chromatography with 5:1 – 3:1 hexanes/EtOAc to yield 11.0 g (25.08 mmol, 85%) of a diasterometric mixture of **12** as a clear oil. R_f (1:1 hexanes/EtOAc, CAM) = 0.50; $[\alpha]_D^{25}$ = +25.53 (*c* = 1.3, CH_2Cl_2); IR (thin film, cm^{-1}): 3339.78, 2977.37, 1691.67, 1511.36, 1455.14, 1392.20, 1366.47, 1248.80, 1157.12, 1040.71, 845.51, 738.57, 697.65; ^1H NMR (500 MHz, $CDCl_3$) δ 7.37 – 7.33 (m, 5H), 5.68 (d, *J* = 9.1 Hz, 1H), 5.46 (bs, 1H), 5.09 (s, 2H), 3.95 – 3.88 (m, 1H), 3.65 – 3.53 (m, 2H), 3.02 (dt, *J* = 14.9, 4.7 Hz, 1H), 2.67 (dd, *J* = 16.4, 5.8 Hz, 1H), 2.61 – 2.54 (m, 1H), 1.44 (s, 9H), 1.42 (s, 9H). ^{13}C NMR (125 MHz, $CDCl_3$) δ 171.6, 157.8, 156.5, 136.3, 128.5, 128.1, 128.0, 81.2, 79.8, 73.0, 66.8, 50.2, 43.2, 36.2, 28.4, 27.9. HRMS-ESI (*m / z*): $[M + Na]^+$ calcd for $C_{22}H_{34}N_2O_7Na$, 461.2264 Da; found 461.2260 Da.

Lactam **13**:

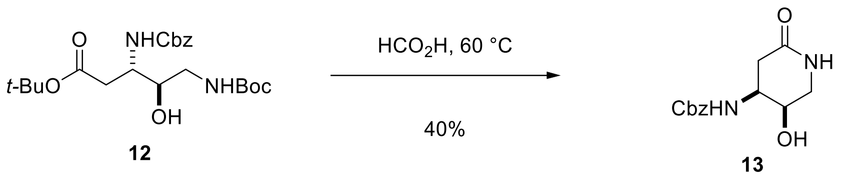

A stirring solution of **12** (5.33 g, 12.15 mmol) in formic acid (118 mL, 3.11 mol) was heated to 60 °C for 8 h under argon. The solvent was removed under reduced pressure, and the residue was concentrated in heptane (3 x 100 mL) to remove residual formic acid. The residue was diluted in MeOH (100 mL) and sodium carbonate (12.88 g, 121.54 mmol) was added, and the reaction mixture was stirred for 1 h. The reaction mixture was diluted in CH_2Cl_2 (100 mL), filtered through a celite pad, and concentrated to a residue. The crude product was purified by silica gel chromatography with 10% MeOH/EtOAc to yield 1.30 g (4.92 mmol, 40%) of **13** as a white solid: mp 166-171 °C. R_f (12% MeOH/EtOAc, CAM) = 0.28; $[\alpha]_D^{28}$ = -22.17 (*c* = 0.1, MeOH); IR (thin film, cm^{-1}): 3307.14, 2922.42, 1699.35, 1649.39 1540.04, 1495.69, 1455.16, 1333.15, 1257.42, 1042.46, 738.55, 697.28; ^1H NMR (500 MHz, CD_3OD) δ 7.40 – 7.27 (m, 5H), 5.09 (s, 2H), 4.09 (q, *J* = 2.7 Hz, 1H), 4.02 – 3.94 (m, 1H), 3.44 (dd, *J* = 13.4, 3.0 Hz, 1H), 3.28 (dd, *J* = 13.4, 2.8 Hz, 1H), 2.52 (dd, *J* = 17.4, 11.1 Hz, 1H), 2.45 (dd, *J* = 17.4, 6.6 Hz, 1H); ^{13}C NMR (125 MHz, $(CD_3)_2SO$) δ 169.0, 155.5, 137.1, 128.4, 127.9, 127.9, 65.4, 63.4, 48.9, 46.1, 32.9. HRMS-ESI (*m / z*): $[M + Na]^+$ calcd for $C_{13}H_{16}N_2O_4Na$, 287.1007 Da; found 287.1005 Da.

Silyl lactam **S1**:

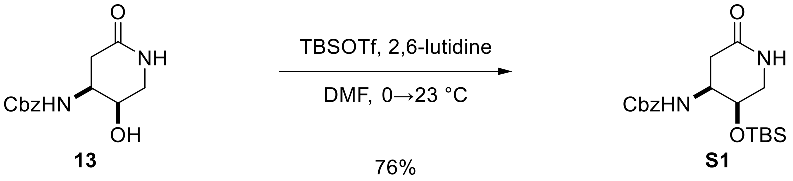

To a stirring solution of **13** (1.34 g, 5.05 mmol) in anhydrous DMF (21.9 mL) was added 2,6-lutidine (2.37 mL, 20.21 mmol) and *tert*-butyldimethylsilyl trifluoromethanesulfonate (2.37 mL, 10.10 mmol) at 0 °C under a stream of argon. After 20 mins, the reaction mixture was warmed to rt and stirred for 12 h. Upon consumption of starting material determined by TLC, the reaction was quenched with the addition of brine (50 mL). Ether (50 mL) was added to the mixture and used to extract the organic layer (3 x 50 mL), and then the organic extracts were washed with H_2O (2 x 50 mL) and brine (50 mL). The combined organic layers were dried over sodium sulfate, filtered, and concentrated to a residue. The crude product was purified by silica gel chromatography with 2:1 – 10:1 EtOAc/hexanes to yield 1.46 g (3.86 mmol, 76%) of protected lactam **S1** as a white solid: mp 57-59 °C. R_f (10:1 EtOAc/hexanes, CAM) = 0.17; $[\alpha]_D^{22}$ = -26.40 (*c* = 1.0, CH_2Cl_2); IR (thin film, cm^{-1}): 2927.82, 1705.17, 1664.49, 1494.26, 1336.45, 1258.41, 1088.70, 1047.16, 835.69; ^{1}H NMR (500 MHz, $CDCl_3$) δ 7.39 – 7.30 (m, 5H), 6.06 (bs, 1H), 5.10 (s, 2H), 4.83 (d, *J* = 7.9 Hz, 1H), 4.19 – 4.15 (m, 1H), 4.08 – 3.99 (m, 1H), 3.44 (d, *J* = 12.5 Hz, 1H), 3.23 (dt, *J* = 13.0, 3.0 Hz, 1H), 2.53 (dd, *J* = 17.0, 6.3 Hz, 1H), 2.46 (dd, *J* = 17.0, 11.5 Hz, 1H), 0.87 (s, 9H), 0.05 (d, *J* = 7.5 Hz, 6H); ^{13}C NMR (100 MHz, $CDCl_3$) δ 170.5, 155.6, 136.3, 128.7, 128.4, 128.3, 67.1, 65.7, 49.2, 47.2, 33.2, 25.8, 18.1, -4.6, -4.8. HRMS-ESI (*m / z*): [M + Na]$^+$ calcd for $C_{19}H_{30}N_2O_4SiNa$, 401.1872 Da; found 401.1868 Da. ^{1}H assignments are included along the f2 projection for the 1H-1H COSY spectrum.

Amino-silyl lactam **S2**:

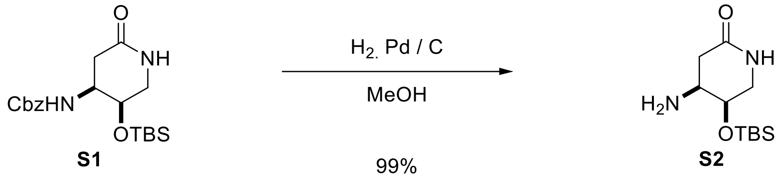

To a stirring solution of **S1** (1.37 g, 3.63 mmol) in anhydrous MeOH (1.77 mL) was added palladium on carbon (Pd 10% on carbon, 0.386 g). The reaction vessel was evacuated and purged with argon gas (7x), evacuated and purged with hydrogen gas (7x), and then stirred for 12 h under a hydrogen atmosphere (balloon). The reaction mixture was filtered over a celite bed, concentrated to 0.887 g (3.63 mmol, 99%) of **S2** as a white solid and used directly in the next step without further purification. mp 125-127 °C. R_f (10:1 EtOAc/hexanes, CAM) = 0.20; $[\alpha]_D^{25}$ = -24.47 (*c* = 0.3, MeOH); IR (thin film, cm^{-1}): 3269.06, 2928.20, 2855.92, 1641.22, 1492.71, 1343.97, 1250.63, 1099.55, 1046.55, 991.50, 937.86, 832.82, 773.80, 705.88, 489.40; ^{1}H NMR (500 MHz, CD_3OD) δ 4.08 (q, *J* = 3.1 Hz, 1H), 3.36 (dd, *J* = 13.2, 3.1 Hz, 1H), 3.28 (dd, *J* = 13.2, 3.8 Hz, 1H), 3.15 (ddd, *J* = 9.7, 5.9, 2.1 Hz, 1H), 2.49 (dd, *J* = 17.5, 5.8 Hz, 1H), 2.29 (dd, *J* = 17.5, 9.7 Hz, 1H), 0.93 (s, 9H), 0.16 (s, 3H), 0.15 (s, 3H); ^{13}C NMR (125 MHz, $CDCl_3$) δ 171.7, 68.2, 49.3, 45.9, 37.0, 25.7, 18.1, -4.5, -4.7. HRMS-ESI (*m / z*): $[M + H]^+$ calcd for $C_{11}H_{25}N_2O_2Si$, 245.1685 Da; found 245.1681 Da.

Dicarbamate lactam **4**:

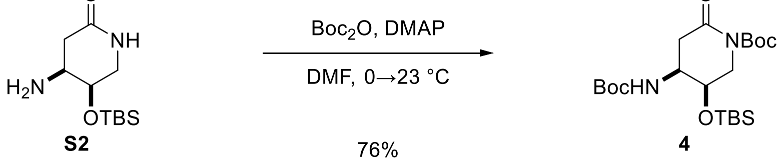

To a stirring solution of **S2** (0.725 g, 2.97 mmol) in anhydrous DMF (14.8 mL) was added 4-dimethylaminopyridine (0.036 g, 0.297 mmol) at 0 °C under a stream of argon. The reaction mixture was stirred for 10 mins before di-*tert*-butyl dicarbonate (1.29 g, 5.93 mmol) was added. The reaction mixture was warmed to rt and stirred for 3 h, cooled again to 0 °C, then di-*tert*-butyl dicarbonate (0.647 g, 2.97 mmol) was added. The reaction mixture was warmed to rt, stirred for 3 h and then consumption of starting material was observed through TLC analysis. The reaction was quenched with the addition of brine (50 mL). EtOAc (50 mL) was added to the mixture and used to extract the organic layer (3 x 30 mL), and then the organic extracts were washed with H_2O (2 x 50 mL) and brine (50 mL). The combined organic layers were dried over sodium sulfate, filtered, and concentrated to a residue. The crude product was purified by silica gel chromatography with 5:1 hexanes:EtOAc to yield 1.00 g (2.25 mmol, 76%) of **4** as an off-white solid: mp 107-110 °C. R_f (2:1 hexanes:EtOAc, $KMnO_4$) = 0.50; $[\alpha]_D^{27}$ = -22.66 (c = 0.5, CH_2Cl_2); IR (thin film, cm^{-1}): 2930.53, 1774.16, 1714.63, 1501.52, 1391.05, 1367.40, 1298.34, 1253.20, 1158.03, 1095.42, 1070.34, 974.58, 837.56, 778.60; ^1H NMR (500 MHz, $CDCl_3$) δ 4.57 (d, J = 8.6 Hz, 1H), 4.22 – 4.19 (m, 1H), 3.99 – 3.94 (m, 1H), 3.92 (dd, J = 13.8, 3.3 Hz, 1H), 3.45 (dd, J = 13.8, 2.1 Hz, 1H), 2.68 (dd, J = 16.9, 6.4 Hz, 1H), 2.57 (dd, J = 17.0, 11.5 Hz, 1H), 1.51 (s, 9H), 1.44 (s, 9H), 0.88 (s, 9H), 0.10 (s, 3H), 0.09 (s, 3H); ^{13}C NMR (125 MHz, $CDCl_3$) δ 168.8, 155.0, 152.5, 83.4, 80.1, 66.1, 50.5, 48.6, 36.4, 28.4, 28.1, 25.7, 18.1, -4.7, -4.8. HRMS-ESI (m / z): $[M + Na]^+$ calcd for $C_{21}H_{40}N_2O_6SiNa$, 467.2553 Da; found 467.2545 Da.

α-azidolactam **15**:

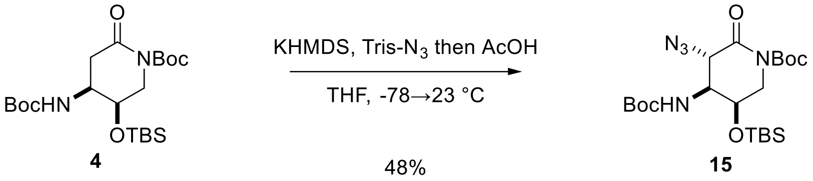

To a flame dried, round bottom flask was added freshly distilled THF (14 mL) under argon which was then cooled to -78 °C. Potassium bis(trimethylsilyl)amide (1 M in THF, 6.75 mmol) was added to the round bottom flask and stirred. A separate solution was prepared by diluting **4** (1.00 g, 2.25 mmol) in freshly distilled THF (5.6 mL) and then cooling to -78 °C in a pear-shaped flask under argon. The solution of **4** was then added dropwise via cannula to the potassium bis(trimethylsilyl)amide solution and continued stirring for 40 mins. A separate solution was prepared by diluting 2,4,6-triisopropylbenzenesulfonyl azide (1.39 g, 4.5 mmol) in freshly distilled THF (8.7 mL) and then cooled to -78 °C in a pear-shaped flask under argon. The newly prepared solution was then added via cannula to the reaction mixture and allowed to stir for 2 mins. The reaction was then quenched with acetic acid (0.592 mL, 10.35 mmol) at - 78 °C, the cold bath was removed, and the reaction mixture was warmed to room temperature over 3 h. A saturated solution of sodium bicarbonate (40 mL) was added and then EtOAc was used to extract the organic layer (3 x 50 mL). The organic extracts were washed with brine, dried over sodium sulfate, filtered, and concentrated to a residue. The crude product was purified by silica gel chromatography with 9:1 hexanes/EtOAc to yield 0.522 g (1.07 mmol, 48%) of azido lactam **15** as a white solid: mp 135-139 °C. R_f (4:1 hexanes/EtOAc, $KMnO_4$) = 0.42; $[\alpha]_D^{27}$ = -68.95 (c = 0.2, CH_2Cl_2); IR (thin film, cm^{-1}): 2930.81, 2857.92, 2112.43, 1776.10, 1723.43, 1506.43, 1472.32, 1392.00, 1368.49, 1286.21, 1256.70, 1157.04, 1067.35, 976.29, 837.92, 780.01; ^{1}H NMR (500 MHz, $CDCl_3$) δ 4.75 (d, J = 7.9 Hz, 1H), 4.32 – 4.28 (m, 1H), 4.13 (d, J = 11.2 Hz, 1H), 3.90 (dd, J = 13.8, 3.3 Hz, 1H),

3.77 (t, J = 9.4 Hz, 1H), 3.47 (dd, J = 13.9, 2.0 Hz, 1H), 1.52 (s, 9H), 1.46 (s, 9H), 0.89 (s, 9H), 0.11 (s, 3H), 0.09 (s, 3H); ^{13}C NMR (125 MHz, $CDCl_3$) δ 167.6, 155.1, 152.2, 84.2, 80.5, 66.4, 62.4, 54.0, 49.9, 28.4, 28.0, 25.7, 18.1, -4.8, -4.9. HRMS-ESI (*m / z*): $[M + Na]^+$ calcd for $C_{21}H_{40}N_2O_6SiNa$, 508.2567 Da; found 508.2565 Da. ^{1}H assignments are included along the f2 projection for the 1H-1H COSY spectrum.

Isothiocyanate **3**:

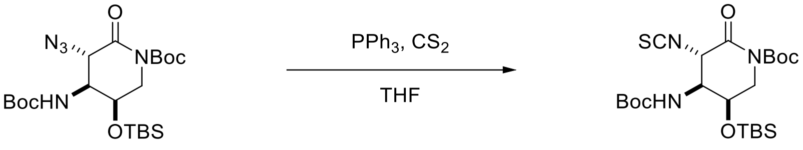

15 3

To a stirring solution of **15** (0.279 g, 0.574 mmol) in freshly distilled THF (1.15 mL) was added carbon disulfide (0.288 mL, 4.6 mmol) and triphenylphosphine (0.151 g, 0.574 mmol) at rt. The reaction vessel was sealed with a glass stopper and stirred at rt for 12 h when consumption of starting material was observed through TLC analysis. The reaction mixture was concentrated to a residue and purified by silica gel chromatography with 5% acetone, 5% CH_2Cl_2 in hexanes to yield 0.195 g (0.389 mmol, 68%) of **3** as a colorless gum. R_f (18:1:1 hexanes/acetone/CH_2Cl_2, $KMnO_4$) = 0.11; $[\alpha]_D^{28}$ = -37.47 (c = 0.4, CH_2Cl_2); IR (thin film, cm^{-1}): 2930.00, 2857.64, 2056.47, 1776.99, 1719.43, 1500.65, 1391.79, 1368.15, 1290.56, 1256.15, 1152.22, 1124.77, 1057.84, 969.41, 838.32, 779.85, 491.38; ^{1}H NMR (500 MHz, $CDCl_3$) δ 4.79 (d, J = 8.7 Hz, 1H), 4.60 (d, J = 11.5 Hz, 1H), 4.29 – 4.26 (m, 1H), 4.08 (t, J = 9.5 Hz, 1H), 3.87 (dd, J = 13.9, 3.2 Hz, 1H), 3.50 (dd, J = 14.0, 2.3 Hz, 1H), 1.51 (s, 9H), 1.47 (s, 9H), 0.88 (s, 9H), 0.12 (s, 3H), 0.09 (s, 3H); ^{13}C NMR (125 MHz, $CDCl_3$) δ 164.8, 154.8, 152.3, 141.1, 84.4, 80.9, 66.6, 60.7, 54.7, 49.9, 28.5, 28.0, 25.7,

18.0, -4.8, -4.9. HRMS-ESI (*m / z*): [M + Na]$^+$ calcd for $C_{22}H_{39}N_3O_6SSiNa$, 524.2227 Da; found 524.2225 Da. ^{1}H assignments are included along the f2 projection for the 1H-1H COSY spectrum.

Mesylate **S4**:

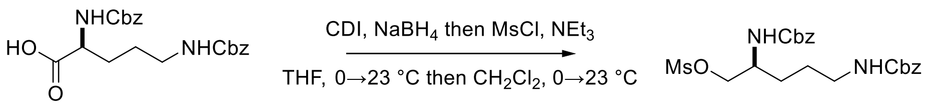

S3 **S4**

79%, two steps

To a stirring solution of (*S*)-2,5-bis(((benzyloxy)carbonyl)amino)pentanoic acid **S3** (0.200 g, 0.50 mmol) in freshly distilled THF (1.7 mL) was added carbonyl diimidazole (0.081 g, 0.50 mmol) and the solution was stirred at room temperature for 30 mins under a stream of argon. The reaction mixture was cooled to 0 °C then an aqueous solution of $NaBH_4$ (1.5 M, 0.50 mmol) was added dropwise over 10 mins. The solution was brought to room temperature and stirred for 1 hour. The reaction mixture was then neutralized with 4 M HCl (10 mL) and extracted with EtOAc (3 x 10 mL). The combined organic extracts were washed with brine (50 mL), dried over sodium sulfate, and concentrated under reduced pressure to yield the crude alcohol. To a solution of the crude alcohol (0.193 g) in anhydrous CH_2Cl_2 (2.5 mL) was added triethylamine (0.108 mL, 0.75 mmol). The mixture was cooled to 0 °C then methanesulfonyl chloride (0.120 mL, 1.55 mmol) was added dropwise. The solution was then brought to rt and stirred for 12 h under a stream of argon. The reaction mixture was quenched with the addition of H_2O (10 mL) and extracted with CH_2Cl_2 (3 x 25 mL). The organic layer was dried over sodium sulfate, filtered, and concentrated to a residue. The crude product was purified by silica gel chromatography with 1:1 hexanes/EtOAc to yield 0.183 g (0.394 mmol, 79% over 2 steps) of **S4** as a white solid: mp 105-106 °C. R_f (2:1 EtOAc/hexanes, CAM) = 0.46; $[\alpha]_D^{29}$ = -16.96 (*c* = 0.3, CH_2Cl_2); IR (thin film, cm^{-1}): 3320.66, 2933.70, 1697.00, 1527.98, 1454.78, 1352.22, 1248.41, 1173.73, 1025.92, 960.74, 820.67, 740.67, 698.10, 528.46; ^{1}H NMR (500 MHz, $CDCl_3$) δ 7.37 – 7.30 (m, 10H), 5.11 – 5.07 (m, 4H), 4.90

(bs, 1H), 4.24 (dd, J = 10.6, 3.8 Hz, 1H), 4.17 (dd, J = 10.5, 4.1 Hz, 1H), 3.95 – 3.88 (m, 1H), 3.23 – 3.16 (m, 2H), 2.95 (s, 3H), 1.63 – 1.51 (m, 4H); ^{13}C NMR (125 MHz, $CDCl_3$) δ 156.7, 156.2, 136.5, 136.3, 128.5, 128.5, 128.2, 128.1, 128.0, 70.9, 66.9, 66.6, 49.9, 40.4, 37.1, 27.9, 26.2. HRMS-ESI (*m / z*): $[M + Na]^+$ calcd for $C_{22}H_{28}N_2O_7SNa$, 487.1515 Da; found 487.1509 Da.

Nitrile **S5**:

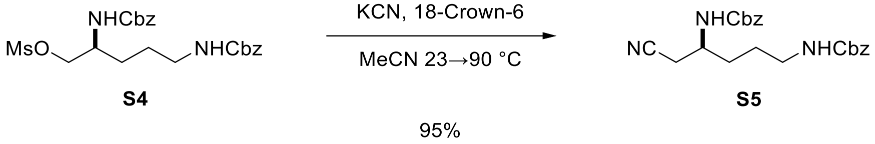

To a stirring solution of **S4** (3.27 g, 7.03 mmol) in MeCN (88 mL) was carefully added 18-crown-6-ether (2.23 g, 8.4 mmol) and (KCN (1.37 g, 21.1 mmol) at rt. The reaction was warmed to 90 °C and stirred for 1 h then quenched with a saturated solution of sodium bicarbonate (100 mL) and extracted with EtOAc (4 x 50 mL). The organic layer was washed with brine (100 mL), dried over sodium sulfate, filtered, and concentrated to a residue. The crude product was purified by silica gel chromatography with 5:1 hexanes:EtOAc to yield 2.65 g (6.70 mmol, 95%) of **S5** as a white solid: mp 78-79 °C. R_f (2:1 hexanes/EtOAc, CAM) = 0.74; $[\alpha]_D^{29}$ = -30.51 (c = 0.6, CH_2Cl_2); IR (thin film, cm^{-1}): 3321.76, 2949.49, 1693.77, 1528.55, 1454.28, 1252.72, 1136.00, 1026.37, 739.04, 697.61; ^{1}H NMR (500 MHz, $CDCl_3$) δ 7.38 – 7.30 (m, 10H), 5.12 – 5.08 (m, 4H), 4.86 (t, J = 6.2 Hz, 1H), 3.94 – 3.86 (m, 1H), 3.26 – 3.17 (m, 2H), 2.72 (dd, J = 16.9, 5.5 Hz, 1H), 2.53 (dd, J = 16.8, 4.3 Hz, 1H), 1.64 – 1.51 (m, 4H); ^{13}C NMR (100 MHz, $CDCl_3$) δ 156.6, 155.9, 136.5, 136.1, 128.6, 128.6, 128.3, 128.2, 128.1, 117.4, 67.0, 66.7, 47.7, 40.3, 30.4, 26.5, 23.9. HRMS-ESI (*m / z*): $[M + Na]^+$ calcd for $C_{22}H_{25}N_3O_4Na$, 418.1743 Da; found 418.1741 Da.

Aldehyde **S6**:

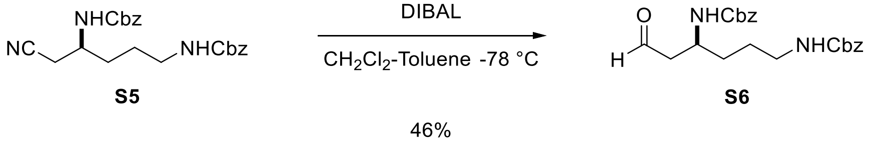

To a stirring solution of **S5** (1.00g, 2.53 mmol) in anhydrous CH_2Cl_2 (23 mL) was added diisobutylaluminum hydride (1M in toluene, 7.59 mmol) dropwise at -78 °C under a stream of argon over 15 mins. The reaction mixture was stirred at -78 °C for 3 h until consumption of starting material was observed through TLC analysis. The reaction was quenched by dropwise addition of cold MeOH (25 mL) before an aqueous solution of saturated potassium sodium tartrate (50 mL) was added. The mixture was left to stir for 12 h then was extracted with CH_2Cl_2 (3 x 25 mL). The organic layer was dried over sodium sulfate, filtered, and concentrated to a residue. The crude product was purified by silica gel chromatography with 3% MeOH/CH_2Cl_2 to yield 0.466 g (1.17 mmol, 46%) of **S6** as a colorless film. R_f (5% MeOH/CH_2Cl_2, CAM) = 0.32; $[\alpha]_D^{29}$ = -14.16 (c = 0.7, CH_2Cl_2); IR (thin film, cm^{-1}): 3325.45, 2930.21, 1690.90, 1525.55, 1454.14, 1247.89, 1073.72, 738.43, 697.23; ^1H NMR (500 MHz, $CDCl_3$) δ 9.73 (s, 1H), 7.36 – 7.31 (m, 10H), 5.08 (s, 2H), 5.07 (s, 2H), 4.87 (bs, 1H), 4.10 – 4.04 (m, 1H), 3.22 – 3.17 (m, 2H), 2.68 – 2.63 (m, 2H), 1.60 – 1.52 (m, 4H); ^{13}C NMR (125 MHz, $CDCl_3$) δ 201.0, 156.6, 156.0, 136.6, 136.4, 128.6, 128.6, 128.2, 128.2, 128.1, 128.1, 66.8, 66.6, 48.8, 46.7, 40.5, 31.9, 26.6. HRMS-ESI (m/z): $[M + Na]^+$ calcd for $C_{22}H_{26}N_2O_5Na$, 421.1739 Da; found 421.1736 Da.

Partially protected β-lysine **6**:

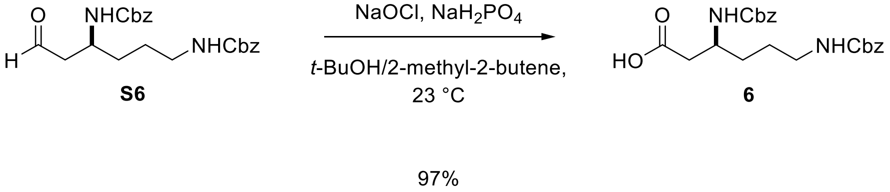

To a stirring solution of **S6** (0.710 g, 1.78 mmol) in *t*-BuOH (60 mL) was added 2-methyl-2-butene (3.78 mL, 35.64 mmol) at rt followed by solutions of NaOCl (1M in H_2O, 15.2 mmol) and NaH_2PO_4 (1M in H_2O, 15.2 mmol). The reaction mixture was stirred for 1 h at rt then quenched with a saturated solution of sodium sulfite (100 mL) and 1 M HCl until the solution was acidic (pH < 4). The aqueous layer was extracted with EtOAc (3 x 50 mL), washed with brine, then concentrated to a residue. The crude material was suspended in CH_2Cl_2 and the resulting precipitate was filtered off to afford 0.718 g (1.73 mmol, 97%) of **6** as a white solid: mp 146-149 °C (lit. mp 155 °C[S2]). $[\alpha]_D^{25}$ = +1.78 (*c* = 0.2, DMF) (lit $[\alpha]_D^{15}$ = +1.0 (*c* = 1.0, DMF)[S2]); IR (thin film, cm^{-1}): 3316.39, 2924.52, 1699.32, 1534.47, 1454.56, 1257.86, 1026.56, 738.50, 697.23; ^1H NMR (500 MHz, $(CD_3)_2SO$) δ 7.38 – 7.28 (m, 10H), 7.23 (t, *J* = 5.7 Hz, 1H), 7.19 (d, *J* = 8.7 Hz, 1H), 5.00 (s, 4H), 3.85 – 3.71 (m, 1H), 2.96 (q, *J* = 5.9 Hz, 2H), 2.34 (qd, *J* = 15.4, 6.9 Hz, 2H), 1.46 – 1.33 (m, 4H); ^{13}C NMR (125 MHz, $(CD_3)_2SO$) δ 172.5, 156.1, 155.6, 137.3, 137.3, 128.4, 127.8, 127.8, 127.7, 65.2, 65.1, 47.9, 40.3, 31.7, 31.4, 26.2. HRMS-ESI (*m / z*): $[M + Na]^+$ calcd for $C_{22}H_{26}N_2O_6Na$, 437.1689 Da; found 437.1683 Da.

D-Glucal **S7**:

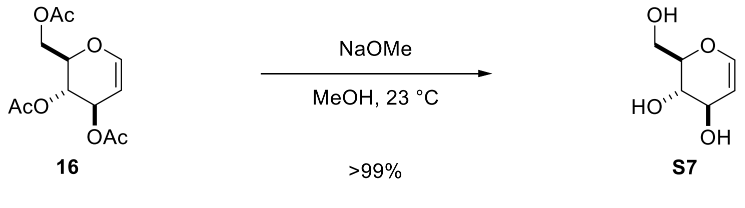

Sodium methoxide (0.304 g, 7.29 mmol) was added to a solution of **16** (25.0 g, 91.83 mmol) in MeOH (255 mL) and the reaction mixture was stirred at rt for 5 h. The solvent was concentrated to a residue to yield 13.40 g (13.40 g, >99%) of D-glucal **S7** as an amber oil and was carried on to the next step without further purification. R_f (9:1 CH_2Cl_2/MeOH, CAM) = 0.27; ^1H NMR (500

MHz, CD_3OD) δ 6.35 (dd, J = 6.1, 1.8 Hz, 1H), 4.68 (dd, J = 6.1, 2.2 Hz, 1H), 4.11 (dt, J = 7.1, 2.0 Hz, 1H), 3.88 (dd, J = 12.0, 2.5 Hz, 1H), 3.79 (dd, J = 12.0, 5.4 Hz, 1H), 3.72 (ddd, J = 9.8, 5.5, 2.5 Hz, 1H), 3.56 (dd, J = 9.7, 7.1 Hz, 1H); ^{13}C NMR (100 MHz, CD_3OD) δ 144.9, 104.5, 80.3, 70.9, 70.5, 62.2. The ^1H NMR and ^{13}C NMR data of **S7** are in accordance with those reported previously.[S3]

Benzyl glucal **9**:

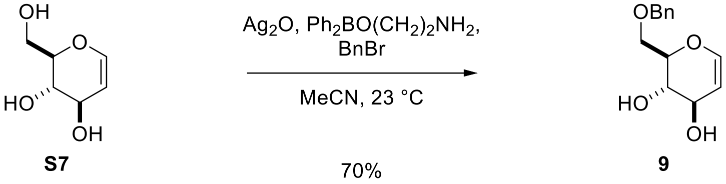

Silver oxide (32.06 g, 138.33 mmol) and 2-aminoethyl diphenylborinate (3.11 g, 13.93 mmol) were added successively to a stirred solution of **S7** (20.22 g, 138.33 mmol) diluted in acetonitrile (680 mL). Benzyl bromide (32.86 mL, 276.66 mmol) was added dropwise via addition funnel, and the reaction mixture was stirred at rt for 12 h. Following reaction completion, the reaction mixture was filtered on a celite pad, washed with EtOAc, and then concentrated to remove most of the acetonitrile. EtOAc (300 mL) was added and then brine (300 mL) was used to wash the organic layer. The organic layer was dried over sodium sulfate, filtered, and concentrated to a residue. The crude product was purified by silica gel chromatography with 1:1 hexanes:EtOAc to yield 22.97 g (97.22 mmol, 70%) of **9** as an off-white solid: mp 37-38 °C. R_f (1:1 EtOAc/hexanes, CAM) = 0.20; ^1H NMR (500 MHz, $CDCl_3$) δ 7.38 – 7.30 (m, 5H), 6.35 (dd, J = 6.1, 1.8 Hz, 1H), 4.74 (dd, J = 6.1, 2.3 Hz, 1H), 4.65 (d, J = 12.0 Hz, 1H), 4.57 (d, J = 12.0 Hz, 1H), 4.25 (t, J = 6.4 Hz, 1H), 3.92 (dt, J = 9.3, 4.1 Hz, 1H), 3.85 – 3.78 (m, 3H). ^{13}C NMR (100 MHz, $CDCl_3$) δ 144.5, 137.7, 128.7,

128.1, 128.0, 102.8, 76.6, 73.9, 71.6, 69.8, 69.5. The ^{1}H NMR and ^{13}C NMR data of **9** are in accordance with those reported previously.[S3]

Silyl glucal **S8**:

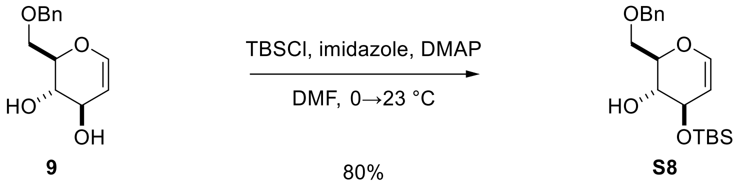

A solution of **9** (11.25 g, 47.62 mmol) in anhydrous DMF (112 mL) was cooled to 0 °C with an ice bath under a stream of argon. Imidazole (6.48 g, 95.23 mmol) and 4-dimethylaminopyridine (290 mg, 2.38 mmol) were added successively and allowed to stir for 5 mins. Next, *tert*-butyldimethylsilyl chloride (7.37 g, 47.62 mmol) was added and the reaction was stirred for 15 minutes before removing the ice bath. The reaction mixture was stirred for 12 h and reaction completion was determined through TLC analysis. The reaction mixture was diluted with diethyl ether (200 mL) and then quenched with brine (200 mL). The brine layer was extracted with diethyl ether (3 x 100 mL) and the combined organic extracts were washed with H_2O (2 x 300 mL) and brine (300 mL). The organic layer was dried over sodium sulfate, filtered, and concentrated to a residue. The crude product was purified by silica gel chromatography with 12:1 hexanes:EtOAc to yield 13.37 g (38.14 mmol, 80%) of **S8** as a clear oil. R_f (9:1 hexanes/EtOAc, CAM) = 0.31; ^{1}H NMR (500 MHz, $CDCl_3$) δ 7.38 – 7.27 (m, 5H), 6.30 (dd, J = 6.1, 1.6 Hz, 1H), 4.64 (dd, J = 6.1, 2.5 Hz 1H), 4.63 (d, J = 12.2 Hz, 1H), 4.58 (d, J = 12.2 Hz, 1H), 4.22 (dt, J = 6.5, 2.0 Hz, 1H), 3.99 (ddd, J = 8.8, 5.4, 3.3 Hz, 1H), 3.84 – 3.76 (m, 3H), 0.90 (s, 9H), 0.11 (s, 6H); ^{13}C NMR (100 MHz, $CDCl_3$) δ 143.3, 137.8, 128.4, 127.8, 127.7, 103.5, 77.1, 73.5, 70.4, 69.6, 69.0, 25.8, 18.1, -4.4, -4.5. The ^{1}H NMR and ^{13}C NMR data of **S8** are in accordance with those reported previously.[S3]

Mesyl glucal **17**:

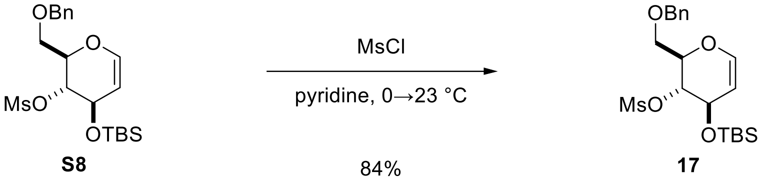

A solution of **S8** (20.37 g, 58.11 mmol) in anhydrous pyridine (118 mL) was cooled to 0 °C with an ice bath under a stream of argon. Mesyl chloride (9.00 mL, 116.22 mmol) was added dropwise to the reaction mixture, and a color change from light yellow to dark amber was observed following the remaining addition. The reaction was stirred for 15 mins before removing the ice bath, and stirring was continued for 12 h before reaction completion was determined through TLC analysis. The reaction mixture was diluted with diethyl ether (300 mL) and then quenched with H_2O (200 mL). The aqueous layer was extracted with diethyl ether (3 x 100 mL) and the combined organic extracts were washed with H_2O (2 x 300 mL) and 10% cupric sulfate pentahydrate (5 x 100 mL). The organic layer was dried over sodium sulfate, filtered, and concentrated to a residue. The crude product was purified by silica gel chromatography with 15:1 hexanes:EtOAc to yield 21.01 g (49.02 mmol, 84%) of **17** as a clear oil. R_f (9:1 hexanes/EtOAc, CAM) = 0.15; ^{1}H NMR (500 MHz, $CDCl_3$) δ 7.36 – 7.27 (m, 5H), 6.39 (dd, *J* = 6.2, 1.1 Hz, 1H), 4.79 – 4.75 (m, 2H), 4.60 (d, *J* = 11.9 Hz, 1H), 4.56 (d, *J* = 11.9 Hz, 1H), 4.40 – 4.33 (m, 1H), 4.31 – 4.26 (m, 1H), 3.82 (dd, *J* = 10.9, 7.1 Hz, 1H), 3.71 (dd, *J* = 10.9, 3.5 Hz, 1H), 3.06 (s, 3H), 0.87 (s, 9H), 0.10 (s, 6H); ^{13}C NMR (100 MHz, $CDCl_3$) δ 143.5, 137.7, 128.4, 127.9, 127.8, 101.5, 77.2, 75.3, 73.5, 67.8, 64.6, 38.8, 25.7, 17.9, -4.5, -4.6. The ^{1}H NMR and ^{13}C NMR data of **17** are in accordance with those reported previously.[S3]

Desilyl glucal **18**:

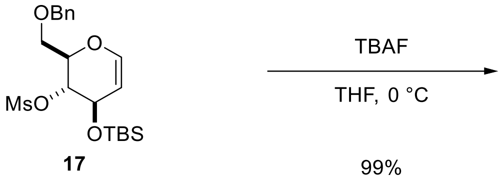

A solution of **17** (7.20 g, 16.79 mmol) in THF (80 mL) was cooled to 0 °C with an ice bath under a stream of argon. Tetra-n-butylammonium fluoride (1M in THF, 16.79 mmol) was added dropwise and the solution was stirred at 0 °C for 20 mins before reaction completion was determined through TLC analysis. The reaction mixture was poured into H_2O (100 mL) and extracted with diethyl ether (3 x 80 mL). The organic layer was dried over sodium sulfate, filtered, and concentrated to a residue. The crude product was purified by silica gel chromatography with 2:1 hexanes:EtOAc to yield 5.27 g (16.76 mmol, 99%) of **18** as a clear oil which was used in the next step immediately. R_f (1:1 hexanes/EtOAc, CAM) = 0.29; ^{1}H NMR (500 MHz, $CDCl_3$) δ 7.39 – 7.26 (m, 5H), 6.42 (dd, J = 6.0, 1.6 Hz, 1H), 4.88 – 4.78 (m, 2H), 4.60 (s, 2H), 4.47 (dt, J = 6.6, 2.3 Hz, 1H), 4.11 – 4.07 (dt, J = 9.3, 3.7 Hz, 1H), 3.84 – 3.78 (m, 2H), 3.11 (s, 3H); ^{13}C NMR (100 MHz, $CDCl_3$) δ 144.7, 137.5, 128.6, 128.1, 128.0, 102.2, 79.3, 75.2, 74.0, 68.3, 67.5, 38.6. The ^{1}H NMR and ^{13}C NMR data of **18** are in accordance with those reported previously.[S3]

Benzyl gulal **21**:

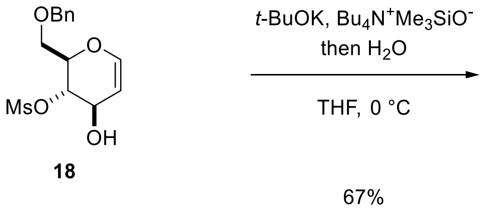

A solution of **18** (6.21 g, 19.75 mmol) in freshly distilled THF (95 mL) was added potassium *tert*-butoxide (2.28 g, 20.35 mmol) and stirred for 30 mins at rt. Meanwhile, to a solution of tetra-n-

butylammonium bromide (25.47 g, 79.02 mmol) in freshly distilled THF (200 mL) was added potassium trimethylsilanolate[S4] (10.56 g, 79.02 mmol) and stirred for 10 mins at rt. The reagent solution was filtered, concentrated to half its volume (100 mL), and added dropwise to the solution of 6-*O*-(benzyl)-4-*O*-mesyl D-glucal and potassium *tert*-butoxide over 30 mins at rt. The reaction mixture was stirred for 24 h before reaction completion was determined through TLC analysis. Diethyl ether (300 mL) was added, and the reaction mixture was poured into brine. The layers were separated, and the aqueous layer was extracted with diethyl ether (4 x 200 mL). The organic layer was dried over sodium sulfate, filtered, and concentrated to a residue. The crude product was purified by silica gel chromatography with 1:1 hexanes:EtOAc to yield 3.13 g (13.23 mmol, 67%) of **21** as an amber solid: mp 50-52 °C. R_f (3:7 hexanes/EtOAc, CAM) = 0.29; ^{1}H NMR (500 MHz, $CDCl_3$) δ 7.37 – 7.29 (m, 5H), 6.62 (d, *J* = 6.1 Hz, 1H), 4.99 (t, *J* = 6.3 Hz, 1H), 4.66 (d, *J* = 11.9 Hz, 1H), 4.60 (d, *J* = 12.0 Hz, 1H), 3.96 – 3.85 (m, 5H); ^{13}C NMR (125 MHz, $CDCl_3$) δ 147.0, 137.3, 128.6, 128.1, 127.9, 100.6, 74.0, 71.6, 71.1, 70.1, 63.9. The ^{1}H NMR and ^{13}C NMR data of **21** are in accordance with those reported previously.[S5]

Silyl glucal **S9**:

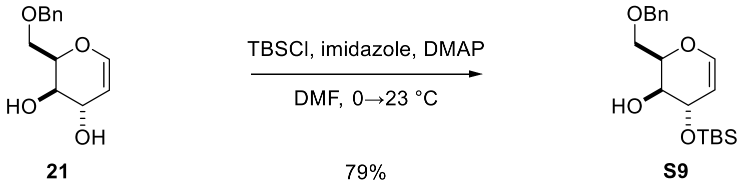

A solution of **21** (5.79 g, 24.51 mmol) in anhydrous DMF (65 mL) was cooled to 0 °C with an ice bath under a stream of argon. Imidazole (3.34 g, 49.03 mmol) and *tert*-butyldimethylsilyl chloride (4.43 g, 29.42 mmol) were added successively and the reaction was stirred for 15 minutes before removing the ice bath. The reaction mixture was stirred for 18 h and reaction completion was

determined through TLC analysis. The reaction mixture was diluted with diethyl ether (150 mL) and then quenched with brine (100mL). The brine layer was extracted with diethyl ether (3 x 100 mL) and the combined organic extracts were washed with H_2O (2 x 100 mL) and brine (50 mL). The organic layer was dried over sodium sulfate, filtered, and concentrated to a residue. The crude product was purified by silica gel chromatography with 4:1 hexanes:EtOAc to yield 6.79 g (19.37 mmol, 79%) of **S9** as a clear oil. R_f (1:1 hexanes/EtOAc, CAM) = 0.63; ^{1}H NMR (500 MHz, $CDCl_3$) δ 7.40 – 7.28 (m, 5H), 6.55 (d, J = 6.1 Hz, 1H), 4.89 – 4.83 (m, 1H), 4.66 (d, J = 12.0 Hz, 1H), 4.59 (d, J = 12.0 Hz, 1H), 4.03 – 3.98 (m, 1H), 3.93 – 3.87 (m, 2H), 3.85 (dd, J = 10.7, 4.6 Hz, 1H), 3.80 – 3.76 (m, 1H), 0.87 (s, 9H), 0.09 (s, 6H); ^{13}C NMR (125 MHz, $CDCl_3$) δ 145.8, 137.5, 128.6, 128.1, 127.9, 101.8, 74.0, 71.6, 71.6, 71.0, 64.6, 25.9, 18.2, -4.1, -4.5. The ^{1}H NMR and ^{13}C NMR data of **S9** are in accordance with those reported previously.[S5]

Carbomoylated gulal **22**:

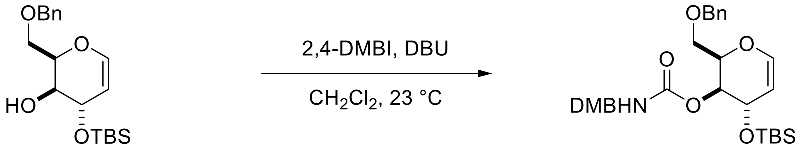

S9 96% **22**

To a stirring solution of **S9** (3.93 g, 11.21 mmol) in anhydrous CH_2Cl_2 (44 mL) was added a solution of freshly prepared 2,4-dimethoxybenzyl isocyanate[S6] (4.33 g, 22.43 mmol) in anhydrous CH_2Cl_2 (44 mL) and stirred for 1 h at rt under a stream of argon. LC-MS analysis was performed to determine reaction progress and upon completion, the reaction mixture was diluted with CH_2Cl_2 (50 mL) and quenched with a 10% solution of sodium bicarbonate (100 mL). The aqueous layer was extracted with CH_2Cl_2 (3 x 30 mL) and the combined organic extracts were washed with H_2O (200 mL). The organic layer was dried over sodium sulfate, filtered, and concentrated to a residue.

The crude product was purified by silica gel chromatography with 13:1 hexanes:EtOAc to yield 5.83 g (10.73 mmol, 96%) of **22** as a clear oil. R_f (5:1 hexanes/EtOAc, CAM) = 0.39; $[\alpha]_D^{28}$ = +66.11 (c = 0.8, CH_2Cl_2); IR (thin film, cm^{-1}): 2929.00, 2856.11, 1723.17, 1643.20, 1614.24, 1590.10, 1507.05, 1462.80, 1289.74, 1244.63, 1208.27, 1156.46, 1130.89, 1040.03, 937.34, 863.09, 836.22, 778.39, 739.13, 698.20; ^1H NMR (500 MHz, $CDCl_3$) δ 7.35 – 7.26 (m, 5H), 7.16 (d, J = 8.2 Hz, 1H), 6.51 (d, J = 6.1 Hz, 1H), 6.46 – 6.38 (m, 2H), 5.17 (t, J = 6.0 Hz, 1H), 4.83 (t, J = 5.4 Hz, 1H), 4.78 – 4.73 (m, 1H), 4.59 (d, J = 12.1 Hz, 1H), 4.52 (d, J = 12.2 Hz, 1H), 4.29 – 4.23 (m, 3H), 3.93 (dd, J = 5.4, 2.5 Hz, 1H), 3.80 (s, 3H), 3.79 (s, 3H), 3.67 (dd, J = 10.3, 7.6 Hz, 1H), 3.62 (dd, J = 10.5, 4.6 Hz, 1H), 0.89 (s, 9H), 0.13 (s, 3H), 0.10 (s, 3H); ^{13}C NMR (125 MHz, $CDCl_3$) δ 160.6, 158.5, 155.3, 145.4, 137.9, 130.2, 128.4, 127.7, 127.6, 118.9, 103.8, 101.7, 98.5, 73.4, 71.3, 70.4, 69.4, 62.0, 55.4, 55.3, 40.7, 25.8, 18.0, -4.4, -4.5. HRMS-ESI (m / z): $[M + Na]^+$ calcd for $C_{29}H_{41}NO_7SiNa$, 566.2550 Da; found 566.2545 Da.

Diol **23**:

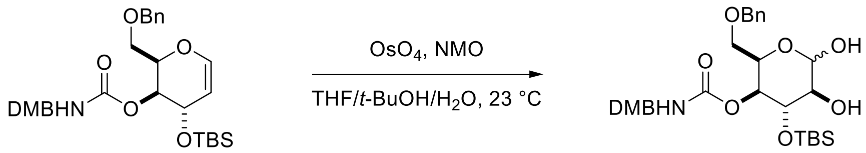

22 88% **23**

To a stirring solution of **22** (13.0 g, 23.91 mmol) in THF (70 mL), t-BuOH (40 mL) and H_2O (10 mL) was added N-methylmorpholine N-oxide (8.4 g, 71.73 mmol) and osmium tetraoxide (4% in H_2O, 15.2 mL) and continued to stir for 12 h. Consumption of starting material was determined through TLC analysis, and H_2O (200 mL) was used to dilute the reaction mixture. Sodium sulfite (15.1 g, 119.54 mmol) was added to the reaction mixture and stirred for an additional 2 h. CH_2Cl_2 (3 x 100 mL) was used to extract the aqueous layer and the combined organic layers were washed

with H_2O (150 mL), dried over sodium sulfate, filtered, and concentrated to a residue. The crude product was purified by silica gel chromatography with 4:1 hexanes:EtOAc to yield 12.12 g (21.01 mmol, 88%) of **23** as a clear, colorless oil. R_f (1:1 hexanes/EtOAc, CAM) = 0.46; $[\alpha]_D^{28}$ = +0.22 (*c* = 0.4, CH_2Cl_2); IR (thin film, cm^{-1}): 3367.52, 2929.15, 2857.12, 1725.89, 1614.46, 1590.21, 1508.14, 1463.29, 1253.54, 1208.47, 1156.50, 1095.17, 1035.67, 937.15, 838.52, 780.83, 736.80, 698.78; ^1H NMR (500 MHz, $CDCl_3$, 8:1 mixture of anomers, reporting for major) δ 7.31 – 7.27 (m, 5H), 7.14 (d, *J* = 8.2 Hz, 1H), 6.44 (d, *J* = 2.3 Hz, 1H), 6.39 (dd, *J* = 8.3, 2.4 Hz, 1H), 5.32 (t, *J* = 6.0 Hz, 1H), 4.99 (d, *J* = 11.3 Hz, 1H), 4.66 – 4.62 (m, 1H), 4.52 (d, *J* = 11.8 Hz, 1H), 4.41 (d, *J* = 11.9 Hz, 1H), 4.27 (dd, *J* = 6.1, 2.9 Hz, 2H), 4.22 (t, *J* = 6.0 Hz, 1H), 4.16 – 4.13 (m, 1H), 3.97 (d, *J* = 11.7 Hz, 1H), 3.80 (s, 3H), 3.78 (s, 3H), 3.59 (dd, *J* = 10.0, 6.6 Hz, 1H), 3.53 (dd, *J* = 10.0, 5.9 Hz, 1H), 3.42 (d, *J* = 8.9 Hz, 1H), 2.64 (d, *J* = 10.5 Hz, 1H), 0.88 (s, 9H), 0.16 (s, 3H), 0.12 (s, 3H); ^{13}C NMR (125 MHz, $CDCl_3$) δ 160.5, 158.4, 154.9, 137.8, 129.9, 128.3, 127.7, 127.7, 118.8, 103.9, 98.6, 93.0, 73.5, 72.0, 70.8, 69.9, 69.2, 69.1, 55.3, 55.3, 40.4, 25.6, 17.8, -4.9, -5.2. HRMS-ESI (*m / z*): $[M + Na]^+$ calcd for $C_{29}H_{43}NO_9SiNa$, 600.2605 Da; found 600.2602 Da.

Alloc-modified Burgess reagent **24**:

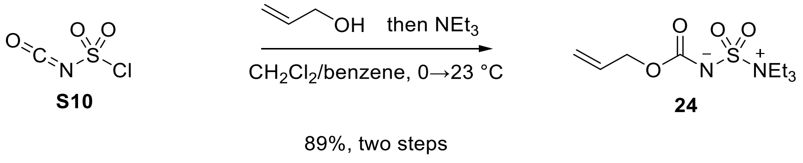

To a stirring solution of chlorosulfonylisocyanate **S10** (1.4 mL, 16.07 mmol) in anhydrous CH_2Cl_2 (4 mL) was added a solution of allyl alcohol (1.16 mL, 16.88 mmol) in anhydrous CH_2Cl_2 (4 mL) over 30 mins at 0 °C under a stream of argon. Upon complete addition, the reaction mixture was immediately concentrated to a residue and then diluted in anhydrous benzene (32 mL). This newly prepared solution was added dropwise to a solution of triethylamine (5.03 mL, 36.07 mmol) in

anhydrous benzene (20 mL) over 10 mins at rt under a stream of argon. The reaction mixture was stirred for 1 h at rt and then cooled to 4 °C for 20 mins before being filtered. The filtrate was concentrated to a residue to yield 3.98 g (14.3 mmol, 89% over 2 steps) of **24** as a clear oil that solidified upon standing. ^{1}H NMR (500 MHz, $CDCl_3$) δ 5.85 (ddt, *J* = 16.3, 10.8, 5.6 Hz, 1H), 5.23 (d, *J* = 17.3 Hz, 1H), 5.11 (d, *J* = 10.5 Hz, 1H), 4.47 (d, *J* = 5.8 Hz, 2H), 3.37 (q, *J* = 7.3 Hz, 6H), 1.31 (t, *J* = 7.3 Hz, 9H); ^{13}C NMR (100 MHz, $CDCl_3$) δ 157.4, 132.6, 118.0, 66.9, 50.5, 9.4. The ^{1}H NMR and ^{13}C NMR data of **24** are in accordance with those reported previously.[S7]

β-sulfamidate **8**:

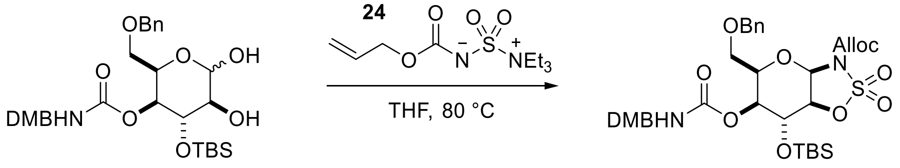

23 28% **8**

To a stirring solution of **23** (1.07 g, 1.86 mmol) in freshly distilled THF (19 mL) was added alloc-modified burgess reagent **25** (1.43 g, 4.64 mmol) which was immediately placed in an oil bath pre-warmed to 80 °C. The reaction mixture was refluxed for 2 h and consumption of starting material was determined through TLC analysis. Upon cooling to rt, the reaction mixture was diluted with CH_2Cl_2 (40 mL) and was added to a saturated ammonium chloride solution (100 mL). The aqueous layer was extracted with CH_2Cl_2 (3 x 40 mL) and the combined organic extracts were washed with H_2O (80 mL). The organic layer was dried over sodium sulfate, filtered, and concentrated to a residue. The crude product was purified by silica gel chromatography with 15:1 hexanes:EtOAc to yield 0.375 g (0.518 mmol, 28%) of **8** as a clear oil. R_f (3:1 hexanes/EtOAc, $KMnO_4$) = 0.36; $[\alpha]_D^{28}$ = -14.99 (*c* = 0.3, CH_2Cl_2); IR (thin film, cm^{-1}): 2928.66, 2856.86, 1754.50, 1722.04, 1614.36, 1590.41, 1508.24, 1455.92, 1388.06, 1321.81, 1288.54, 1258.51, 1207.50, 1102.98,

1036.88, 1000.58, 937.10, 837.22, 809.56, 784.38, 699.03, 571.37; ^{1}H NMR (500 MHz, $CDCl_3$) δ 7.33 – 7.26 (m, 5H), 7.15 (d, *J* = 8.2 Hz, 1H), 6.45 (d, *J* = 2.3 Hz, 1H), 6.43 – 6.36 (m, 2H), 5.93 (ddt, *J* = 16.3, 10.8, 5.4 Hz, 1H), 5.71 – 5.68 (m, 1H), 5.44 (d, *J* = 16.7 Hz, 1H), 5.35 (t, *J* = 5.8 Hz, 1H), 5.30 (d, *J* = 10.3 Hz, 1H), 4.83 (dd, *J* = 13.4, 5.4 Hz, 1H), 4.78 (dd, *J* = 13.4, 5.4 Hz, 1H), 4.61 – 4.58 (m, 1H), 4.54 (d, *J* = 12.0 Hz, 1H), 4.43 (d, *J* = 12.1 Hz, 1H), 4.40 – 4.38 (m, 2H), 4.28 (d, *J* = 6.0 Hz, 2H), 4.22 (t, *J* = 6.2 Hz, 1H), 3.81 (s, 3H), 3.79 (s, 3H), 3.62 – 3.55 (m, 2H), 0.90 (s, 9H), 0.21 (s, 3H), 0.17 (s, 3H); ^{13}C NMR (125 MHz, $CDCl_3$) δ 160.7, 158.7, 155.3, 148.8, 137.9, 130.4, 130.0, 128.5, 127.8, 127.7, 119.7, 118.7, 103.8, 98.6, 80.3, 77.3, 73.5, 70.2, 68.6, 67.3, 64.6, 55.5, 55.4, 40.9, 29.8, 25.6, 17.9, -4.8, -5.2. HRMS-ESI (*m / z*): $[M + Na]^+$ calcd for $C_{33}H_{46}N_2O_{12}SSiNa$, 745.2438 Da; found 745.2439 Da.

Azide **S11**:

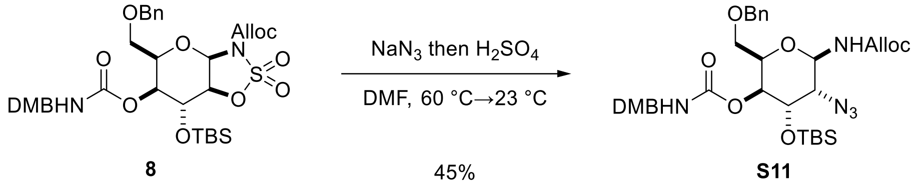

To a stirring solution of **8** (1.77 g, 2.44 mmol) in anhydrous DMF (24.4 mL) was added sodium azide (0.794 g, 12.22 mmol) at rt under a stream of argon. The reaction mixture was warmed to 60 °C and stirred for 3 h. LC-MS analysis was performed to determine reaction progress and upon completion, the reaction mixture was cooled to rt and quenched with 10% sulfuric acid (24 mL). The reaction mixture was stirred for an additional 30 mins and then poured into brine (50 mL). The brine layer was extracted with EtOAc (3 x 50 mL) and the combined organic extracts were washed with H_2O (2 x 100 mL) and brine (100 mL). The organic layer was dried over sodium sulfate, filtered, and concentrated to a residue. The crude product was purified by silica gel

chromatography with 0.5% MeOH/ CH_2Cl_2 to yield 0.757 g (1.10 mmol, 45%) of **S11** as a clear oil. R_f (0.5% MeOH/CH_2Cl_2, $KMnO_4$) = 0.20; $[\alpha]_D^{29}$ = -9.19 (c = 0.5, CH_2Cl_2); IR (thin film, cm^{-1}): 3334.46, 2930.09, 2857.73, 2106.69, 1728.42, 1614.49, 1589.84, 1508.46, 1463.25, 1255.31, 1209.04, 1131.78, 1099.57, 1037.30, 939.59, 835.55, 780.02, 737.55, 698.77; ^{1}H NMR (500 MHz, $(CD_3)_2CO$) δ 7.36 (d, J = 9.9 Hz, 1H), 7.32 – 7.25 (m, 5H), 7.15 (d, J = 8.3 Hz, 1H), 6.54 (d, J = 2.3 Hz, 1H), 6.53 – 6.49 (m, 1H), 6.42 (dd, J = 8.3, 2.3 Hz, 1H), 5.95 (ddt, J = 16.2, 10.5, 5.3 Hz, 1H), 5.35 – 5.29 (m, 2H), 5.18 (d, J = 10.5 Hz, 1H), 4.77 (d, J = 2.2 Hz, 1H), 4.58 (d, J = 5.4 Hz, 2H), 4.53 (d, J = 12.2 Hz, 1H), 4.48 (d, J = 12.1 Hz, 1H), 4.33 (t, J = 2.8 Hz, 1H), 4.29 (t, J = 6.7 Hz, 1H), 4.25 (d, J = 6.1 Hz, 2H), 3.83 (s, 3H), 3.77 (s, 3H), 3.62 (d, J = 8.9 Hz, 1H), 3.58 – 3.51 (m, 2H), 0.98 (s, 9H), 0.25 (s, 3H), 0.23 (s, 3H); ^{13}C NMR (100 MHz, $(CD_3)_2CO$) δ 161.3, 159.1, 156.4, 156.3, 139.4, 134.0, 129.9, 128.9, 128.1, 128.1, 120.0, 117.5, 104.8, 98.9, 78.5, 73.5, 72.5, 71.3, 70.7, 68.7, 65.9, 60.3, 55.7, 55.5, 40.3, 26.1, 18.5, -4.7, -5.0. HRMS-ESI (m / z): $[M + Na]^+$ calcd for $C_{33}H_{47}N_5O_9SiNa$, 708.3041 Da; found 708.3040 Da.

Gulosamine **7**:

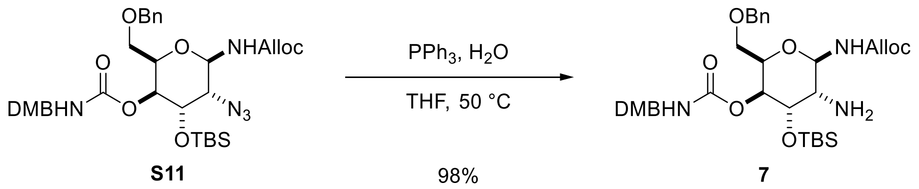

To a stirring solution of **S11** (0.864 g, 1.26 mmol) in THF (11.3 mL) and H_2O (1.1 mL) was added triphenylphosphine (0.991 g, 3.78 mmol) at rt. The reaction mixture was warmed to 50 °C and stirred for 12 h. LCMS analysis was performed to determine reaction progress and upon completion, the reaction mixture was cooled to rt and diluted with EtOAc (30 mL). The reaction mixture was added to H_2O (100 mL) and extracted with EtOAc (3 x 30 mL). The organic layer

was washed with brine (100 mL), dried over sodium sulfate, filtered, and concentrated to a residue. The crude product was purified by silica gel chromatography with 1:1 hexanes:EtOAc, 0.1% TEA to yield 0.812 g (1.23 mmol, 98%) of **7** as a clear oil. R_f (1:1 hexanes/EtOAc, 0.1% TEA, CAM) = 0.34; $[\alpha]_D^{29}$ = -42.10 (*c* =0.5, CH_2Cl_2); IR (thin film, cm^{-1}): 3310.39, 2928.59, 1726.67, 1615.08, 1536.41, 1507.72, 1463.10, 1364.15, 1233.74, 1208.36, 1135.05, 1074.18, 1036.27, 935.86, 836.06, 779.83, 739.16, 698.68; ^{1}H NMR (500 MHz, $CDCl_3$) δ 7.31 – 7.24 (m, 5H), 7.10 (d, *J* = 8.0 Hz, 1H), 6.43 – 6.38 (m, 2H), 6.27 (s, 1H), 5.87 (ddt, *J* = 16.3, 10.5, 5.5 Hz, 1H), 5.29 (d, *J* = 17.3 Hz, 1H), 5.19 (dd, *J* = 10.5, 1.6 Hz, 1H), 5.03 (t, *J* = 10.1 Hz, 1H), 4.84 – 4.80 (m, 1H), 4.61 – 4.53 (m, 2H), 4.51 (d, *J* = 11.8 Hz, 1H), 4.41 (d, *J* = 12.0 Hz, 1H), 4.34 (dd, *J* = 15.0, 6.0 Hz, 1H), 4.30 – 4.24 (m, 2H), 4.06 – 4.01 (m, 1H), 3.78 (s, 3H), 3.77 (s, 3H), 3.61 – 3.51 (m, 2H), 2.96 (d, *J* = 8.7 Hz, 1H), 0.90 (s, 9H), 0.18 (s, 3H), 0.00 (s, 3H); ^{13}C NMR (125 MHz, $CDCl_3$) δ 160.1, 158.0, 156.7, 156.0, 137.9, 132.5, 128.8, 128.4, 127.9, 127.7, 119.4, 117.7, 103.9, 98.7, 81.7, 73.6, 72.1, 70.7, 70.1, 68.4, 65.7, 55.4, 55.3, 50.7, 39.5, 25.8, 18.0, -4.6, -5.3. HRMS-ESI (*m / z*): [M + H]$^+$ calcd for $C_{33}H_{50}N_3O_9Si$, 660.3316 Da; found 660.3317 Da.

Protected β-lysyl-gulosamine **25**:

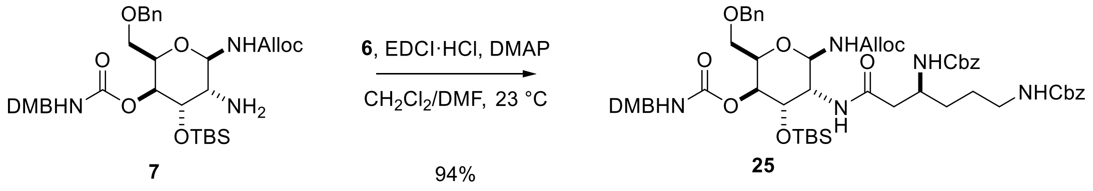

To a stirring solution of **7** (0.220 g, 0.333 mmol) in anhydrous CH_2Cl_2 (2 mL) and anhydrous DMF (1 mL) was added partially protected β-lysine **6** (0.152 g, 0.367 mmol), 4-dimethylaminopyridine (0.049 g, 0.40 mmol) and EDCI·HCl (0.128 g, 0.667 mmol) at rt under a stream of argon. The reaction mixture was stirred for 12 h and reaction completion was determined through TLC

analysis. The reaction mixture was diluted with EtOAc (20 mL) and then poured into brine (50mL). The brine layer was extracted with EtOAc (3 x 25 mL) and the combined organic extracts were washed with H_2O (2 x 30 mL) and brine (30 mL). The organic layer was dried over sodium sulfate, filtered, and concentrated to a residue. The crude product was purified by silica gel chromatography with 1:1 hexanes:EtOAc to yield 0.330 g (0.312 mmol, 94%) of **25** as a clear oil. R_f (1:1 hexanes/EtOAc, $KMnO_4$) = 0.24; $[\alpha]_D^{29}$ = -26.76 (c = 0.6, CH_2Cl_2); IR (thin film, cm^{-1}): 3324.27, 2930.02, 1719.94, 1614.75, 1509.23, 1455.22, 1253.78, 1209.49, 1101.76, 1040.73, 834.99, 779.19, 738.28, 697.93; ^{1}H NMR (500 MHz, $CDCl_3$) δ 7.36 – 7.27 (m, 12H), 7.26 – 7.23 (m, 3H), 7.14 (d, *J* = 8.2 Hz, 1H), 6.45 (d, *J* = 2.3 Hz, 1H), 6.38 (dd, *J* = 8.2, 2.4 Hz, 1H), 5.96 (d, *J* = 8.5 Hz, 1H), 5.91 – 5.87 (m, 1H), 5.87 – 5.79 (m, 1H), 5.40 (d, *J* = 8.6 Hz, 1H), 5.33 (t, *J* = 6.1 Hz, 1H), 5.22 (d, *J* = 17.1 Hz, 1H), 5.15 – 5.11 (m, 2H), 5.08 (s, 2H), 5.04 – 4.98 (m, 2H), 4.96 – 4.89 (m, 1H), 4.76 (d, *J* = 3.6 Hz, 1H), 4.56 – 4.44 (m, 3H), 4.40 (d, *J* = 12.2 Hz, 1H), 4.27 (dd, *J* = 6.0, 3.9 Hz, 2H), 4.21 (t, *J* = 6.4 Hz, 1H), 4.14 – 4.09 (m, 1H), 4.04 (t, *J* = 3.0 Hz, 1H), 3.96 – 3.89 (m, 1H), 3.81 (s, 3H), 3.78 (s, 3H), 3.54 (dd, *J* = 9.7, 6.0 Hz, 1H), 3.51 – 3.44 (m, 1H), 3.20 – 3.15 (m, 2H), 2.44 (d, *J* = 12.0 Hz, 1H), 2.36 (d, *J* = 11.5 Hz, 1H), 1.57 – 1.49 (m, 4H), 0.94 (s, 9H), 0.23 (s, 3H), 0.16 (s, 3H); ^{13}C NMR (100 MHz, $CDCl_3$) δ 171.47, 160.8, 158.78, 156.5, 156.0, 155.9, 155.2, 138.1, 136.7, 136.6, 132.5, 130.3, 128.6, 128.3, 128.2, 128.1, 127.6, 127.6, 118.7, 117.9, 103.9, 98.6, 79.9, 73.3, 71.4, 69.8, 69.7, 67.7, 66.8, 66.6, 65.9, 55.4, 55.4, 48.8, 48.3, 41.0, 40.8, 40.6, 31.4, 26.7, 25.9, 18.0, -4.4, -4.9. HRMS-ESI (m / z): $[M + Na]^+$ calcd for $C_{55}H_{73}N_5O_{14}SiNa$, 1078.4821 Da; found 1078.4835 Da.

β-lysyl-gulosamine **25**:

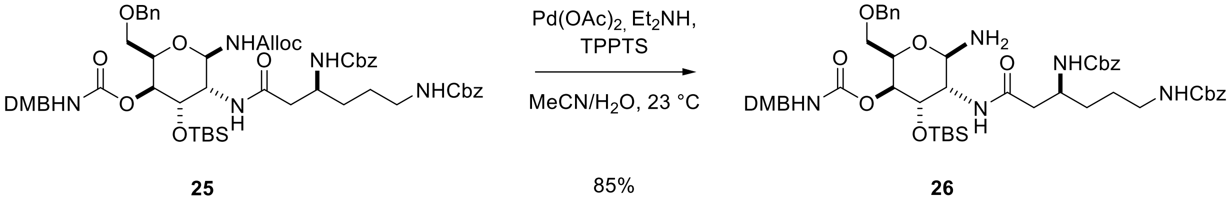

To a stirring solution of **25** (0.410 g, 0.388 mmol) diluted in MeCN (3.5 mL) and H_2O (3.5 mL) was added diethylamine (1.61 mL, 15.53 mmol) at rt. The reaction mixture was stirred for 10 mins, and then triphenylphosphine-3,3′,3″-trisulfonic acid trisodium salt (0.044 g, 0.078 mmol) was added at rt and stirred for 1 h. Consumption of starting material was determined through TLC analysis, the reaction mixture was diluted with EtOAc (20 mL) and poured into H_2O (50 mL). The aqueous layer was extracted with EtOAc (3 x 25 mL) and the combined organic extracts were washed with brine (100 mL). The organic layer was dried over sodium sulfate, filtered, and concentrated to a residue. The crude product was purified by silica gel chromatography with 3% iPrOH/CH_2Cl_2 to yield 0.321 g (0.330 mmol, 85%) of **26** as a colorless gum. R_f (3% iPrOH/CH_2Cl_2, CAM) = 0.15; $[\alpha]_D^{29}$ = -25.07 (c = 0.6, CH_2Cl_2); IR (thin film, cm^{-1}): 3304.97, 2928.37, 1700.19, 1508.82, 1454.60, 1253.08, 1209.23, 1114.19, 1028.68, 833.75, 778.42, 737.72, 697.75; ^{1}H NMR (500 MHz, CD_3OD) δ 7.36 – 7.23 (m, 15H), 7.12 (d, J = 8.3 Hz, 1H), 6.49 (d, J = 2.3 Hz, 1H), 6.40 (dd, J = 8.3, 2.4 Hz, 1H), 5.05 (s, 4H), 4.73 (d, J = 2.5 Hz, 1H), 4.49 (d, J = 11.8 Hz, 1H), 4.44 (d, J = 11.9 Hz, 1H), 4.33 (d, J = 9.7 Hz, 1H), 4.20 (s, 2H), 4.16 (t, J = 6.6 Hz, 1H), 4.08 (t, J = 3.4 Hz, 1H), 3.99 – 3.95 (m, 1H), 3.87 (d, J = 9.7 Hz, 1H), 3.79 (s, 3H), 3.74 (s, 3H), 3.57 – 3.49 (m, 2H), 3.14 – 3.08 (m, 2H), 2.43 (dd, J = 14.2, 6.1 Hz, 1H), 2.37 (dd, J = 14.3, 6.8 Hz, 1H), 1.62 – 1.54 (m, 2H), 1.52 – 1.44 (m, 2H), 0.94 (s, 9H), 0.16 (s, 3H), 0.11 (s, 3H); ^{13}C NMR (100 MHz, $CDCl_3$) δ 170.6, 160.7, 158.7, 156.5, 156.2, 155.2, 138.0, 136.8, 136.7, 130.3, 128.6, 128.5, 128.4, 128.1, 128.1, 128.0, 128.0, 127.7, 118.8, 103.9, 98.6, 82.9, 73.5, 71.6, 70.6,

69.9, 69.2, 66.6, 55.5, 55.4, 50.7, 48.5, 41.3, 41.0, 40.7, 31.4, 29.8, 26.8, 25.8, 18.0, -4.4, -5.0. HRMS-ESI (*m / z*): $[M + H]^+$ calcd for $C_{51}H_{70}N_5O_{12}Si$, 972.4790 Da; found 972.4805 Da.

Thiourea **2**:

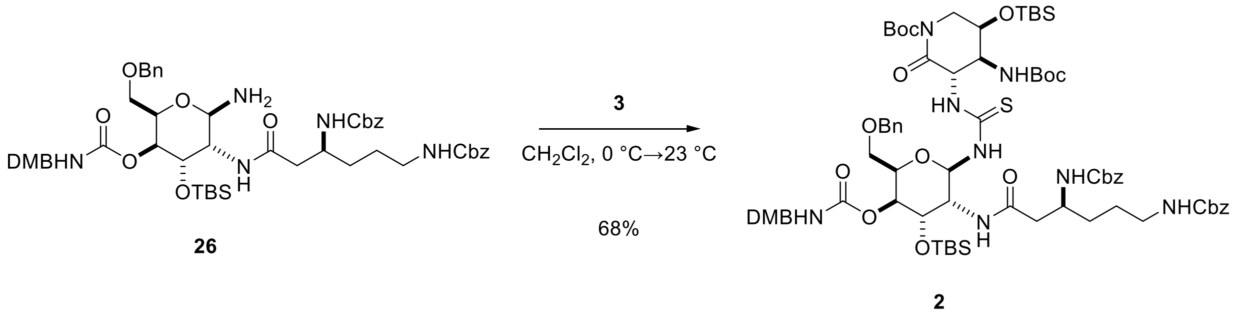

To a stirring solution of **26** (0.104 g, 0.209 mmol) in anhydrous CH_2Cl_2 (0.420 mL) was added a solution of **3** (0.190 g, 0.195 mmol) in anhydrous CH_2Cl_2 (1.5 mL) at 0 °C under a stream of argon. The reaction mixture was stirred for 15 mins, warmed to rt, fitted with a glass stopper, and stirred for 12 h. The reaction mixture was concentrated to a residue and the crude product was purified by silica gel chromatography with 1% MeOH/$CHCl_3$ to yield 0.197 g (0.062 mmol, 68%, 76% brsm) of **2** as an off-white solid: mp 60-63 °C. R_f (3% MeOH/$CHCl_3$, $KMnO_4$) = 0.21; $[\alpha]_D^{29}$ = -17.93 (*c* = 0.2, CH_2Cl_2); IR (thin film, cm^{-1}): 3333.63, 2927.44, 2855.59, 1717.94, 1534.49, 1456.32, 1368.54, 1256.13, 1132.01, 1038.53, 835.55, 779.68, 738.06, 698.07. Two-dimensional nuclear magnetic resonance spectra were recorded at ambient temperature on a 600 MHz Bruker NMR spectrometer. ^1H NMR (500 MHz, $CDCl_3$) δ 7.76 – 7.71 (m, 1H), 7.57 – 7.54 (m, 1H), 7.43 – 7.40 (m, 1H), 7.36 – 7.28 (m, 16H), 7.23 (d, *J* = 6.7 Hz, 1H), 7.16 (d, *J* = 8.1 Hz, 1H), 6.45 (s, 1H), 6.40 (d, *J* = 9.5 Hz, 1H), 6.11 – 6.00 (m, 1H), 5.17 – 5.11 (m, 2H), 5.10 – 5.04 (m, 2H), 4.81 – 4.77 (m, 1H), 4.67 (d, *J* = 12.1 Hz, 1H), 4.46 – 4.36 (m, 2H), 4.33 – 4.25 (m, 5H), 4.13 – 4.09 (m, 3H), 3.99 – 3.89 (m, 1H), 3.81 (s, 3H), 3.79 (s, 3H), 3.77 – 3.76 (m, 1H), 3.60 – 3.57 (m, 1H), 3.51 – 3.41 (m, 1H), 3.26 – 3.11 (m, 2H), 2.60 – 2.47 (m, 1H),

2.34 – 2.24 (m, 1H), 1.74 – 1.72 (m, 2H), 1.64 – 1.62 (m, 2H), 1.34 (s, 9H), 1.27 (s, 9H), 0.96 (s, 9H), 0.92 (s, 9H), 0.23 (s, 3H), 0.16 (s, 3H), 0.14 (s, 3H), 0.08 (s, 3H); ^{13}C NMR (100 MHz, $CDCl_3$) δ 187.2, 171.7, 167.8, 160.7, 158.6, 156.8, 156.6, 155.6, 155.3, 151.8, 138.4, 136.9, 136.8, 132.5, 131.0, 130.3, 128.9, 128.6, 128.5, 128.3, 128.2, 128.1, 127.9, 127.7, 127.5, 119.0, 103.9, 98.6, 83.5, 79.7, 77.4, 73.4, 71.9, 69.8, 68.1, 66.8, 66.5, 66.0, 55.5, 55.4, 50.5, 49.6, 40.8, 32.0, 29.8, 29.5, 28.5, 27.9, 27.8, 26.5, 25.9, 25.8, 22.8, 19.2, 18.1, 18.1, 14.2, -4.5, -4.8, -4.9. HRMS-ESI (*m / z*): $[M + Na]^+$ calcd for $C_{73}H_{108}N_8O_{18}SSi_2Na$, 1495.6939 Da; found 1495.6983 Da. ^{1}H assignments are included along the f2 projection for the ^{1}H-^{1}H COSY, the ^{1}H-^{13}C HSQC, and the ^{1}H-^{13}C HMBC spectra. ^{13}C assignments are not included to prevent the assignment of ambiguous signals.

Protected guanidine **28**:

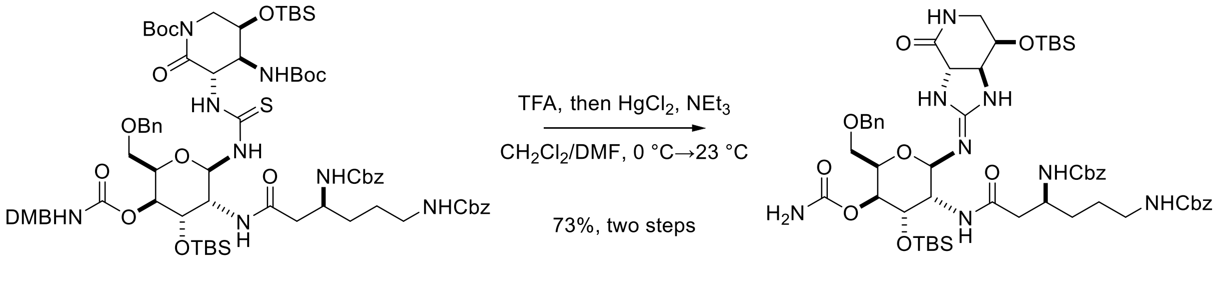

2 **28**

To a stirring solution of **2** (0.074 g, 0.050 mmol) in anhydrous CH_2Cl_2 (5 mL) was added TFA (2.5 mL) at 0 °C under a stream of argon. The reaction mixture was stirred for 4 h at 0 °C, and consumption of starting material was observed through TLC analysis. While maintaining a temperature of 0 °C, the reaction mixture was quenched through the addition of a saturated solution of sodium bicarbonate (20 mL). The reaction mixture was warmed to rt, diluted with EtOAc (20 mL) and extracted with EtOAc (3 x 20 mL). The organic extracts were washed with

brine (100 mL), dried over sodium sulfate, filtered, and concentrated to a residue. The crude amine (0.064 g) was used directly in the next step. To a stirring solution of crude amine (0.064 g) in anhydrous DMF (1.2 mL) was added triethylamine (0.024 mL, 0.171 mmol) and mercury (II) chloride (0.046 g, 0.171 mmol) successively at 0 °C under a stream of argon. The reaction mixture was stirred for 15 mins, warmed to rt and stirred for 3 h when consumption of starting material was observed through TLC analysis. The reaction mixture was diluted with EtOAc (10 mL), filtered through celite, and then poured into brine (25 mL). The brine layer was extracted with EtOAc (3 x 10 mL) and the combined organic extracts were washed with H_2O (3 x 50 mL) and brine (50 mL). The organic layer was dried over sodium sulfate, filtered, and concentrated to a residue. The crude product was purified by silica gel chromatography with 6% MeOH/CH_2Cl_2 to yield 0.045 g (0.041 mmol, 73%, 2 steps) of **28** as a white film. R_f (12% MeOH/CH_2Cl_2, $KMnO_4$) = 0.49; $[\alpha]_D^{29}$ = -4.56 (c = 0.8, MeOH); IR (thin film, cm^{-1}): 3295.76, 2925.21, 2854.12, 2030.25, 1983.45, 1965.40, 1700.21, 1538.76, 1462.74, 1255.35, 1074.39, 836.14, 780.42, 697.90, 472.31; ^{1}H NMR (500 MHz, CD_3OD) δ 7.37 – 7.29 (m, 15H), 5.13 (d, J = 12.5 Hz, 1H), 5.07 (d, J = 8.9 Hz, 3H), 5.01 (d, J = 9.0 Hz, 1H), 4.73 – 4.68 (m, 1H), 4.68 – 4.61 (m, 1H), 4.58 – 4.54 (m, 3H), 4.42 – 4.39 (m, 1H), 4.19 (t, J = 3.3 Hz, 1H), 4.11 (d, J = 9.2 Hz, 1H), 3.99 (t, J = 8.1 Hz, 1H), 3.91 (d, J = 14.5 Hz, 1H), 3.71 – 3.64 (m, 2H), 3.61 (dd, J = 13.9, 4.7 Hz, 1H), 3.23 (d, J = 13.9 Hz, 1H), 3.15 – 3.09 (m, 2H), 2.41 (dd, J = 13.4, 4.3 Hz, 1H), 2.18 (dd, J = 13.4, 9.7 Hz, 1H), 1.60 – 1.54 (m, 2H), 1.52 – 1.45 (m, 2H), 0.97 (s, 9H), 0.92 (s, 9H), 0.18 (s, 3H), 0.15 (s, 3H), 0.14 (s, 3H), 0.11 (s, 3H); ^{13}C NMR (100 MHz, $CDCl_3$) δ 172.0, 167.8, 163.2, 157.0, 156.9, 155.9, 136.9, 136.8, 136.6, 128.6, 128.5, 128.4, 128.2, 128.0, 127.9, 127.8, 127.6, 127.5, 127.3, 78.7, 73.8, 72.1, 70.2, 69.3, 68.6, 66.5, 66.3, 62.9, 61.4, 54.5, 50.9, 50.8, 50.7, 41.9, 40.4, 31.8, 26.2, 25.8, 25.7, 18.1, 17.9, -4.5, -4.7, -4.9, -5.2. HRMS-ESI (m / z): $[M + H]^+$ calcd for $C_{54}H_{81}N_8O_{12}Si_2$, 1089.5512

Da; found 1089.5528 Da. ^{1}H assignments are included along the f2 projection for the ^{1}H-^{1}H COSY and the ^{1}H-^{13}C HSQC. ^{13}C assignments are not included to prevent the assignment of ambiguous signals.

Guanidine **29**:

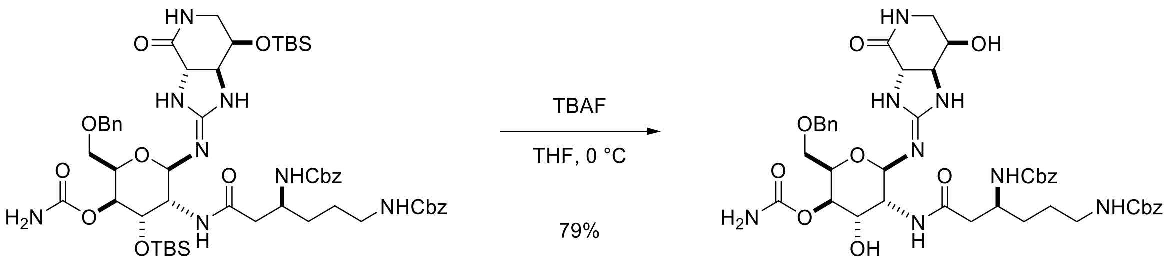

28 **29**

To a stirring solution of **28** (0.051 g, 0.047 mmol) in freshly distilled THF (3.5 mL) was added tetra-n-butylammonium fluoride (1M in THF, 0.500 mL) dropwise and the solution was stirred at 0 °C for 20 mins before reaction completion was determined through TLC analysis. The reaction mixture was poured into brine (10 mL) and extracted with EtOAc (3 x 10 mL). The organic layer was dried over sodium sulfate, filtered, and concentrated to a residue. The crude product was purified by silica gel chromatography with 12% MeOH/CH_2Cl_2 to yield 0.032 g (0.037 mmol, 79%) of **29** as a white film. R_f (12% MeOH/CH_2Cl_2, CAM) = 0.33; $[\alpha]_D^{29}$ = -6.20 (*c* = 0.3, MeOH); IR (thin film, cm^{-1}): 2922.25, 2852.48, 1659.57, 1463.04. ^{1}H NMR (500 MHz, CD_3OD) δ 7.36 – 7.32 (m, 12H), 7.30 – 7.26 (m, 3H), 5.12 (d, *J* = 12.5 Hz, 1H), 5.07 – 5.02 (m, 4H), 4.78 (d, *J* = 2.1 Hz, 1H), 4.57 (d, *J* = 14.4 Hz, 1H), 4.54 (s, 2H), 4.51 – 4.46 (m, 1H), 4.42 (t, *J* = 6.2 Hz, 1H), 4.16 (dd, *J* = 9.7, 2.9 Hz, 1H), 4.00 (t, *J* = 3.3 Hz, 1H), 3.97 – 3.93 (m, 1H), 3.86 (dd, *J* = 14.6, 2.5 Hz, 1H), 3.66 (d, *J* = 6.2 Hz, 2H), 3.64 – 3.59 (m, 1H), 3.30 – 3.26 (m, 1H), 3.10 (t, *J* = 5.9 Hz, 2H), 2.47 – 2.40 (m, 1H), 2.30 (dd, *J* = 13.8, 8.8 Hz, 1H), 1.57 – 1.47 (m, 4H); ^{13}C NMR (150 MHz, CD_3OD) δ 137.06, 136.94, 129.45, 128.16, 128.09, 127.92, 127.78, 127.67, 127.57, 127.37,

127.32, 127.28, 125.57, 76.87, 73.39, 73.25, 72.87, 69.52, 66.16, 65.99, 65.61, 59.58, 58.14, 58.11, 54.87, 48.18, 48.04, 47.90, 47.76, 47.61, 47.47, 47.33, 47.19, 31.67, 29.54, 29.43, 29.39, 29.35, 29.25, 29.23, 29.20, 29.07, 29.05, 28.94, 28.93, 26.71, 25.53, 23.56, 23.42, 22.63, 22.34, 19.33, 13.05, 12.56. HRMS-ESI (*m / z*): $[M + H]^+$ calcd for $C_{42}H_{53}N_8O_{12}$, 861.3783 Da; found 861.3785 Da. ^1H assignments are included along the f2 projection for the ^1H-^1H COSY and the ^1H-^{13}C HSQC. ^{13}C assignments are not included to prevent the assignment of ambiguous signals.

Streptothricin F sulfate **1**:

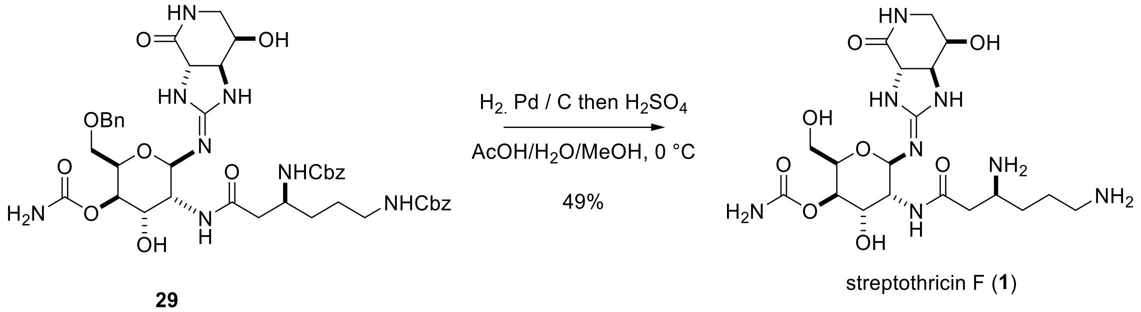

29 streptothricin F (**1**)

To a stirring solution of **29** (0.020 g, 0.023 mmol) in AcOH (0.4 mL), H_2O (0.4 mL) and MeOH (0.2 mL) was added palladium (10% on carbon, 5 mg). The reaction vessel was evacuated and purged with argon gas (7x), evacuated, and purged with hydrogen gas (7x) and then stirred overnight under a hydrogen atmosphere (balloon). The reaction was filtered through a 0.45 µm PVDF filter, the filter was washed with H_2O (2 x 1 mL) and concentrated to a residue. The crude product was diluted in H_2O (0.5 mL) and purified by Sephadex (LH-20) size exclusion gel with a mobile phase of 90% H_2O and 10% methanol at a rate of 0.47 mL / min. Fractions testing positive for the ninhydrin stain were analyzed by LC-MS. Pure fractions were collected and freeze dried overnight, isolating the acetate salt. To a stirring solution of ST-F acetate in H_2O (0.5 mL) was added 1 M H_2SO_4 dropwise until pH = 2. The resulting solution was added dropwise to vigorously stirring MeOH (20 mL) and Et_2O (10 mL). The slurry was stirred for 20 mins and then solids were

collected by centrifugation, washed with 1:1 MeOH:Et_2O (10 mL) and Et_2O (10 mL). The resulting precipitate was collected by centrifugation to yield 5.71 mg of S-F sulfate **1** (0.00823 mmol, 49%) as a white solid: mp >210 °C. $[\alpha]_D^{21}$ = -43.45 (c = 0.1, H_2O). Additional ^{1}H NMR, ^{13}C NMR, and two-dimensional nuclear magnetic resonance spectra were recorded at ambient temperature, on a 600 MHz Bruker NMR spectrometer (operating at 150 MHz for ^{13}C NMR). ^{1}H NMR (500 MHz, D_2O) δ 5.09 (d, J = 9.8 Hz, 1H), 4.75 (d, J = 3.4 Hz, 1H), 4.73 – 4.69 (m, 1H), 4.61 (d, J = 14.7 Hz, 1H), 4.32 (t, J = 5.9 Hz, 1H), 4.24 (dd, J = 9.8, 3.0 Hz, 1H), 4.15 (t, J = 3.5 Hz, 1H), 4.06 (d, J = 13.8 Hz, 1H), 3.79 (dd, J = 14.6, 5.7 Hz, 1H), 3.71 (d, J = 5.7 Hz, 2H), 3.69 – 3.66 (m, 1H), 3.38 (d, J = 14.6 Hz, 1H), 3.05 – 3.01 (m, 2H), 2.79 (dd, J = 16.7, 4.4 Hz, 1H), 2.67 (dd, J = 16.7, 8.3 Hz, 1H), 1.82 – 1.74 (m, 4H). HRMS-ESI (m / z): $[M + H]^+$ calcd for $C_{19}H_{35}N_8O_8$, 503.2577 Da; found 503.2574 Da. The ^{1}H NMR data of S-F are in accordance with those reported previously and the isolated samples of streptothricin F please see Table S3 for an in-depth analysis.[S8]

Streptothricin isolation:

Purification to separate streptothricin compounds of the nourseothricin sulfate mixture was performed through modification of a previously reported method.[S9] A glass column (150 cm x 2.4 cm) was packed with Sephadex LH-20 size exclusion gel using a mobile phase of 10% methanol / H_2O. The flow rate was adjusted using compressed air to 0.6 mL / min. Purifications were run in batches of approximately 300 mg of nourseothricin sulfate. Nourseothricin sulfate was diluted in 0.6 mL of H_2O and loaded dropwise directly onto the top of the column. A mobile phase of 10% methanol / H_2O was used for elution and fraction sizes of 3 mL were collected. Fractions testing positive for the ninhydrin stain were analyzed for purity by LC-MS. Pure streptothricin D began eluting after approximately 120 mL of mobile phase, followed by mixed fractions of streptothricin D / E / F, and finally pure streptothricin F. Pure fractions for streptothricin D were combined, frozen, and lyophilized to give a powdery, off-white solid. Pure fractions for streptothricin F were combined, frozen, and lyophilized to give a powdery, off-white solid.

Streptothricin F elemental analysis results:

Sample: DM1 ST-F

C	H	N	O
31.41 %	5.49 %	15.00 %	35.78 %

S
7.06 %

Figure S4: Elemental analysis of isolated streptothricin F from commercially sourced nourseothricin sulfate. The percent compositions are consistent with a molecular formula of $C_{19}H_{34}N_8O_8$ • 3/2 H_2SO_4 • 3 H_2O. These results align with the previously described elemental analysis and molecular formula of streptothricin F from Taniyama et al.[S9]

Streptothricin F analytical data comparison: previously reported, synthetic, isolated:

Table S3. ^{1}H NMR comparison of Streptothricin F.[S8]

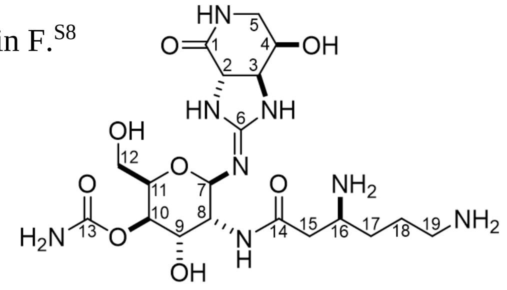

Streptothricin F (**1**)

Position	Ji, Z. et al. *J. Antibiot. (Tokyo).* 2007, 60 (12), 739–744.			Synthetic S-F, 500 MHz			Isolated S-F, 500 MHz			Isolated S-F, 600 MHz		
	δ H (ppm)	mult	J (Hz)	δ H (ppm)	mult	J (Hz)	δ H (ppm)	mult	J (Hz)	δ H (ppm)	mult	J (Hz)
H-2	4.64	d	14	4.61	d	13.7	4.61	d	14.4	4.63	d	13
H-3	4.1	d	14	4.06	d	13.8	4.06	d	15	4.08	d	14.3
H-4	4.74	m	-	4.73	m	-	4.71	m	-	4.73	m	-
H-5_a	3.83	dd	6, 15	3.79	dd	5.7, 14.7	3.79	dd	5.7, 14.7	3.81	dd	5.7, 14.7
H-5_b	3.42	d	15	3.38	d	14.6	3.38	d	14.6	3.39	d	14.6
H-7	5.11	d	10	5.09	d	9.8	5.1	d	9.8	5.11	d	9.8
H-8	4.28	dd	3, 10	4.24	dd	2.6, 9.8	4.23	dd	2.8, 9.9	4.24	dd	2.8, 9.8
H-9	4.18	t	3	4.15	t	3	4.15	t	3.2	4.17	t	3.3
H-10	4.79	m	-	4.75	d	3.4	4.75	m	-	4.76	m	-
H-11	4.35	t	6	4.32	t	6	4.31	t	6	4.33	t	6.1
H-12_a	3.75	d	6	3.71	d	5.7	3.71	d	5.7	3.75-3.64	m	-
H-12_b	3.75	d	6	3.71	d	5.7	3.71	d	5.7	3.75-3.64	m	-
H-15_a	2.82	dd	4, 16	2.79	dd	4.2, 16.5	2.79	dd	4.3, 16.7	2.81	dd	4.3, 16.7
H-15_b	2.7	dd	4, 16	2.67	dd	8.1, 16.3	2.68	dd	8.3, 16.5	2.70	dd	8.3, 16.6
H-16	3.71	m	-	3.69	m	-	3.68	m	-	3.75-3.64	m	-
H-17	1.81	m	-	1.82-1.73	m	-	1.79	m	-	1.80	m	-
H-18	1.81	m	-	1.82-1.73	m	-	1.79	m	-	1.80	m	-
H-19	3.07	m	-	3.05	m	-	3.04	m	-	3.04	t	6.8

Streptothricin F analytical data comparison: previously reported, synthetic, isolated:

Table S4. ^{13}C NMR comparison of Streptothricin F.[S8]

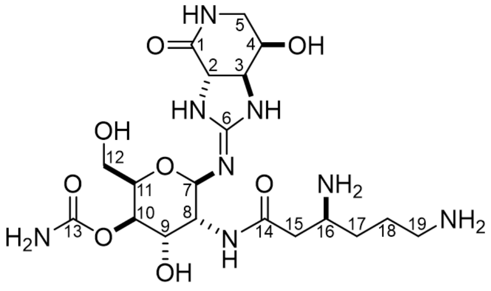

Streptothricin F (**1**)

Position	Ji, Z. et al. *J. Antibiot. (Tokyo).* 2007, 60 (12), 739–744. δ (ppm)	Isolated S-F, 600 MHz δ (ppm)
1	172.5	172.5
2	56.9	56.8
3	63.4	63.3
4	63.4	62.7
5	51.8	51.6
6	165.3	165.1
7	81.3	81.1
8	51.4	51.5
9	69	68.9
10	72.6	72.4
11	76.1	76.0
12	62.9	62.6
13	160.4	160.3
14	174.6	174.5
15	38.8	38.7
16	50.8	50.7
17	31.6	31.43
18	25.5	25.3
19	41.5	41.4

Streptothricin F analytical data comparison: previously reported, synthetic, isolated:

Table S. Optical rotation comparison of Streptothricin F.[S10–S12]

Source	$[\alpha]_D^{°C}$
Synthetic<br>Streptothricin F Sulfate<br>(Manetsch Lab)	$[\alpha]_D^{21}$: -43.45
Isolated<br>Streptothricin F Sulfate<br>(Manetsch Lab)	$[\alpha]_D^{24}$: -41.06
Streptothricin F Hydrochloride<br>Peck, R. et al.<br>*J. Am. Chem. Soc.*<br>1946, 68 (5), 772 –776.	$[\alpha]_D^{25}$: -51.3
Streptothricin F Hydrochloride<br>Kawamura, T. et al.<br>*J. Antibiot. (Tokyo).*<br>1976, *29* (8), 844–846.	$[\alpha]_D^{20}$: -46.1
Streptothricin F Hydrochloride<br>Kusumoto, S. et al.<br>*J. Antibiot. (Tokyo).*<br>1982, *35* (7), 925–927.	$[\alpha]_D^{20}$: -46.7

Antibacterial Procedures.

Minimal inhibitory concentration analysis was performed using the broth microdilution reference method according to Clinical Laboratory and Standards Institutes (CLSI)[S13] with the exception that antibiotics were added to microwell plates using digital dispensing technology as described by our group.[S14–S16] We previously demonstrated that this inkjet printing dispensing method for addition of antimicrobials to microplates was as accurate and more precise that manual reference methods of preparing doubling dilution MIC panels. *B. anthracis* Sterne 9131 was previously described.[S12-15] The methodology has since been adopted the United States Centers for Disease Center and Prevention at its Antibiotic Resistance Laboratory Network based on our reports.[S18] Per standard reference procedure, doubling dilutions of antibiotic were added to microplates using inkjet printing technology, followed by bacteria at ~10^5 colony forming units per mL based on dilutions from a 0.5 McFarland standard in cation-adjusted Mueller Hinton broth.[S13] After overnight growth at 35°C for 16-20 hours, minimal inhibitory concentrations were recorded as the concentration that inhibited growth based on an A_{600} of 0.08, which corresponds to the reference standard, inhibition of visual growth for MIC determinations, on a TECAN M1000 plate reader, as previously described.[S13,S14] *B. cenocepacia* K56-2 was provided by John Lipuma (University of Michigan). Other strains and isolates were from the American Type Culture Collection (ATCC, Manassas, VA), BEI Resources (Manassas, VA), the FDA-CDC Antimicrobial Resistance Isolate Bank, and the Walter-Reed Army Institute of Research.

Catalog of nuclear magnetic resonance spectra:

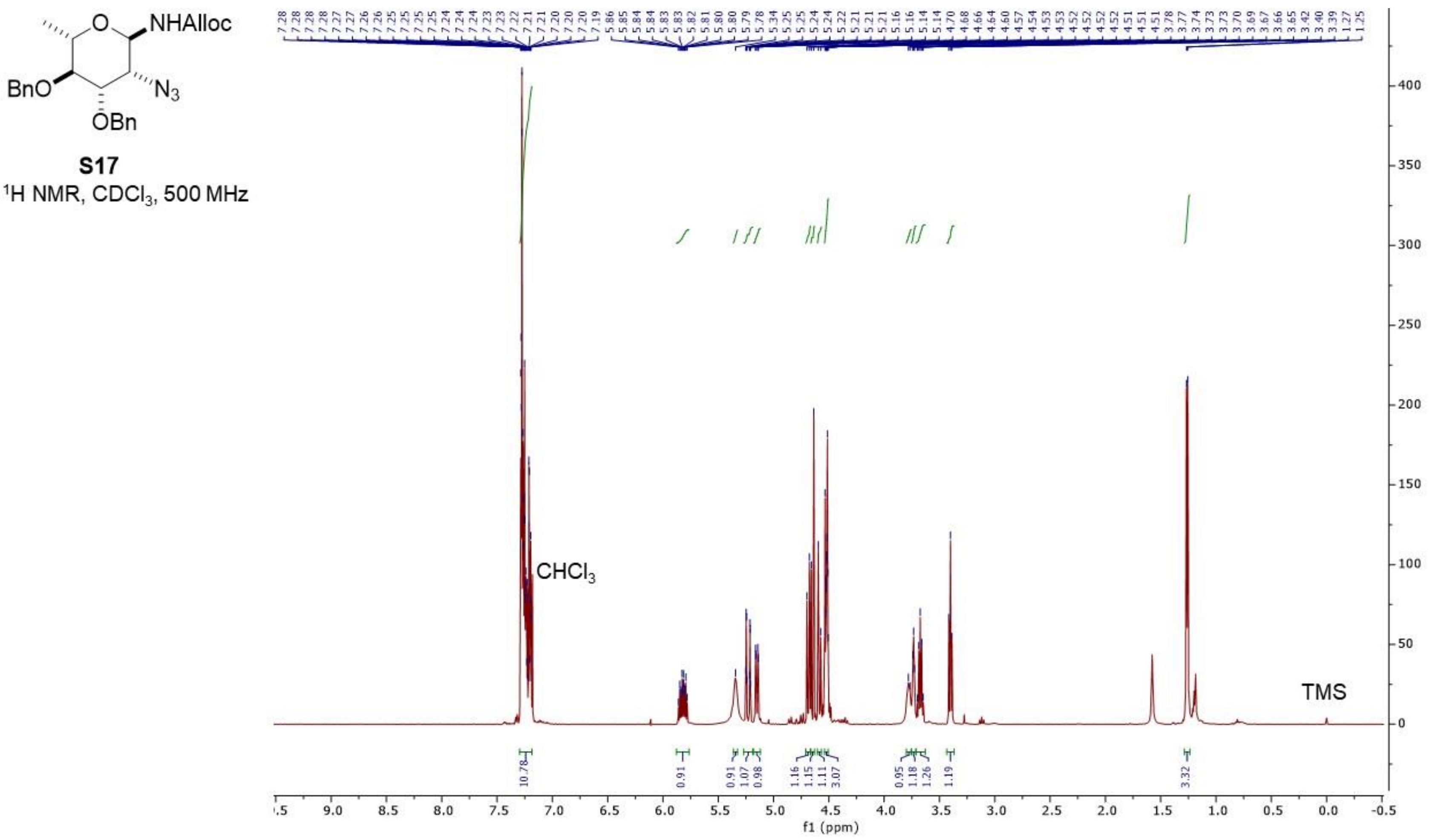

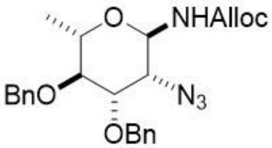

S17

^{13}C NMR, $CDCl_3$, 100 MHz

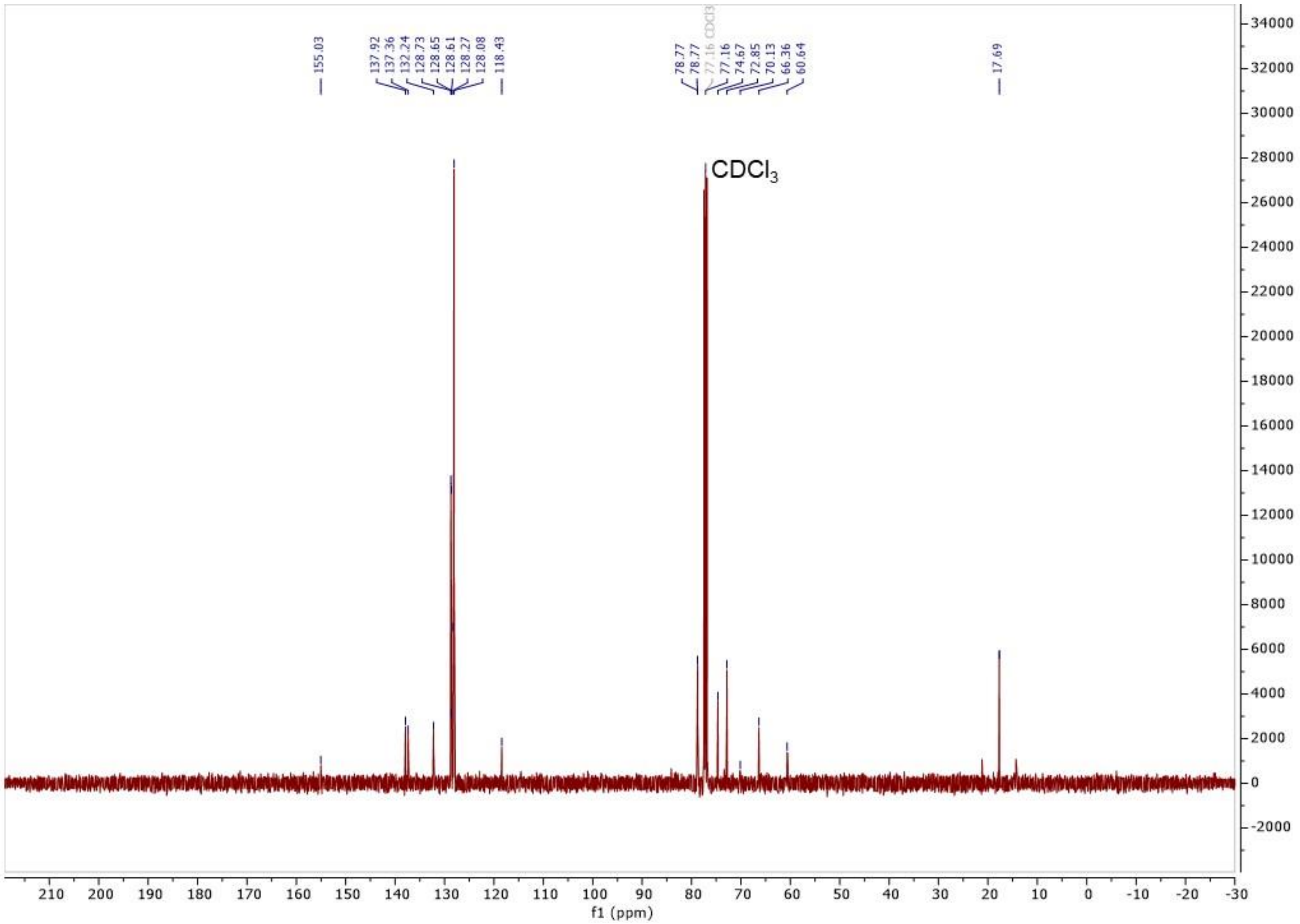

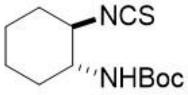

S18

^1H NMR, $CDCl_3$, 500 MHz

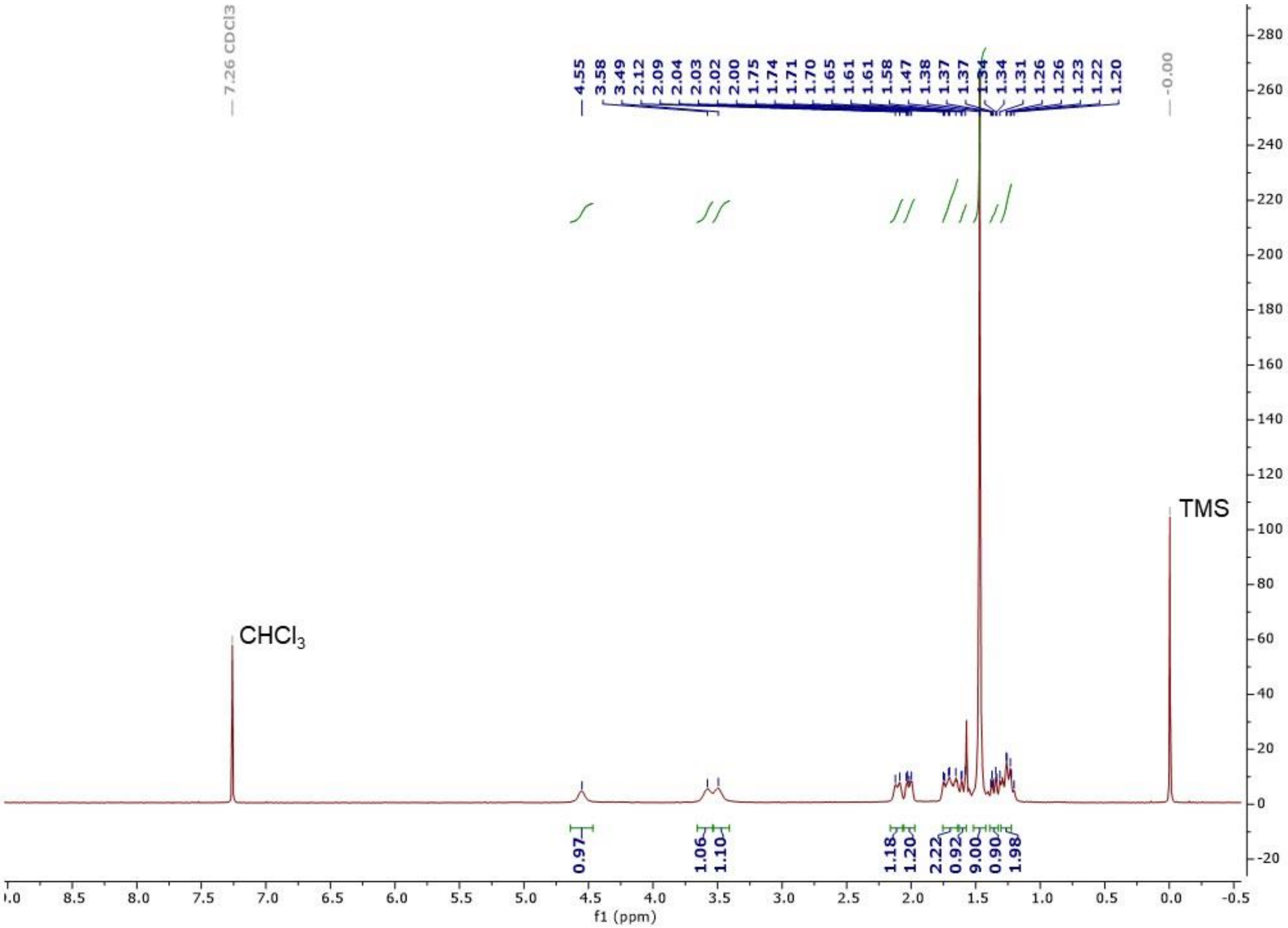

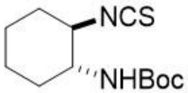

S18

^{13}C NMR, $CDCl_3$, 100 MHz

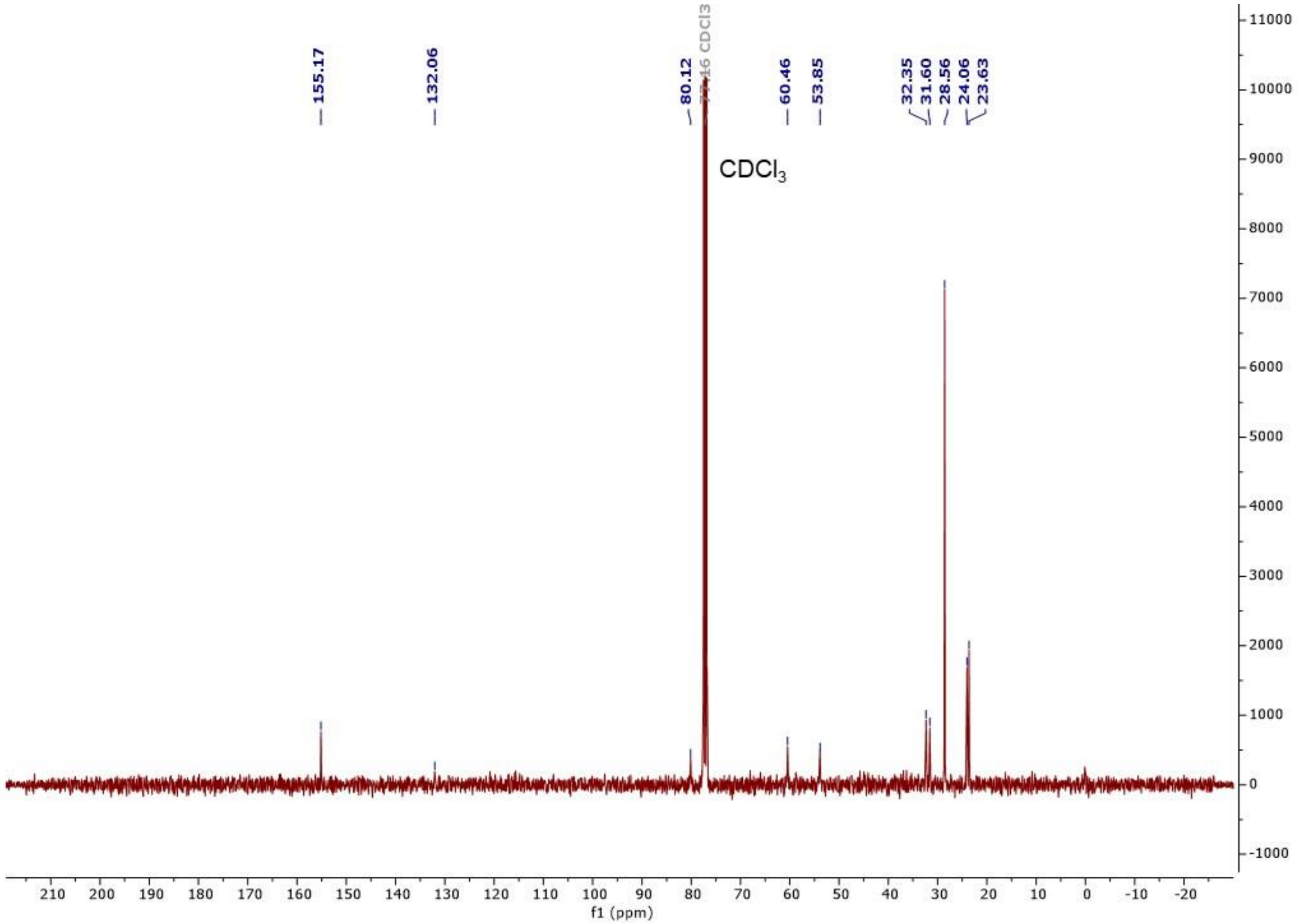

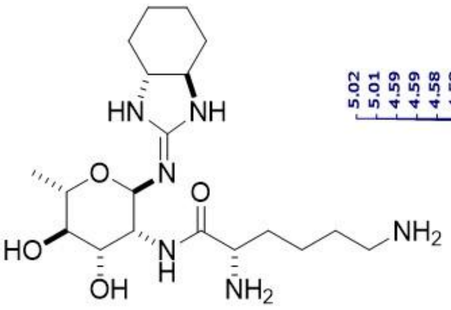

S19

^{1}H NMR, CD_3OD, 500 MHz

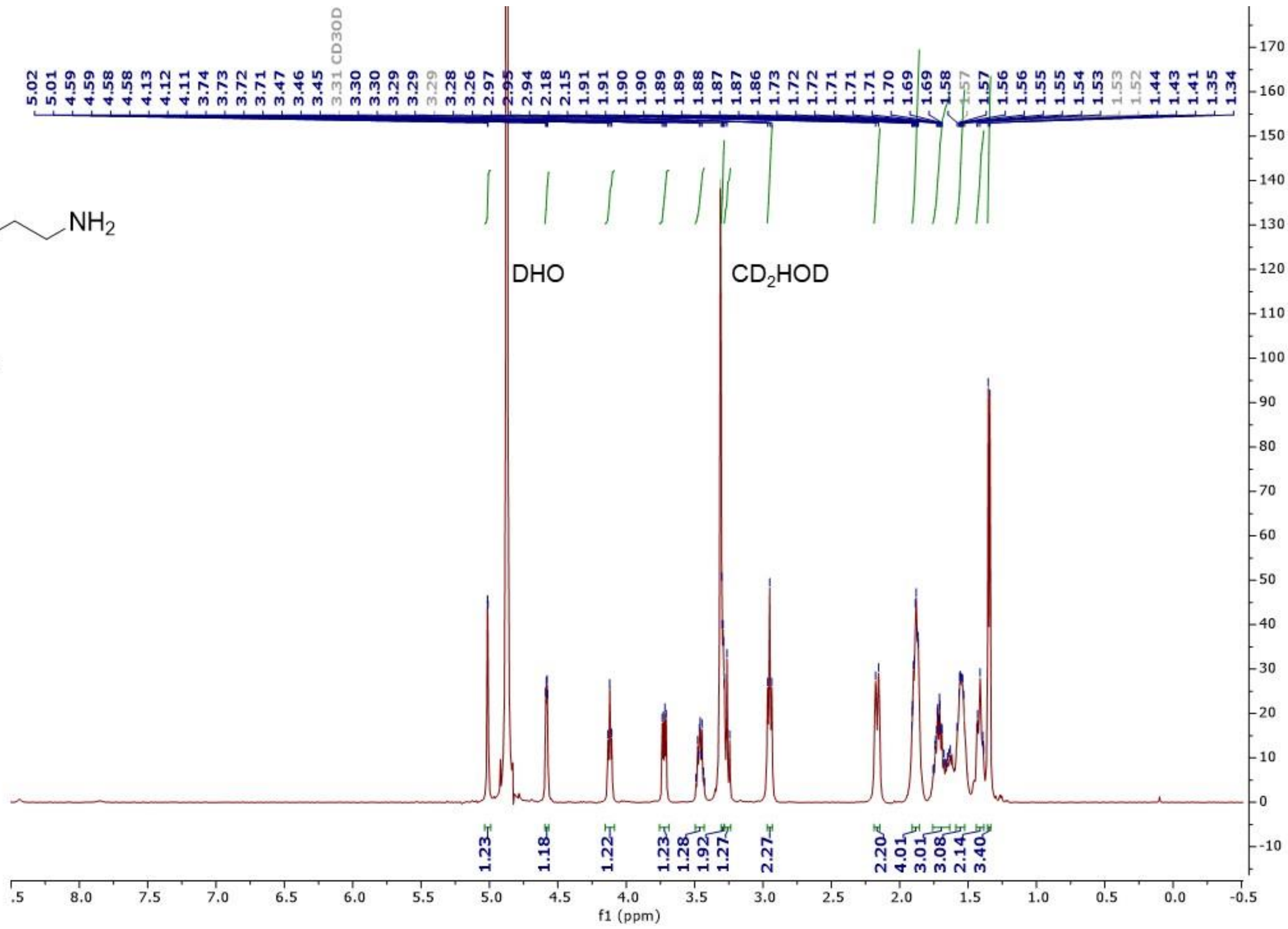

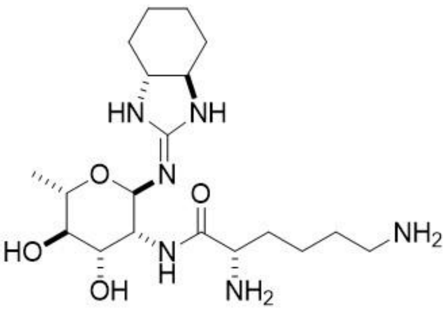

S19

^{13}C NMR, CD_3OD, 100 MHz

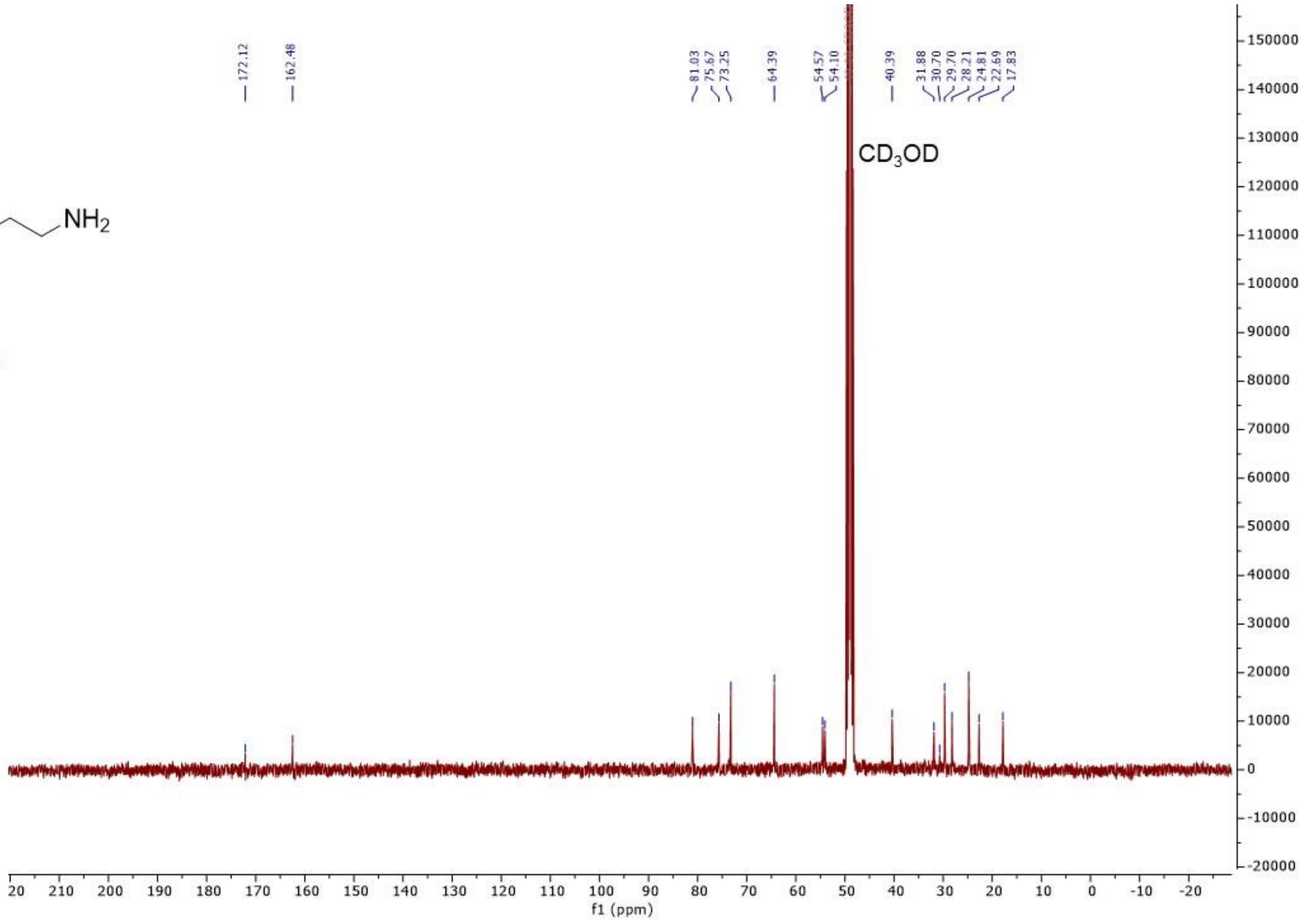

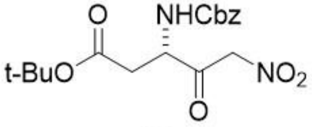

10

^{1}H NMR, $CDCl_3$, 500 MHz

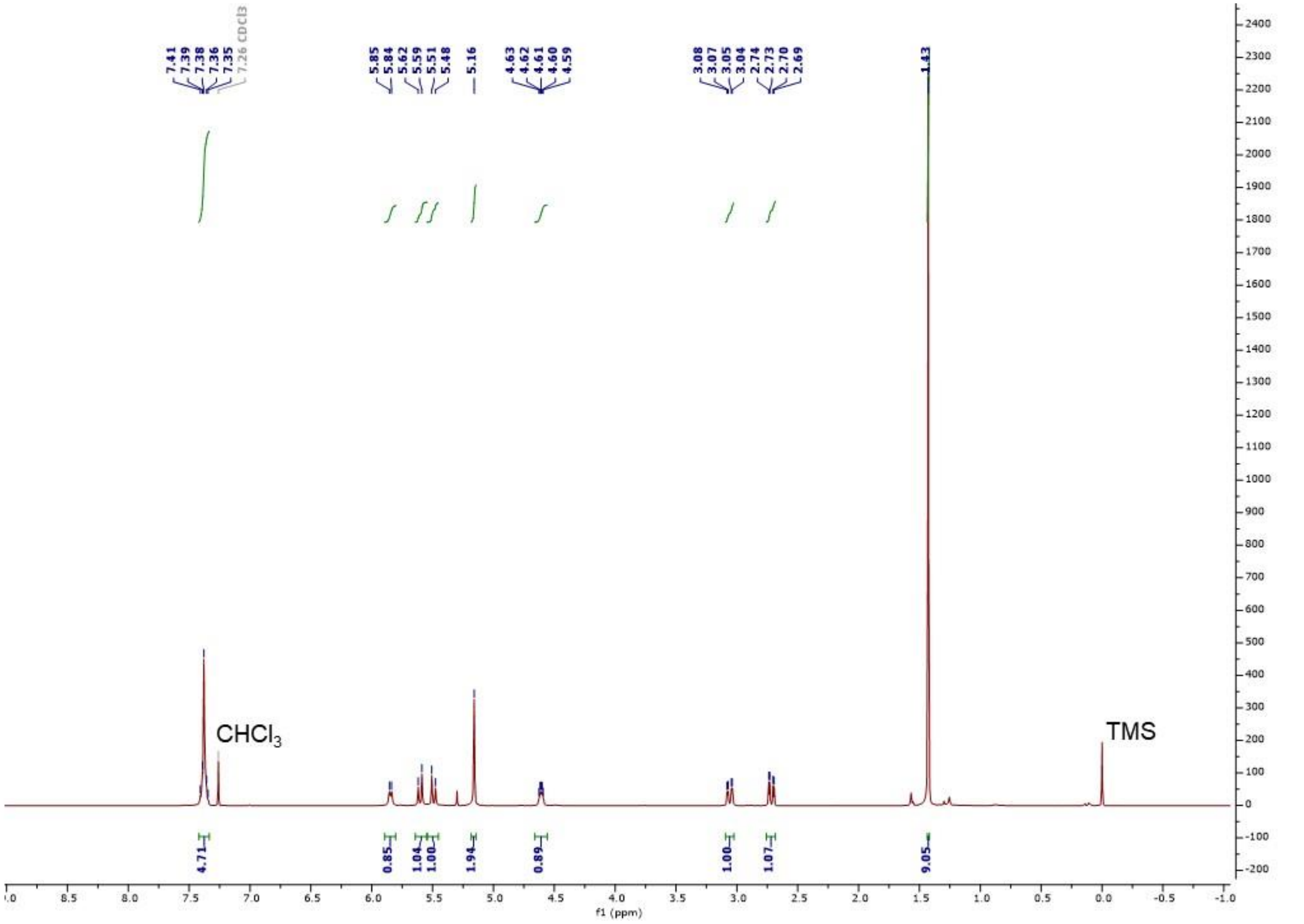

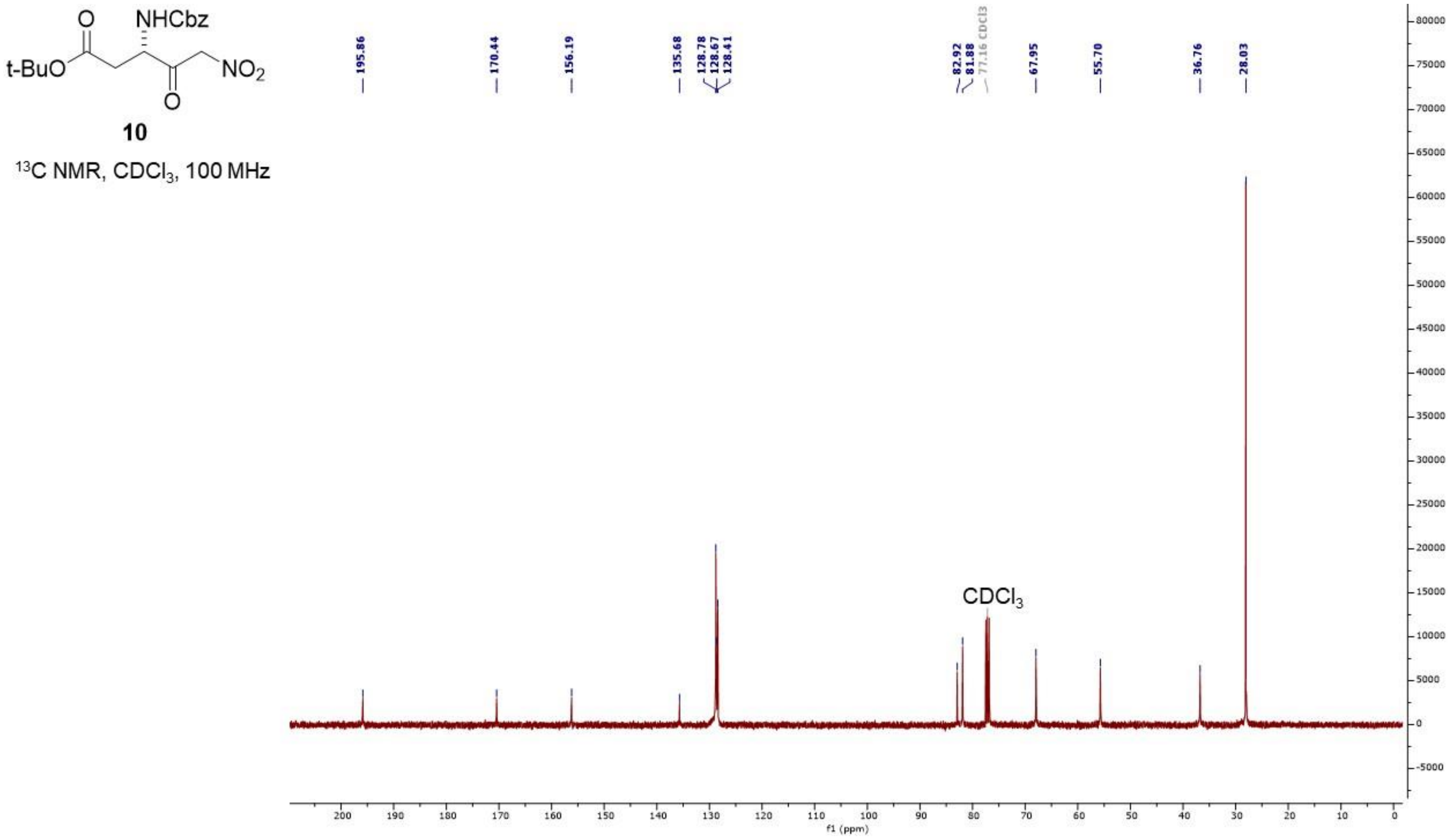
NHCbz
t-BuO
NO2
10
13C NMR, CDCl3, 100 MHz
195.86
170.44
156.19
135.68
128.78
128.67
128.41
82.92
81.88
77.16 CDCl3
67.95
55.70
36.76
28.03
CDCl3
f1 (ppm)

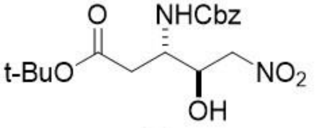

11

^{1}H NMR, CD_3OD, 500 MHz

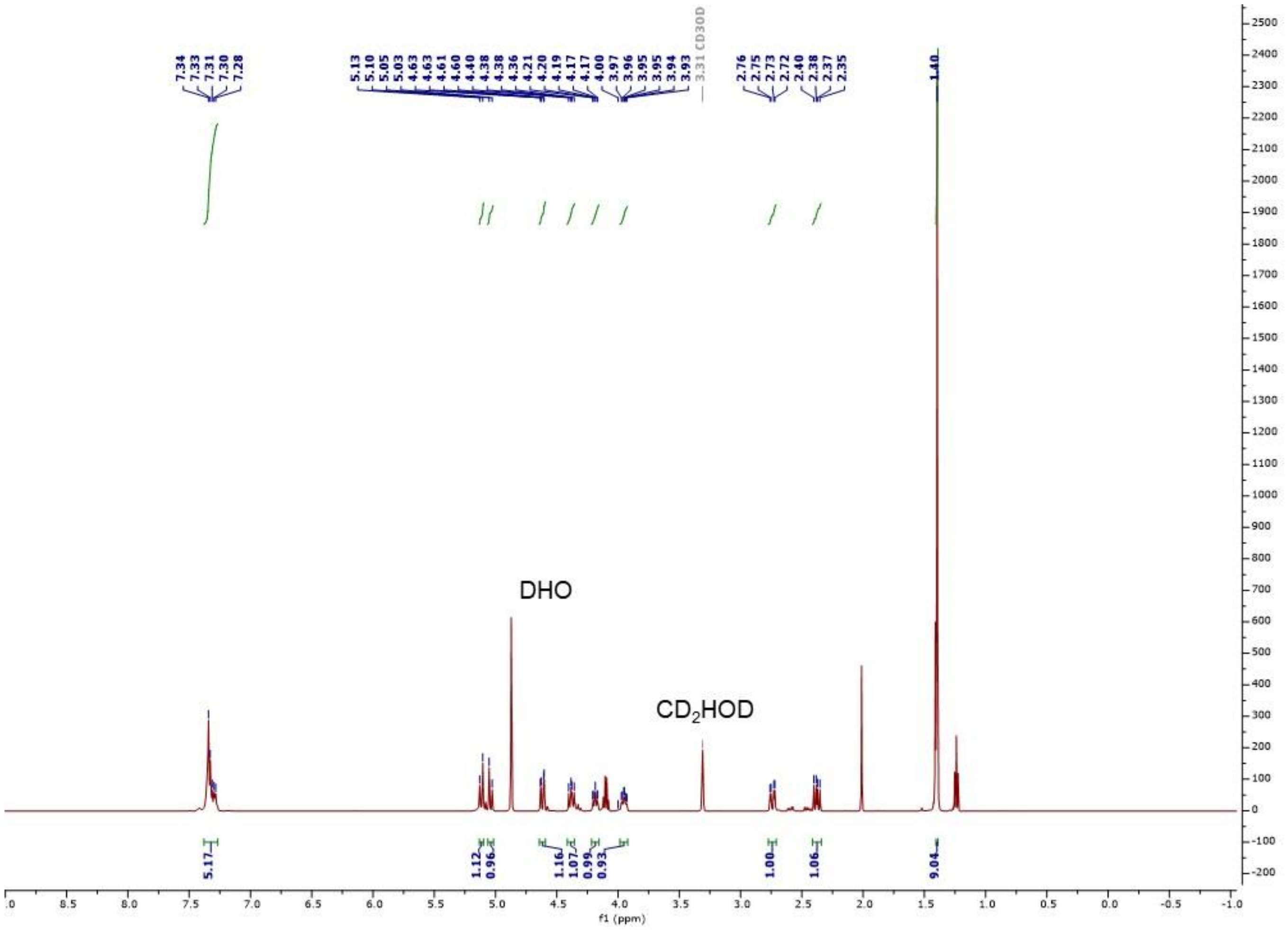

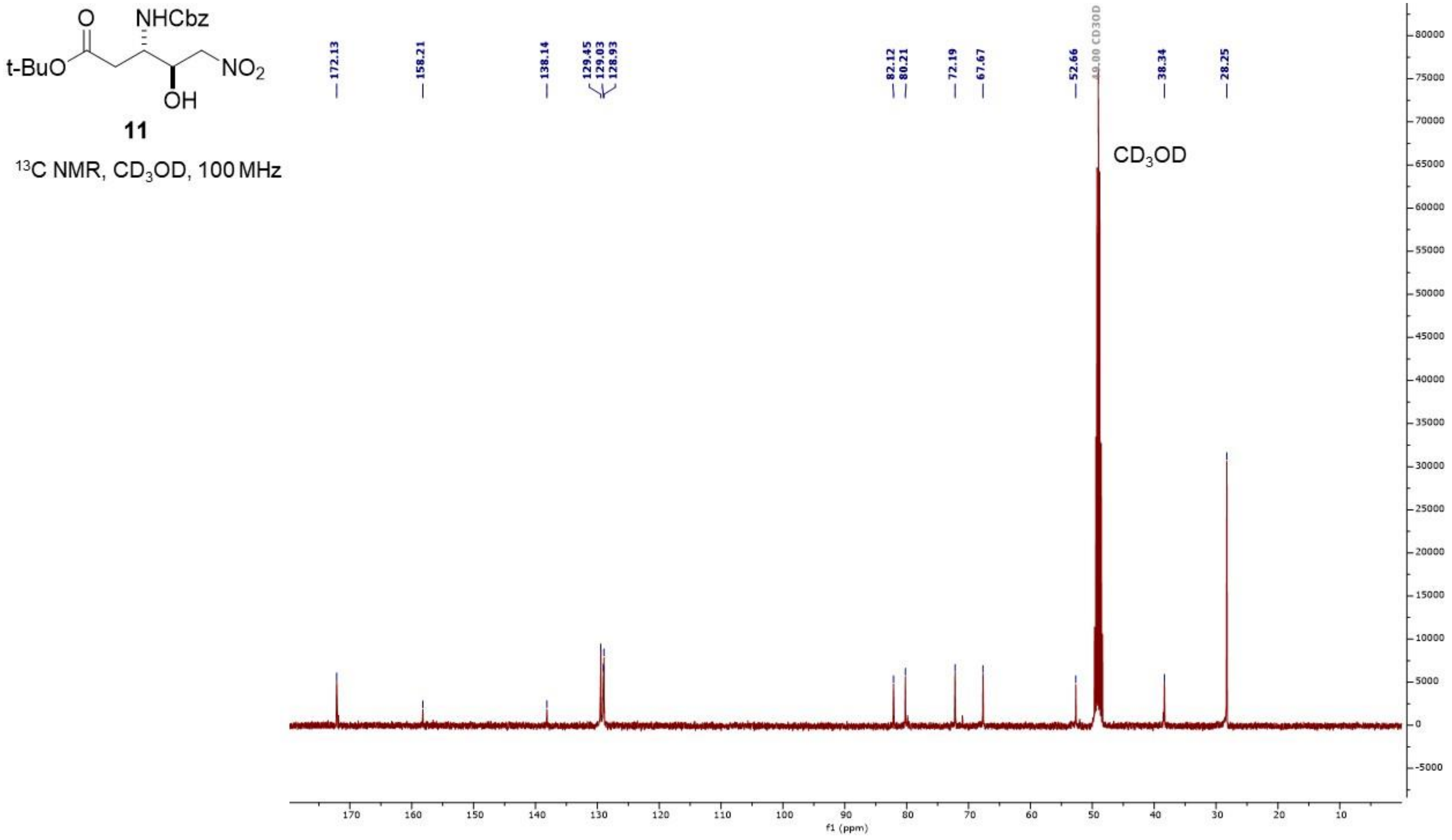

O
NHCbz
t-BuO
NO2
OH
11
13C NMR, CD3OD, 100 MHz
172.13
158.21
138.14
129.45
129.03
128.93
82.12
80.21
72.19
67.67
52.66
49.00 CD3OD
38.34
28.25
CD3OD
f1 (ppm)

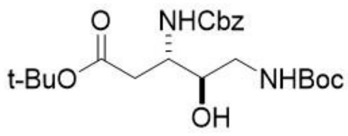

12

^{1}H NMR, $CDCl_3$, 500 MHz

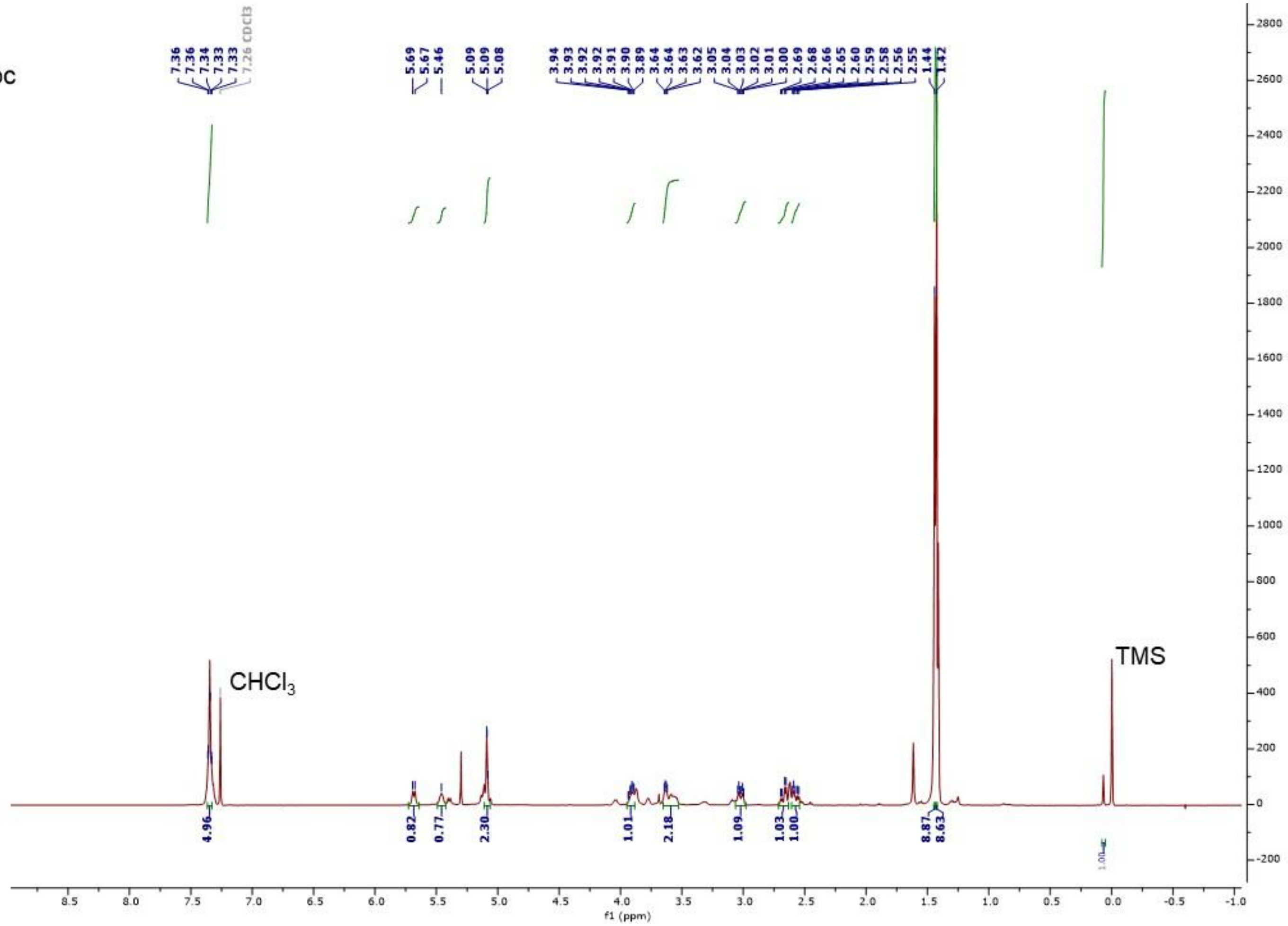

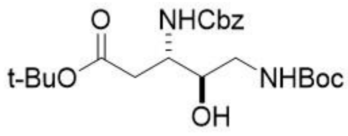

12

^{13}C NMR, $CDCl_3$, 125 MHz

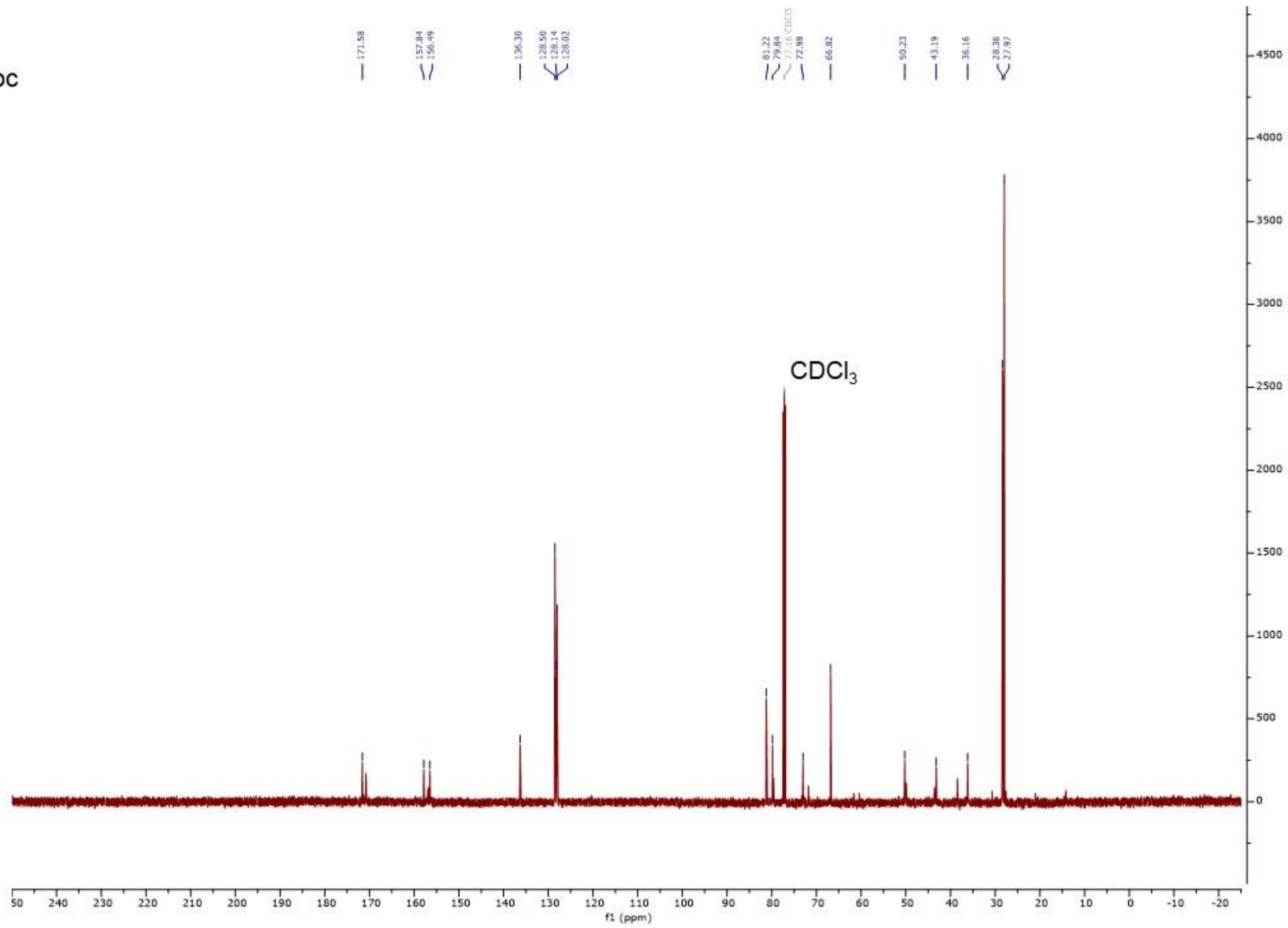

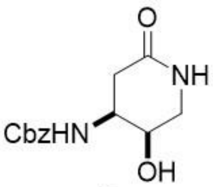

13

^{1}H NMR, CD_3OD, 500 MHz

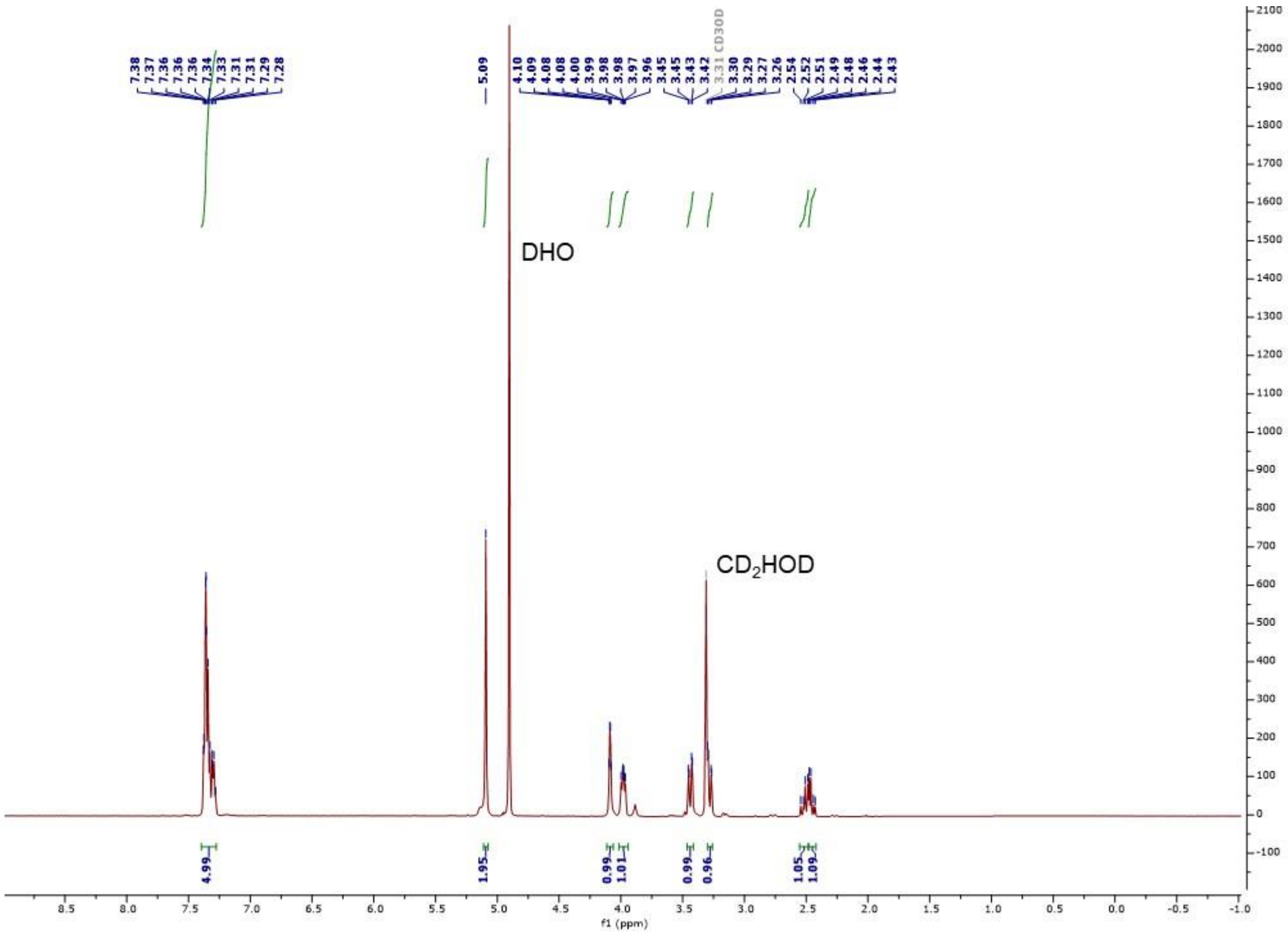

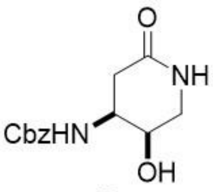

13

^{13}C NMR, $(CD_3)_2SO$, 125 MHz

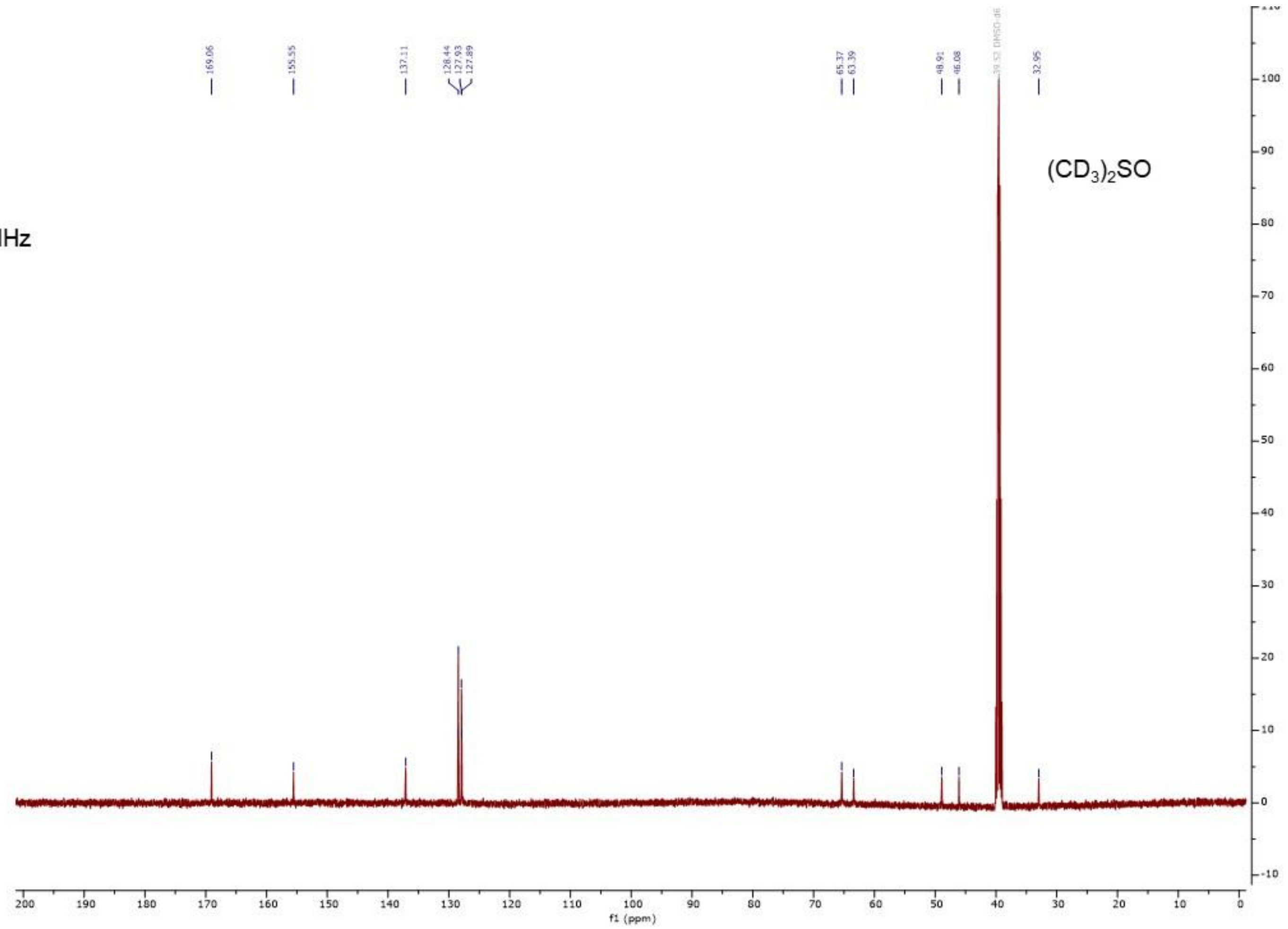

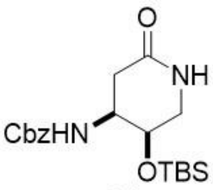

S1

^{1}H NMR, $CDCl_3$, 500 MHz

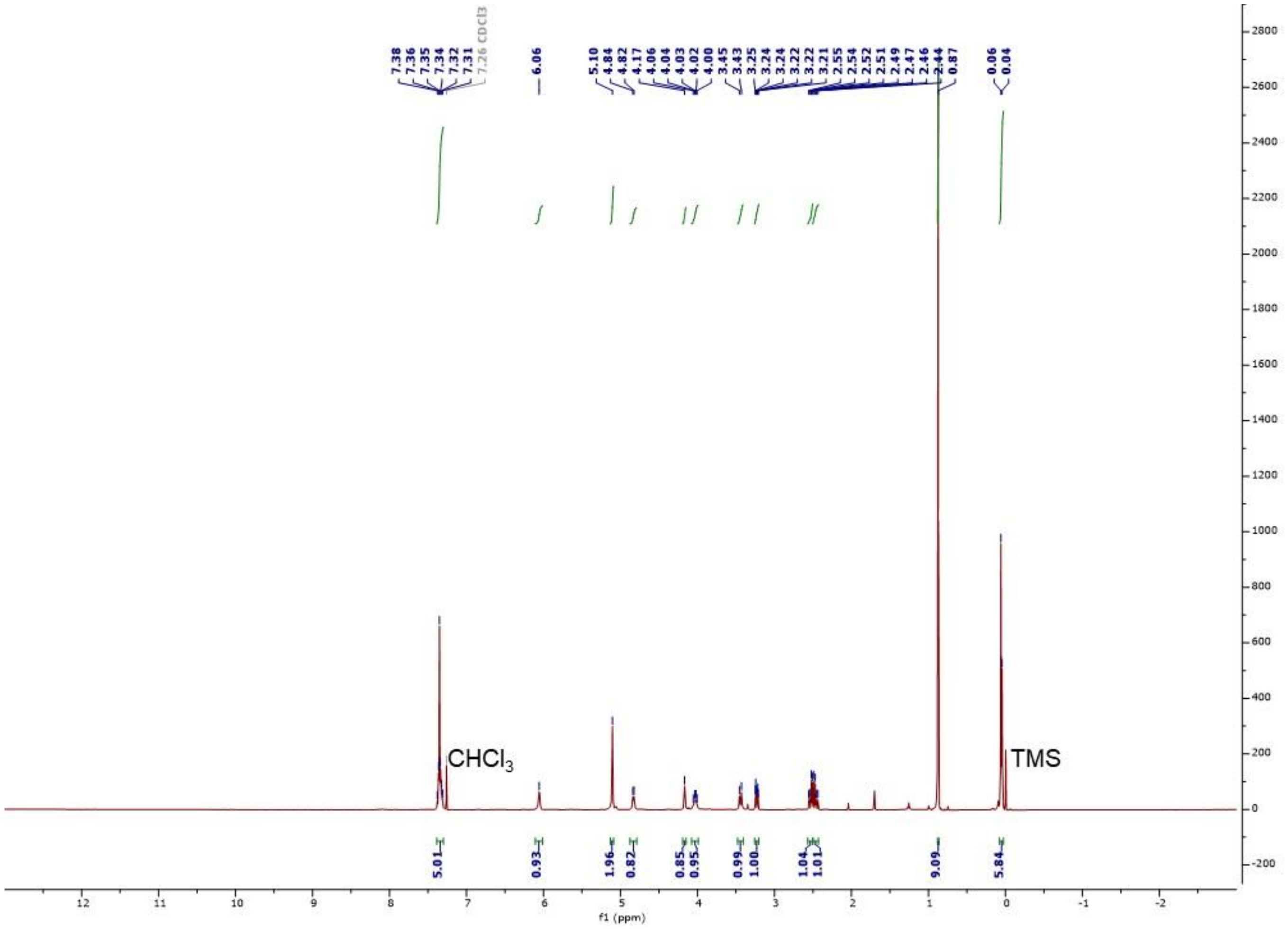

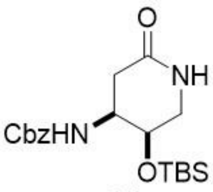

S1

^{13}C NMR, $CDCl_3$, 100 MHz

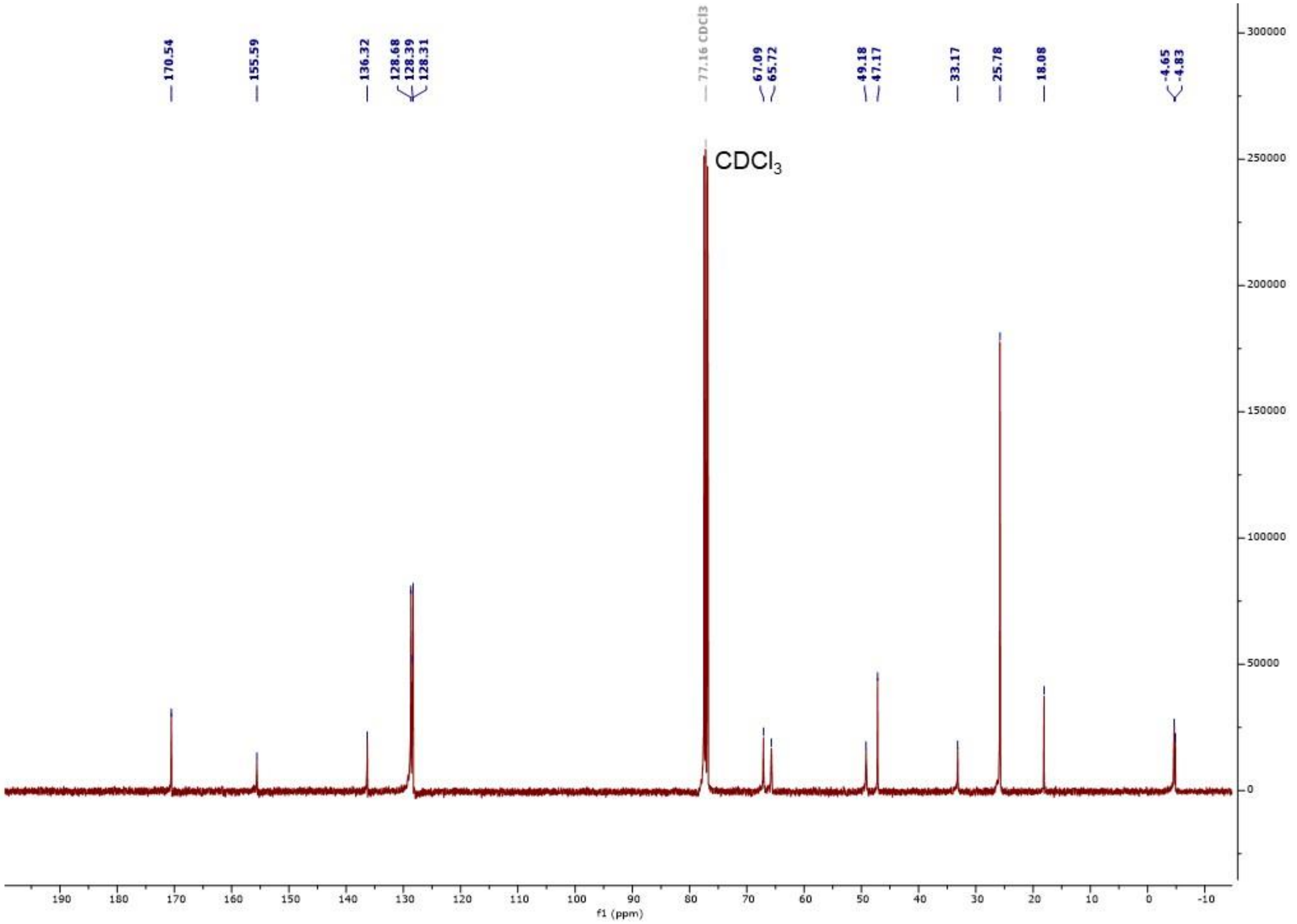

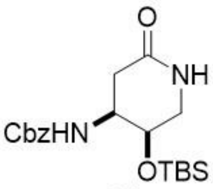

S1

COSY, $CDCl_3$, 500 MHz

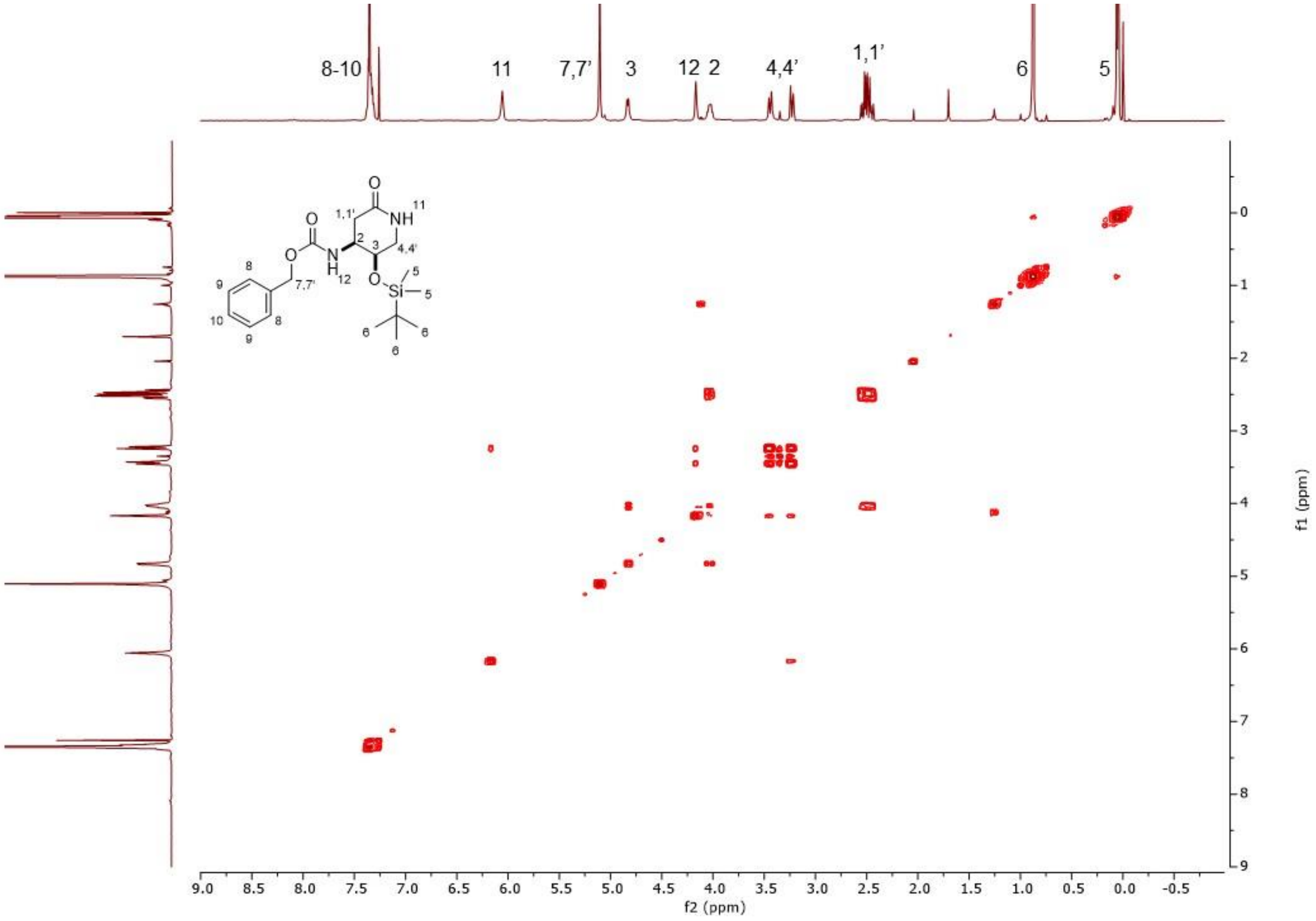

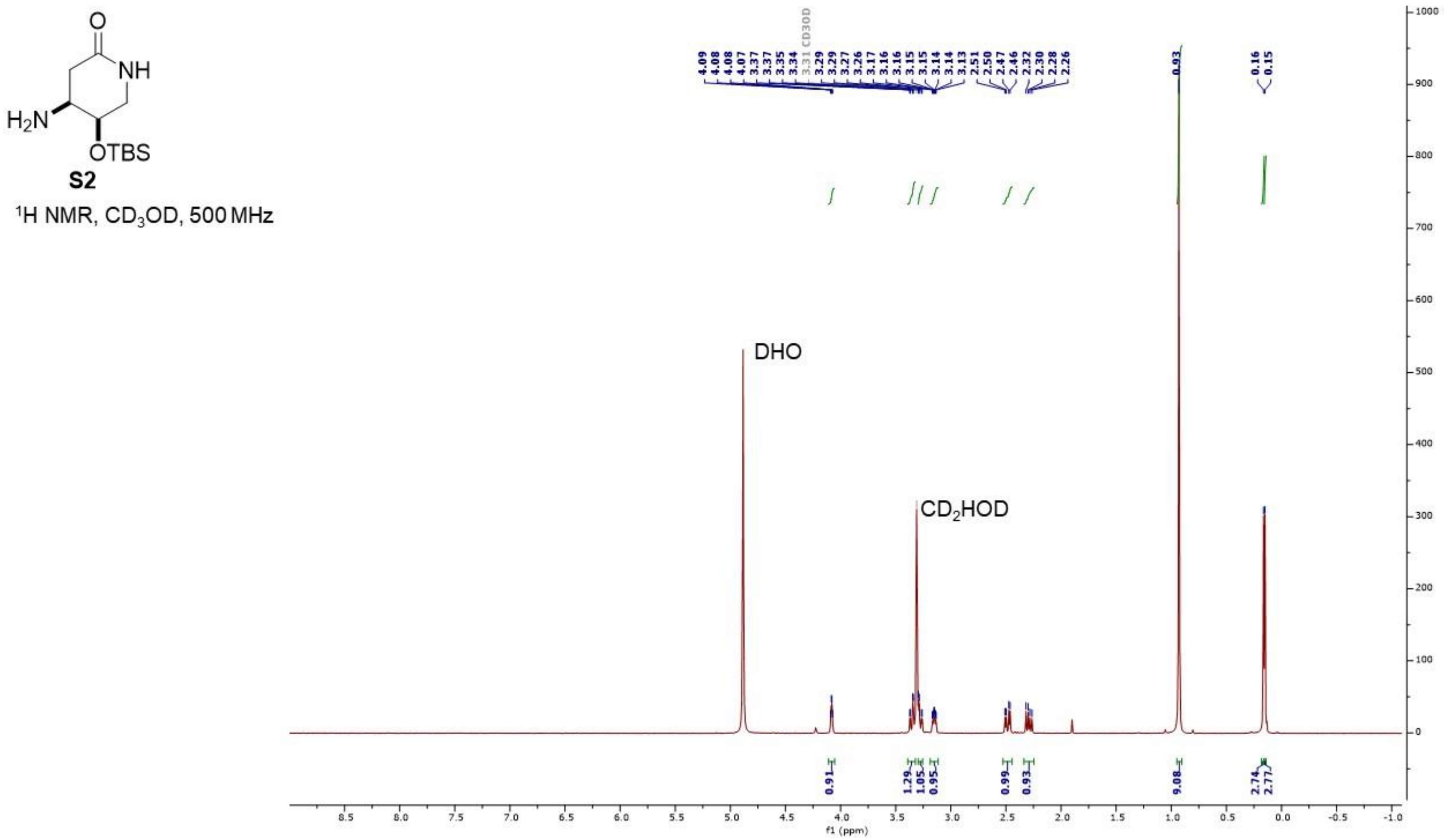

S2
^{1}H NMR, CD_3OD, 500 MHz
DHO
CD_2HOD
f1 (ppm)

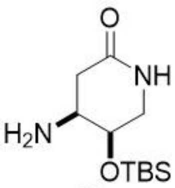

S2

^{13}C NMR, $CDCl_3$, 125 MHz

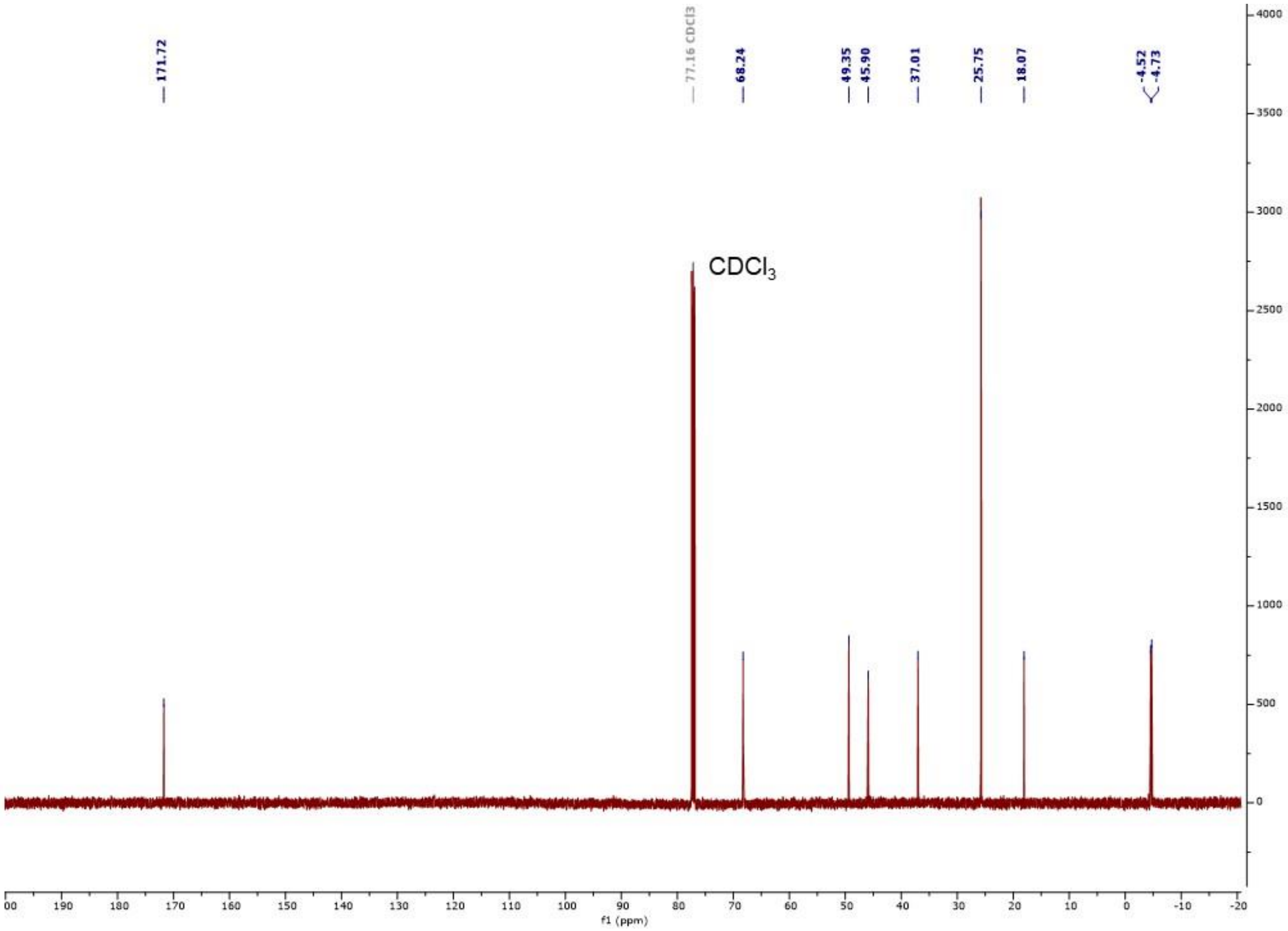

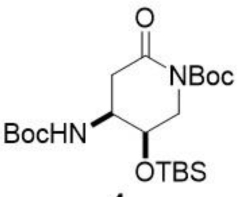

4

^{1}H NMR, $CDCl_3$, 500 MHz

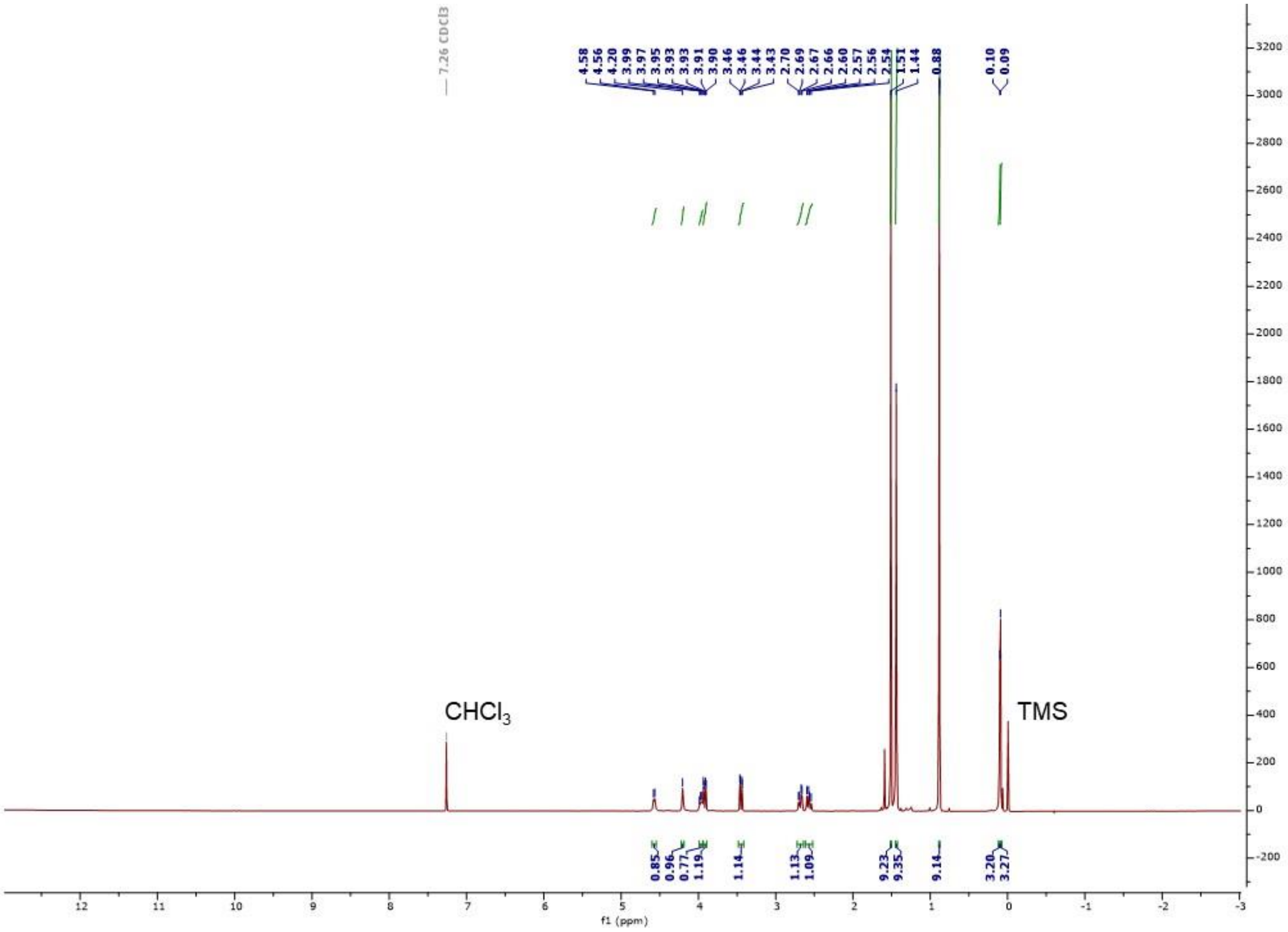

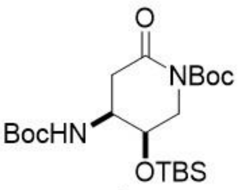

4

^{13}C NMR, $CDCl_3$, 125 MHz

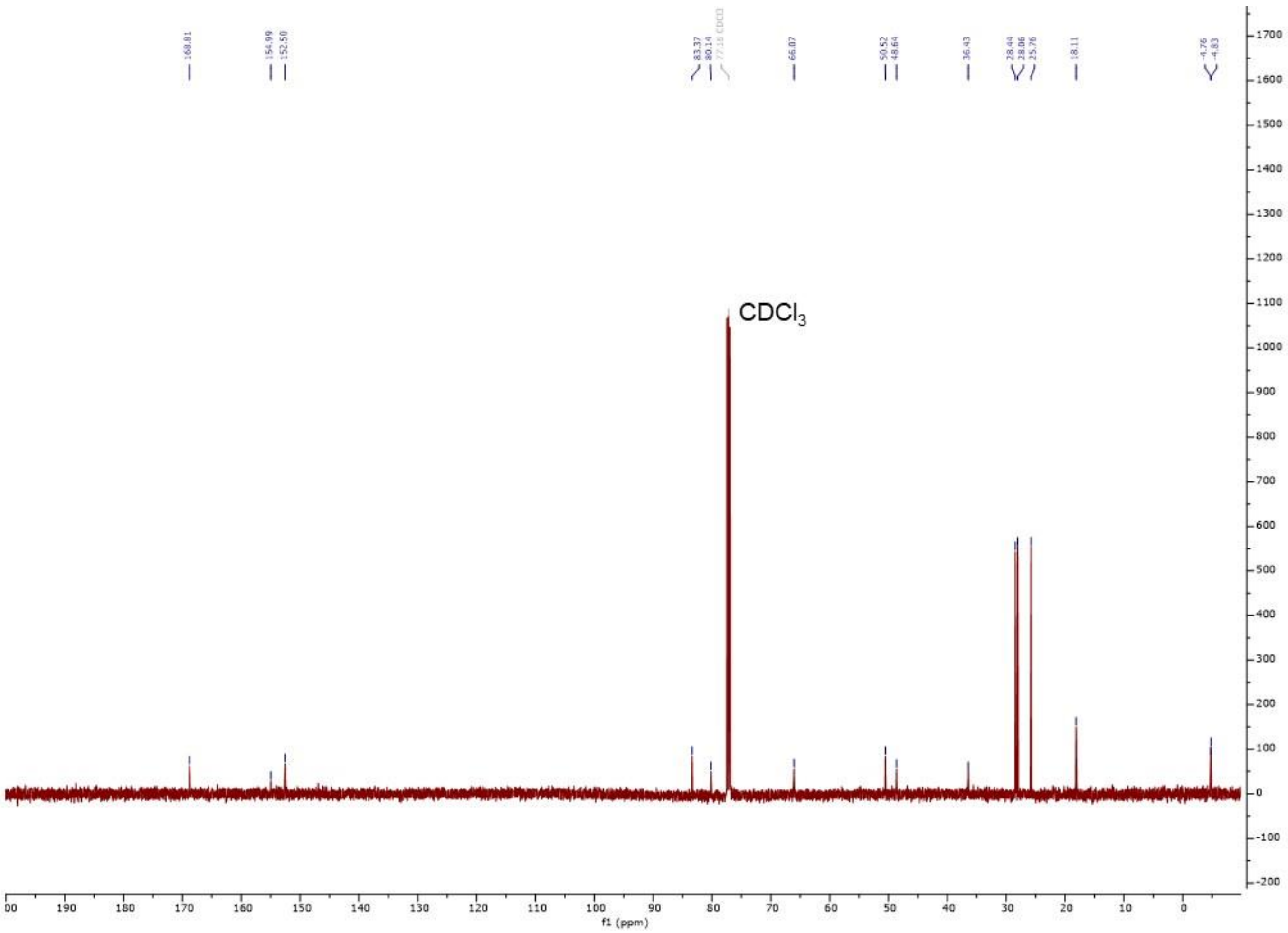

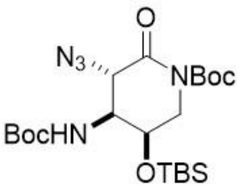

15

^{1}H NMR, $CDCl_3$, 500 MHz

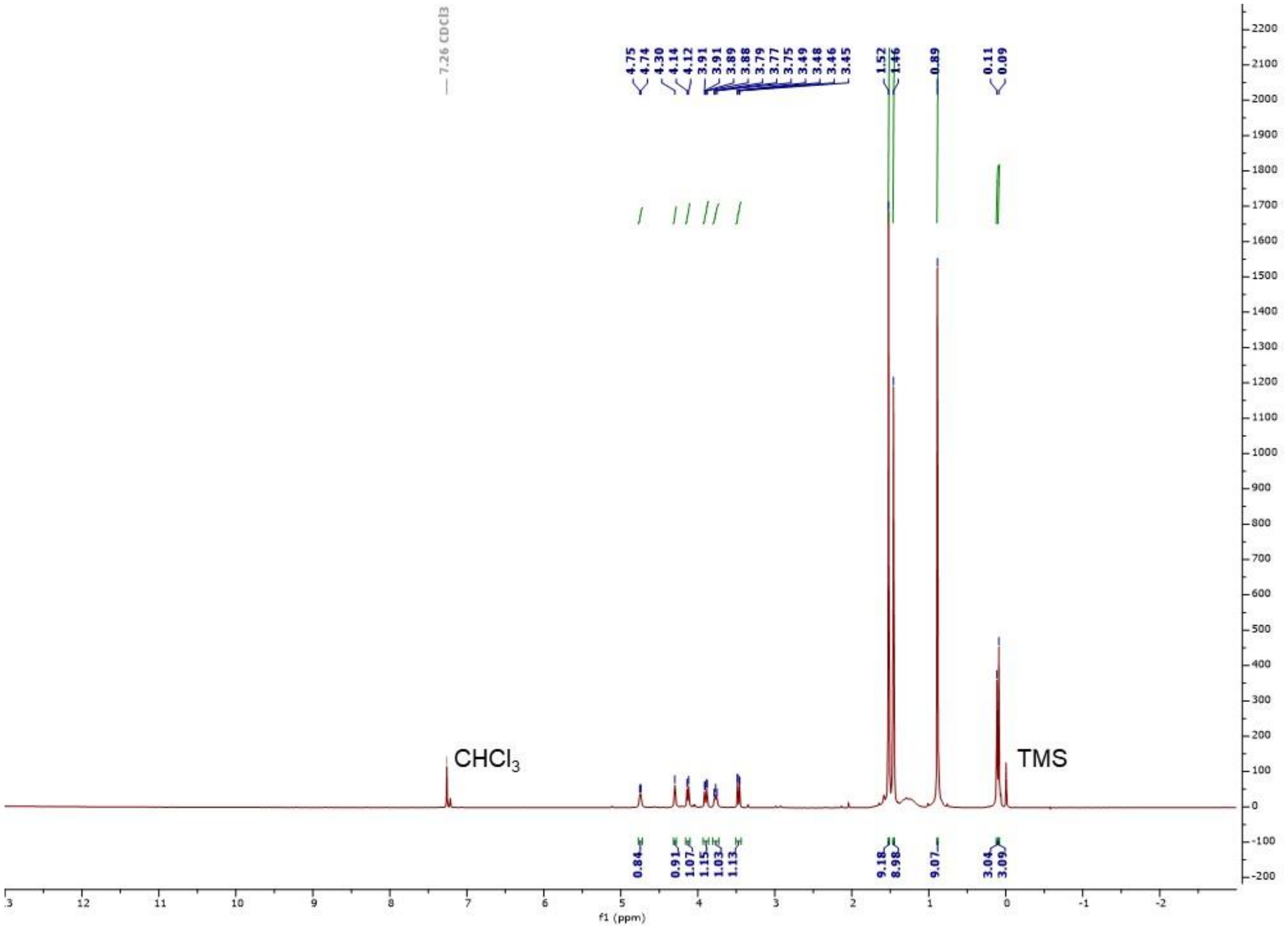

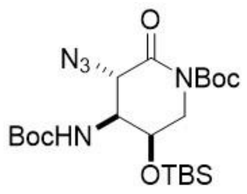

15

^{13}C NMR, $CDCl_3$, 125 MHz

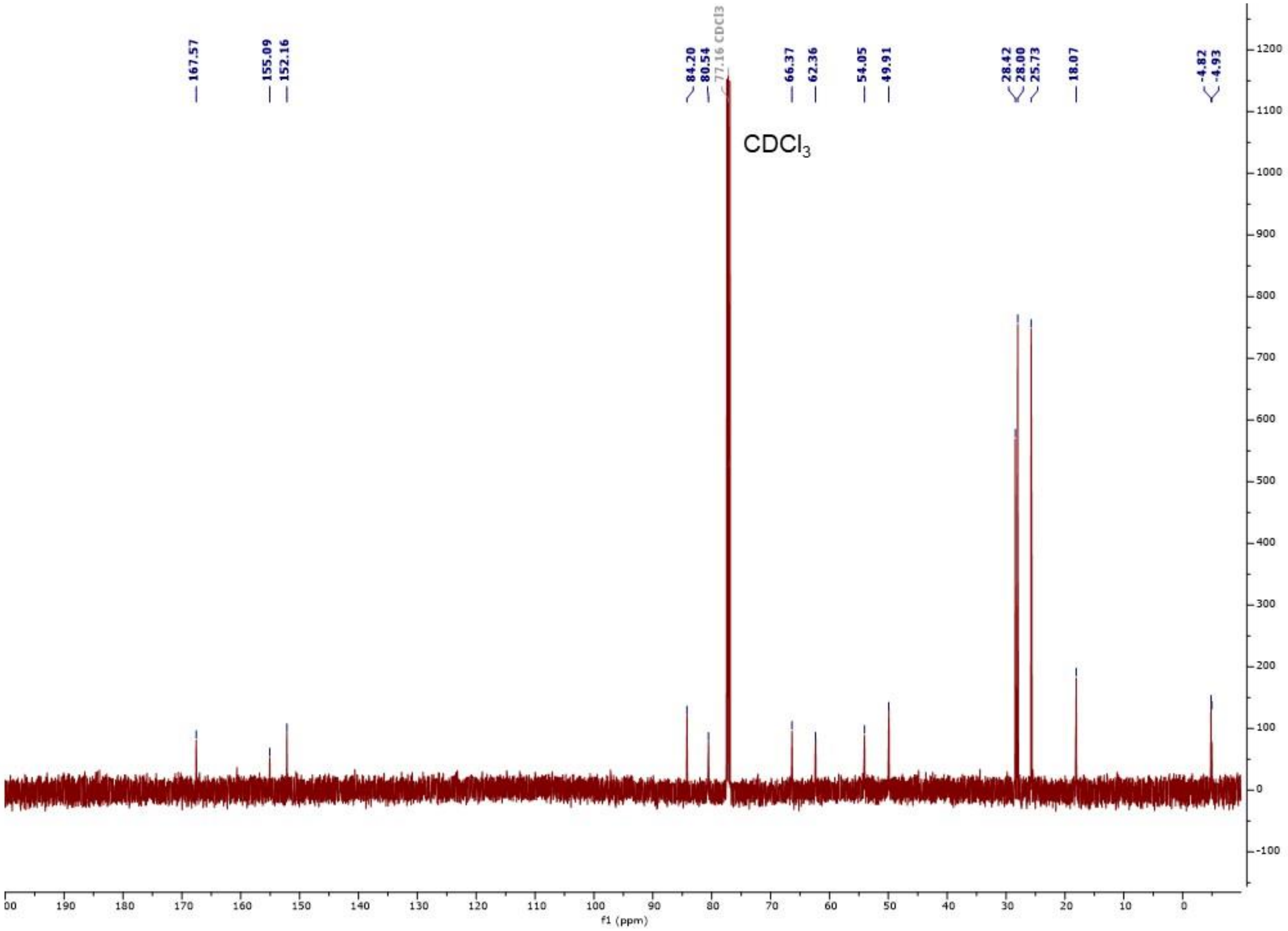

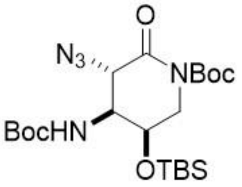

15

COSY, $CDCl_3$, 500 MHz

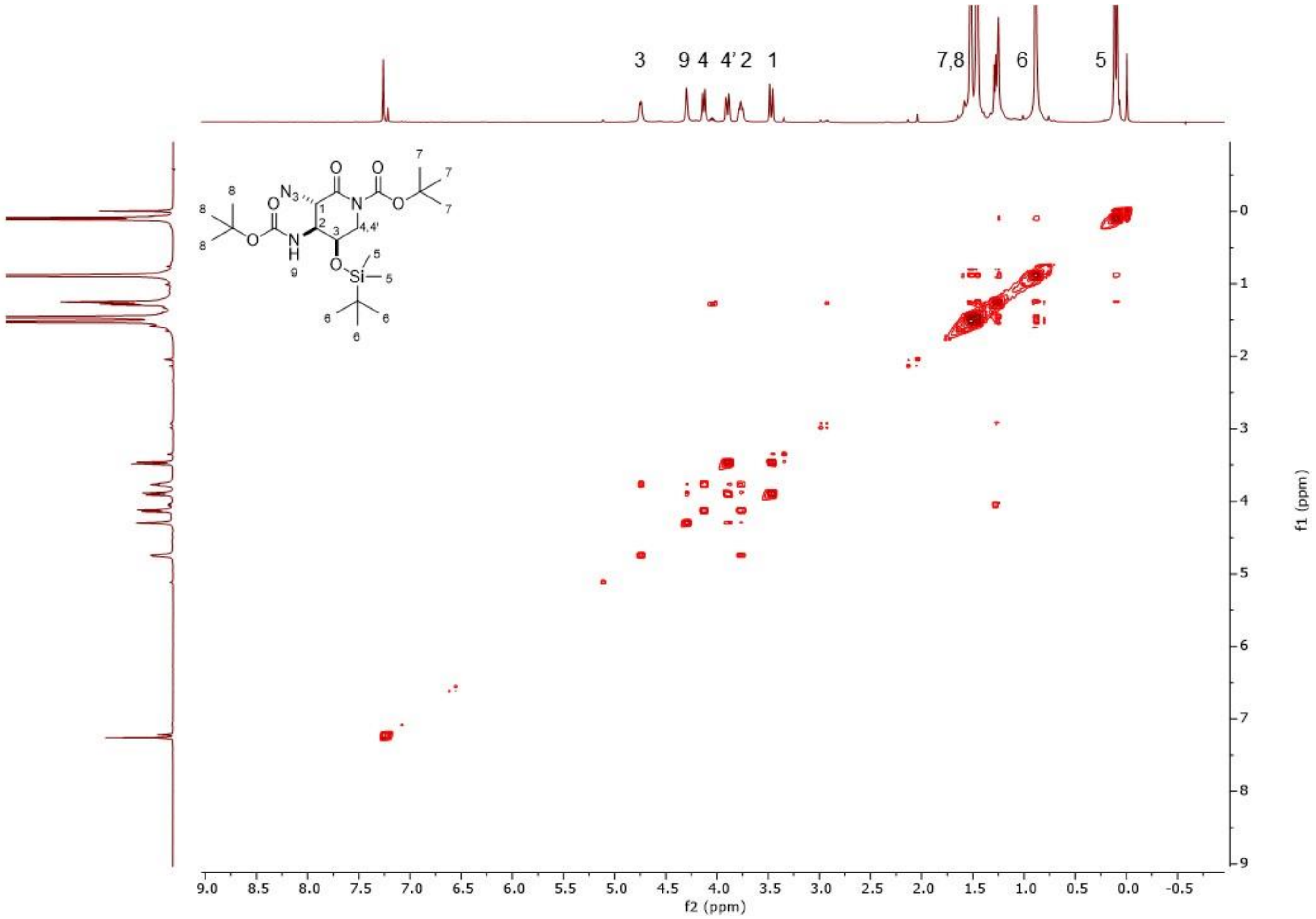

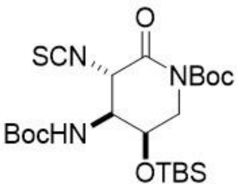

3

^1H NMR, $CDCl_3$, 500 MHz

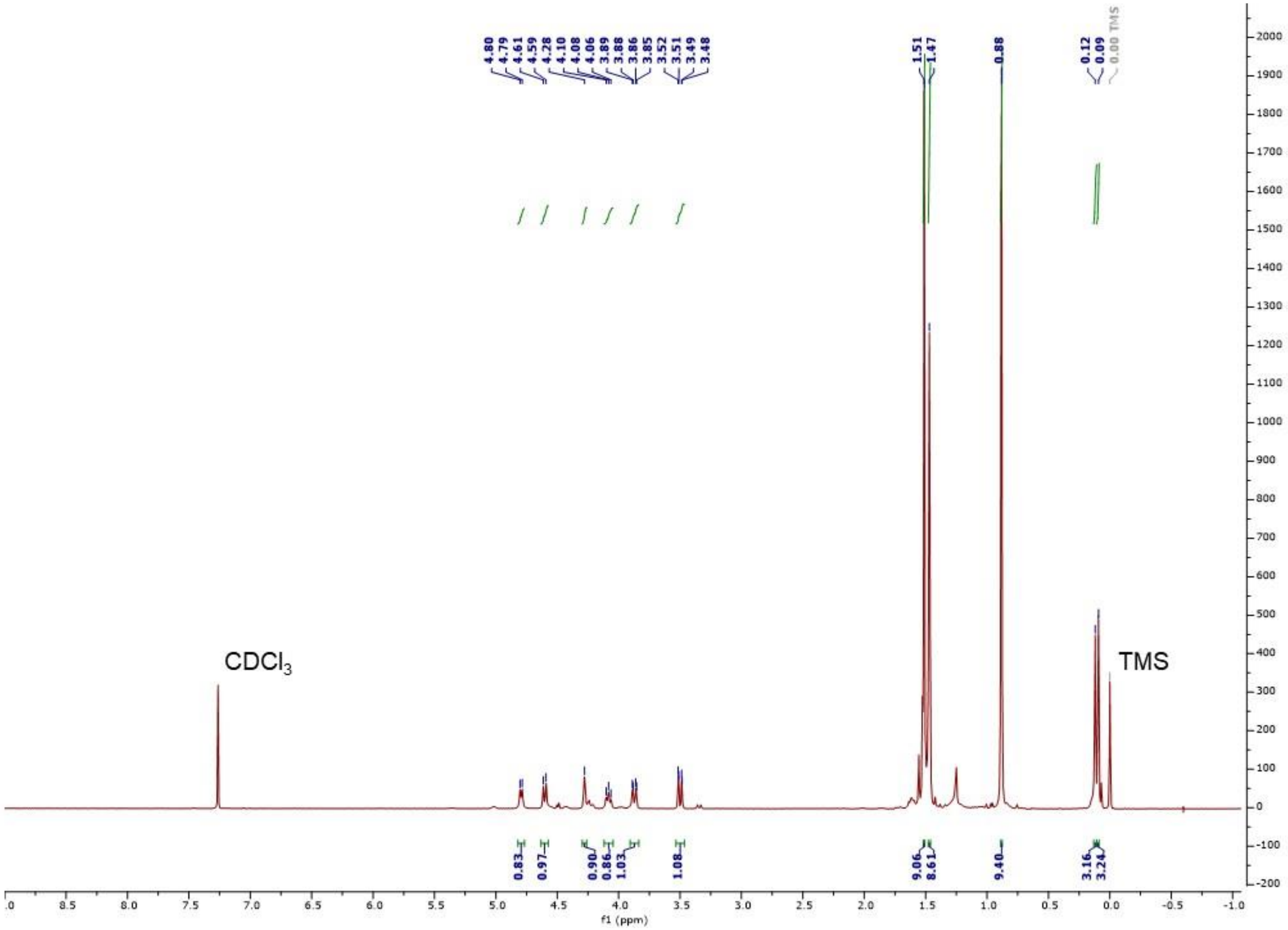

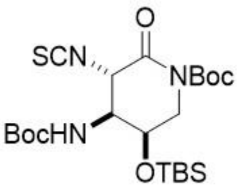

3

^{13}C NMR, $CDCl_3$, 125 MHz

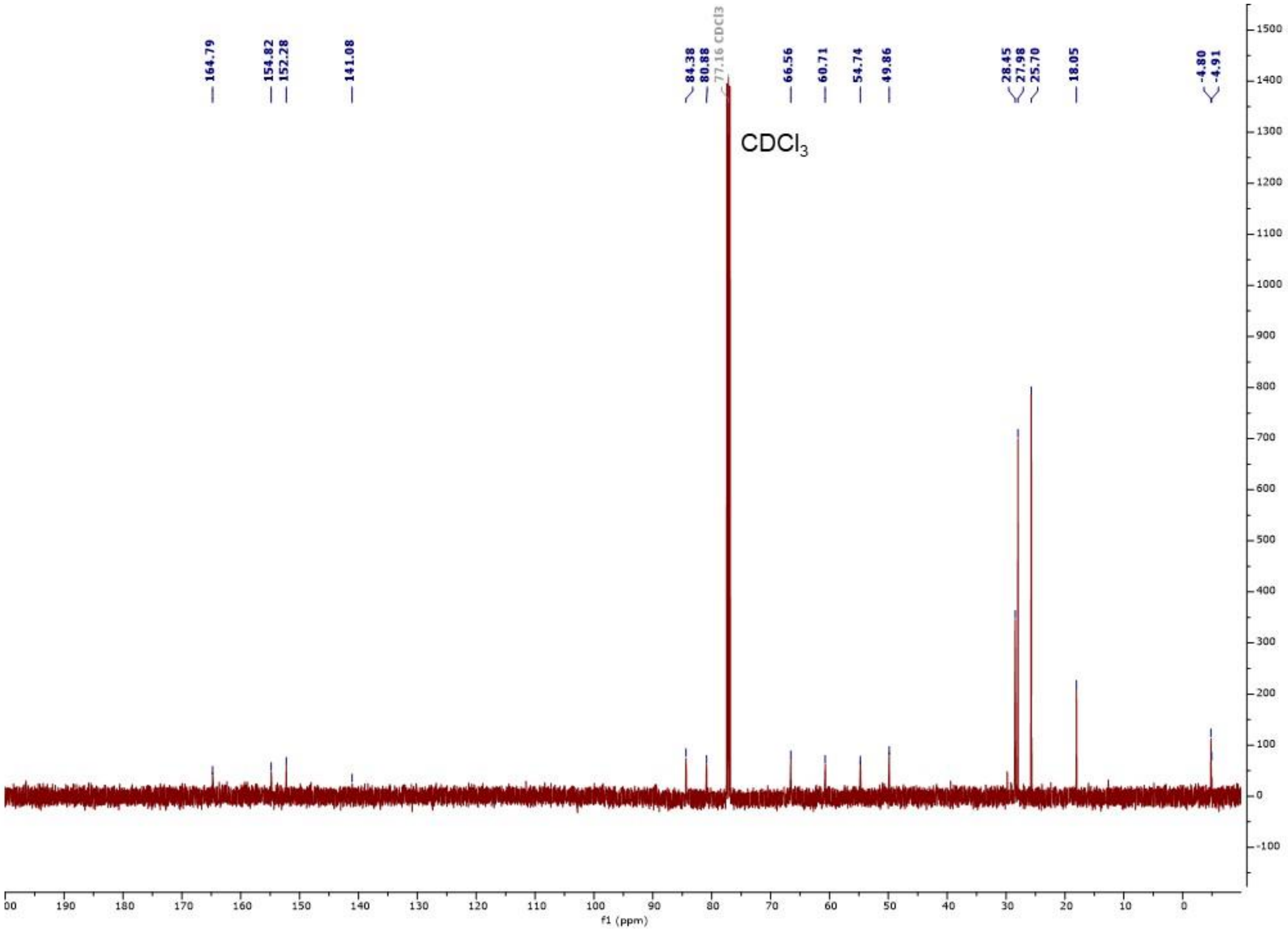

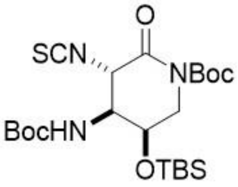

3

COSY, $CDCl_3$, 500 MHz

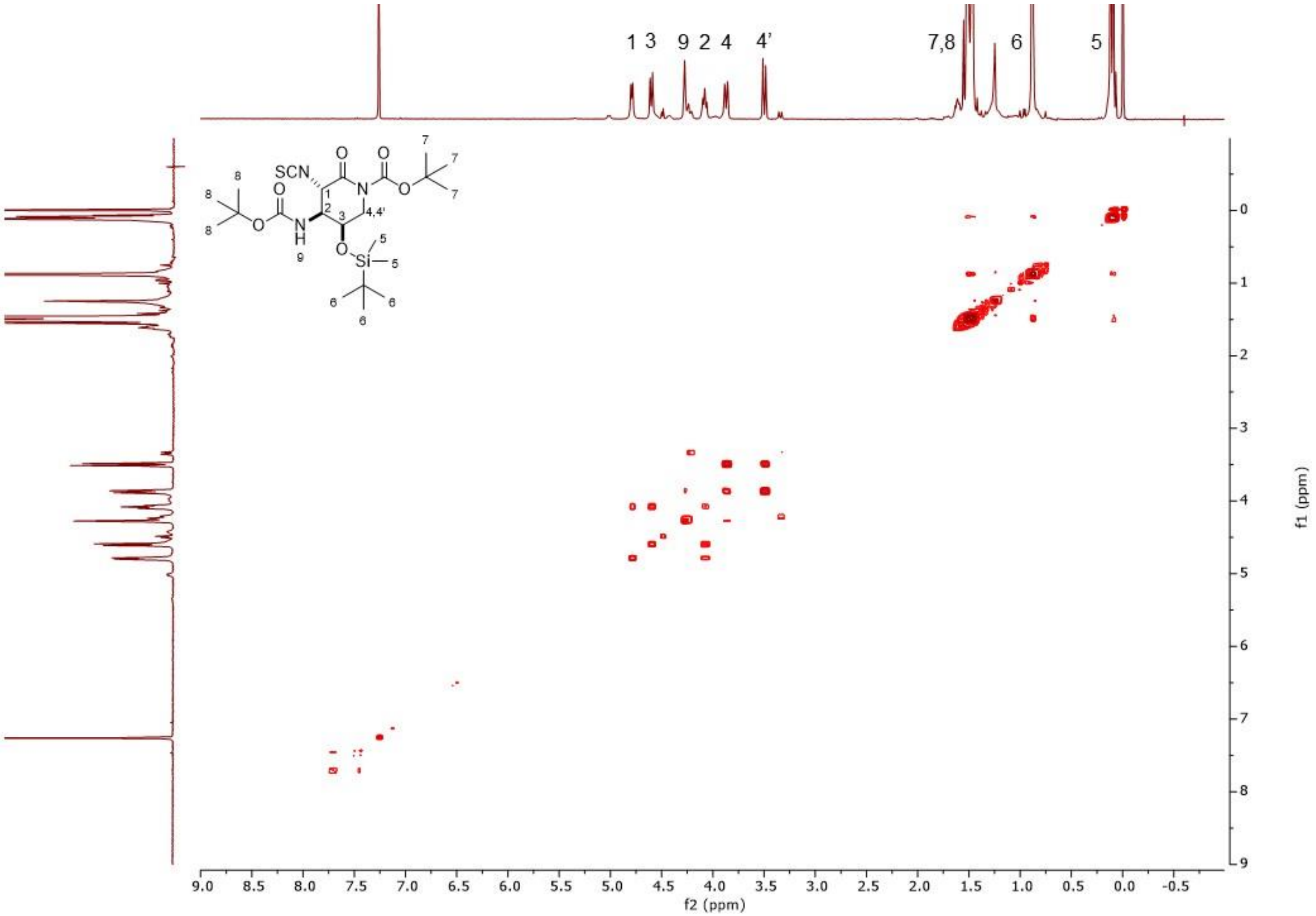

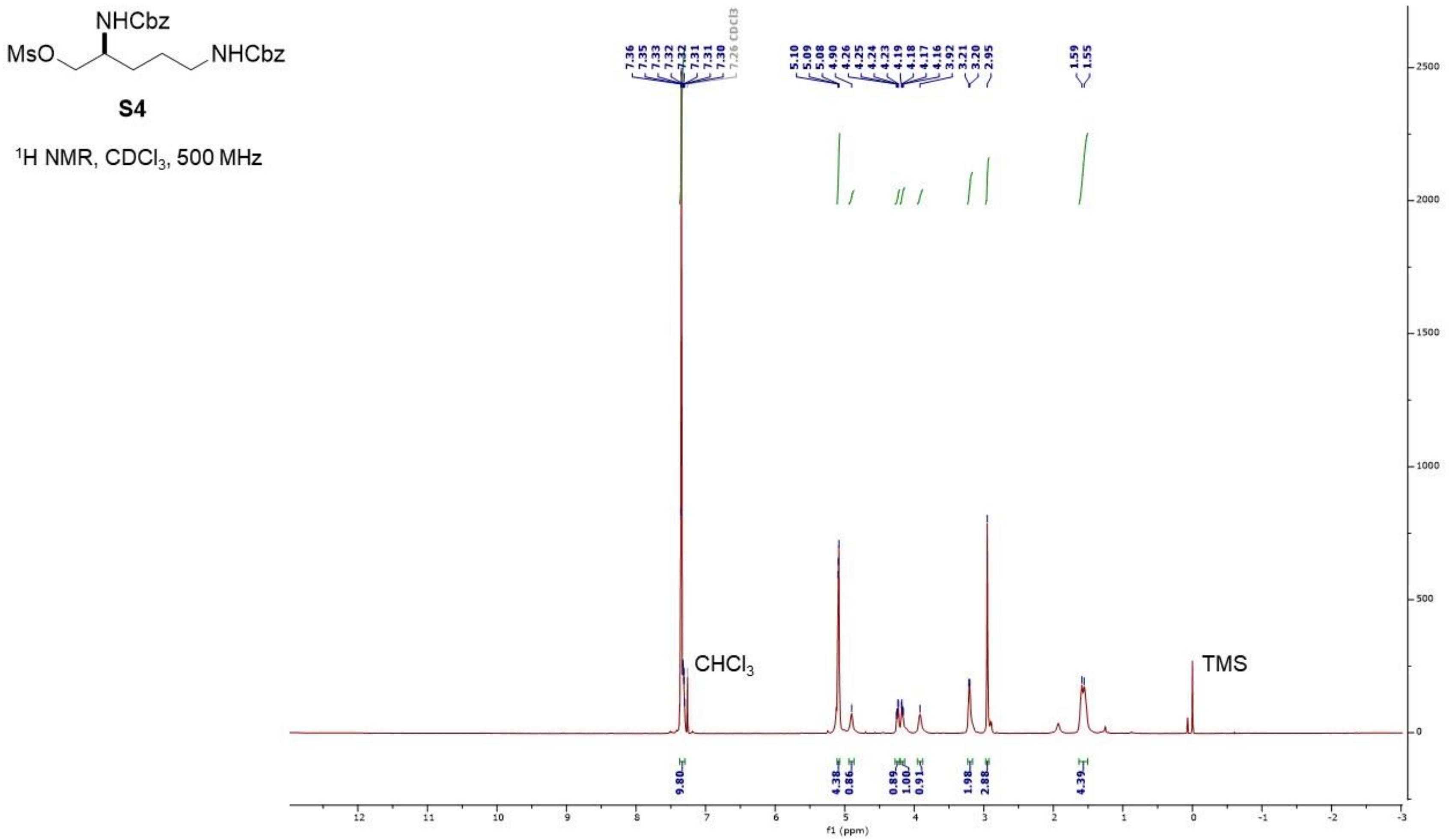

NHCbz
MsO
NHCbz
S4
^{1}H NMR, CDCl$_3$, 500 MHz
CHCl$_3$
TMS
f1 (ppm)

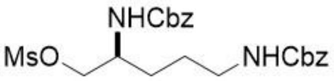

S4

^{13}C NMR, $CDCl_3$, 100 MHz

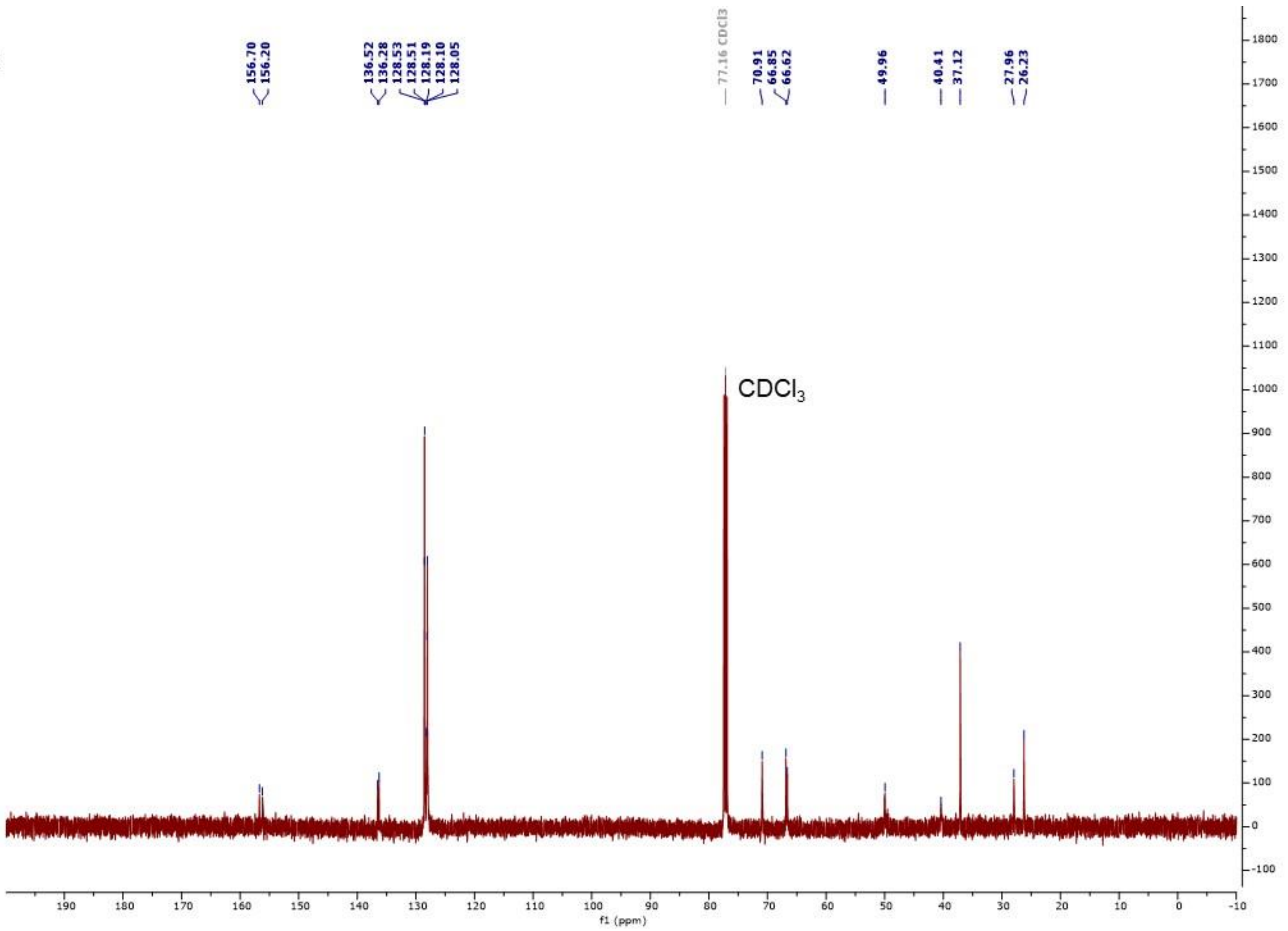

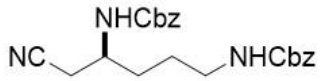

S5

^1H NMR, $CDCl_3$, 500 MHz

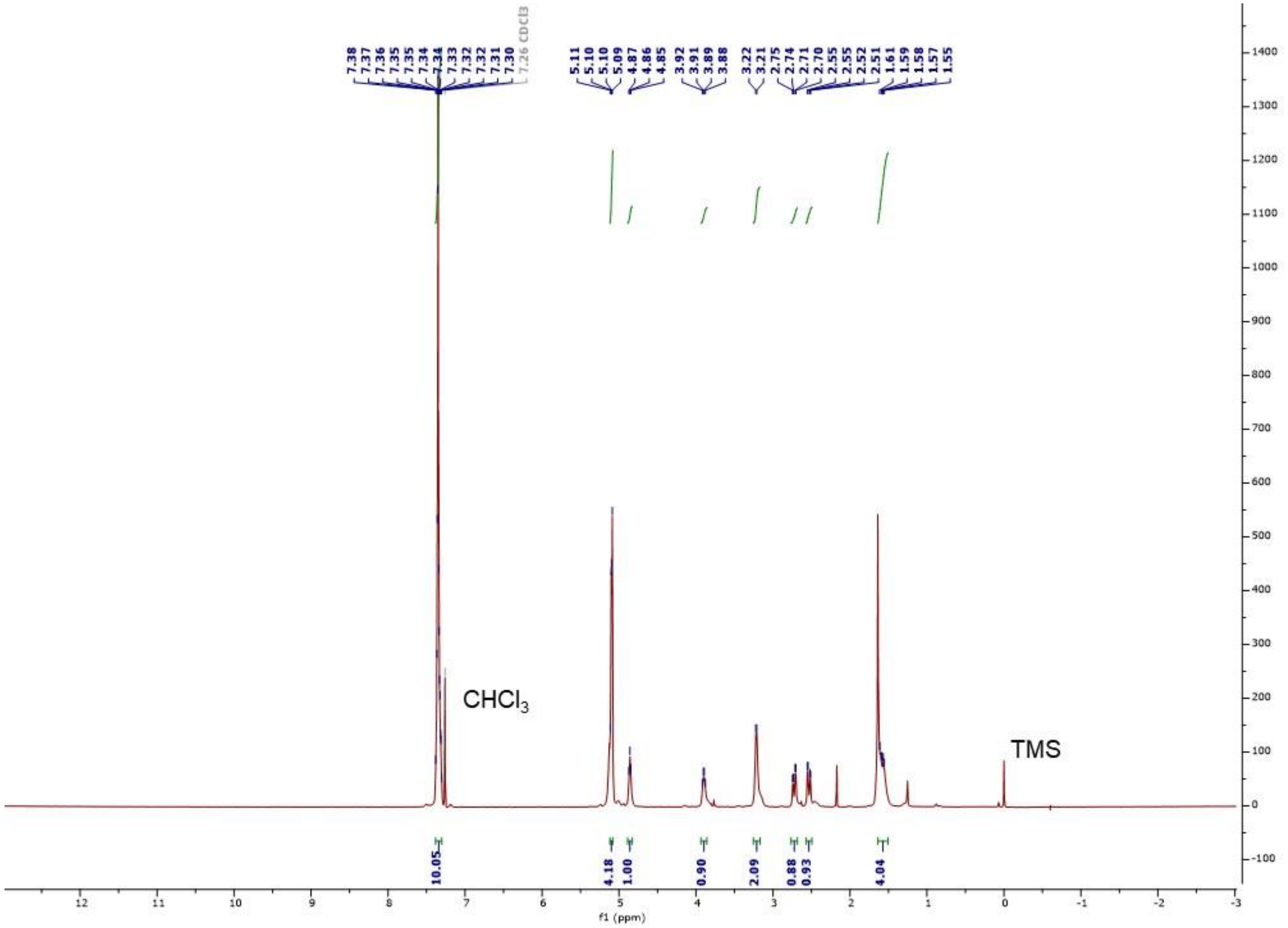

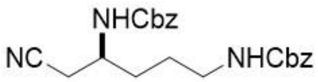

S5

^{13}C NMR, $CDCl_3$, 125 MHz

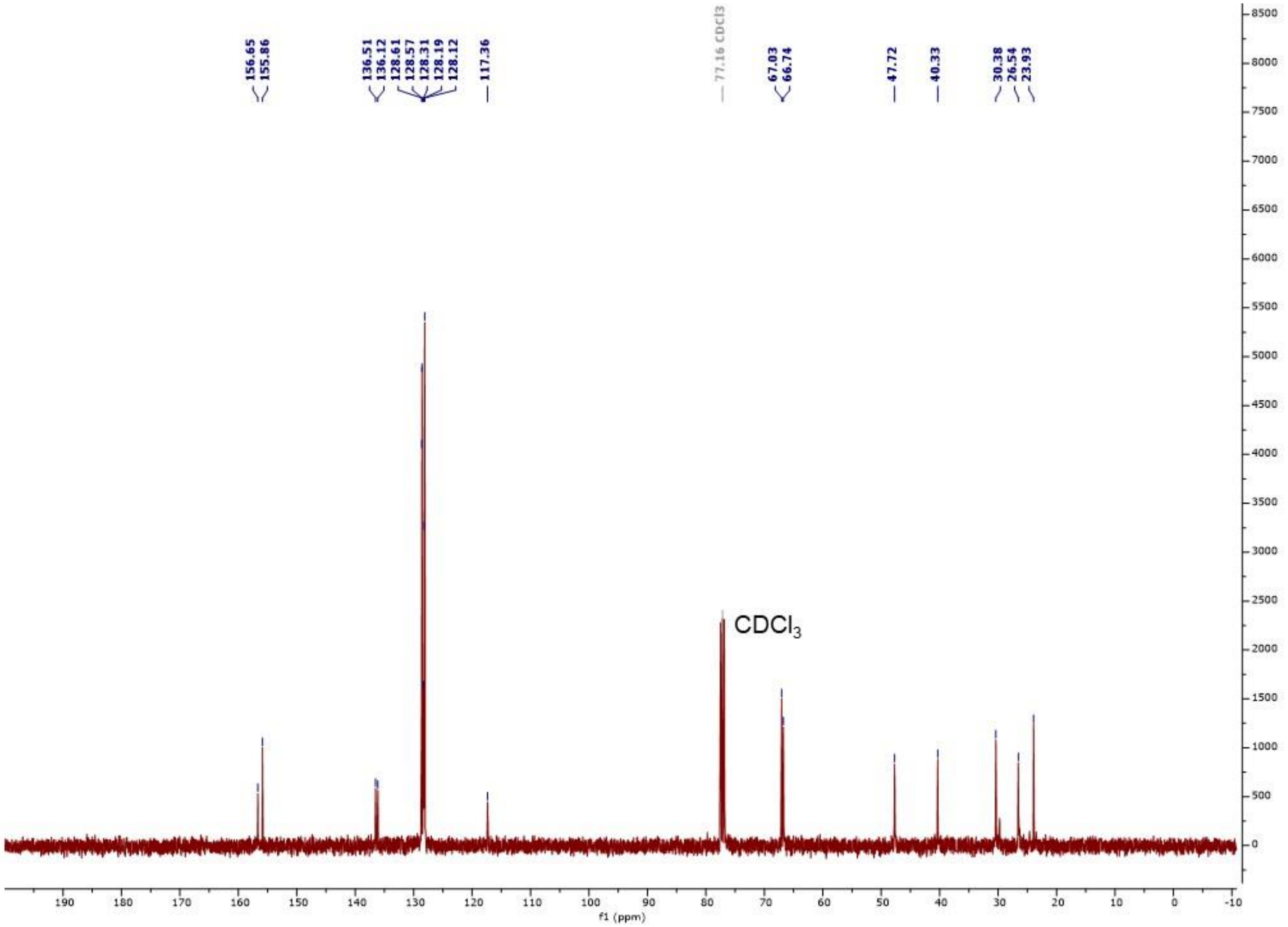

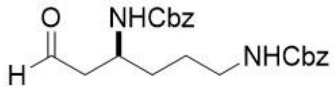

S6

^{1}H NMR, $CDCl_3$, 500 MHz

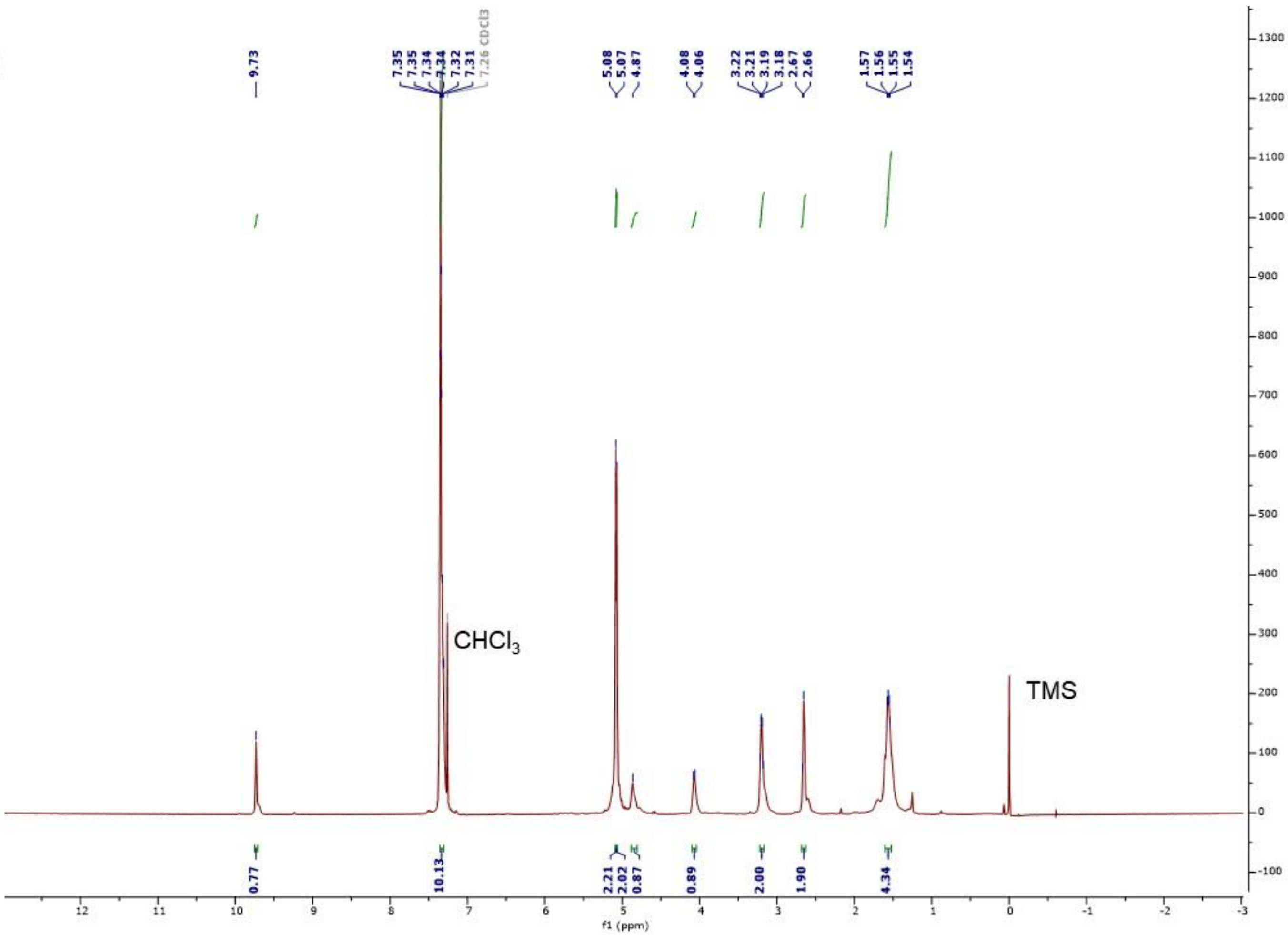

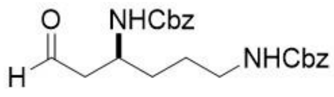

S6

^{13}C NMR, $CDCl_3$, 100 MHz

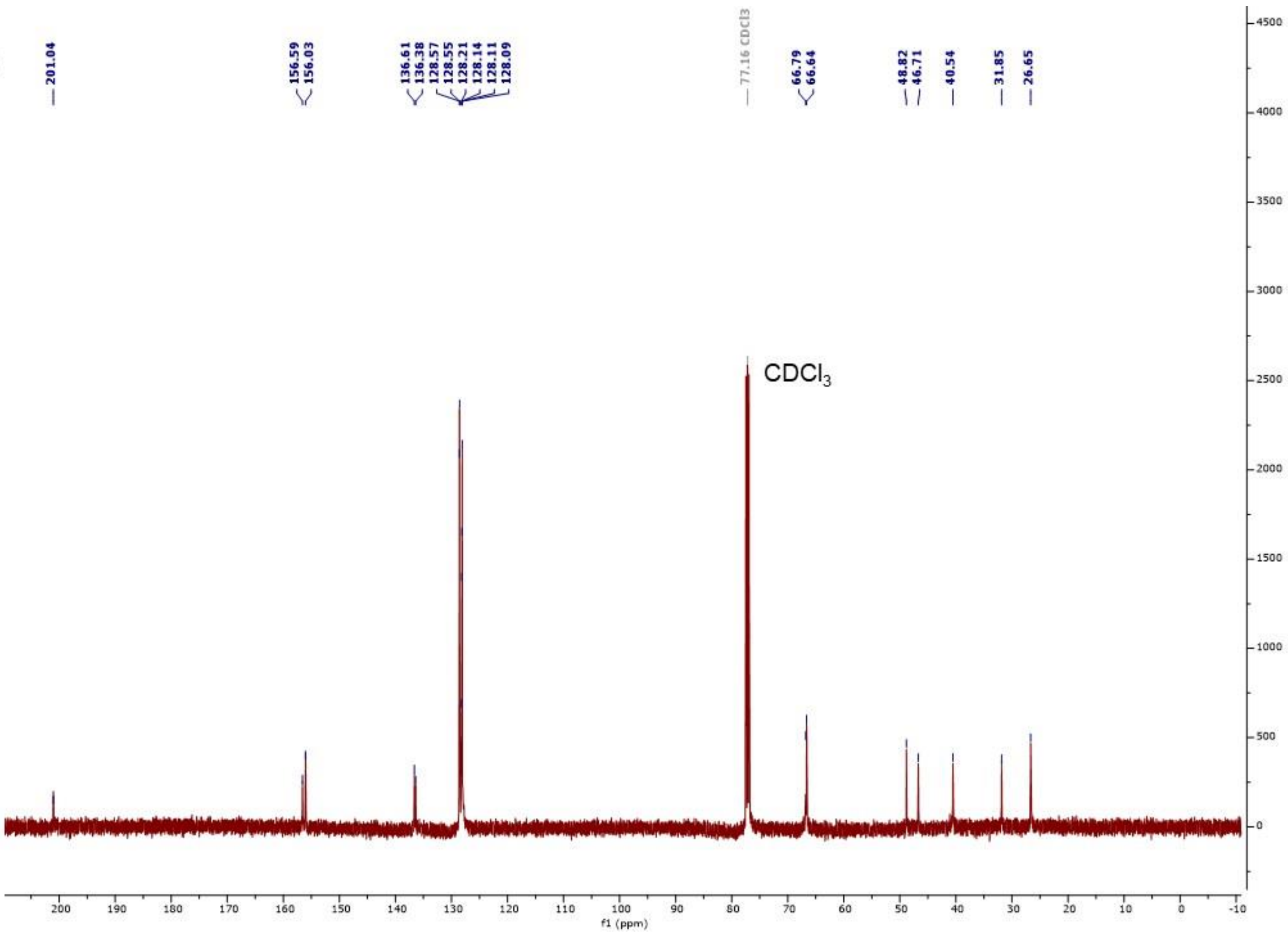

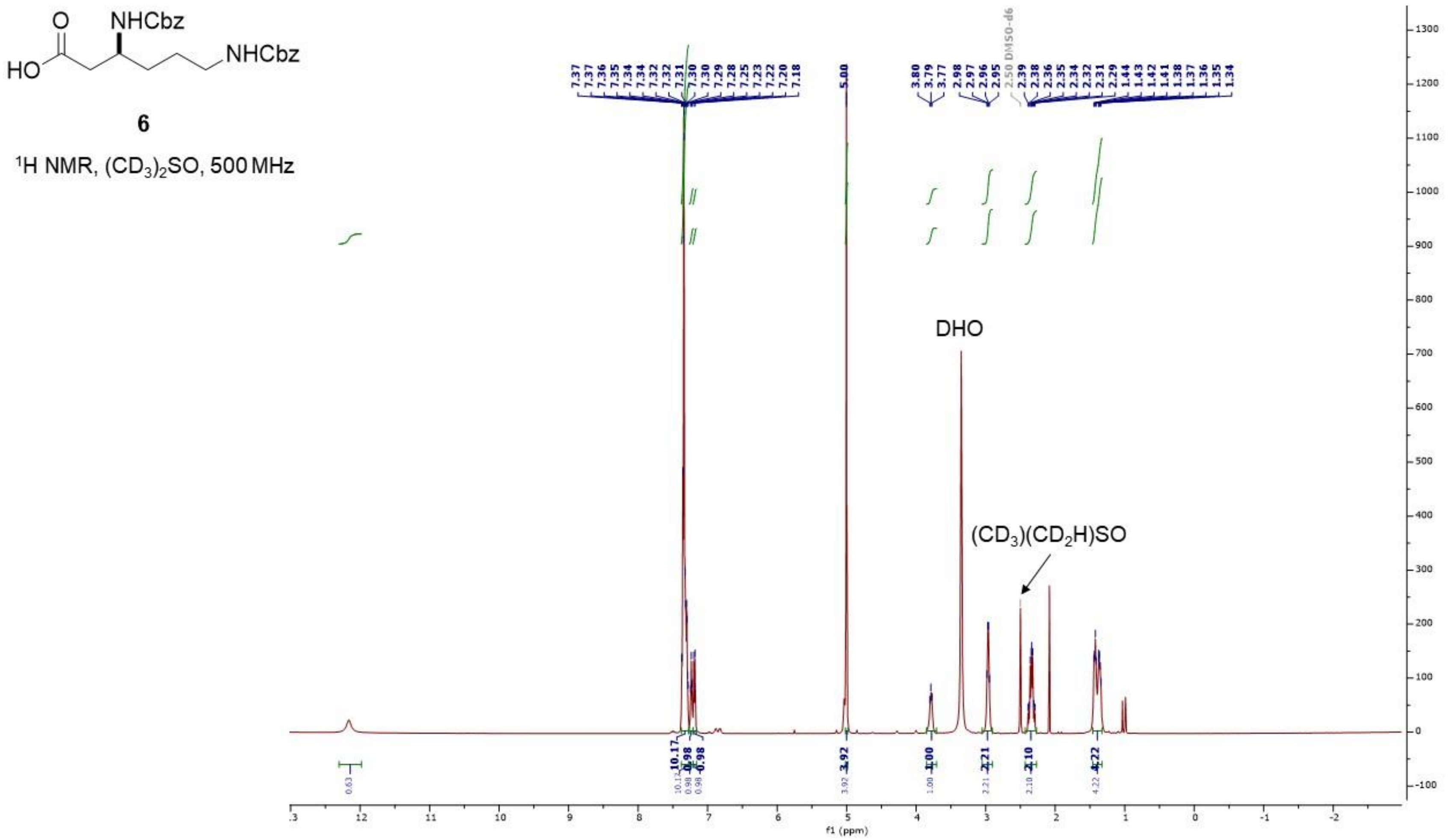
NHCbz
HO
NHCbz
O
6
^{1}H NMR, $(CD_3)_2SO$, 500 MHz
DHO
$(CD_3)(CD_2H)SO$
f1 (ppm)

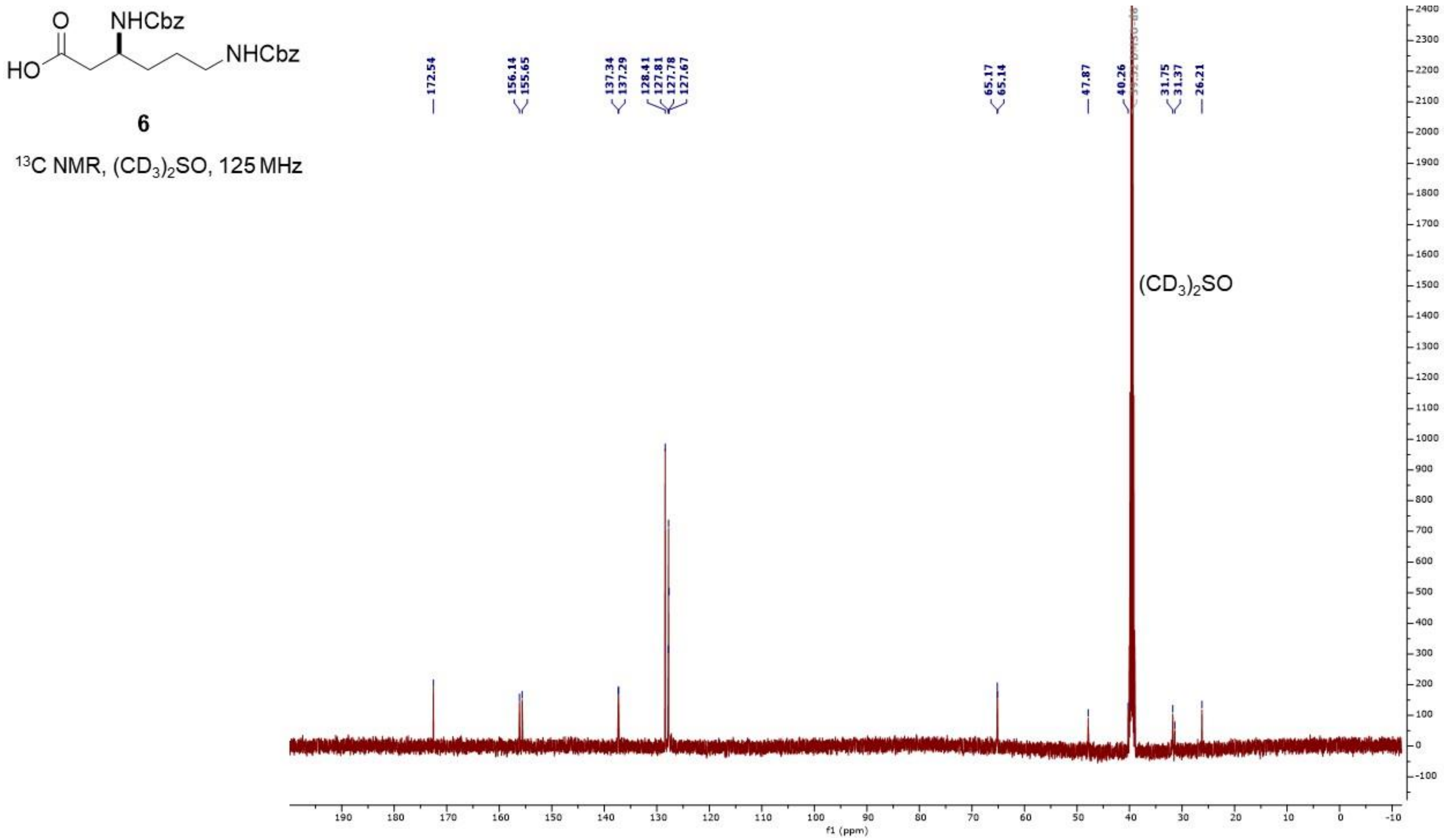

NHCbz
HO
NHCbz
6
^{13}C NMR, $(CD_3)_2SO$, 125 MHz
172.54
156.14
155.65
137.34
137.29
128.41
127.81
127.78
127.67
65.17
65.14
47.87
40.26
31.75
31.37
26.21
$(CD_3)_2SO$
f1 (ppm)

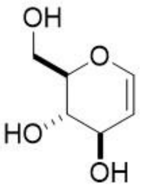

S7

^{1}H NMR, CD_3OD, 500 MHz

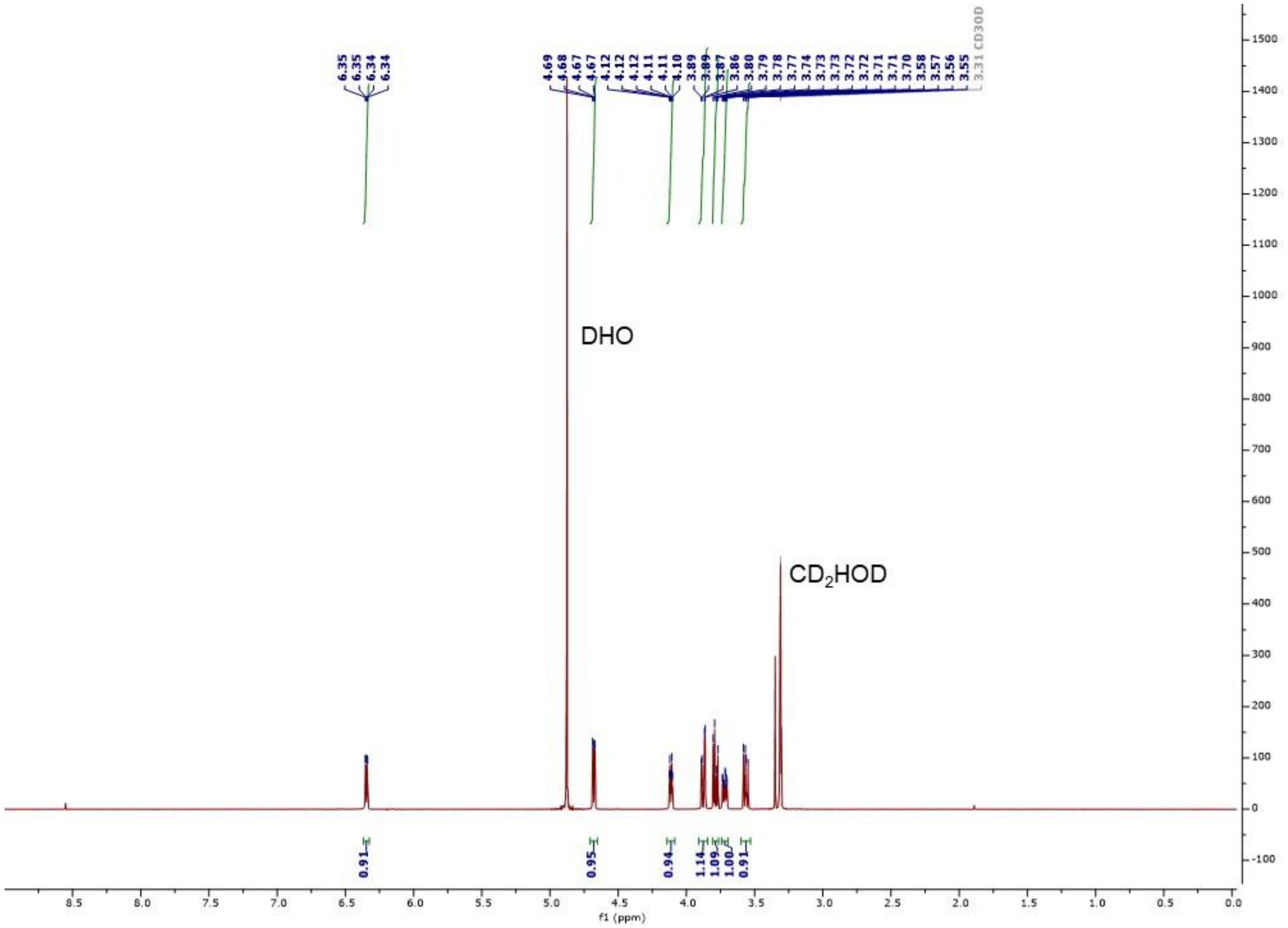

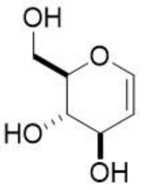

S7

^{13}C NMR, CD_3OD, 100 MHz

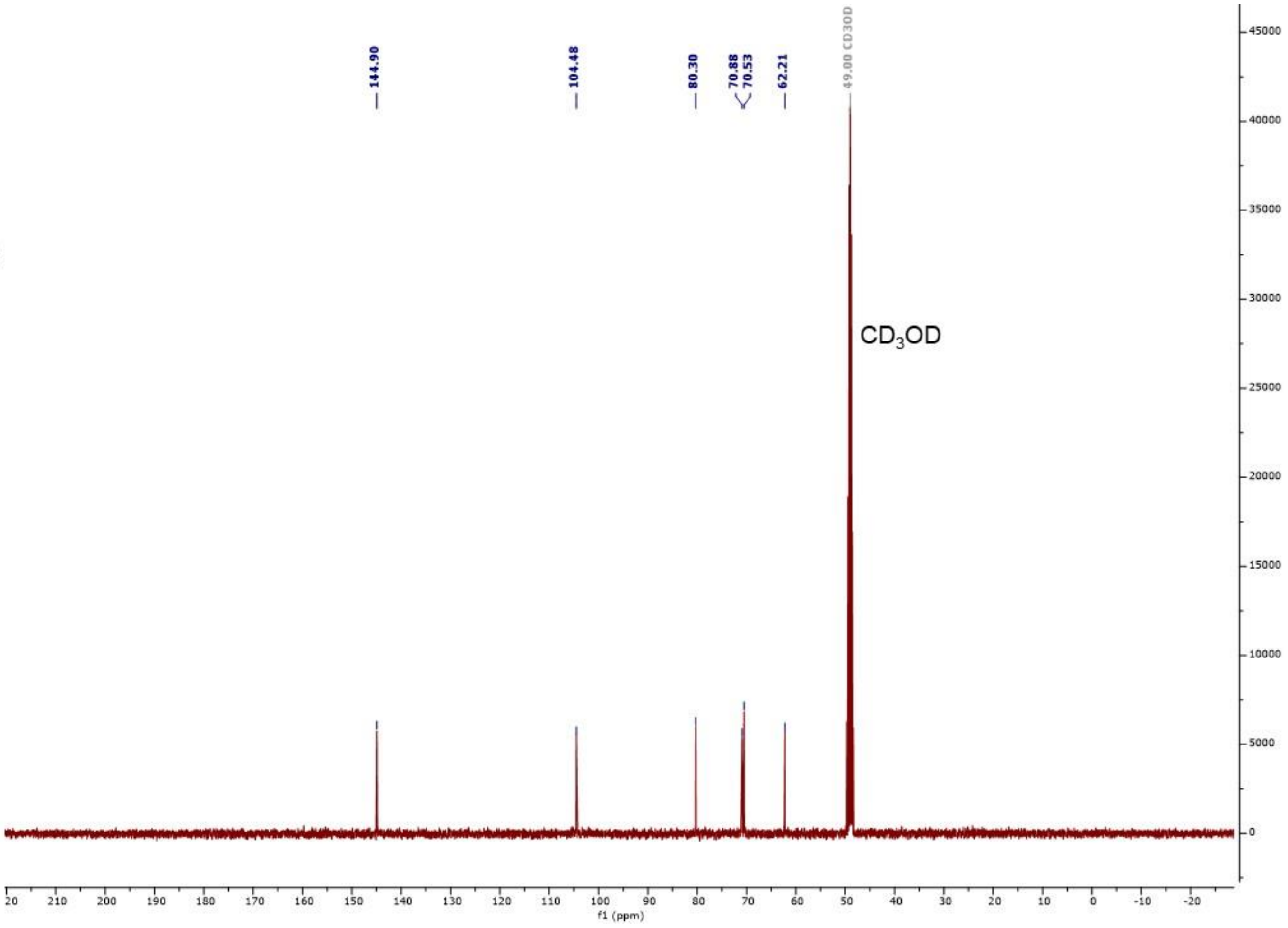

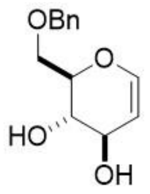

9

^1H NMR, $CDCl_3$, 500 MHz

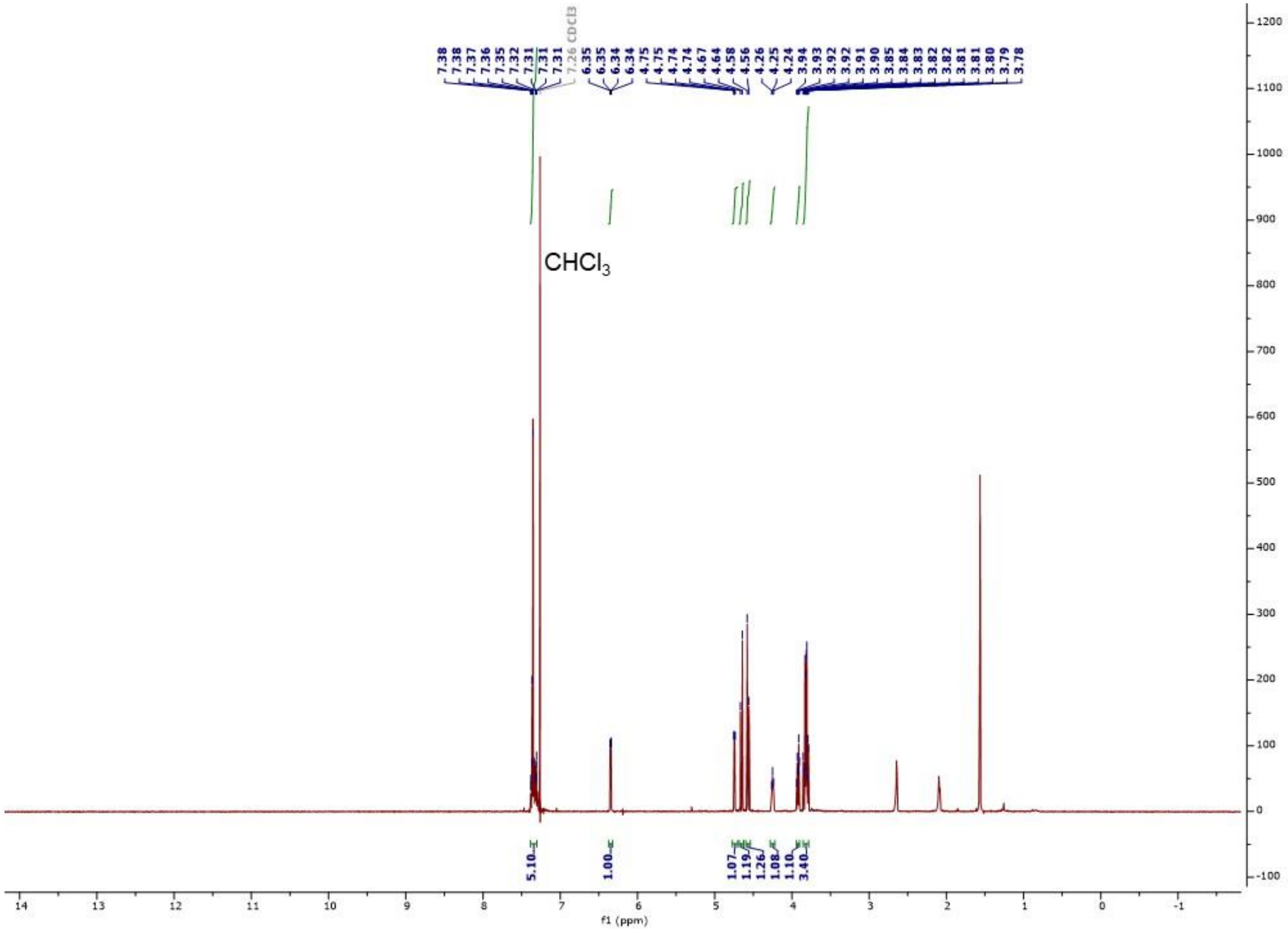

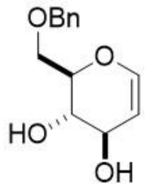

9

^{13}C NMR, $CDCl_3$, 100 MHz

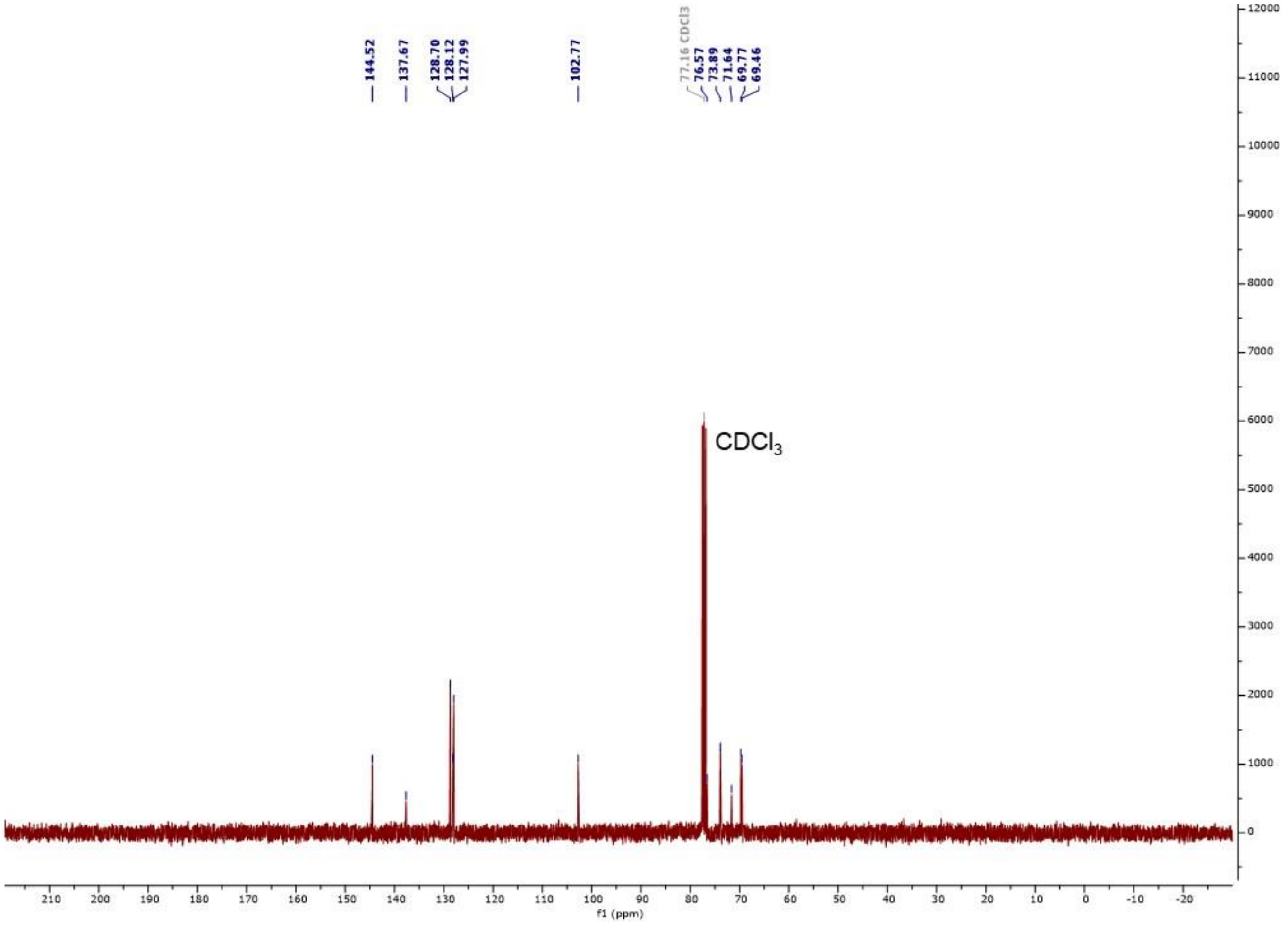

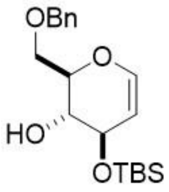

S8

^{1}H NMR, $CDCl_3$, 500 MHz

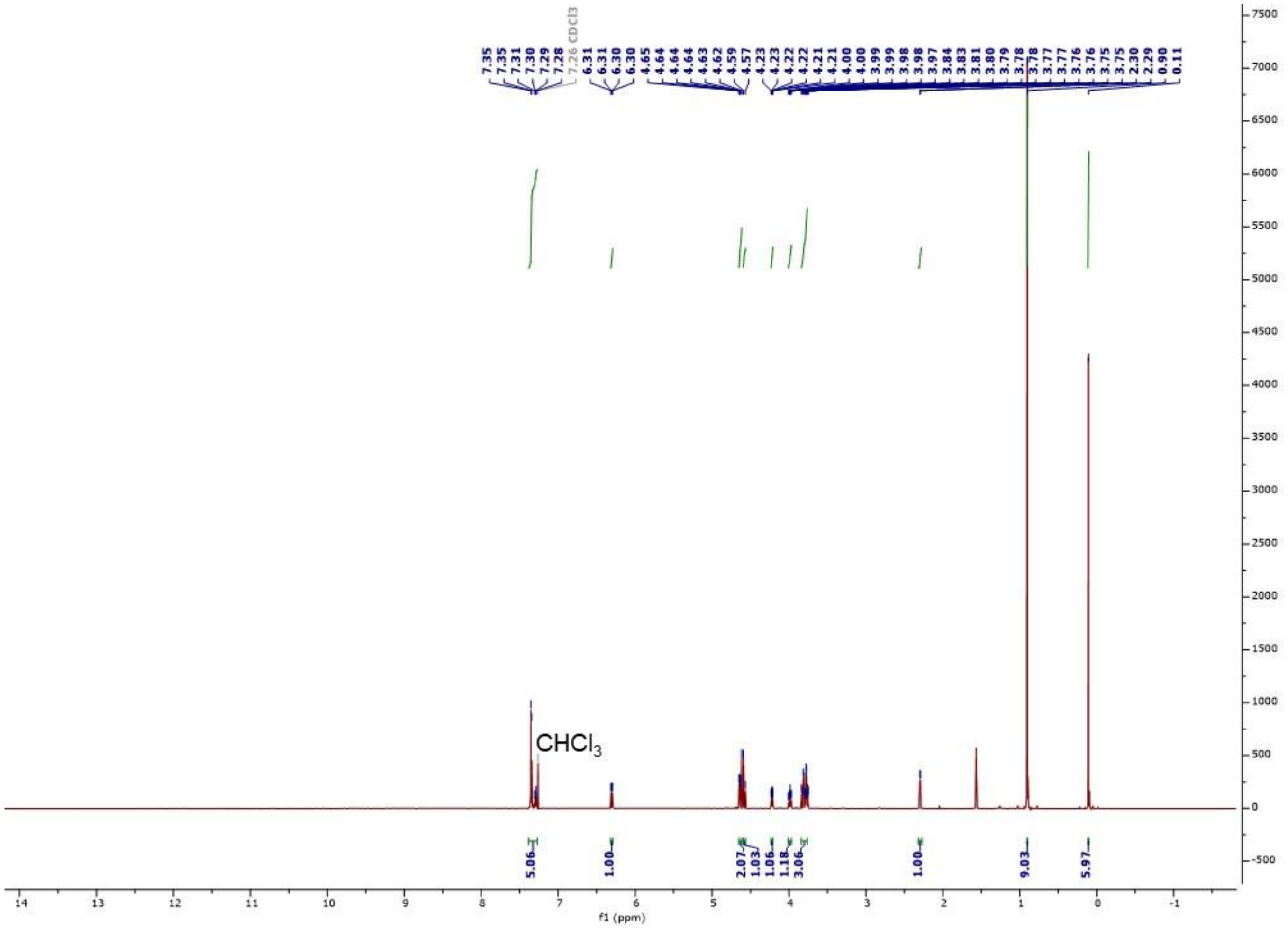

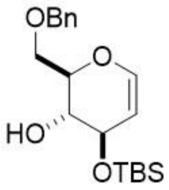

S8

^{13}C NMR, $CDCl_3$, 100 MHz

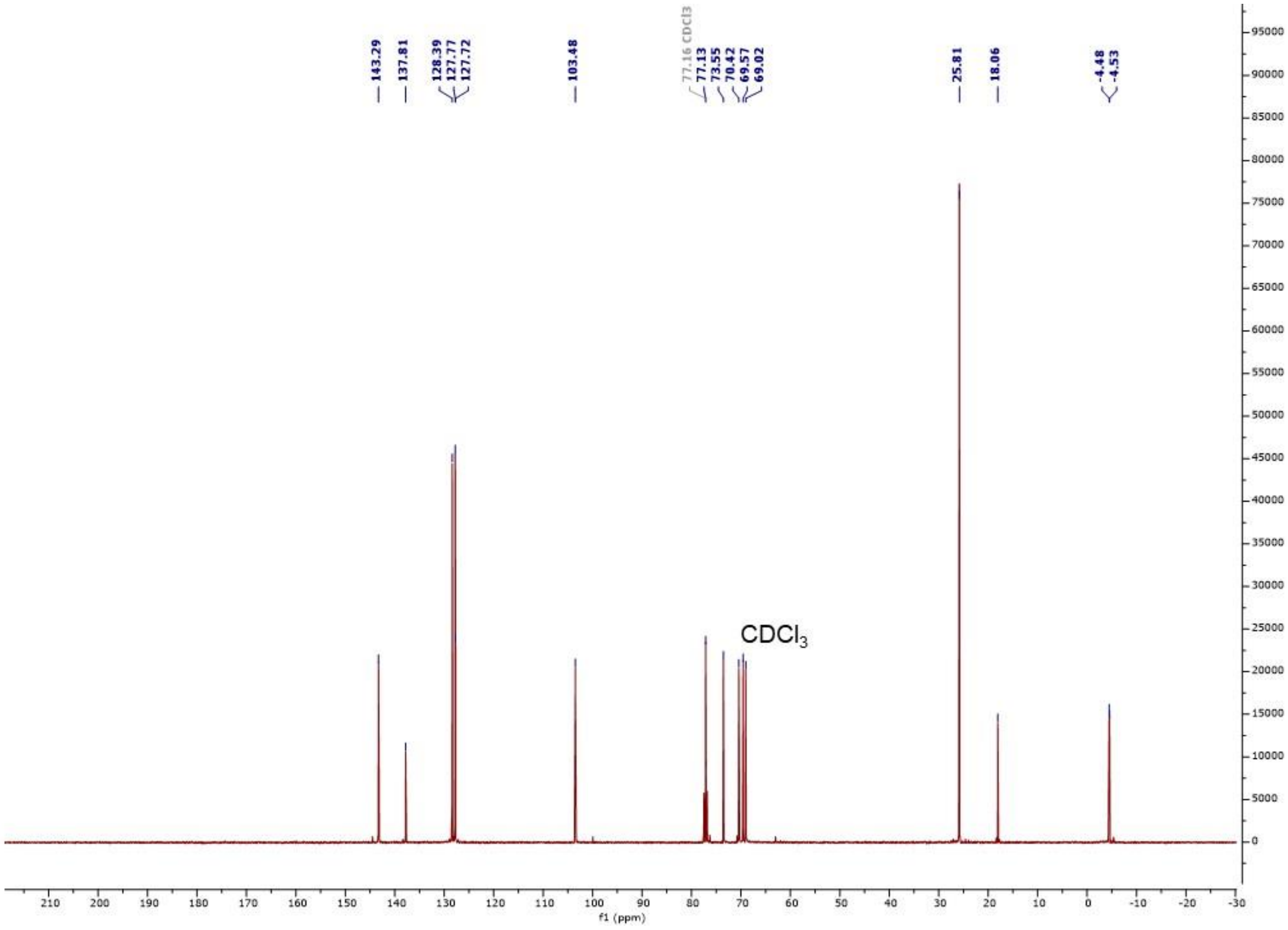

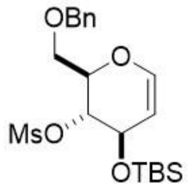

17

^{1}H NMR, $CDCl_3$, 500 MHz

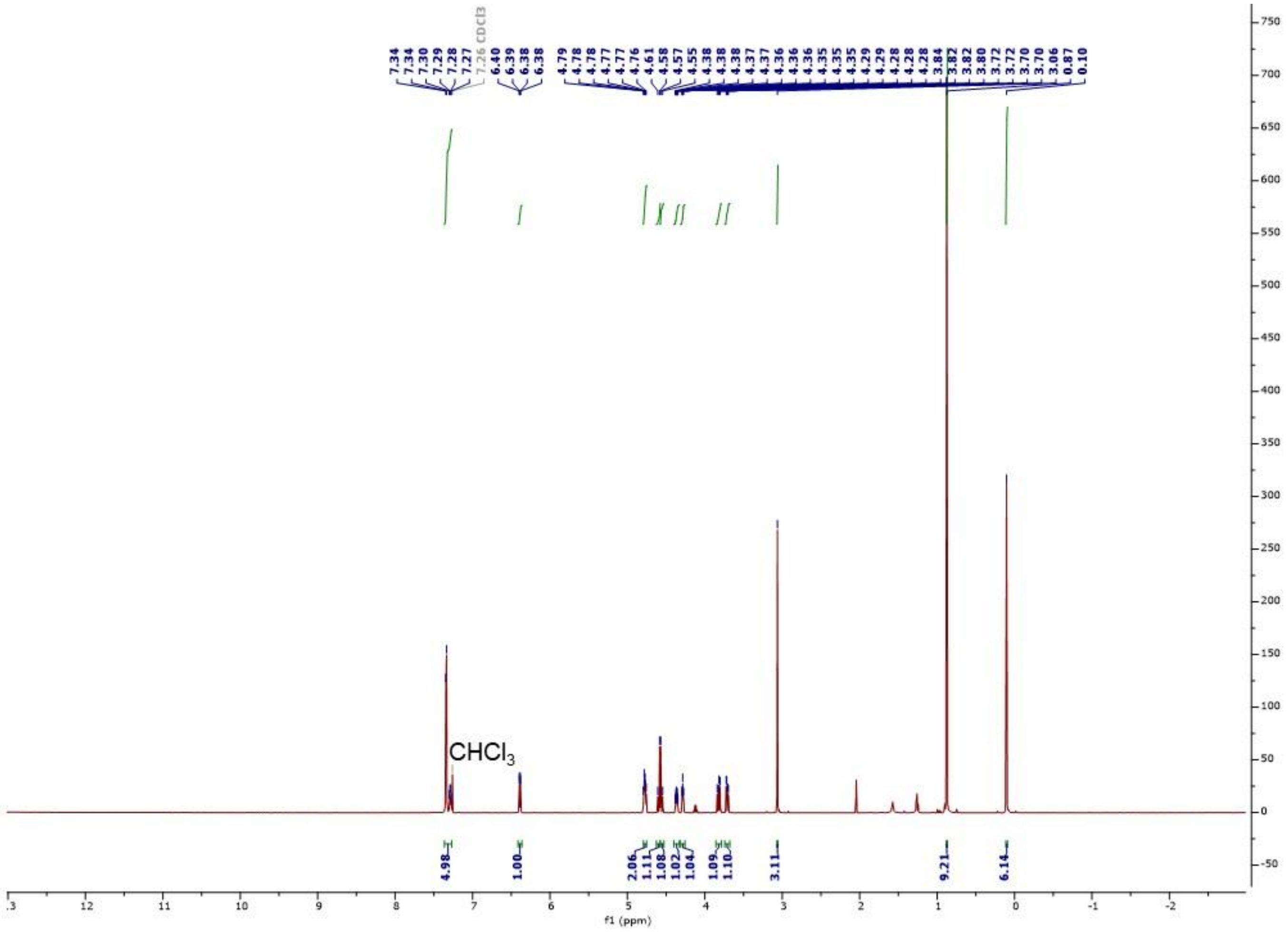

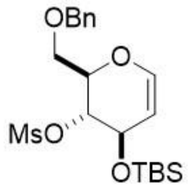

17

^{13}C NMR, $CDCl_3$, 100 MHz

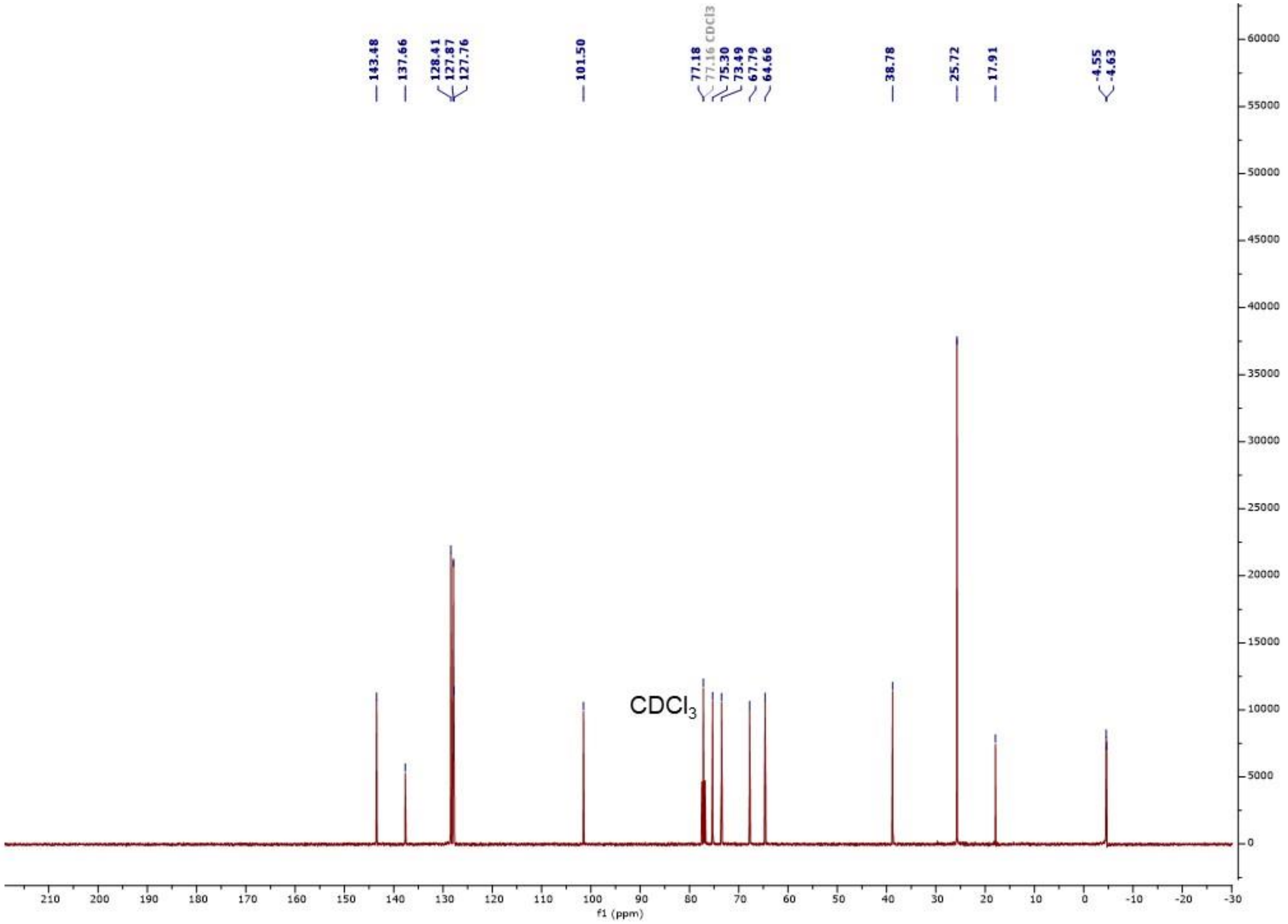

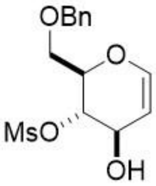

18

^1H NMR, $CDCl_3$, 500 MHz

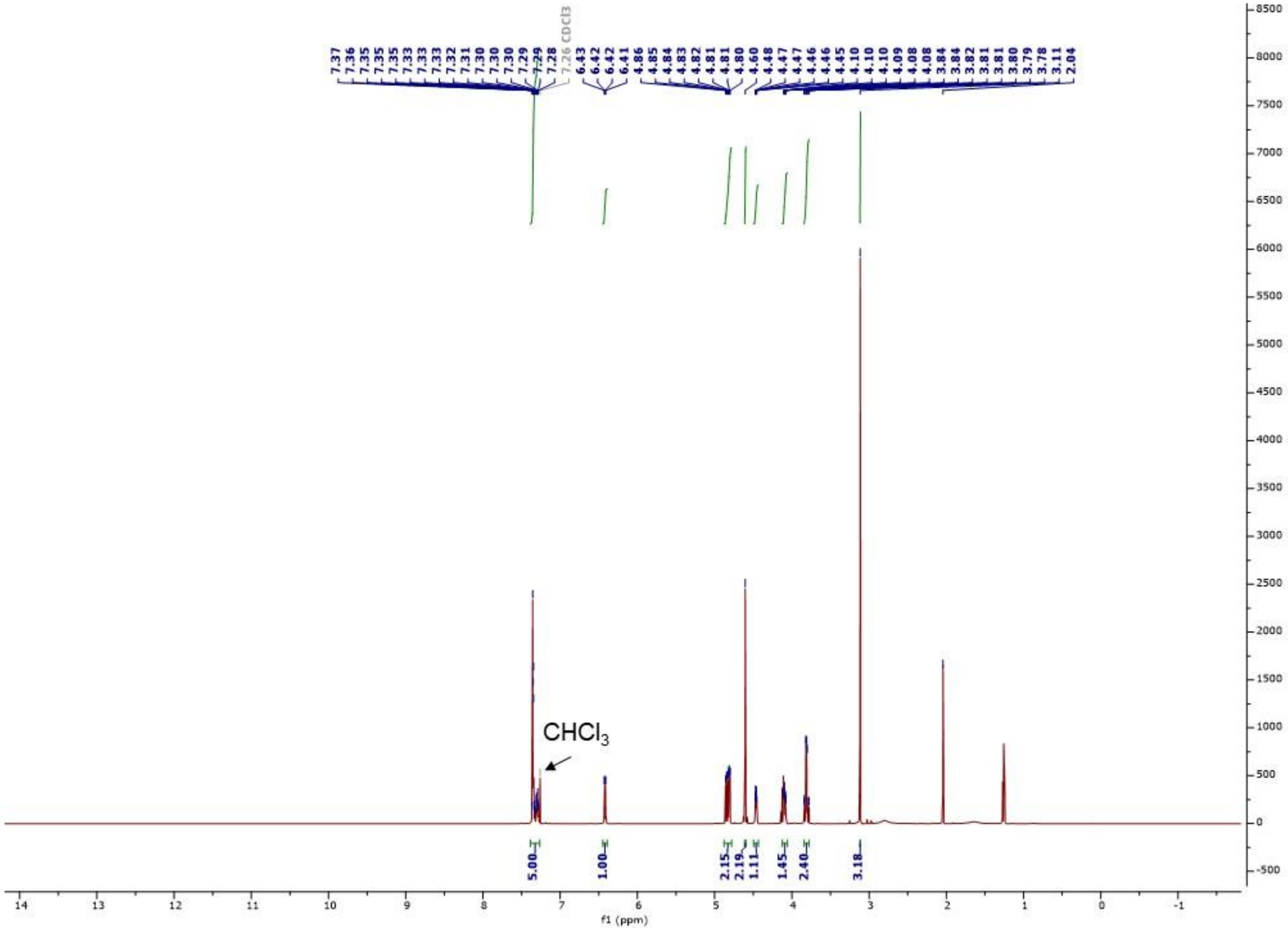

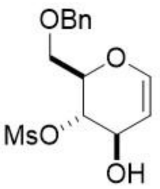

18

^{13}C NMR, $CDCl_3$, 100 MHz

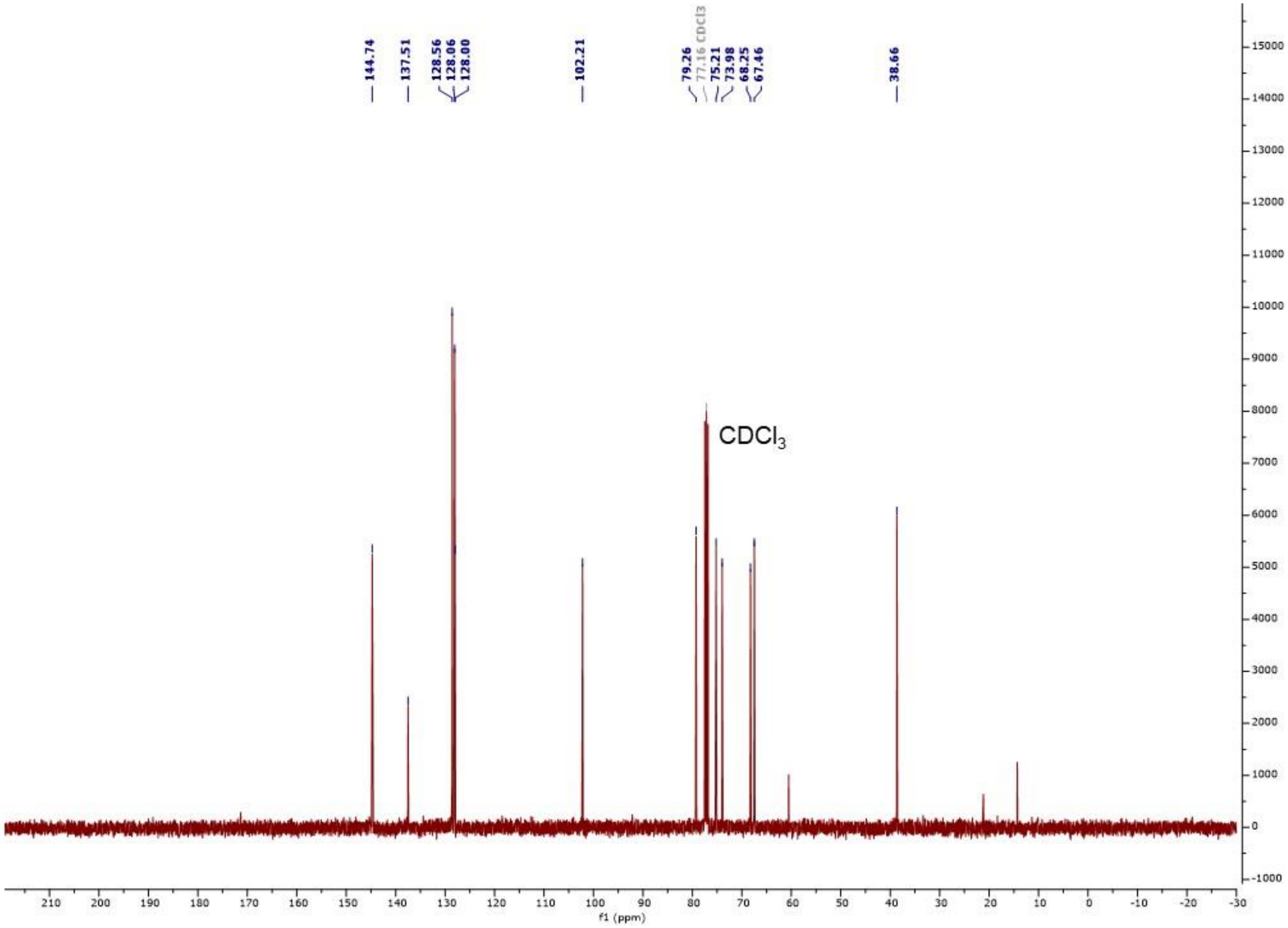

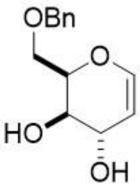

21

^{1}H NMR, $CDCl_3$, 500 MHz

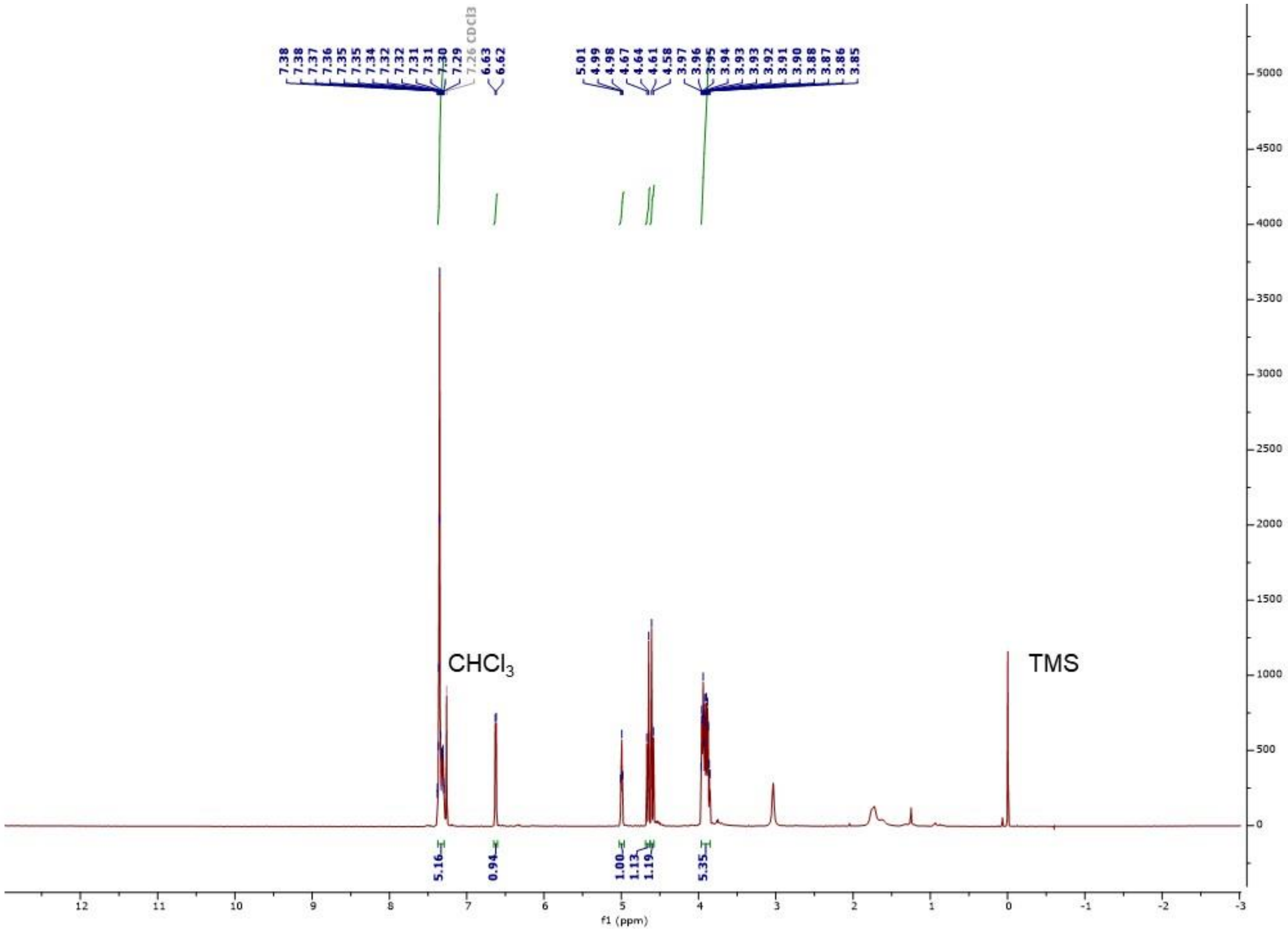

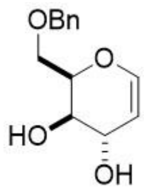

21

^{13}C NMR, $CDCl_3$, 125 MHz

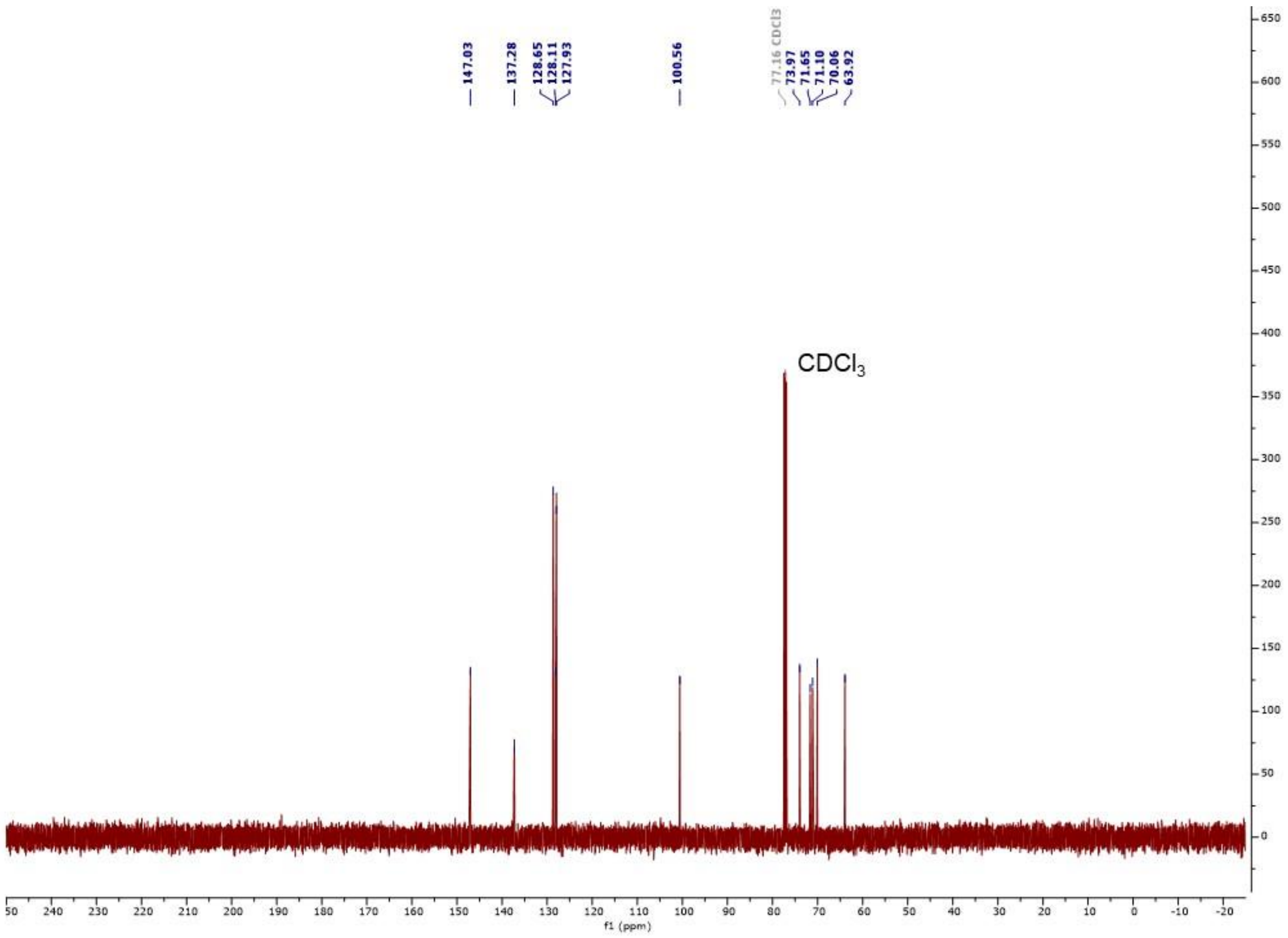

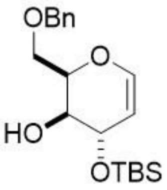

S9

^{1}H NMR, $CDCl_3$, 500 MHz

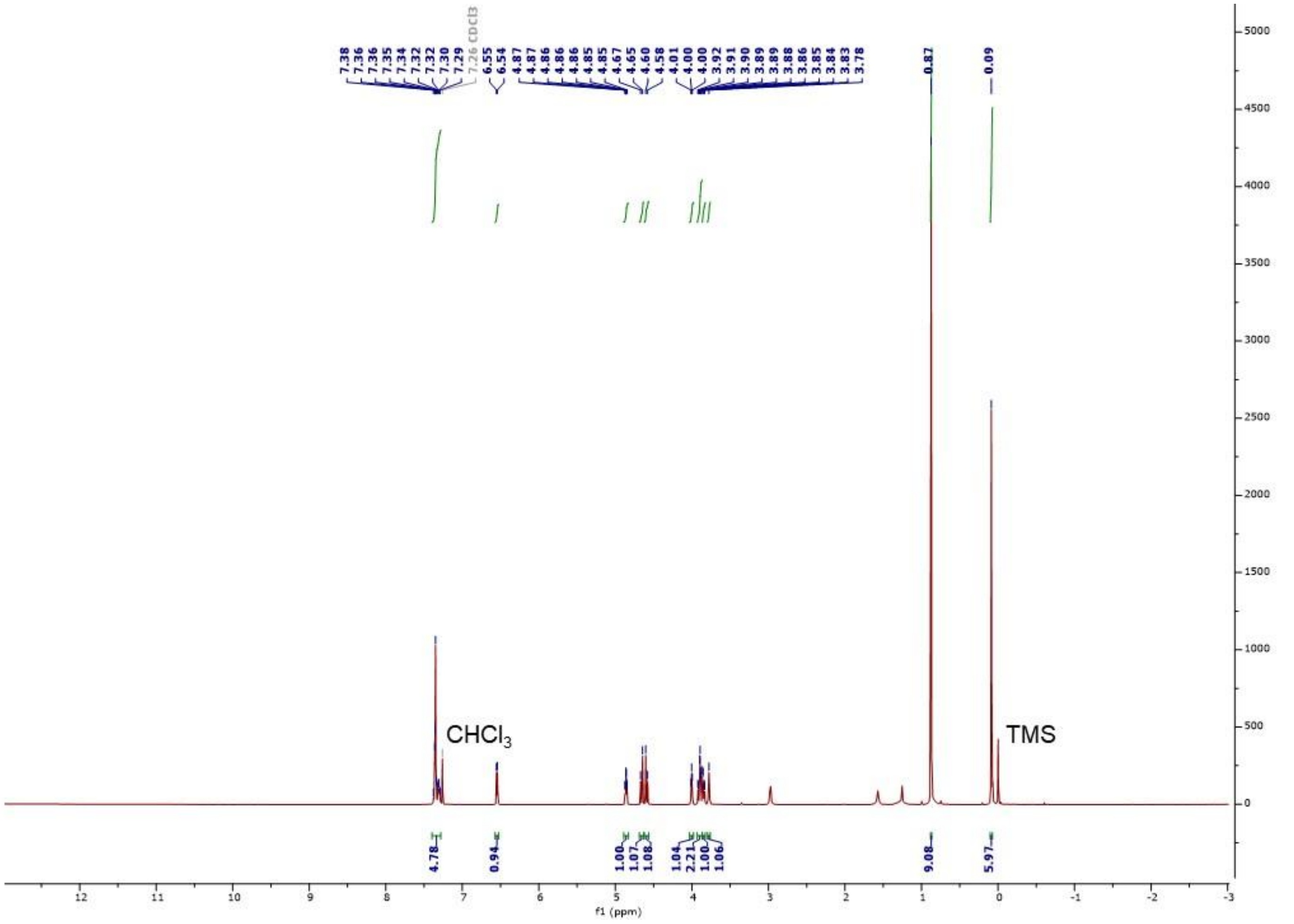

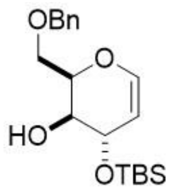

S9

^{13}C NMR, $CDCl_3$, 125 MHz

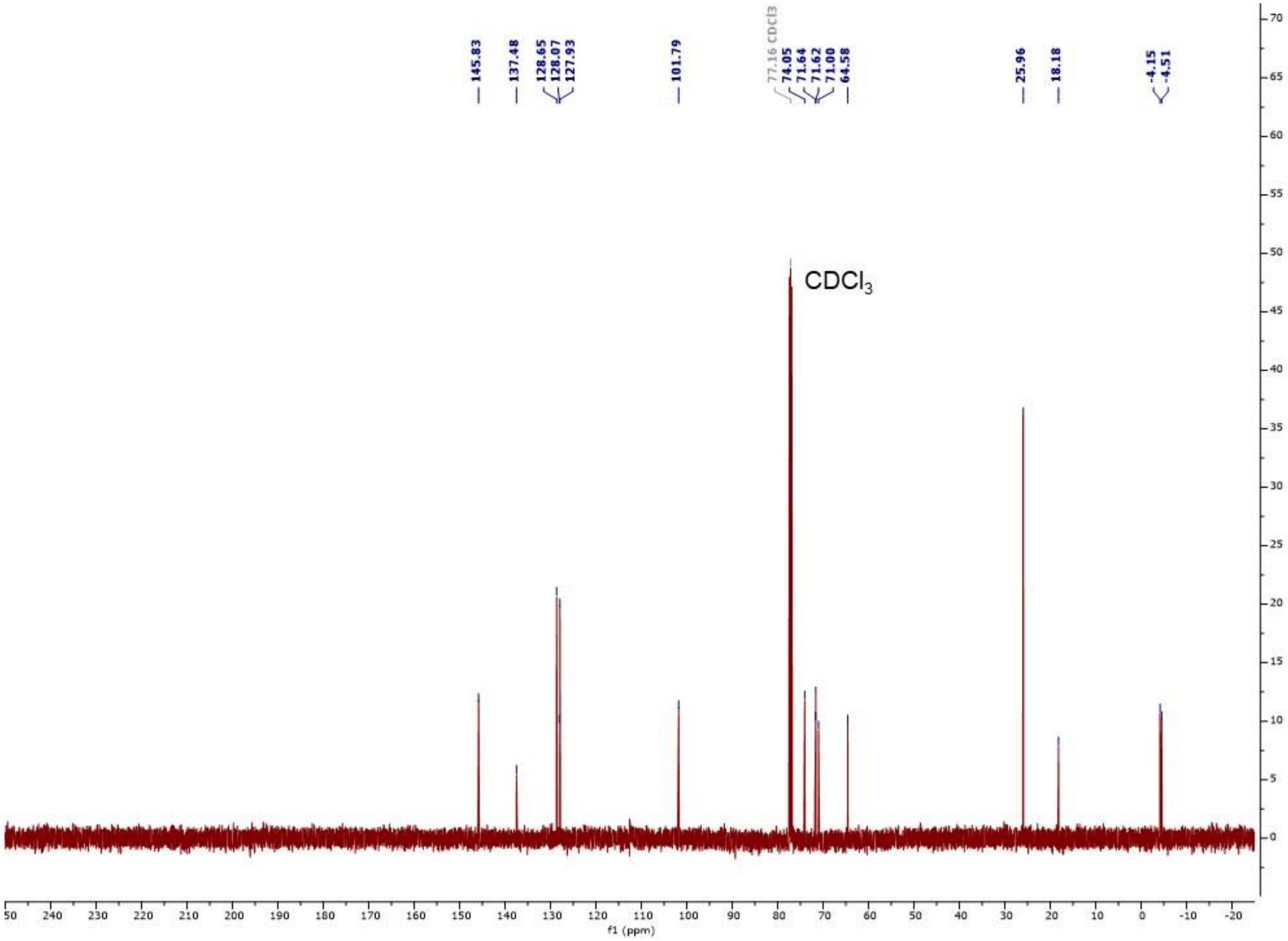

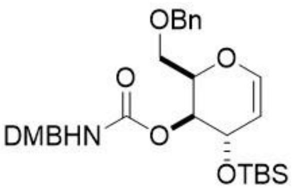

22

^{1}H NMR, $CDCl_3$, 500 MHz

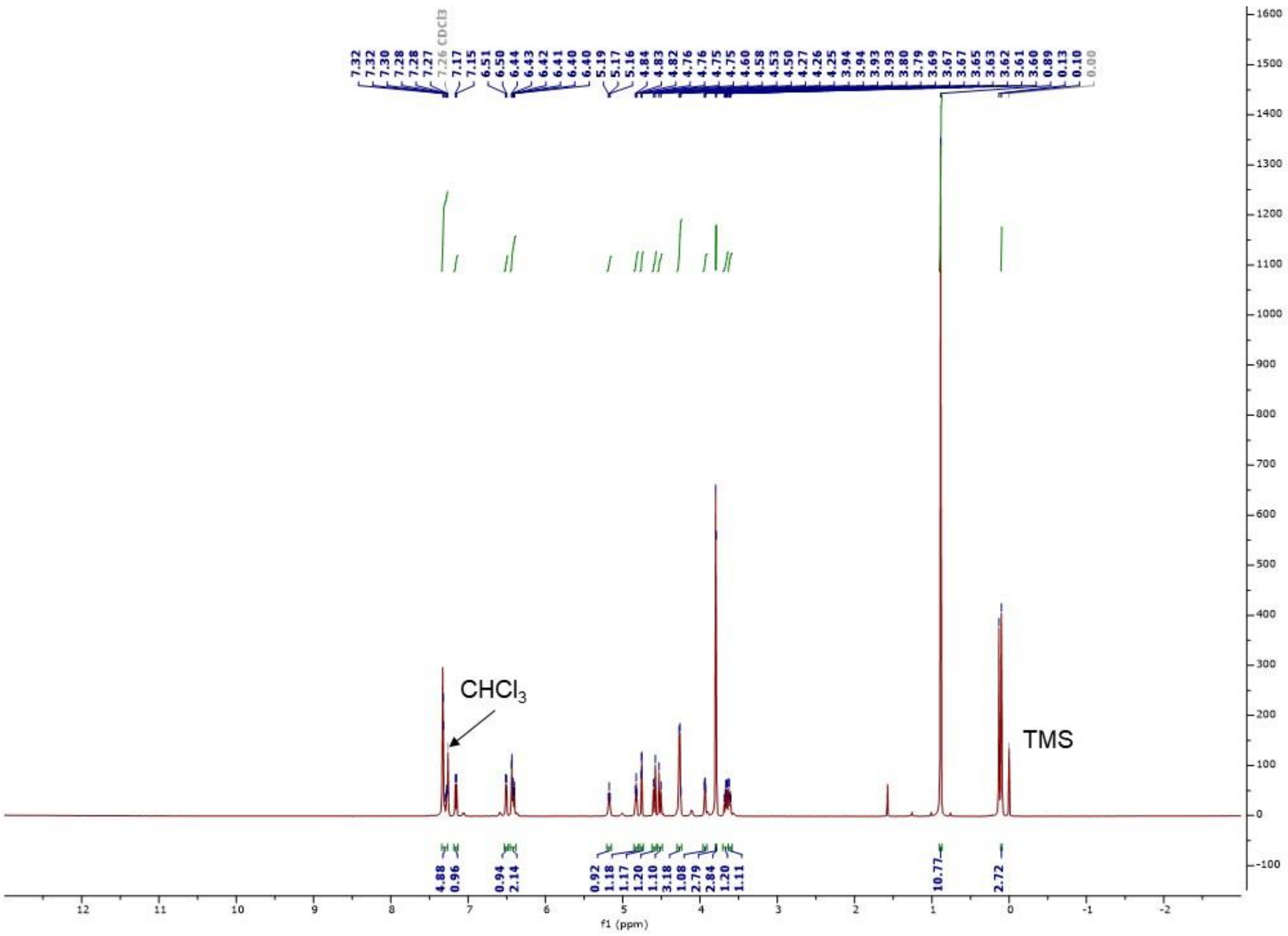

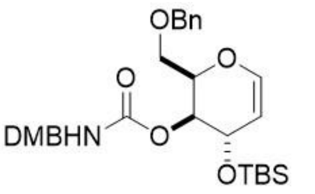

22

^{13}C NMR, $CDCl_3$, 125 MHz

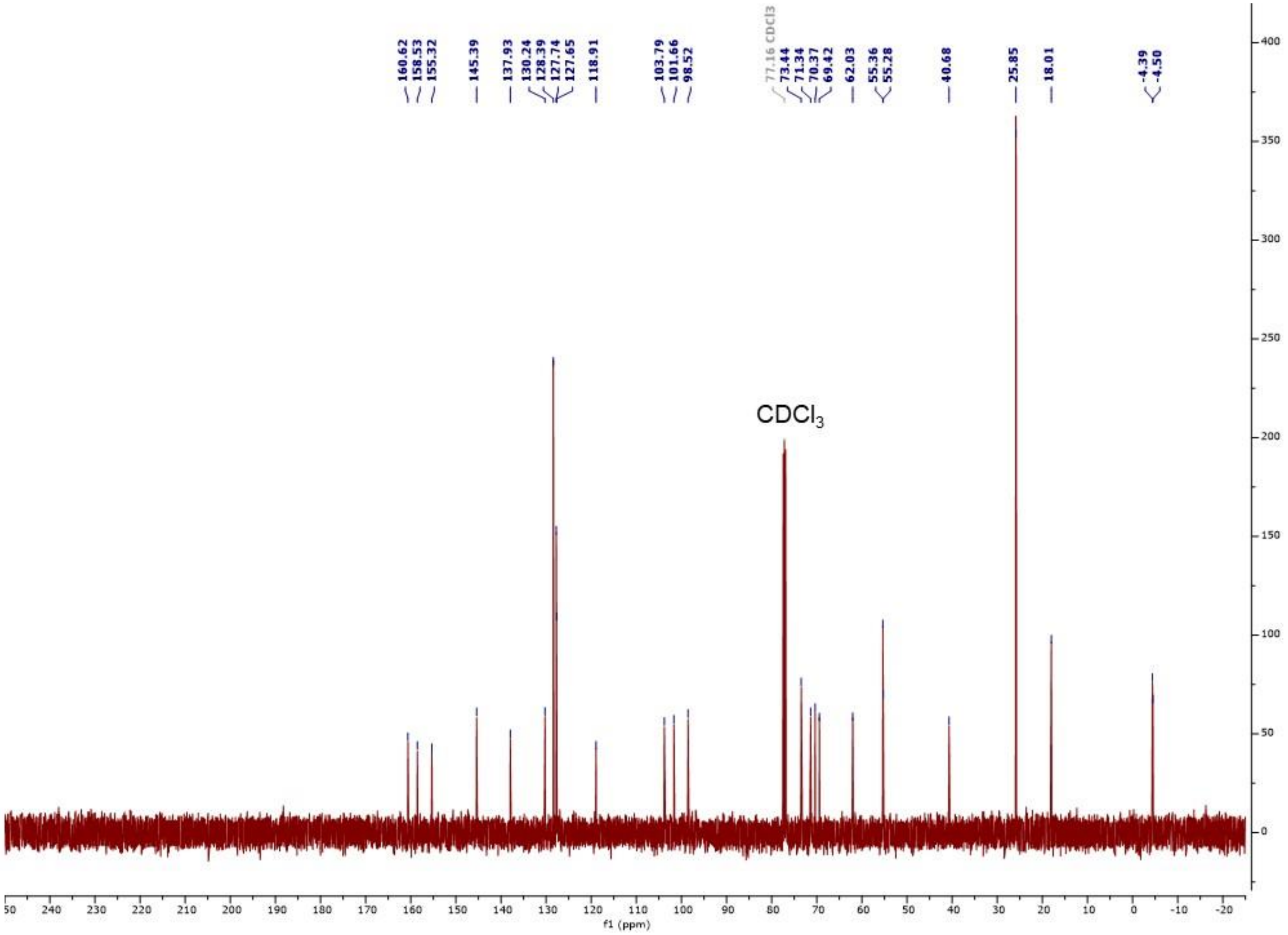

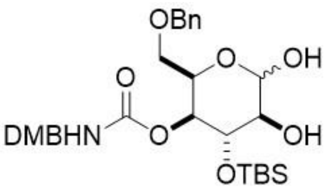

23

^1H NMR, $CDCl_3$, 500 MHz

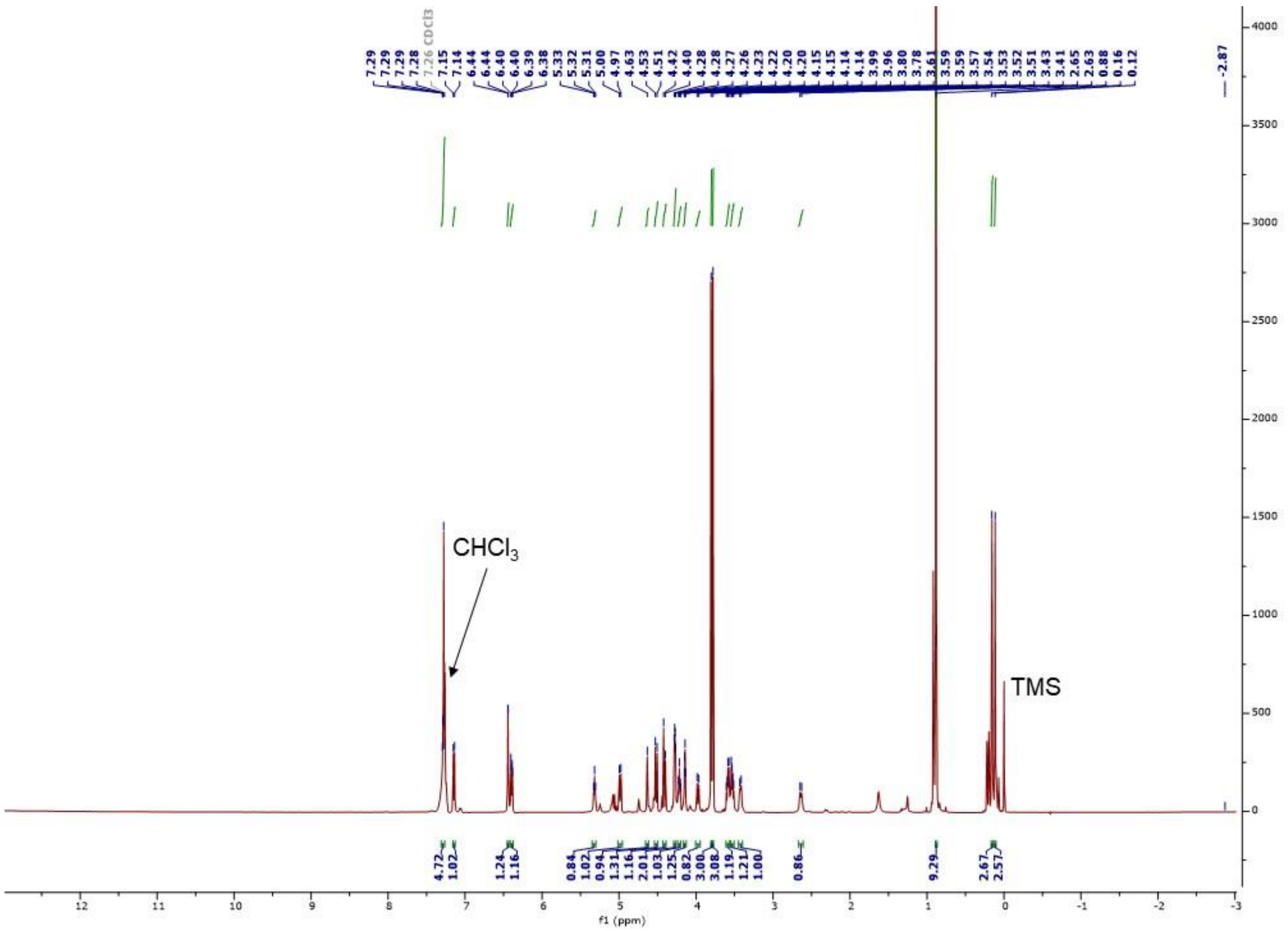

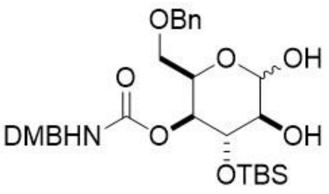

23

^{13}C NMR, $CDCl_3$, 125 MHz

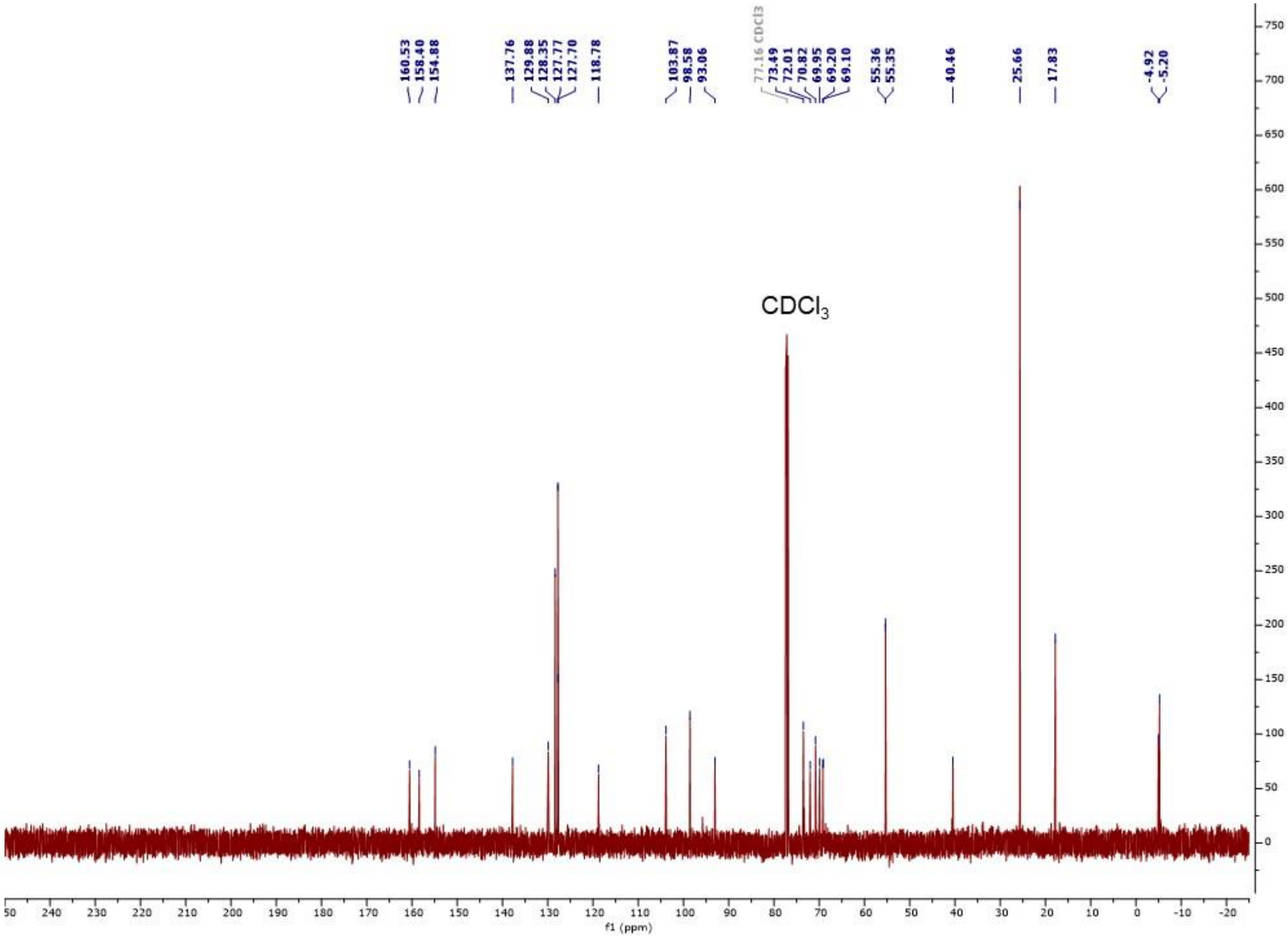

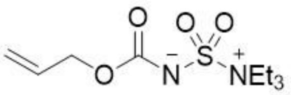

25

^{1}H NMR, CDCl3, 500 MHz

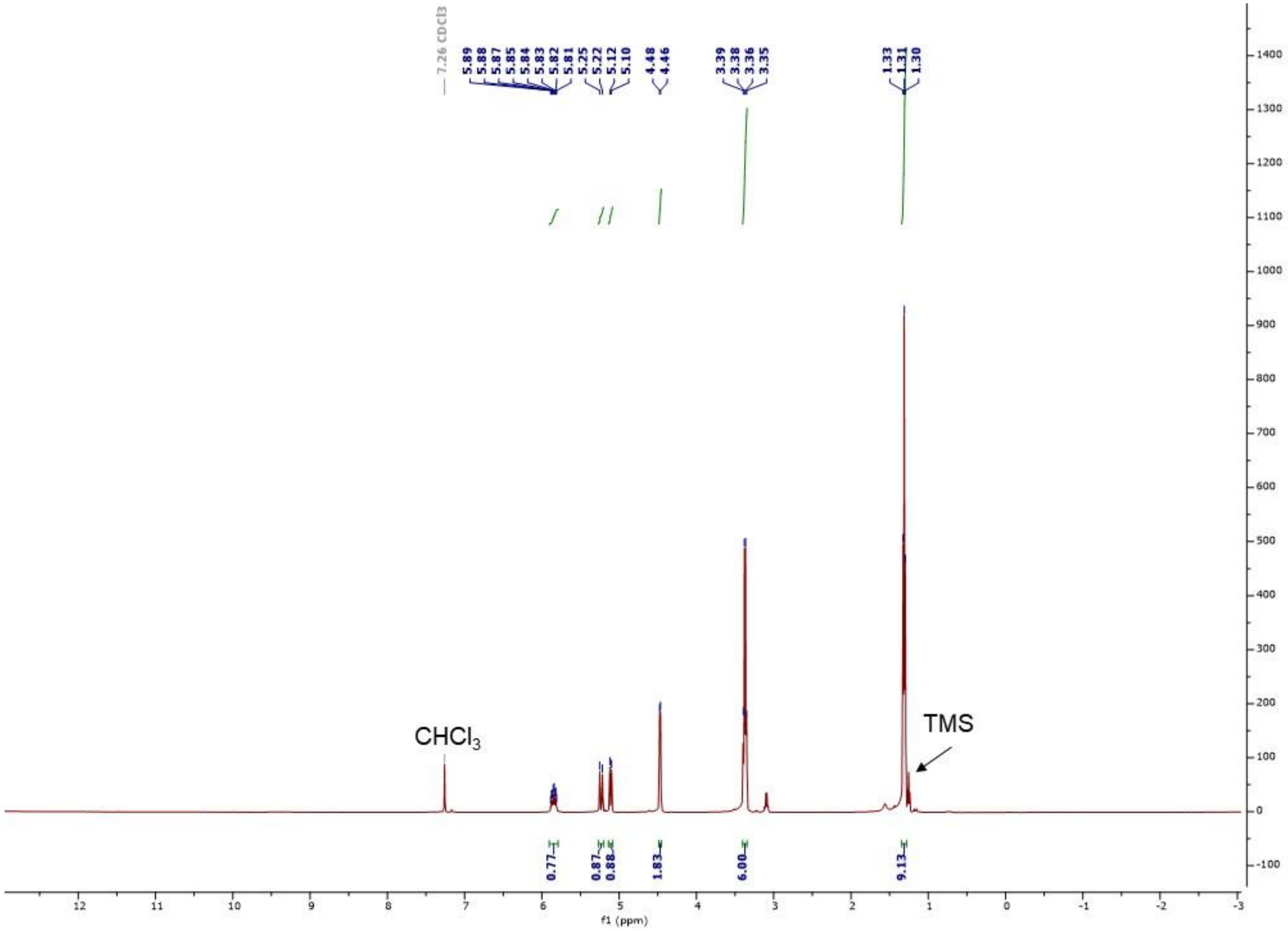

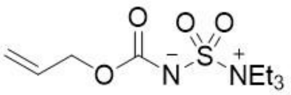

25

^{13}C NMR, $CDCl_3$, 100 MHz

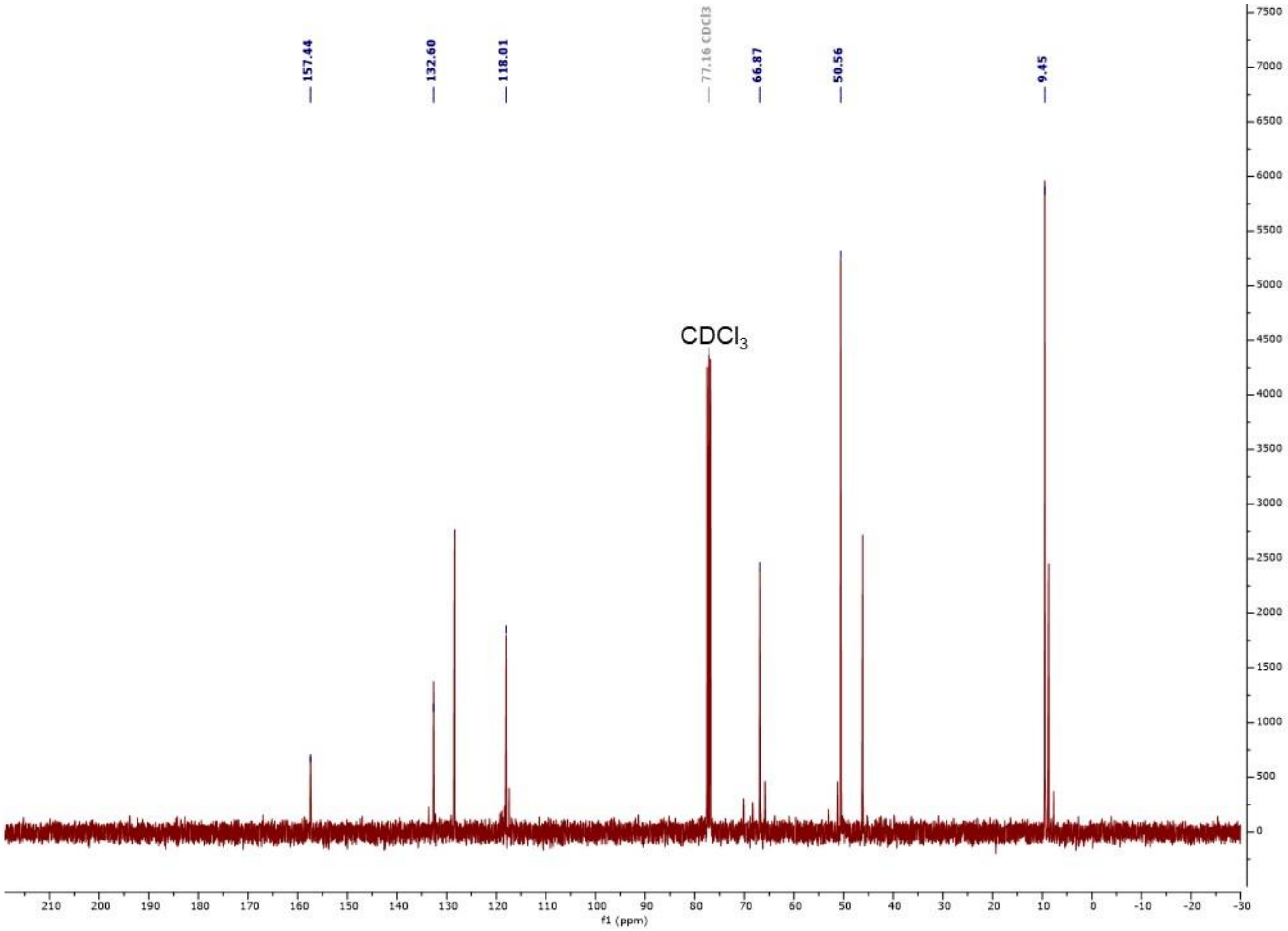

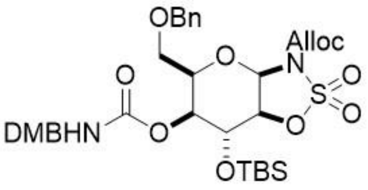

8

^{1}H NMR, $CDCl_3$, 500 MHz

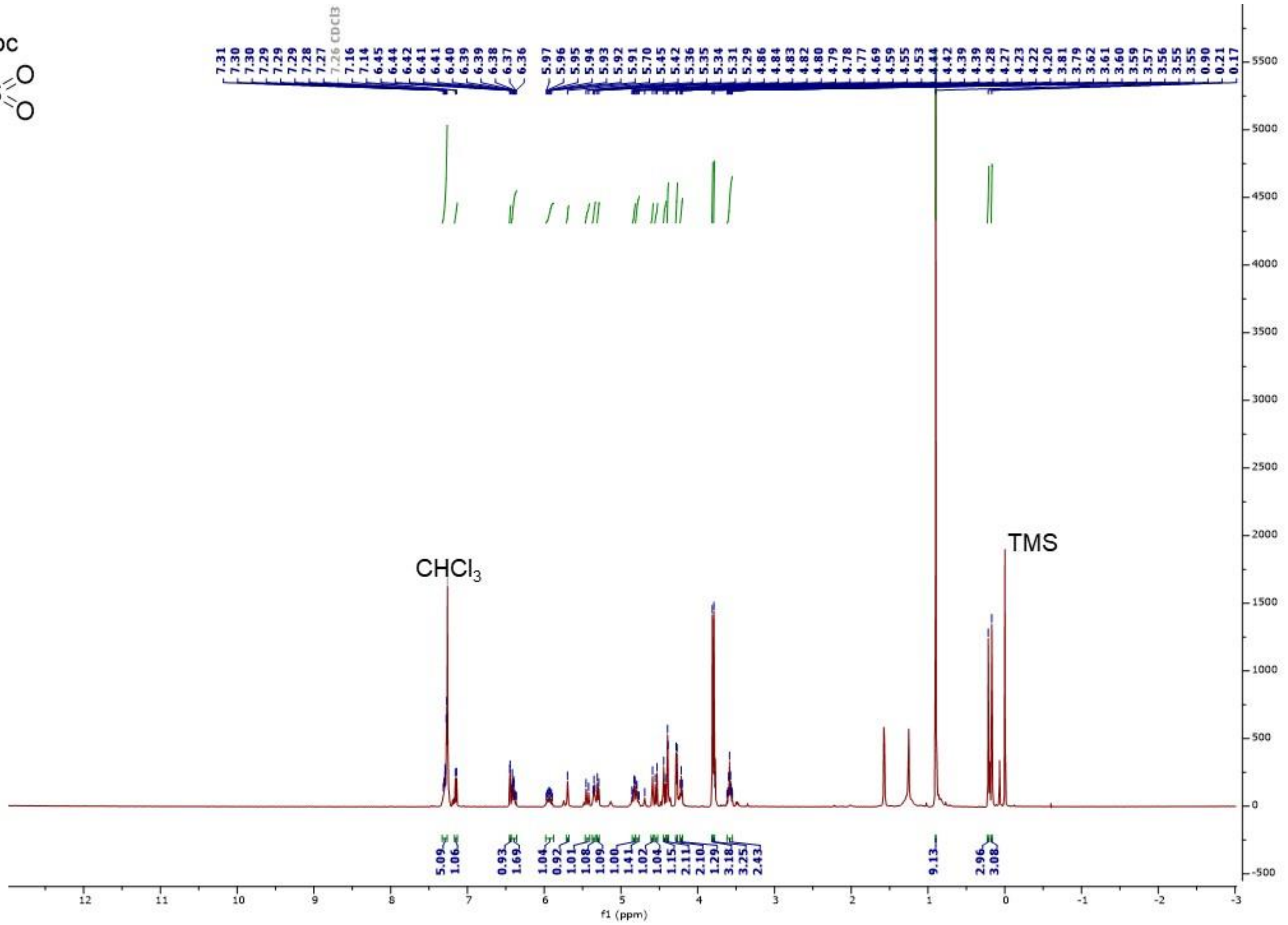

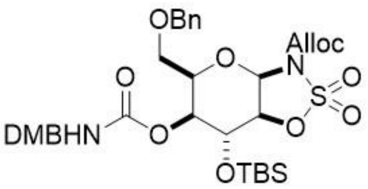

8

^{13}C NMR, $CDCl_3$, 125 MHz

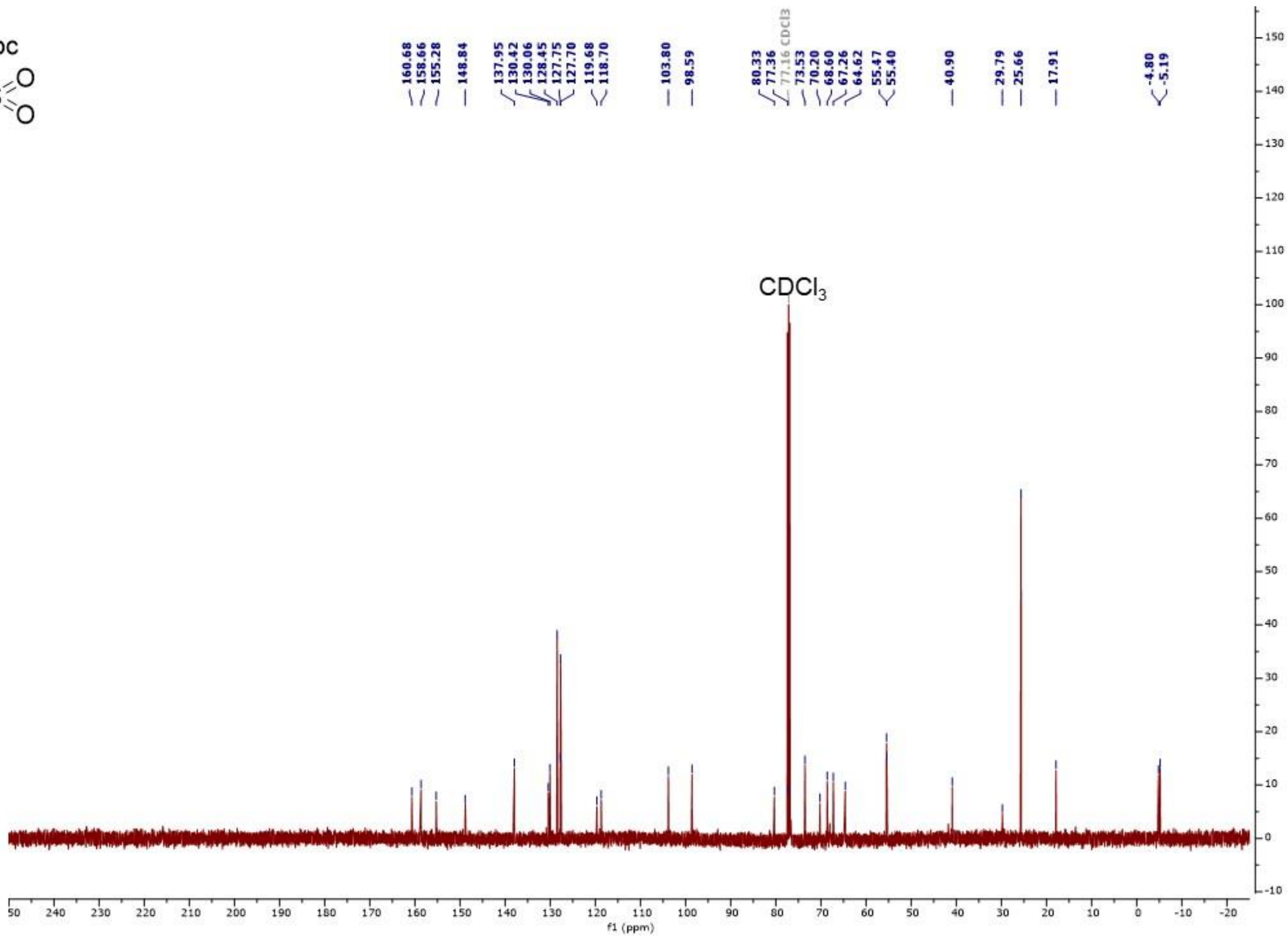

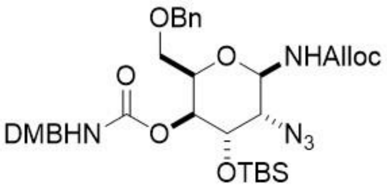

S11

^{1}H NMR, $(CD_3)_2CO$, 500 MHz

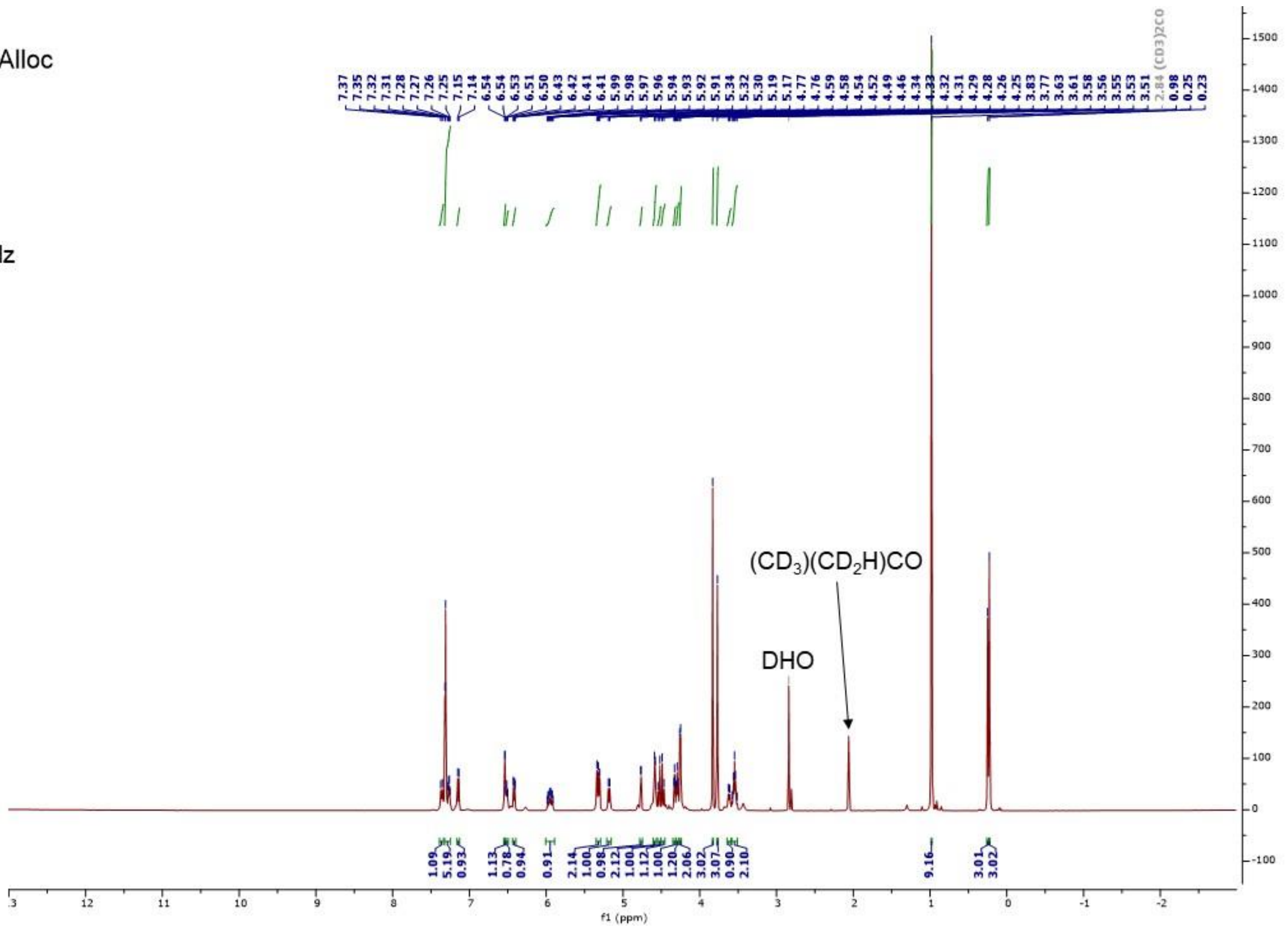

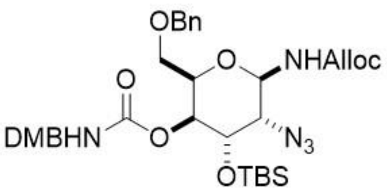

S11

^{13}C NMR, $(CD_3)_2CO$, 100 MHz

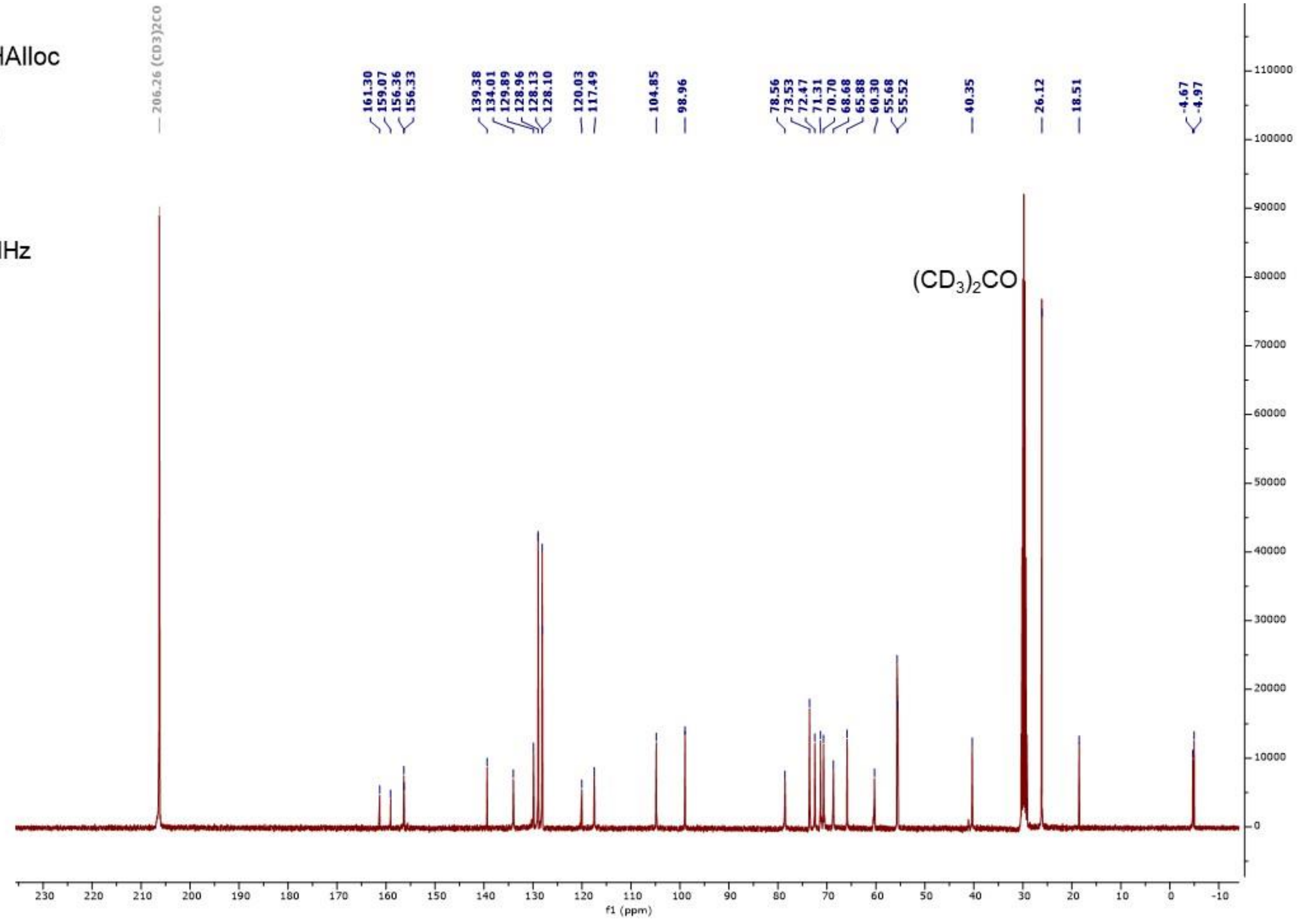

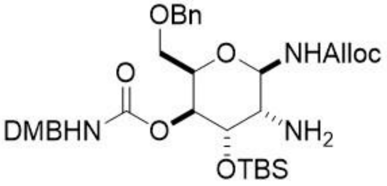

7

^1H NMR, $CDCl_3$, 500 MHz

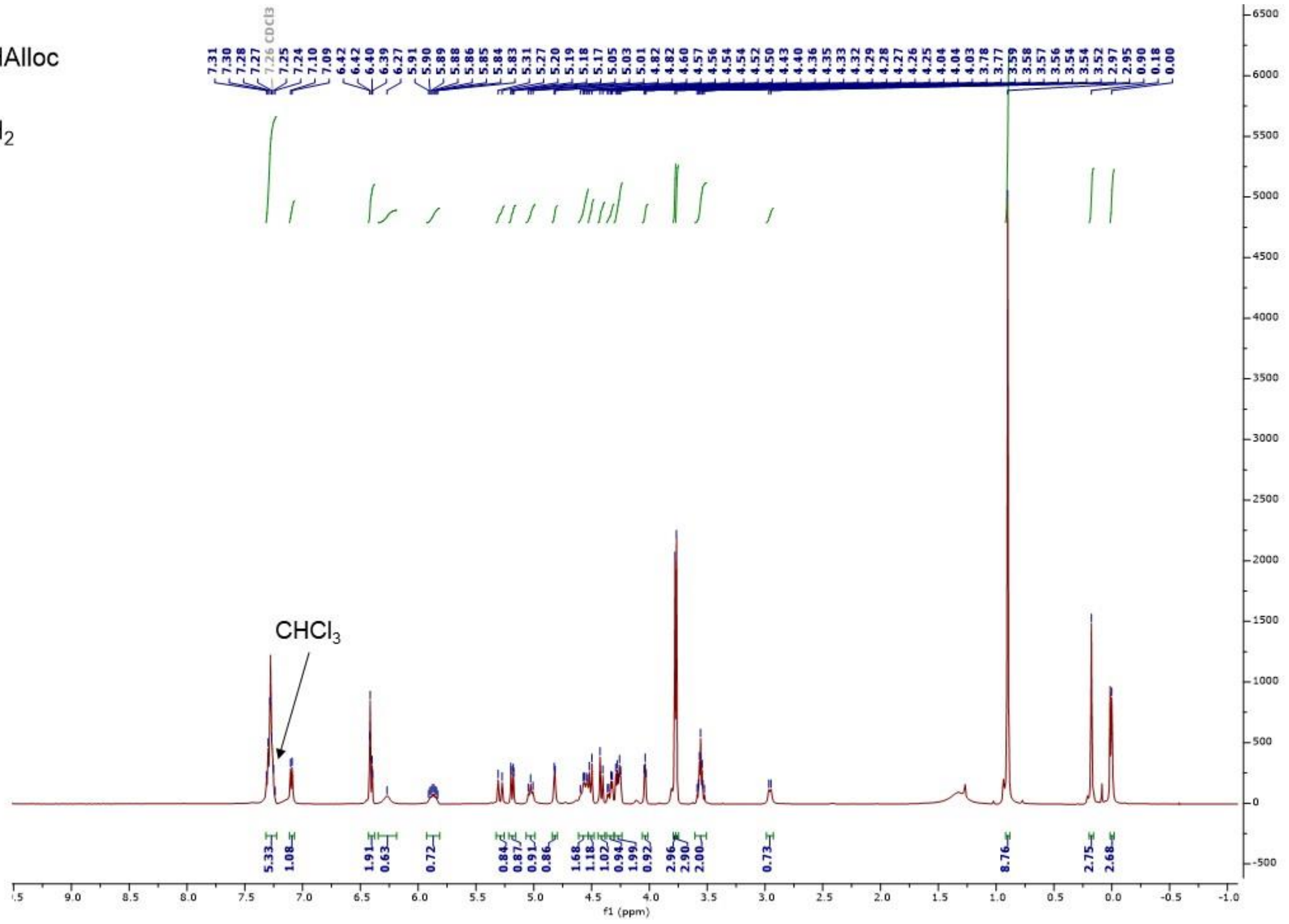

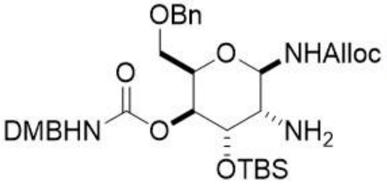

7

^{13}C NMR, $CDCl_3$, 125 MHz

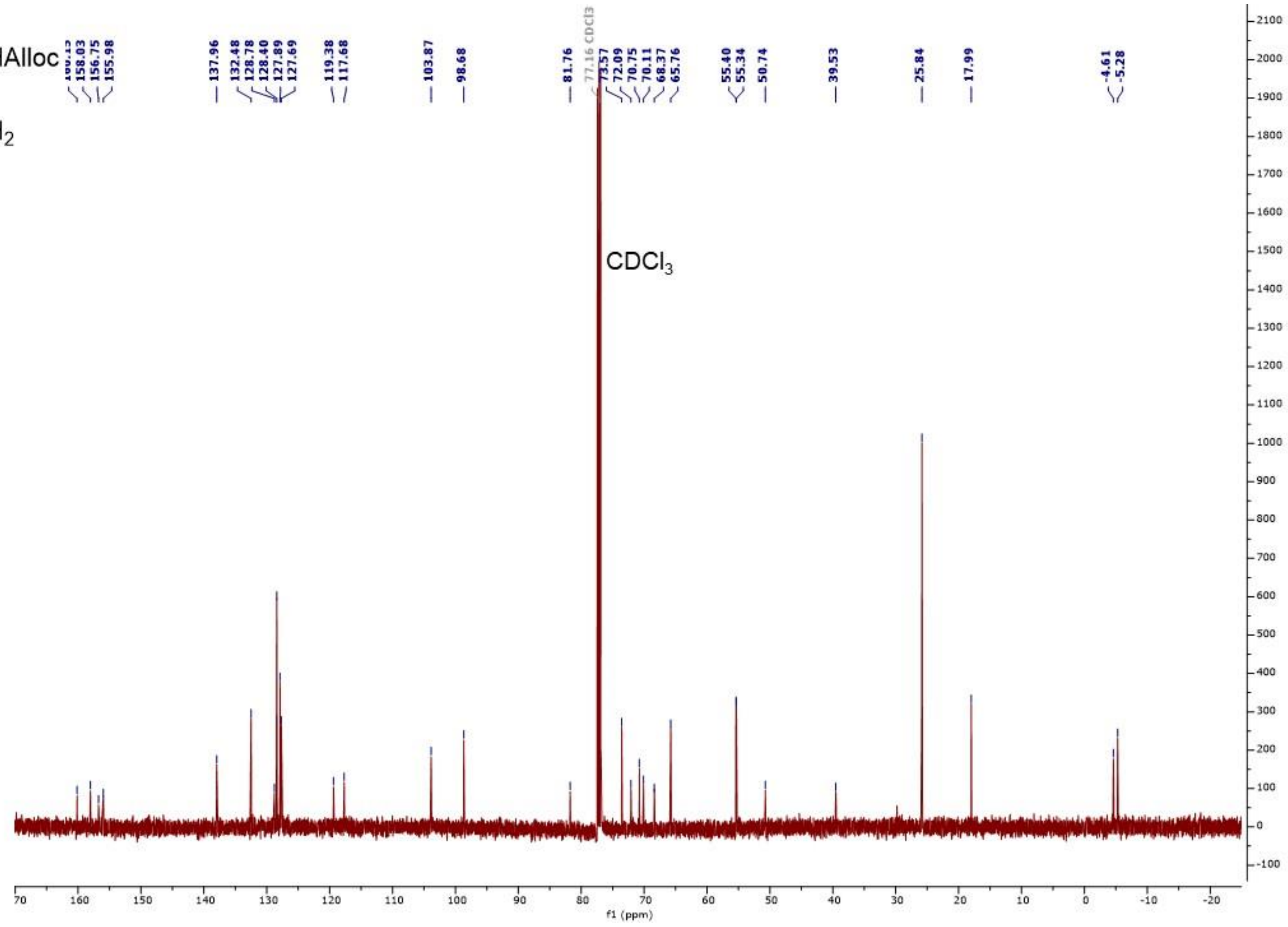

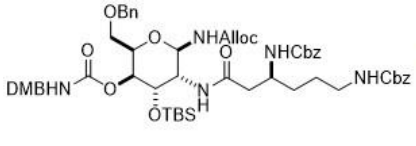

25

^{1}H NMR, $CDCl_3$, 500 MHz

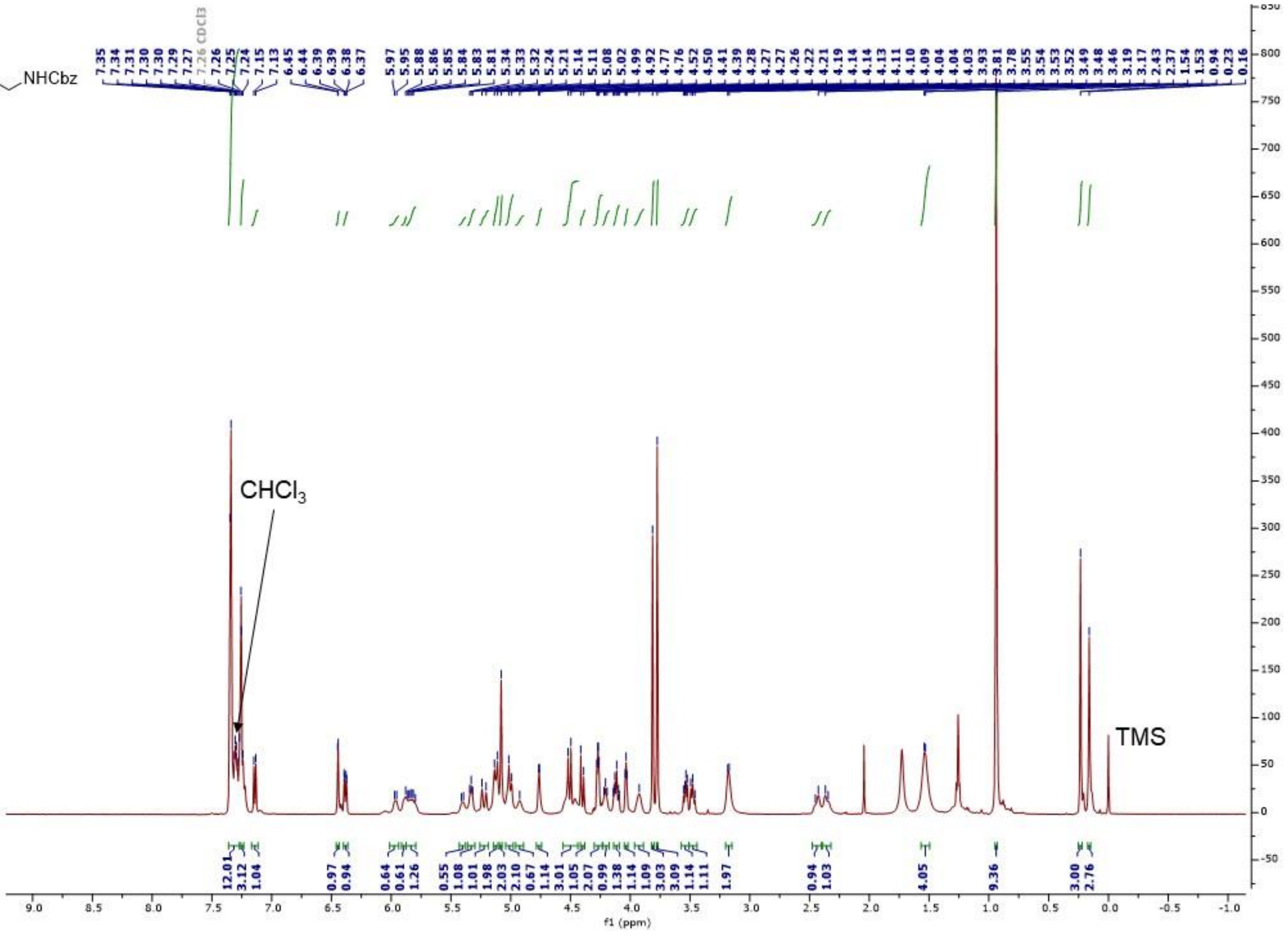

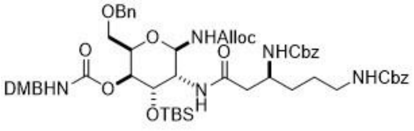

25

^{13}C NMR, $CDCl_3$, 100 MHz

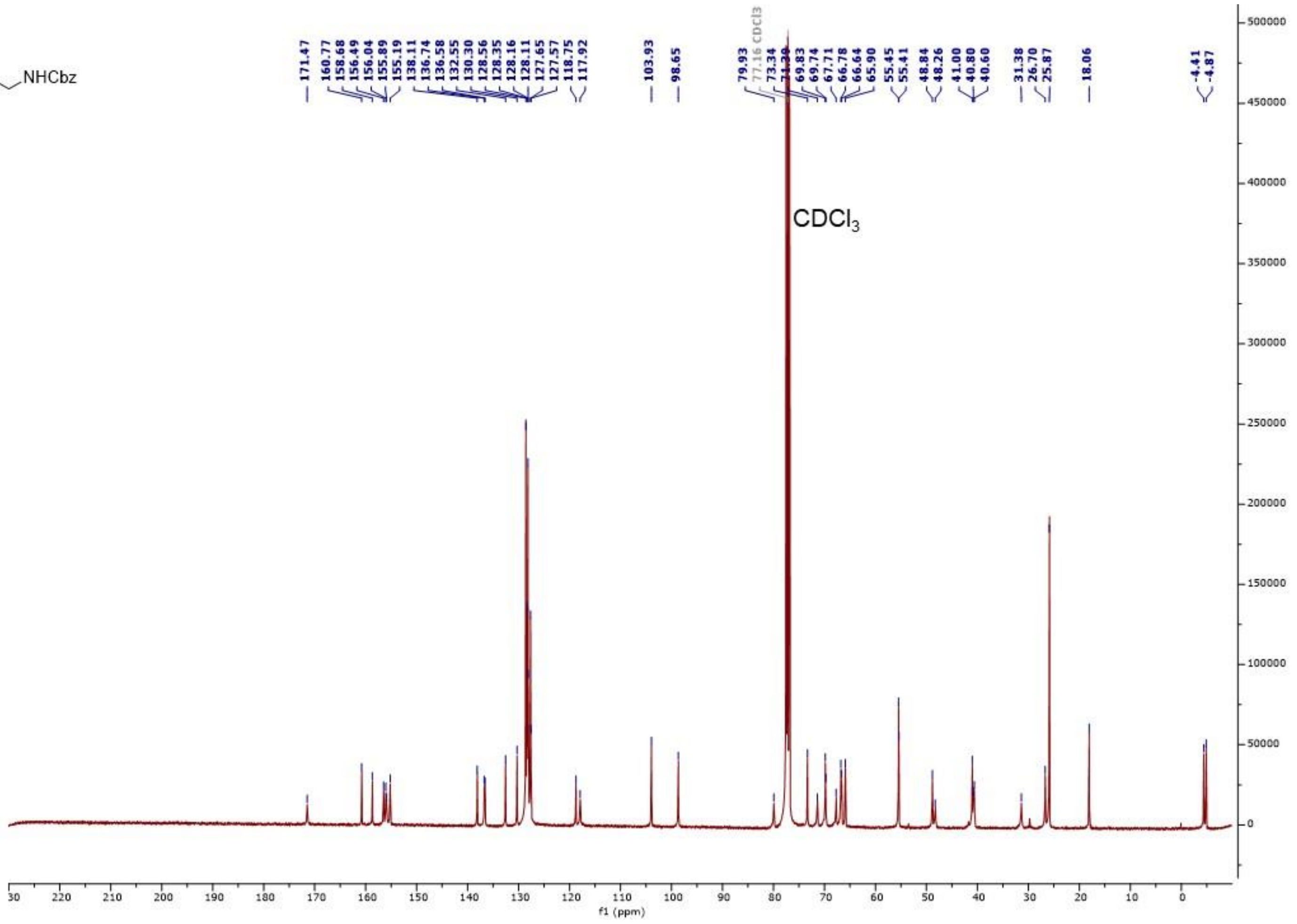

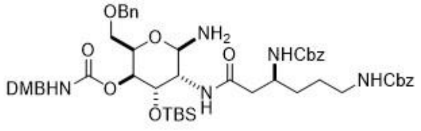

26

^{1}H NMR, CD_3OD, 500 MHz

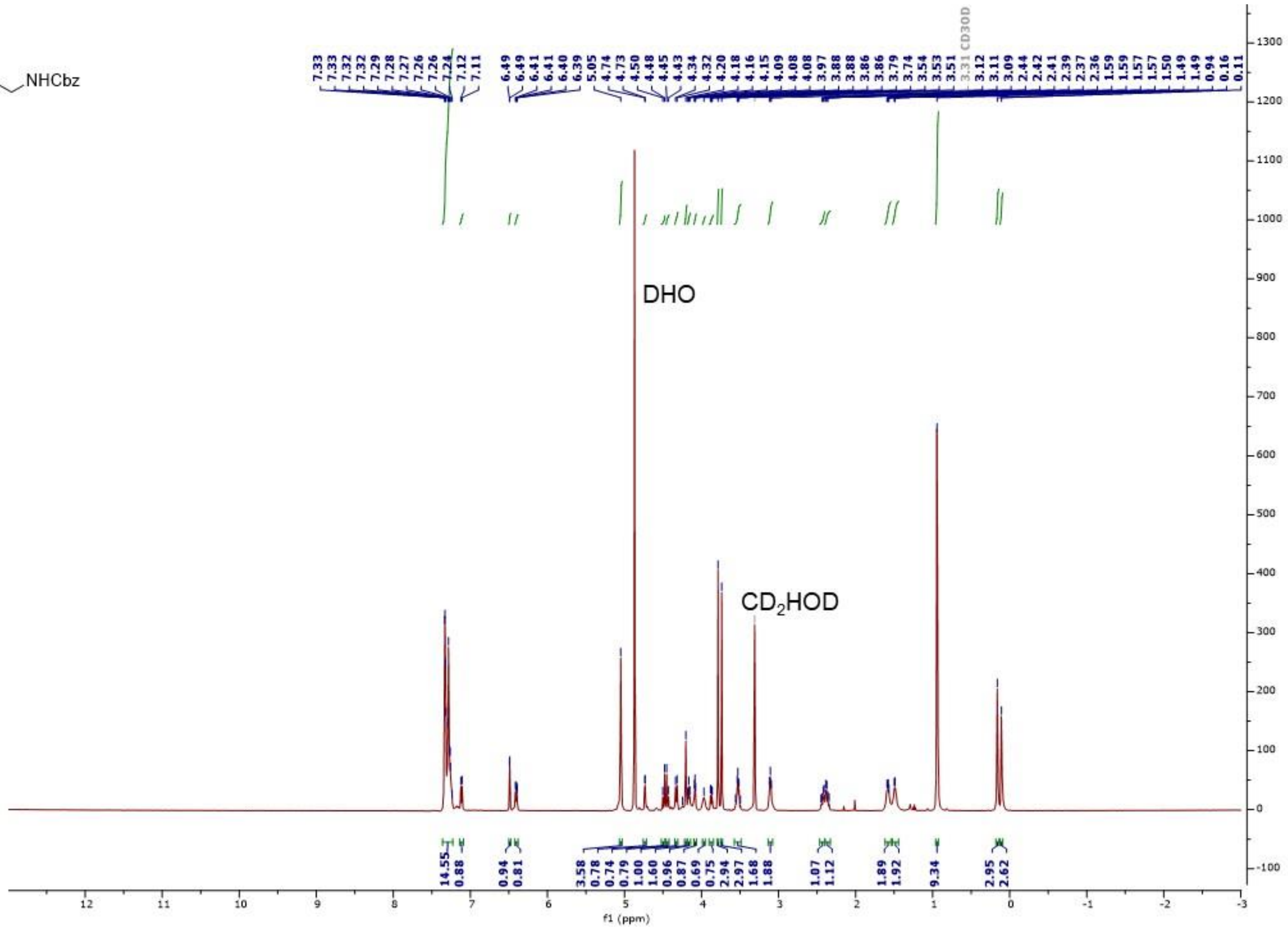

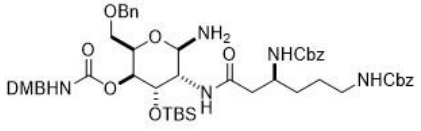

26

^{13}C NMR, $CDCl_3$, 100 MHz

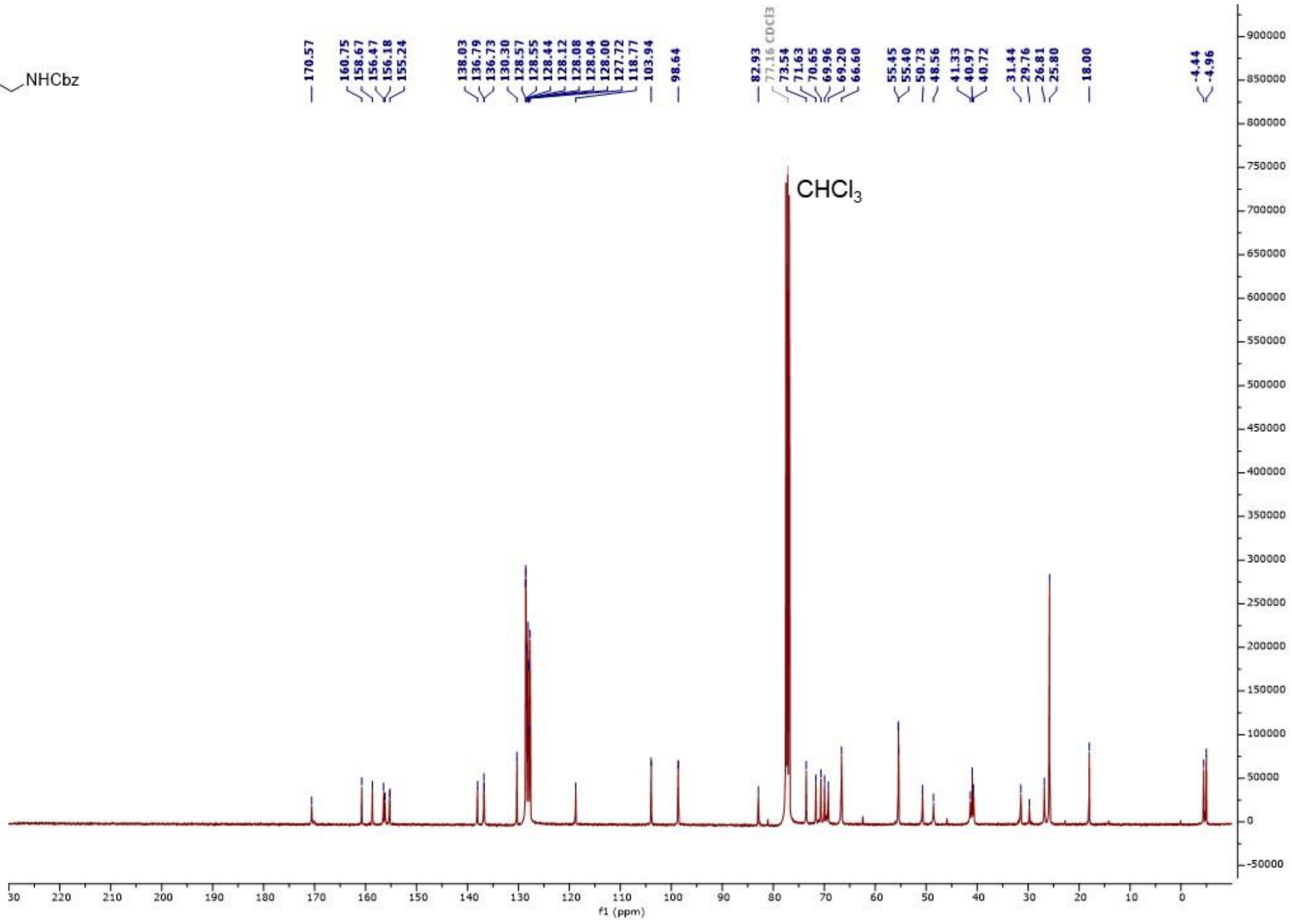

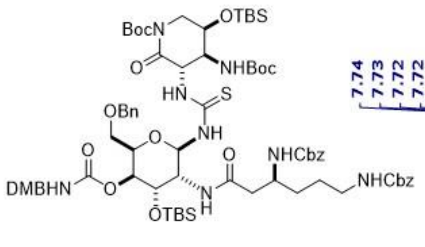

2

^{1}H NMR, $CDCl_3$, 500 MHz

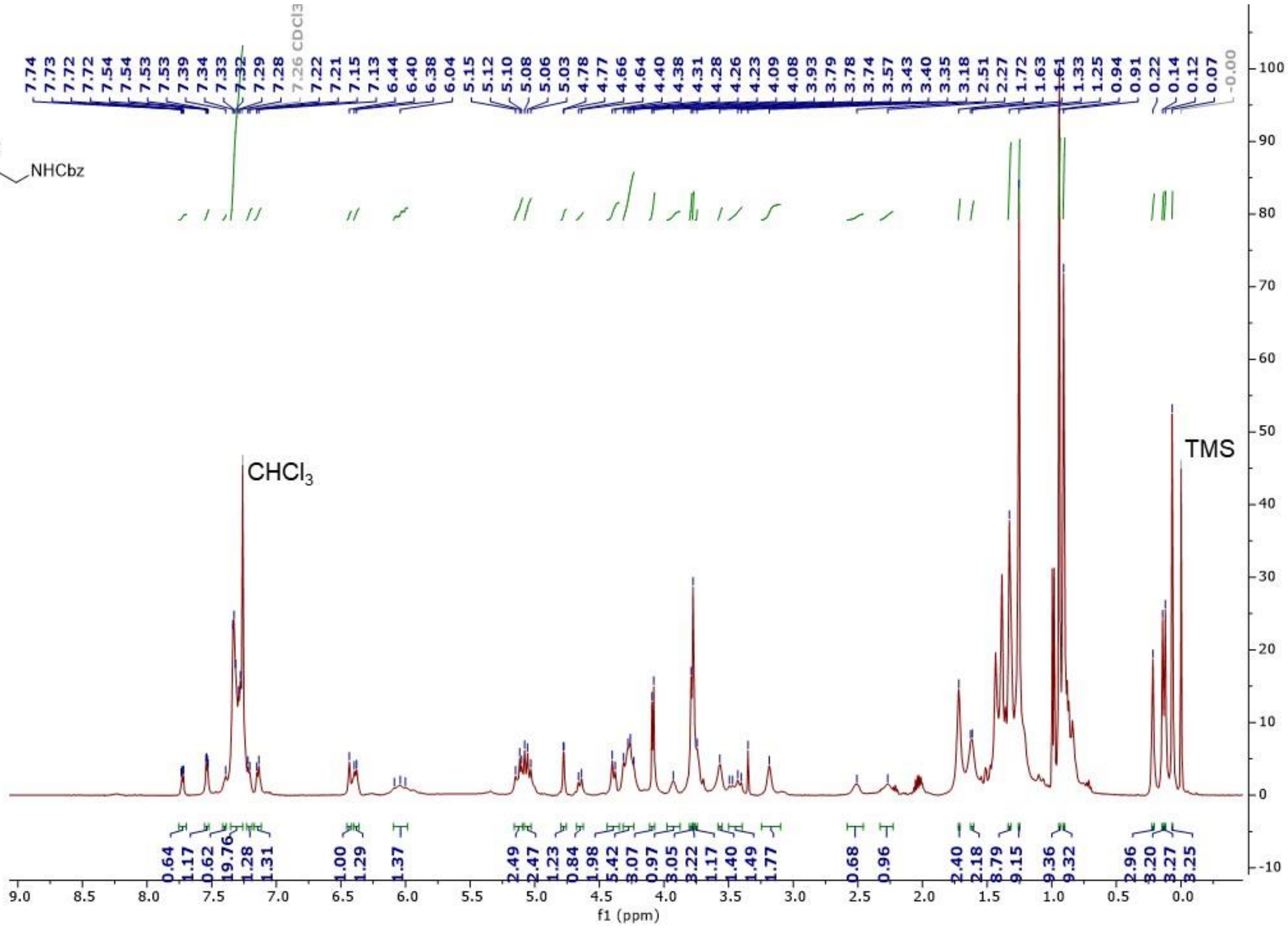

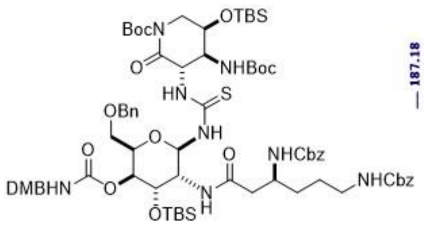

2

^{13}C NMR, $CDCl_3$, 100 MHz

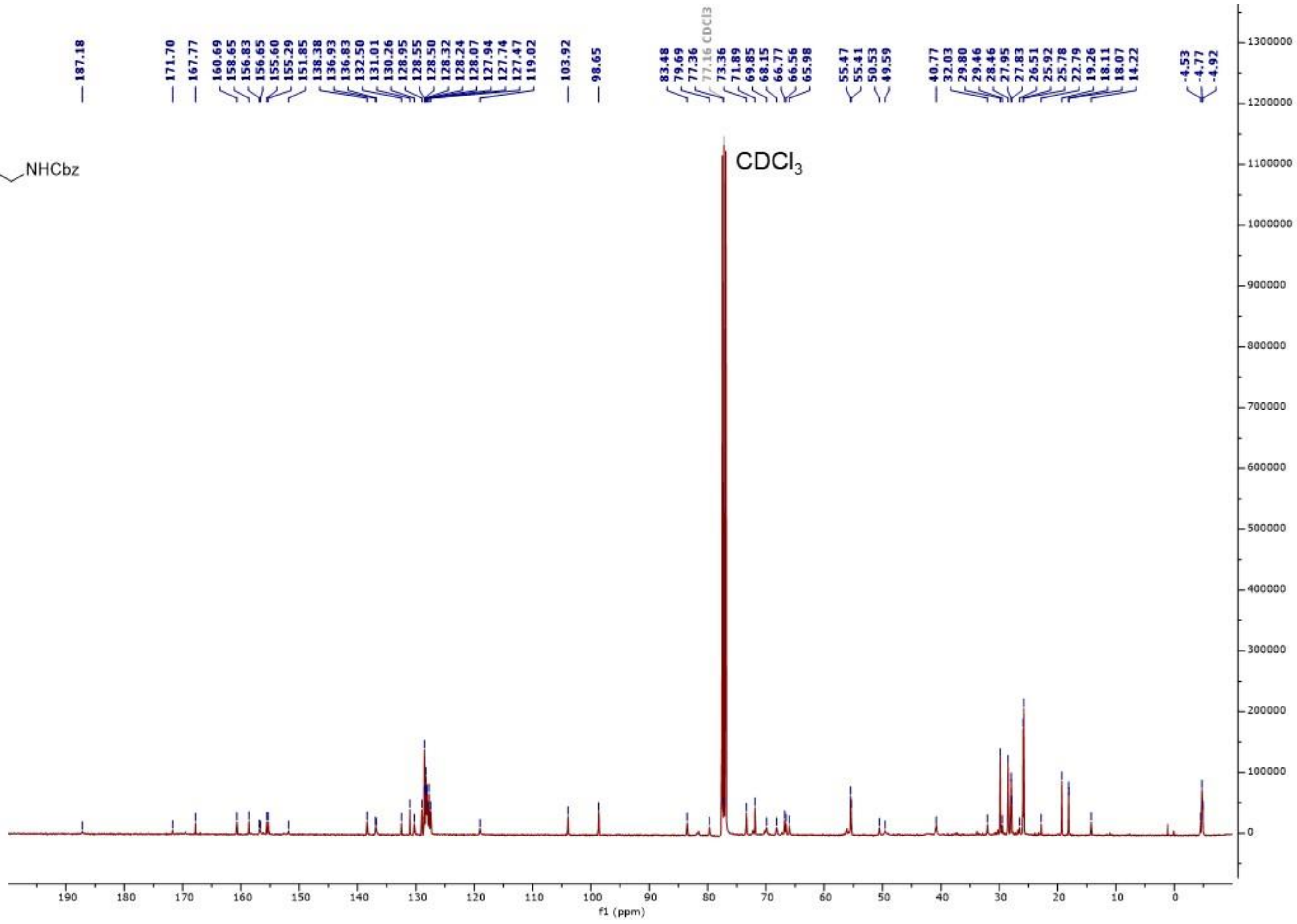

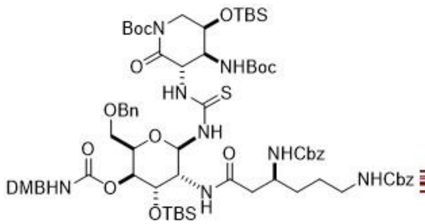

2

COSY, $CDCl_3$, 500 MHz

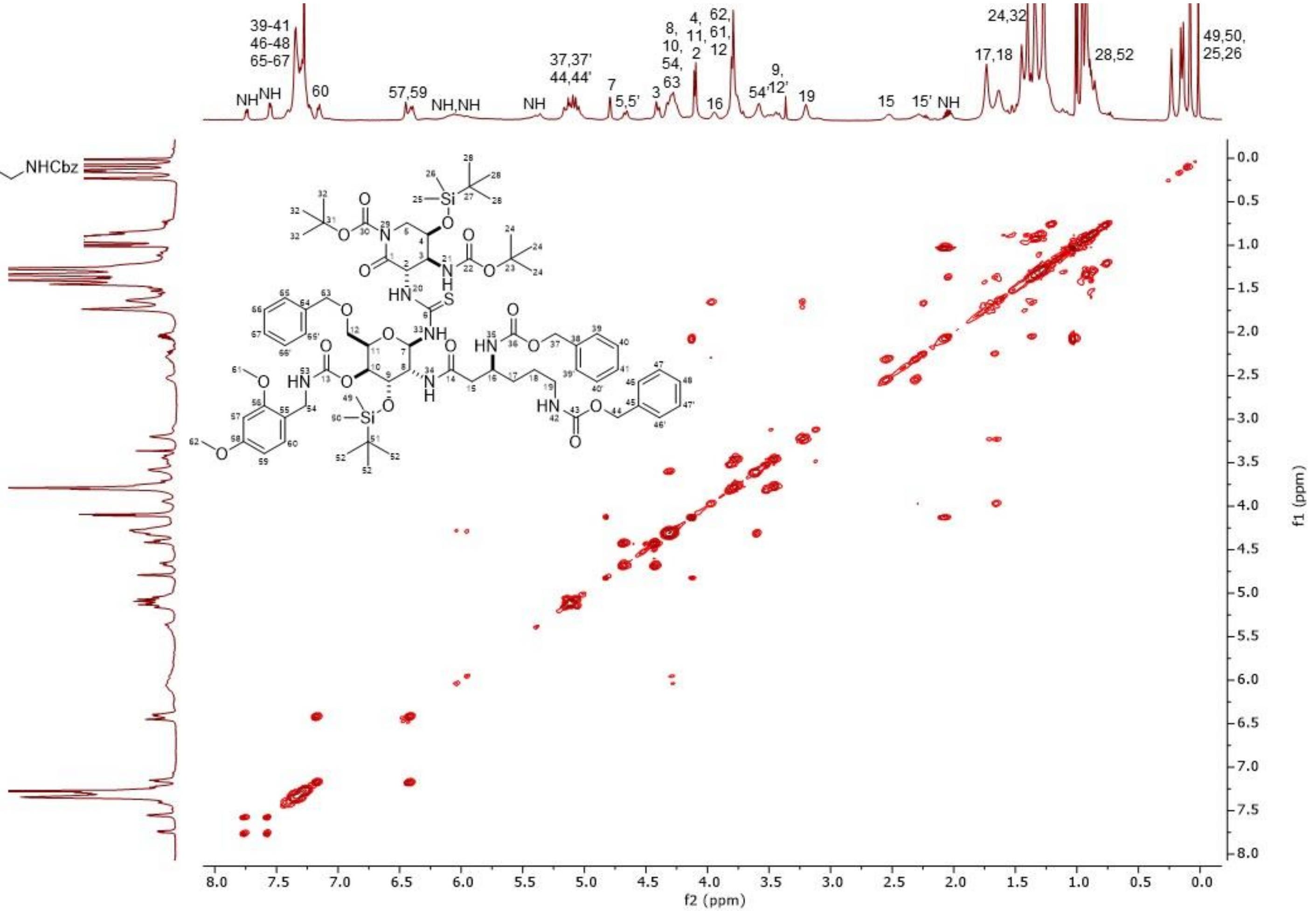

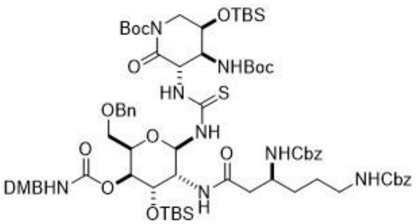

2

HSQC, $CDCl_3$, 500 MHz

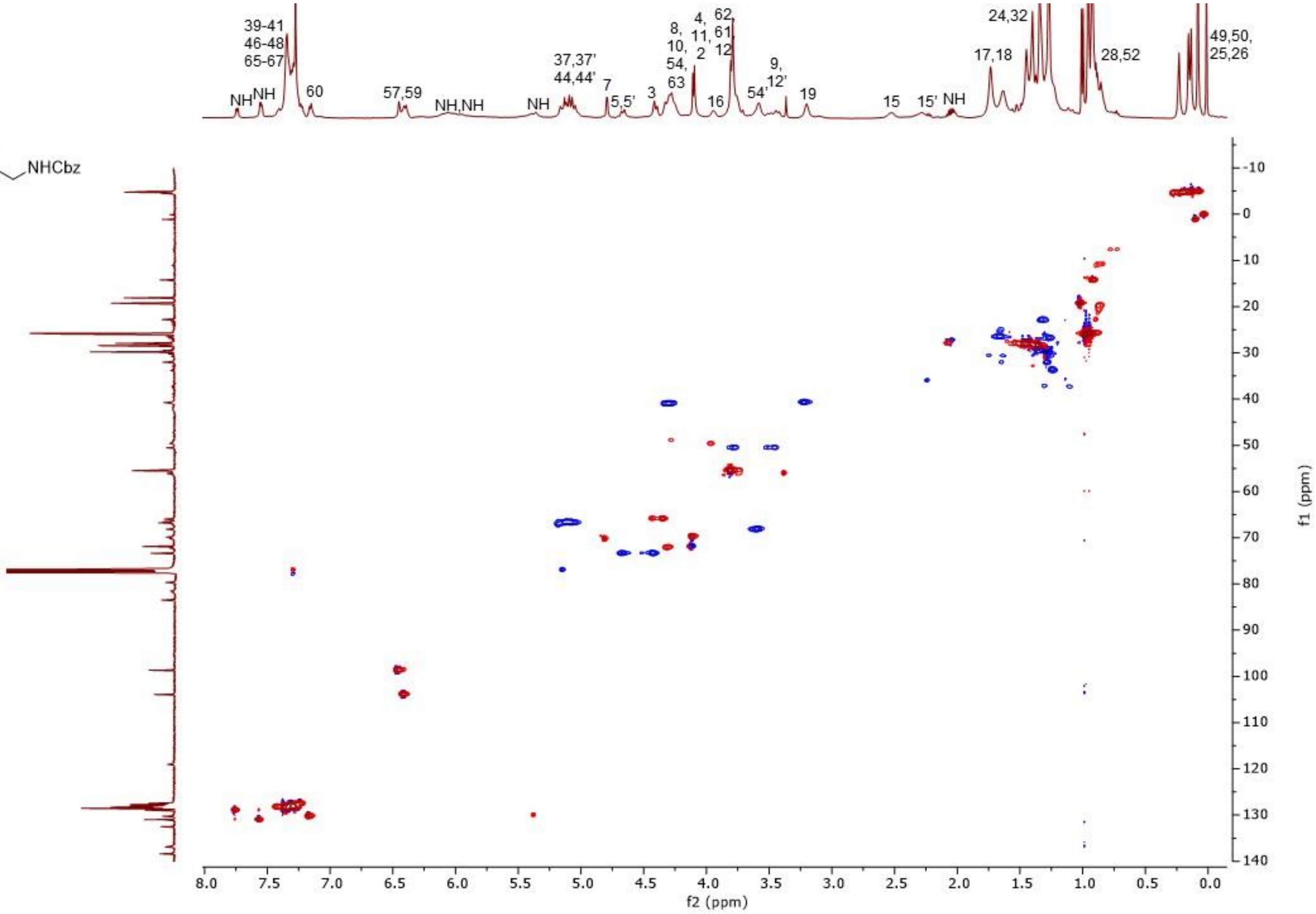

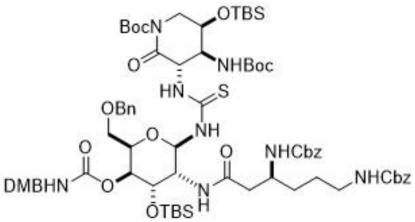

2

HMBC, $CDCl_3$, 500 MHz

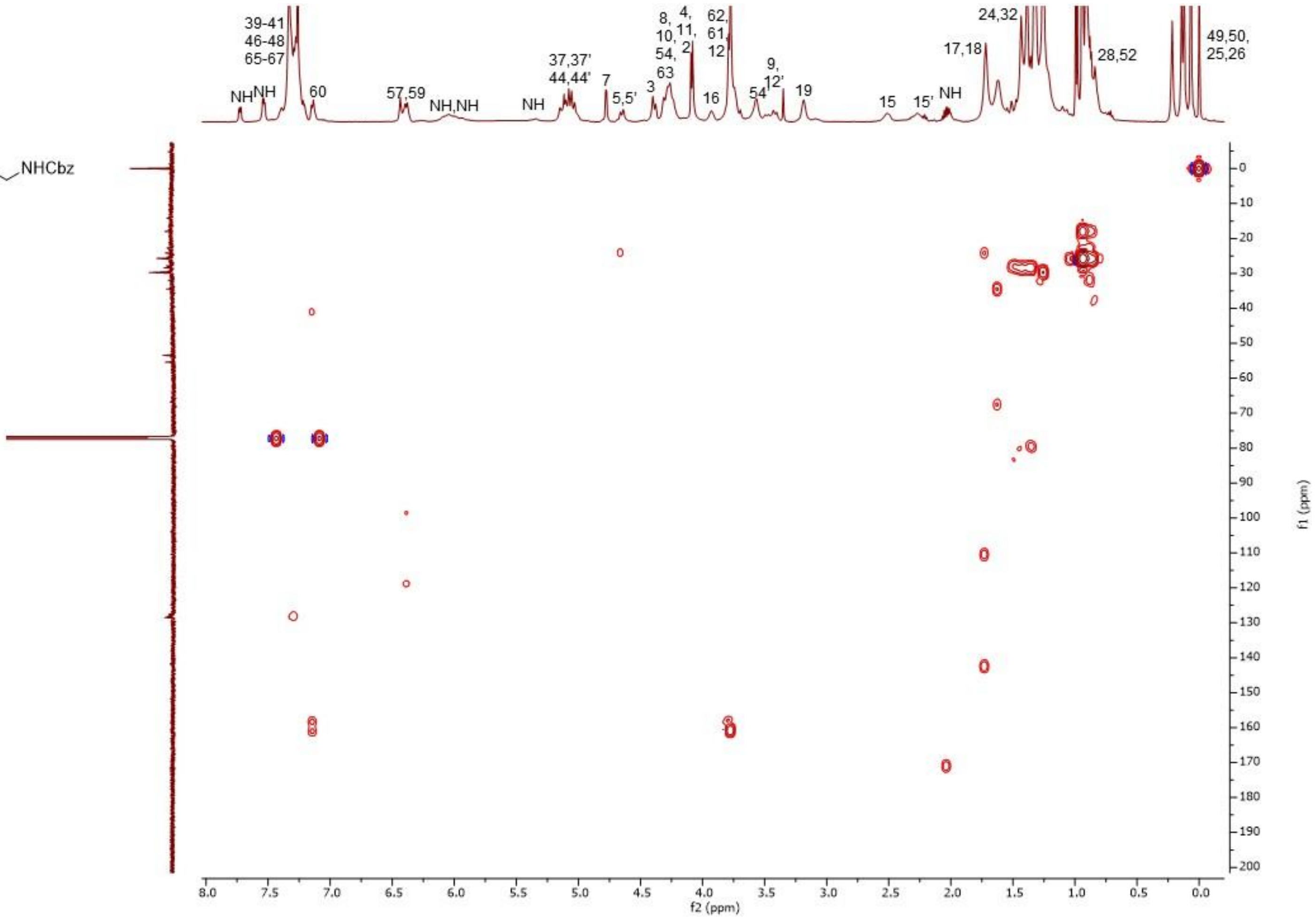

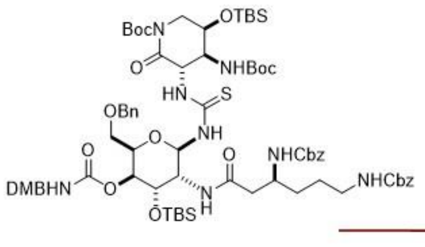

2

ROESY, $CDCl_3$, 500 MHz

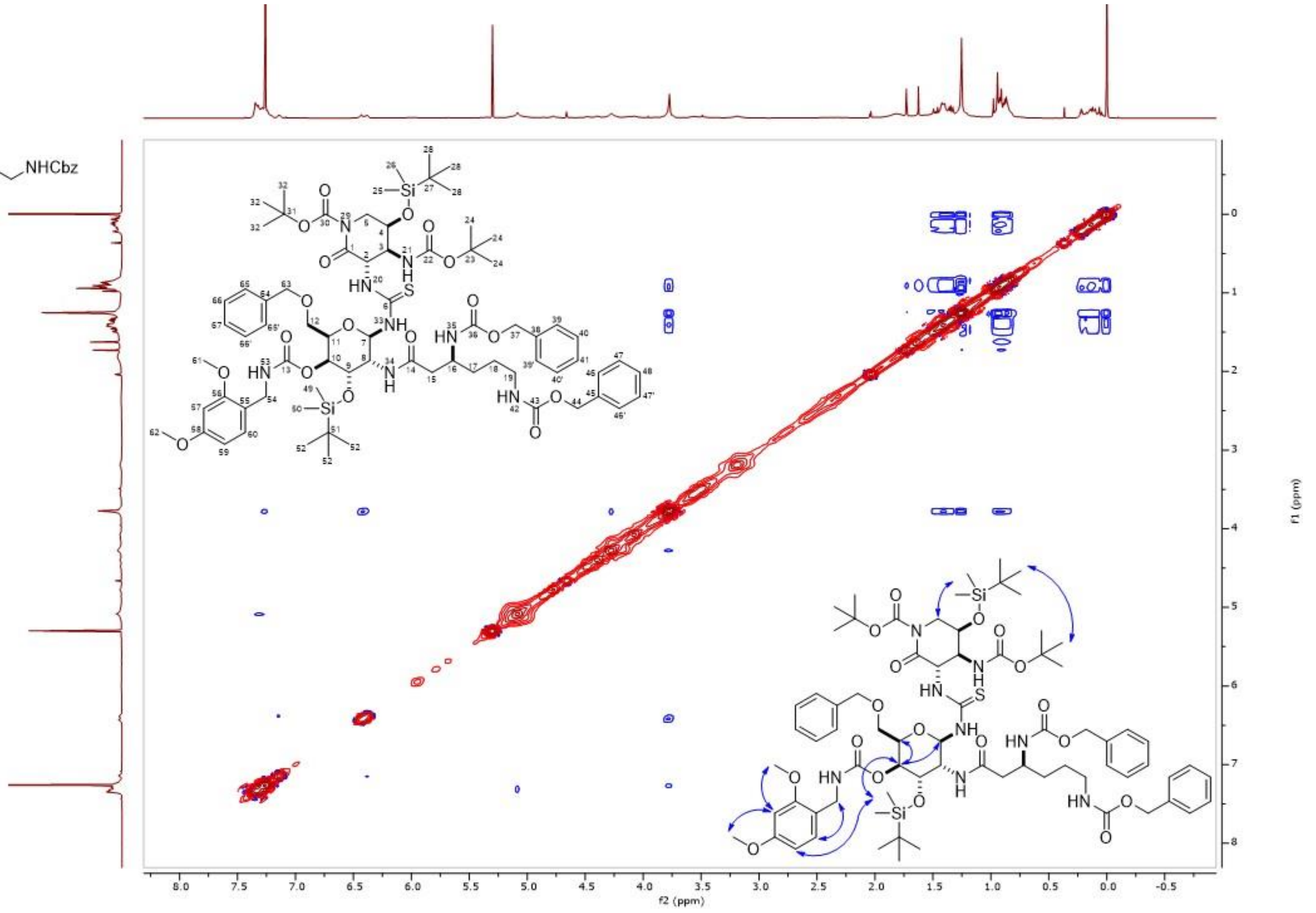

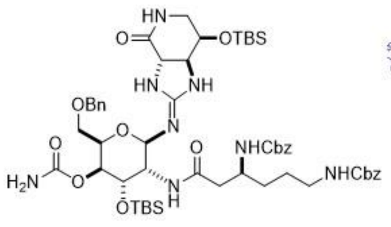

28

^{1}H NMR, CD_3OD, 500 MHz

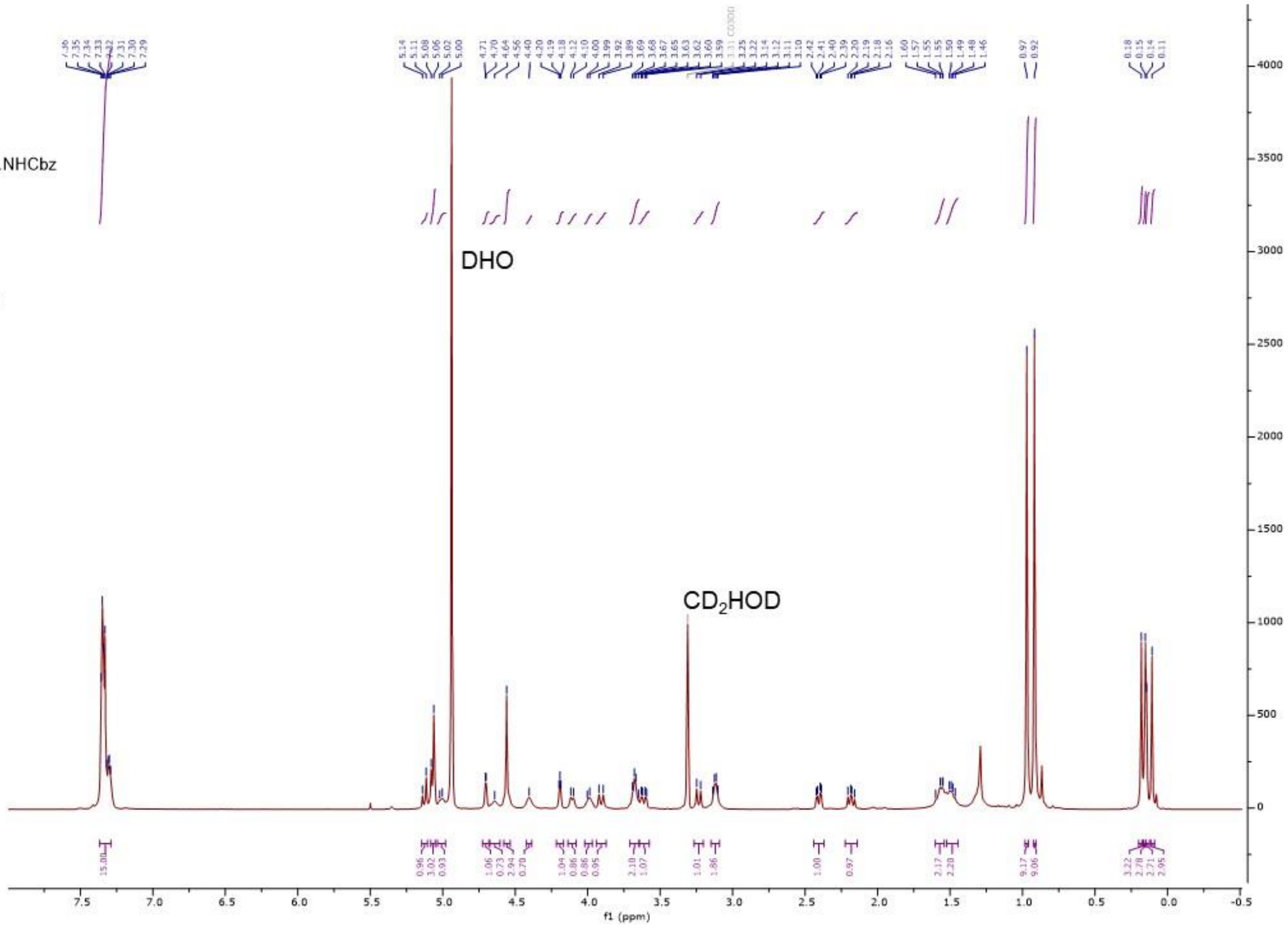

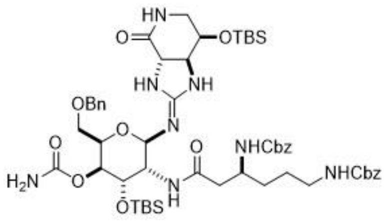

28

^{13}C NMR, $CDCl_3$, 100 MHz

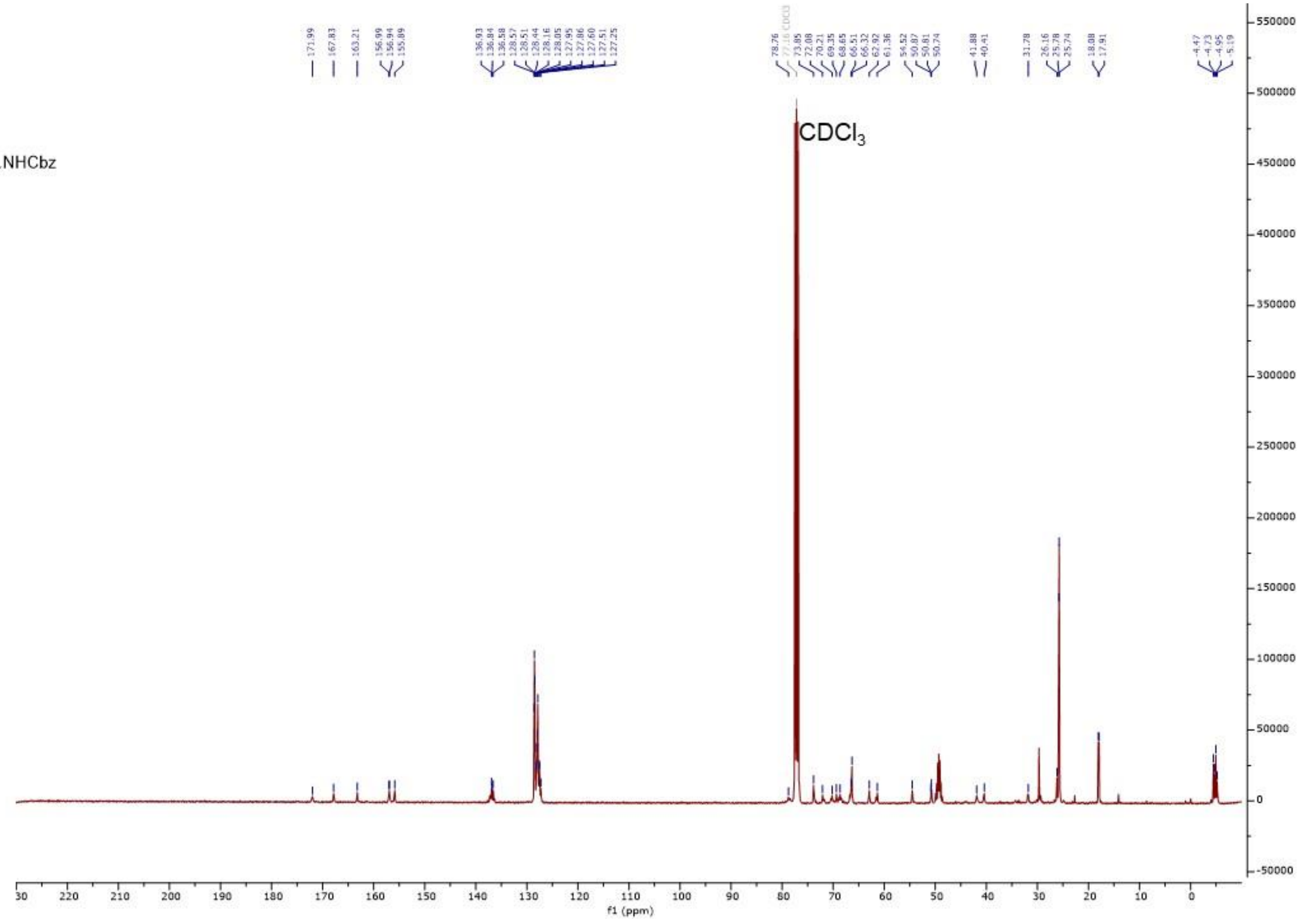

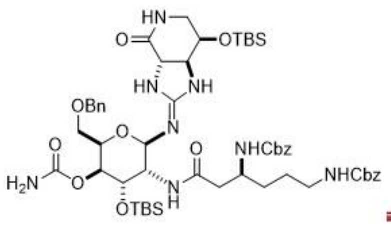

28

COSY, $CDCl_3$, 500 MHz

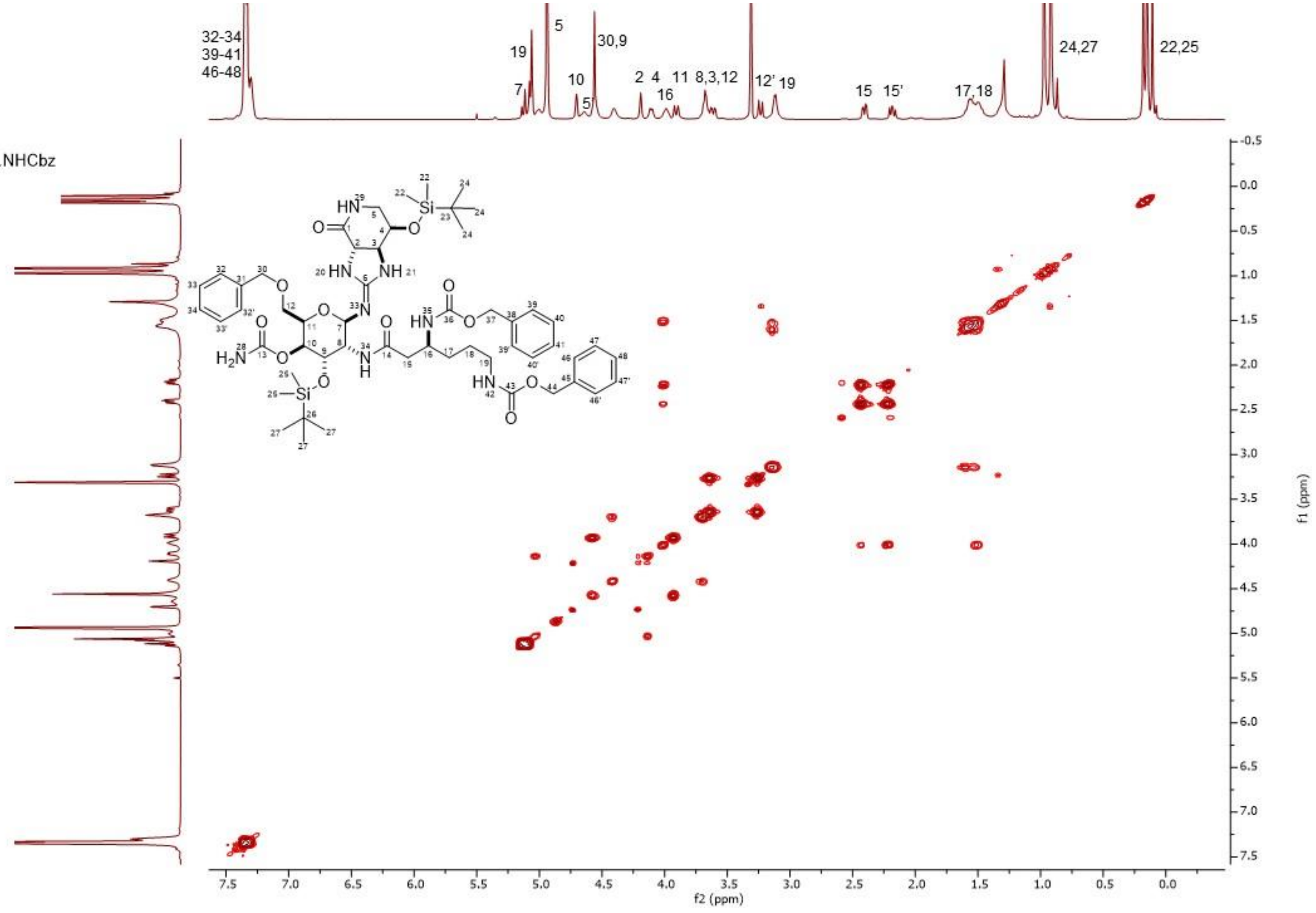

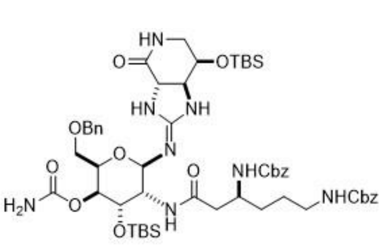

28

HSQC, CDCl3, 500 MHz

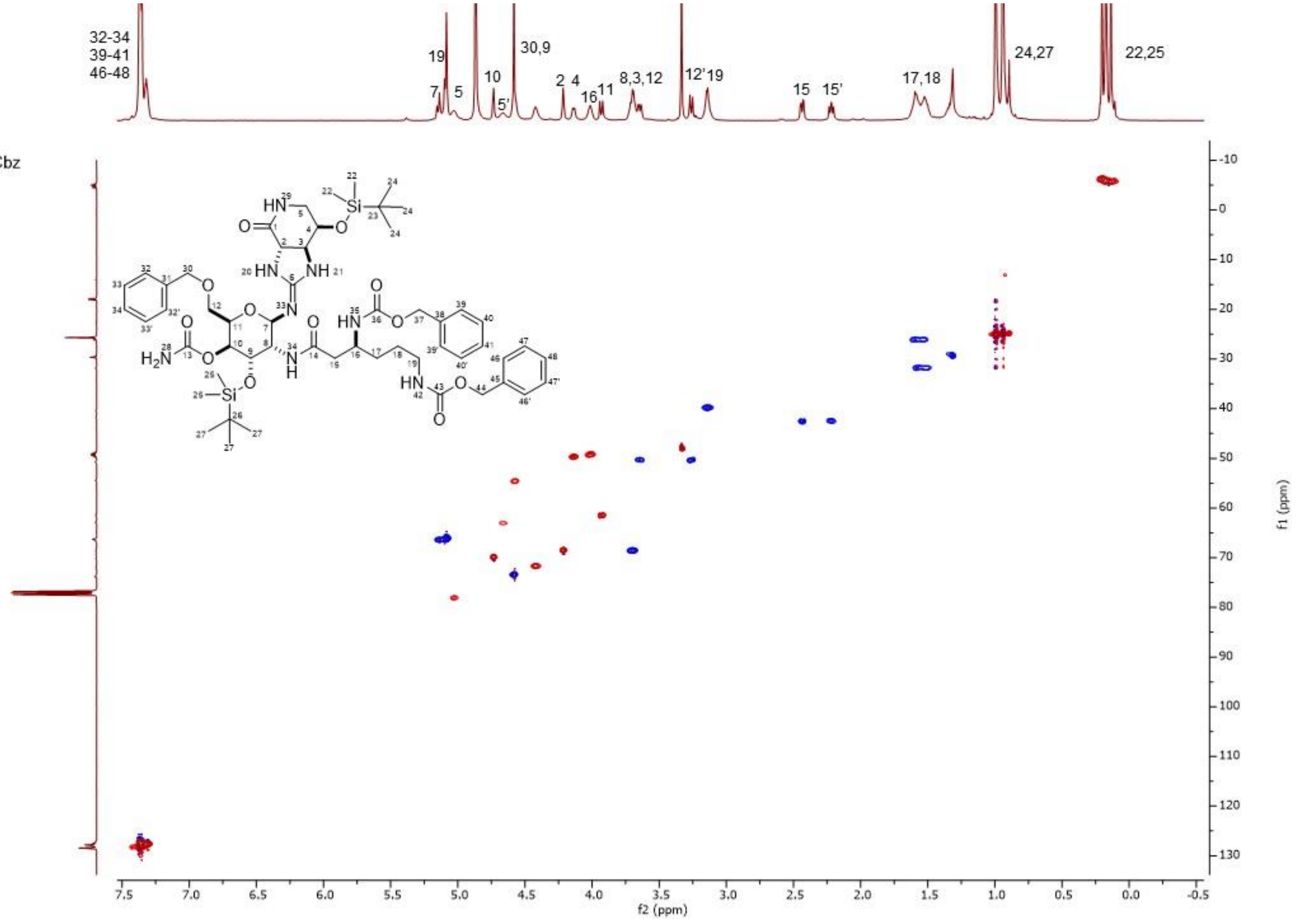

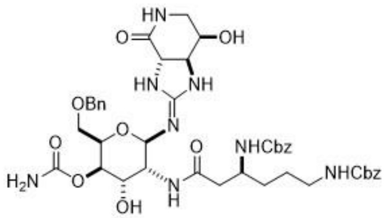

29

^{1}H NMR, CD_3OD, 500 MHz

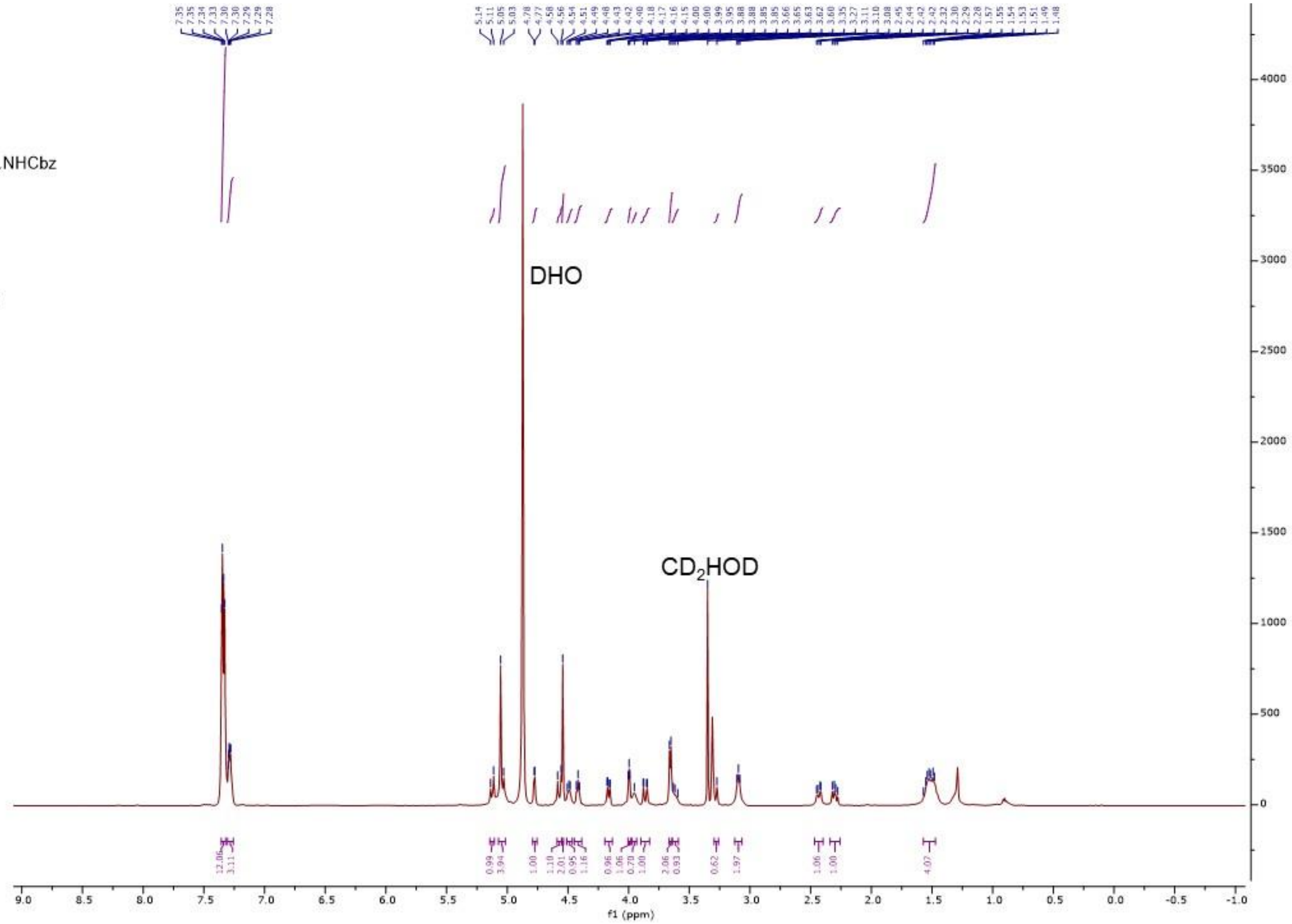

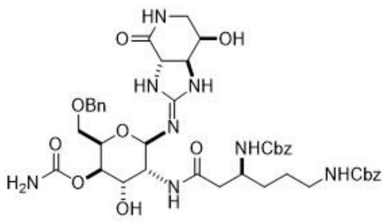

29

^{13}C NMR, CD_3OD, 150 MHz

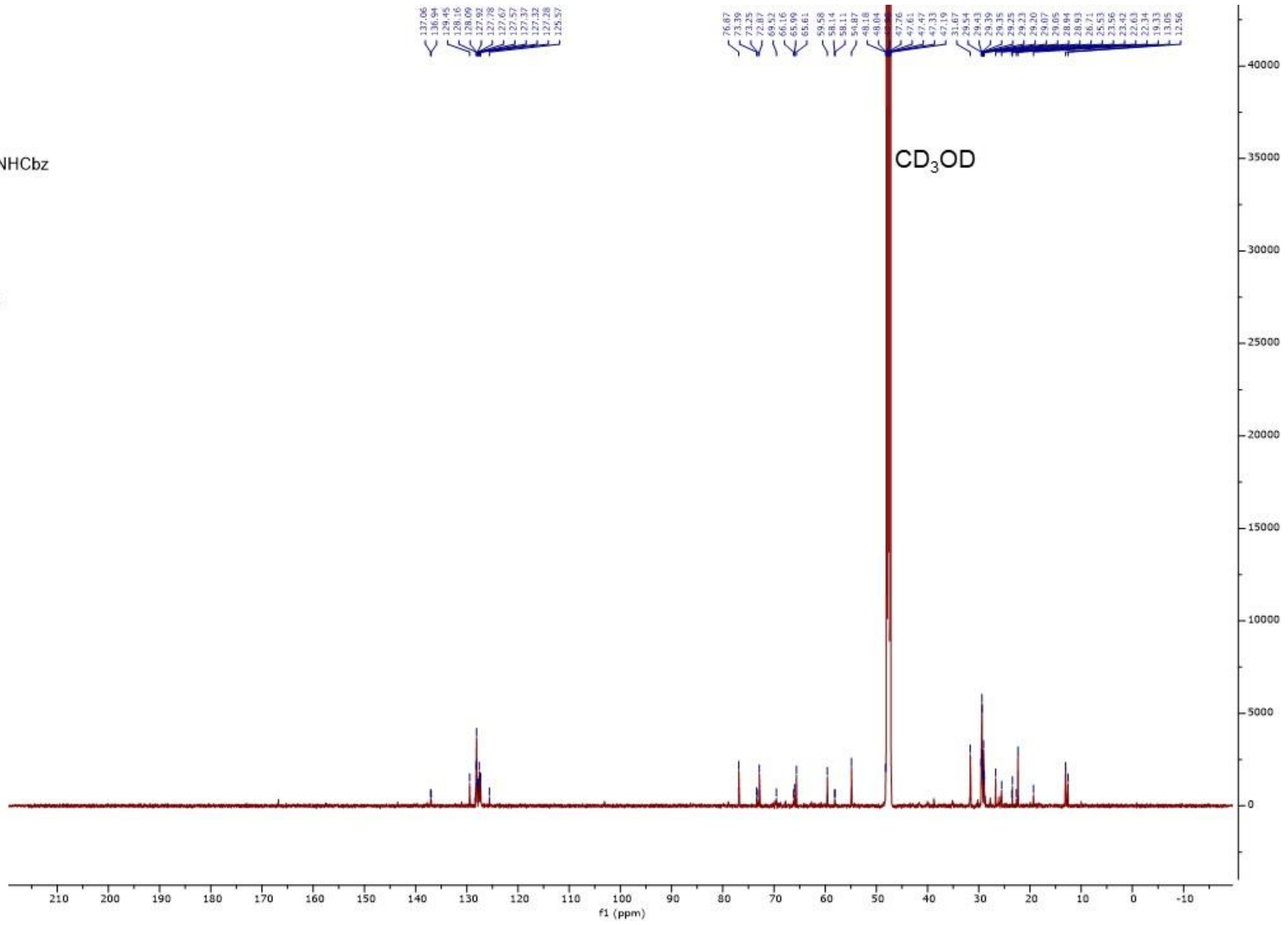

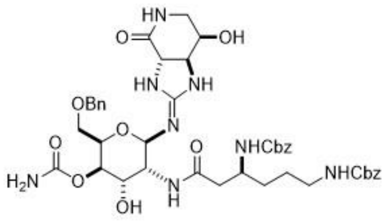

29

COSY, CD_3OD, 500 MHz

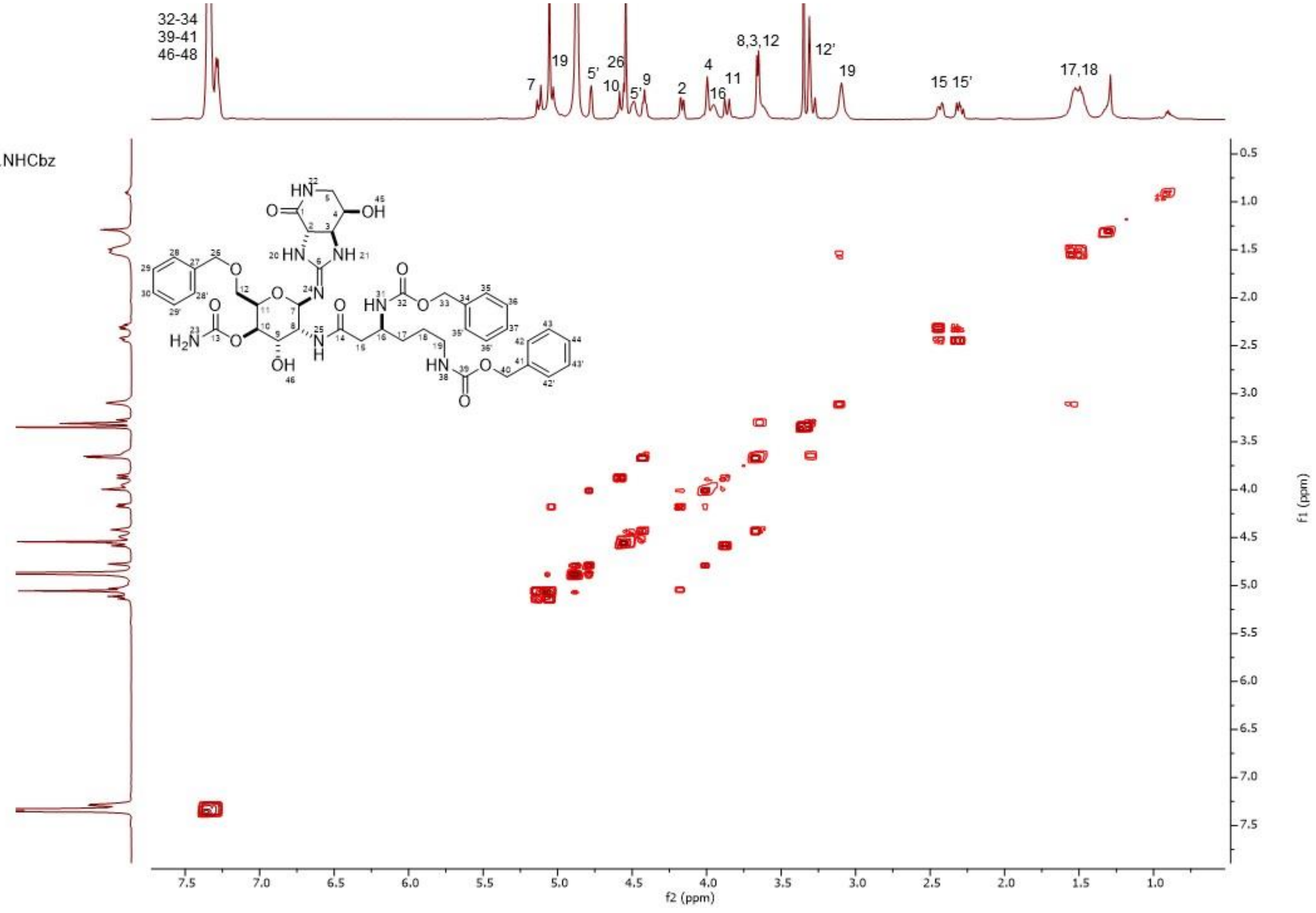

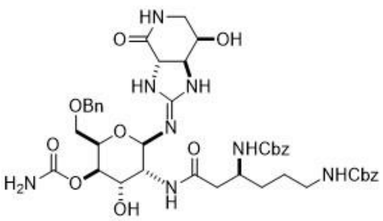

29

HSQC, CD_3OD, 600 MHz

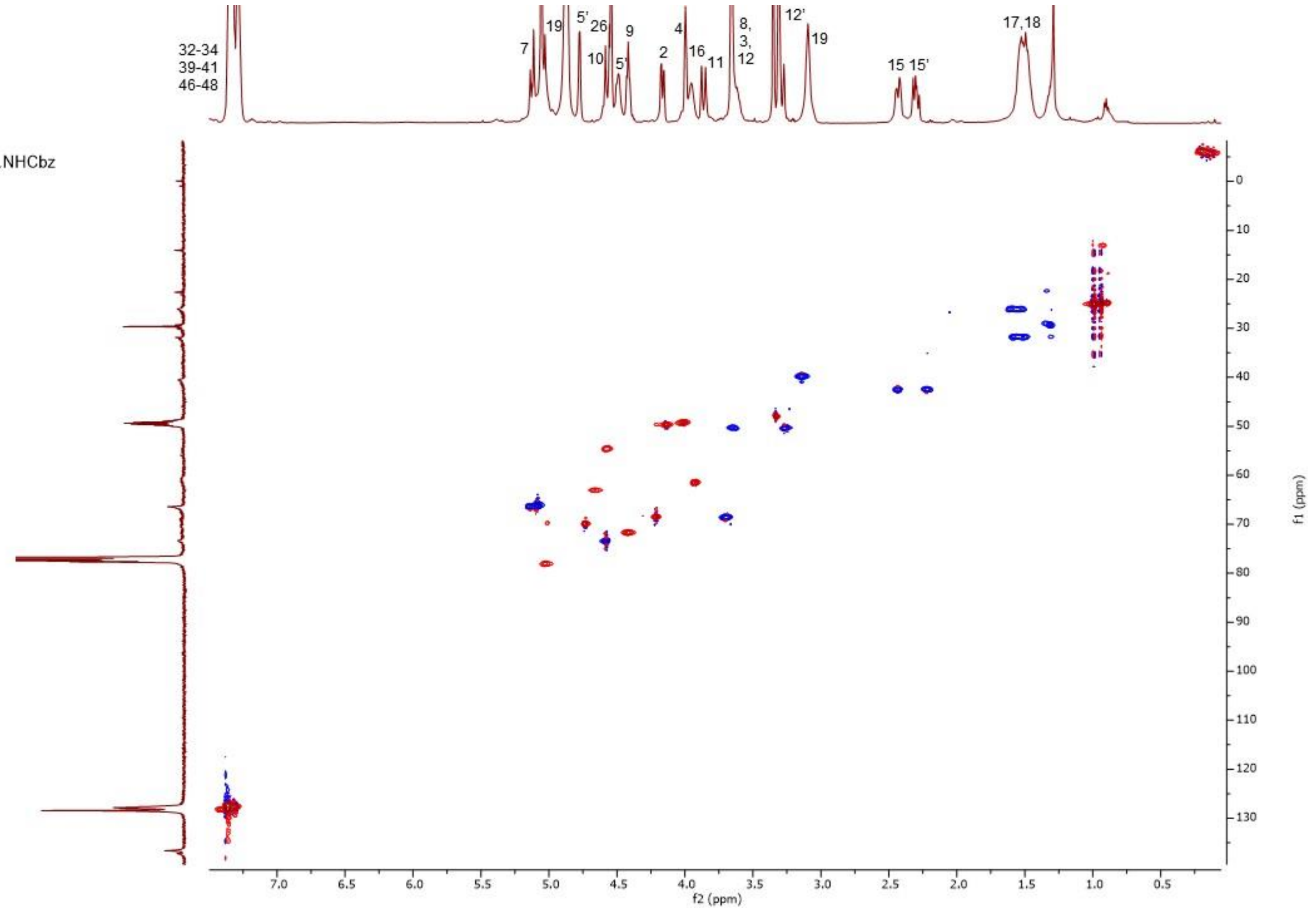

Synthetic streptothricin F.

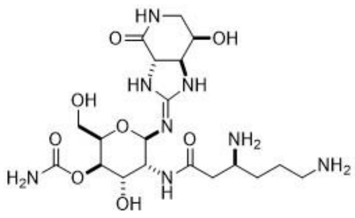

streptothricin F (**1**)

^1H NMR, D_2O, 500 MHz

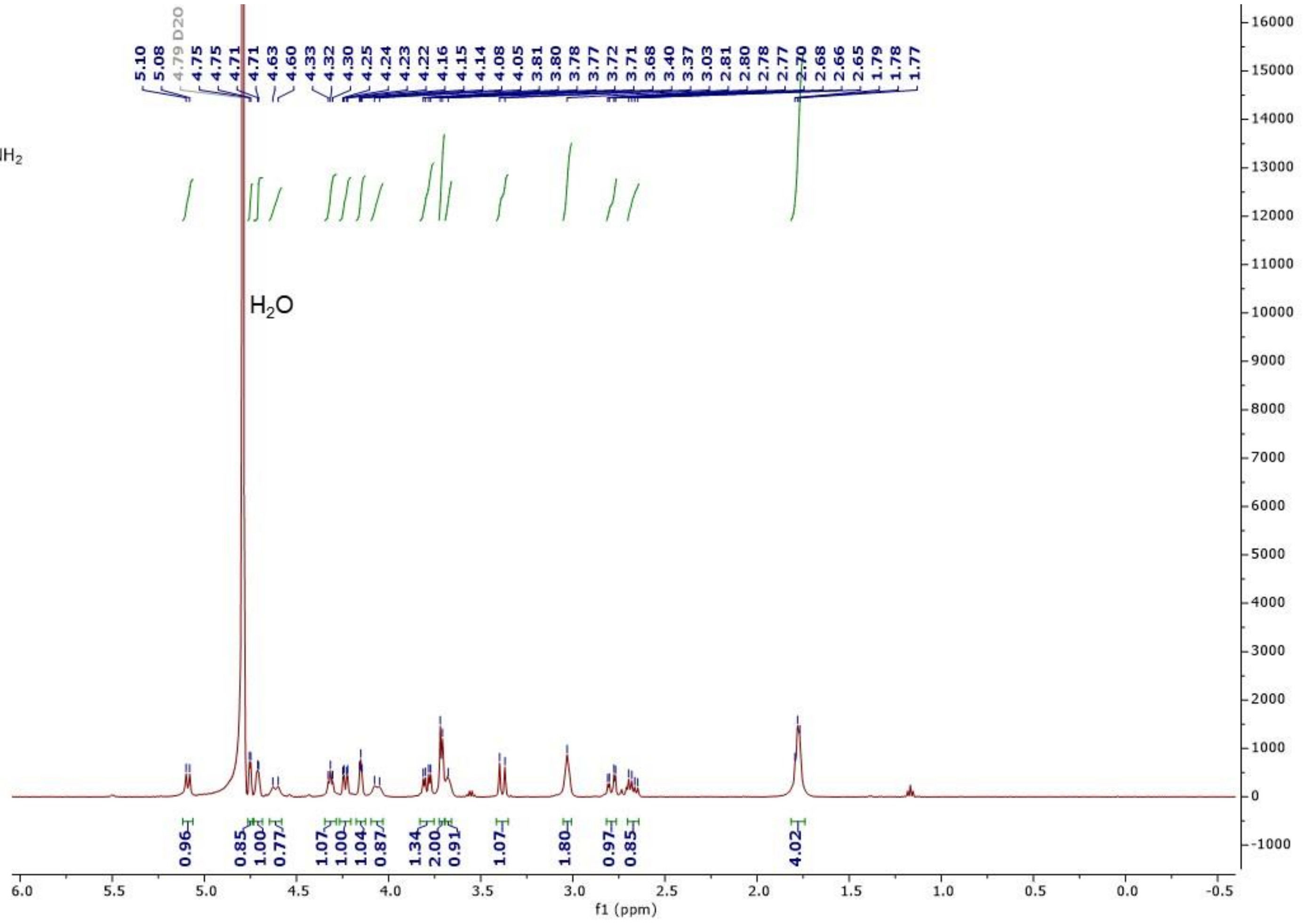

Isolated streptothricin F.

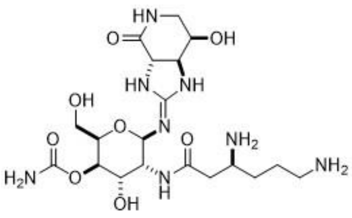

streptothricin F (**1**)

^1H NMR, D_2O, 500 MHz

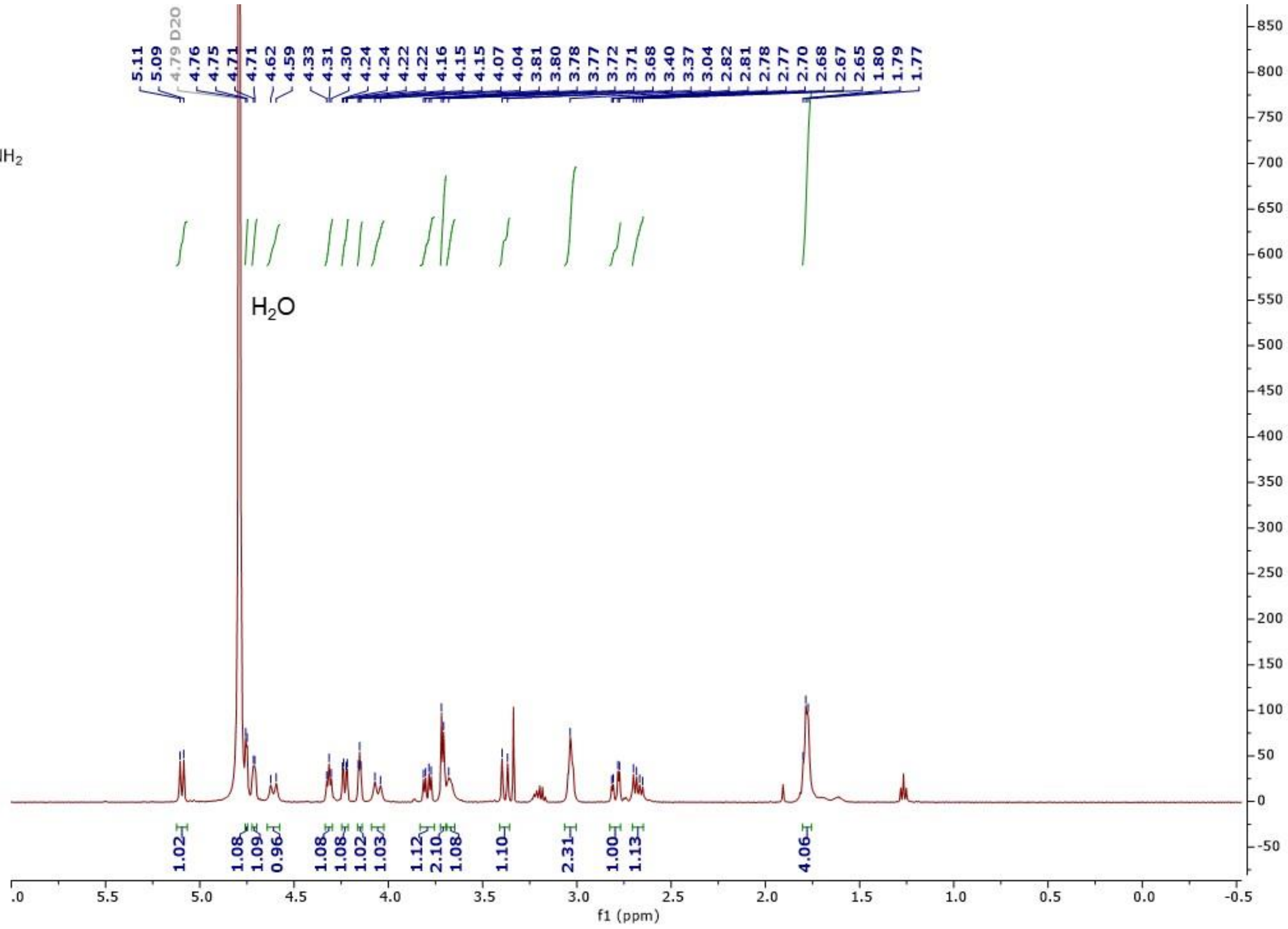

Isolated streptothricin F.

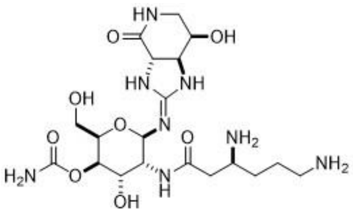

streptothricin F (**1**)

^1H NMR, D_2O, 600 MHz

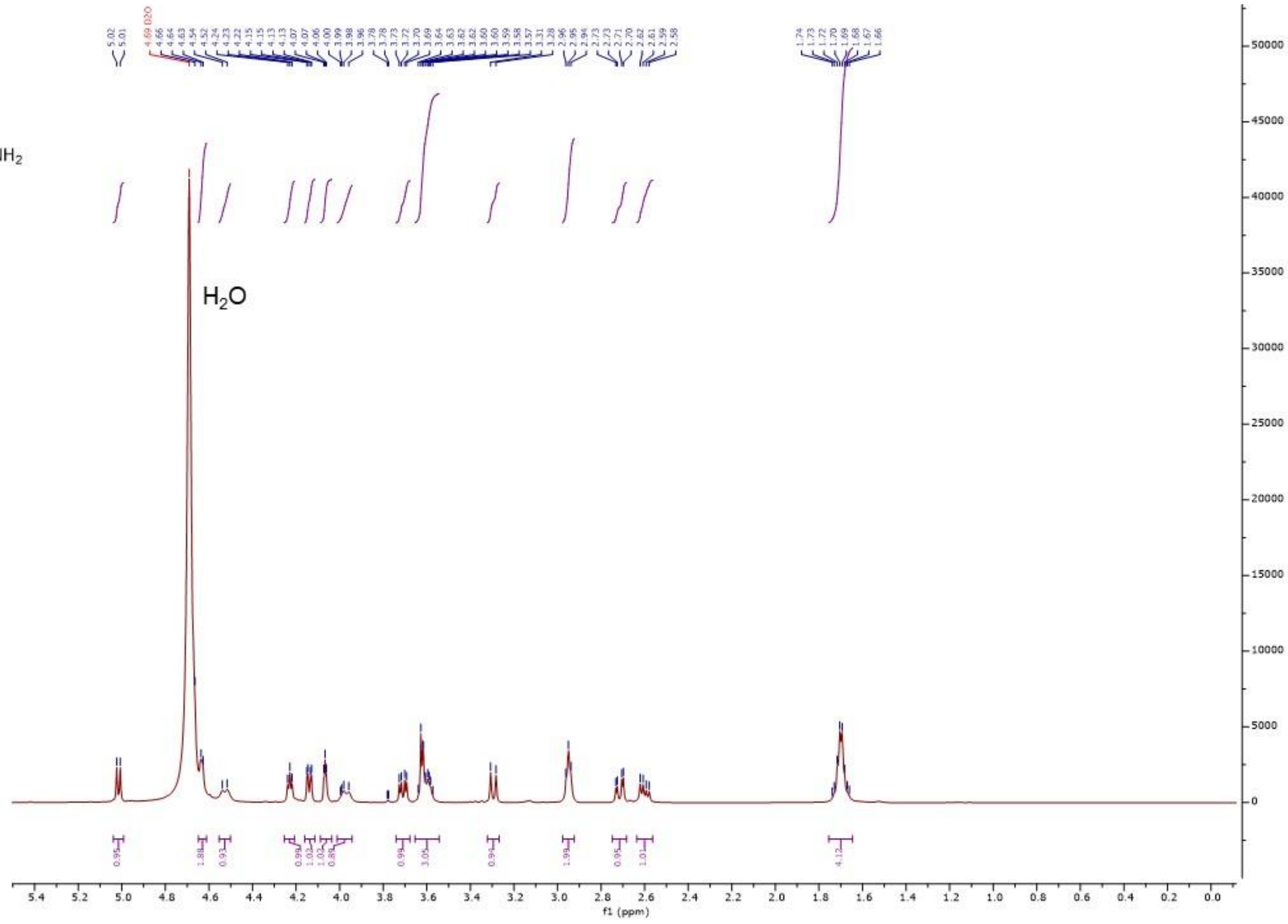

Isolated streptothricin F.

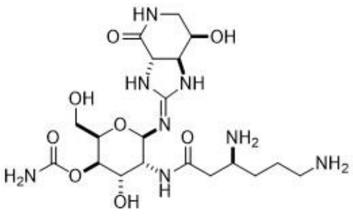

streptothricin F (**1**)

^{13}C NMR, D_2O, 150 MHz

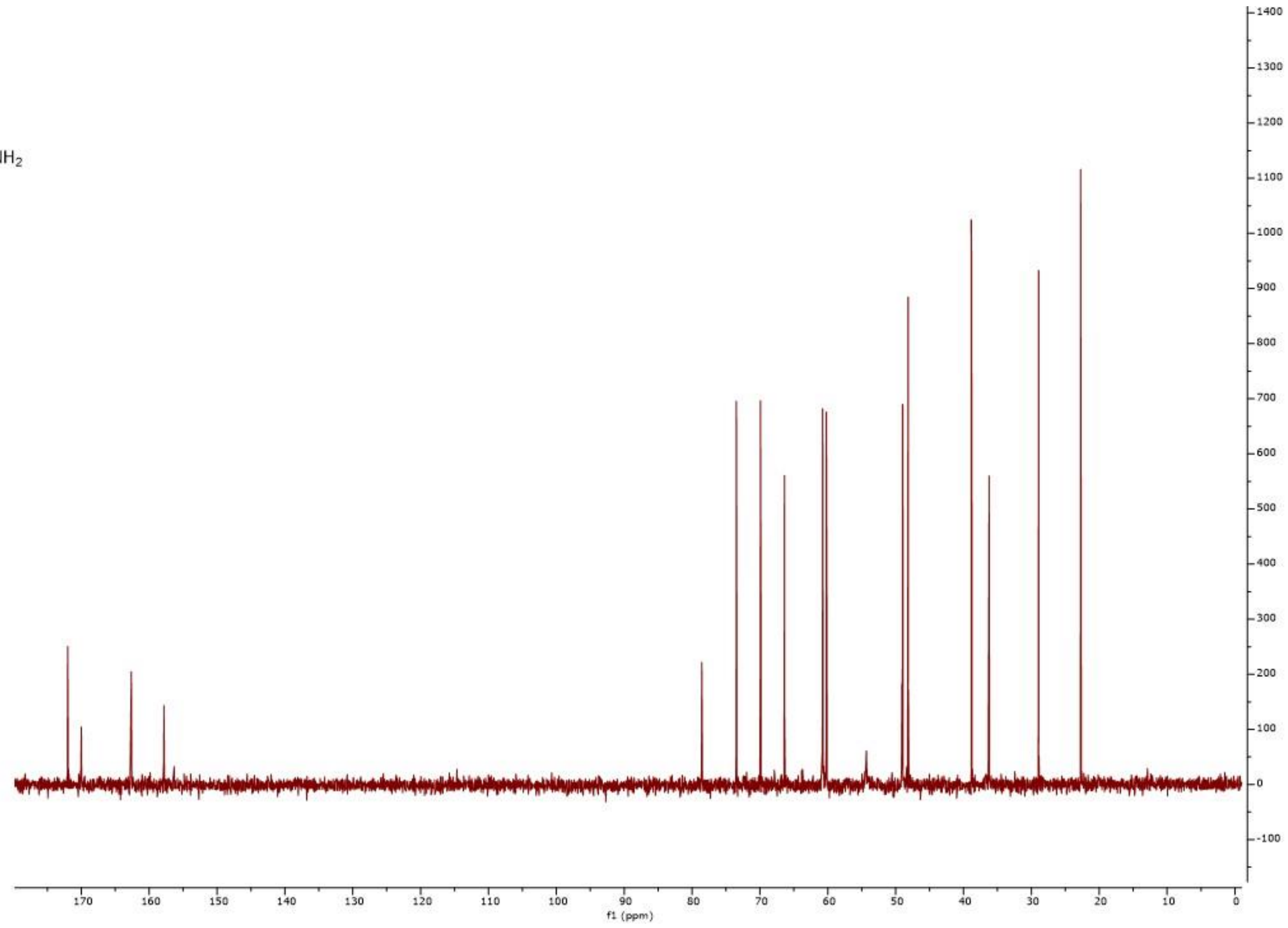

Isolated streptothricin F.

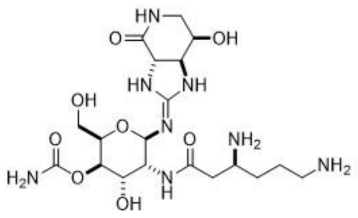
streptothricin F (**1**)

COSY, D_2O, 600 MHz

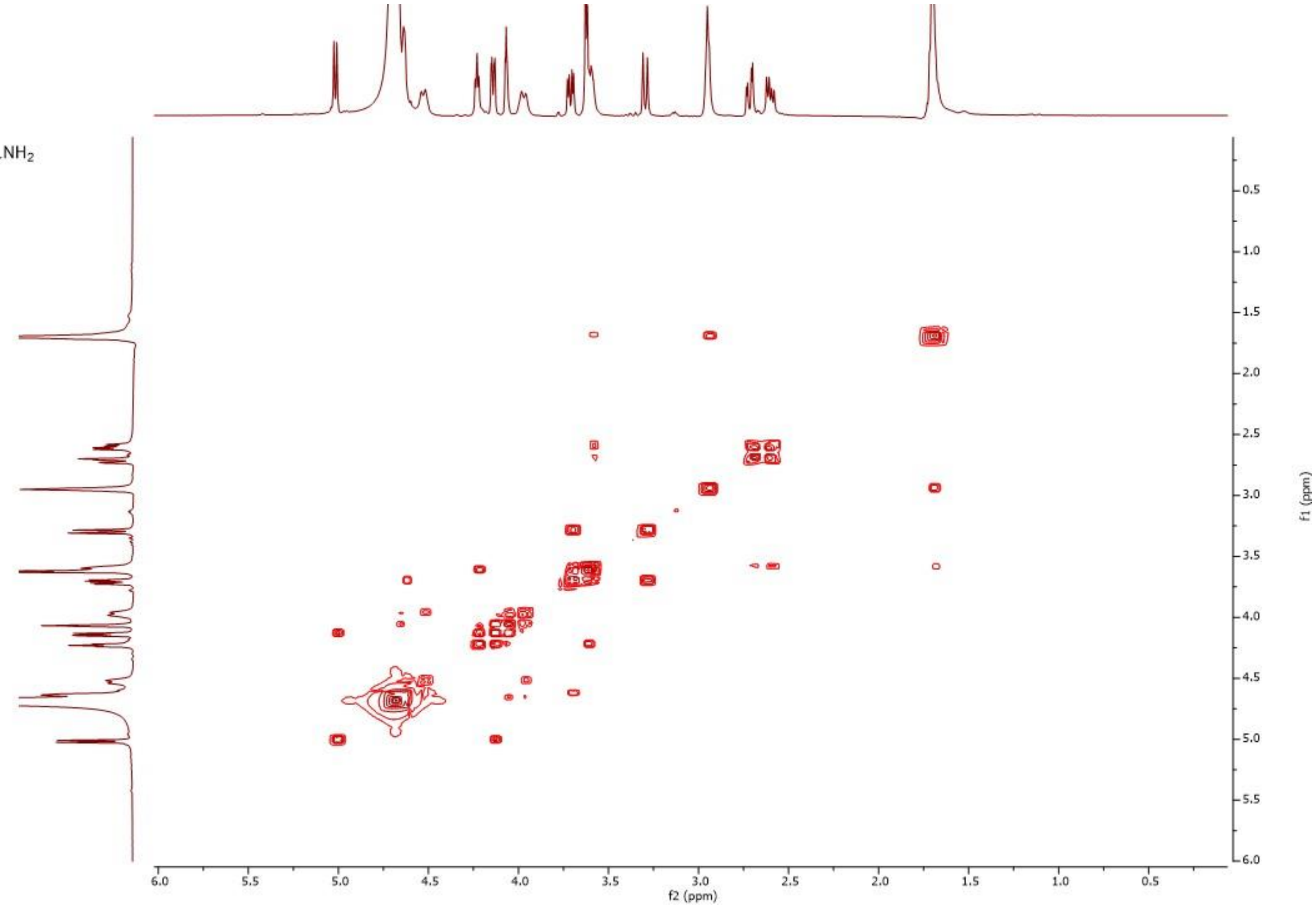

References:

S1. Yu, L.; Li, P. New simple primary amine–thiourea organocatalysts and their application in asymmetric conjugate addition. *Tetrahedron Lett.* **2014**, *55* (27), 3697–3700.

S2. Wakamiya, T.; Uratani, H.; Teshima, T.; Shiba, T. Synthesis of acyl derivatives of β-lysine for peptide synthesis. *Bull. Chem. Soc. Jpn.* **1975**, *48* (8), 2401–2402.

S3. Di Bussolo, V.; Caselli, M.; Pineschi, M.; Crotti, P. New stereoselective β- c -glycosidation by uncatalyzed 1,4-addition of organolithium reagents to a glycal-derived vinyl oxirane. *Org. Lett.* **2003**, *5* (12), 2173–2176.

S4. Yang Shaozu, Wei Juzhi, Wang Huan, L. C. A kind of preparation method and application of trimethyl silicane alkoxide. CN107235996A, 2017.

S5. Di Bussolo, V.; Caselli, M.; Romano, M. R.; Pineschi, M.; Crotti, P. Stereospecific uncatalyzed α-O-glycosylation and α-C-glycosidation by means of a new ᴅ-gulal-derived α vinyl oxirane. *J. Org. Chem.* **2004**, *69* (21), 7383–7386.

S6. Lo, F.; Kramer, T.; Boländer, A.; Plotkin, B.; Eldar-finkelman, H.; Fuertes, A.; Dominguez, J.; Schmidt, B. Bioorganic & medicinal chemistry letters synthesis and biological evaluation of glycogen synthase kinase 3 (GSK-3) inhibitors : an fast and atom efficient access to 1-aryl-3-benzylureas. *Bioorg. Med. Chem. Lett.* **2011**, *21* (18), 5610–5615.

S7. Nicolaou, K. C.; Snyder, S. A.; Longbottom, D. A.; Nalbandian, A. Z.; Huang, X. New uses for the Burgess reagent in chemical synthesis: methods for the facile and stereoselective formation of sulfamidates, glycosylamines, and sulfamides. *Chem. - A Eur. J.* **2004**, *10* (22), 5581–5606.

S8. Ji, Z.; Wang, M.; Zhang, J.; Wei, S.; Wu, W. Two new members of streptothricin class antibiotics from *Streptomyces qinlingensis Sp*. Nov. *J. Antibiot. (Tokyo).* **2007**, *60* (12), 739–744.

S9. Taniyama, H.; Sawada, Y.; Kitagawa, T. Characterization of racemomycins. *Chem. Pharm. Bull.* **1971**, *19* (8), 1627–1634.

S10. Peck, R. L.; Walti, A.; Graber, R. P.; Flynn, E.; Hoffhine, C. E.; Allfrey, V.; Folkers, K. Streptomyces antibiotics. VI. Isolation of streptothricin. *J. Am. Chem. Soc.* **1946**, *68* (5), 772–776.

S11. Kawamura, T.; Kimura, T.; Tago, K.; T Beppu, K. A. The identity of S15-1-A and B with racemomycins A and C. *J. Antibiot. (Tokyo).* **1976**, *29* (8), 844–846.

S12. Kusumoto, S.; Kambayashi, Y.; Imaoka, S.; Shima, K.; Shiba, T. Total chemical structure of streptothricin. *J. Antibiot. (Tokyo).* **1982**, *35* (7), 925–927.

S13. Clinical and Laboratory Standards Institute: Methods for Dilution Antimicrobial Suceptibility Tests for Bacteria that Grow Aerobically: Tenth Edition M07-A10. CLSI: Wayne, PA, USA, 2015.

S14. Smith, K. P.; Kirby, J. E. Verification of an automated, digital dispensing platform for at-will broth microdilution-based antimicrobial susceptibility testing. *J. Clin. Microbiol.* **2016**, *54* (9), 2288–2293.

S15. Brennan-Krohn, T.; Truelson, K. A.; Smith, K. P.; Kirby, J. E. Screening for synergistic activity of antimicrobial combinations against carbapenem-resistant enterobacteriaceae using inkjet printer-based technology. *J. Antimicrob. Chemother.* **2017**, *72* (10), 2775–2781.

S16. Brennan-Krohn, T.; Pironti, A.; Kirby, J. E. Synergistic activity of colistin-containing

combinations against colistin-resistant enterobacteriaceae. *Antimicrob. Agents Chemother.* **2018**, *62* (10), 1–11.

S17. Etienne-Toumelin, I.; Sirard, J. C.; Duflot, E.; Mock, M.; Fouet, A. Characterization of the *Bacillus anthracis* S-layer: cloning and sequencing of the structural gene. *J. Bacteriol.* **1995**, *177* (3), 614–620.

S18. Ransom, E.; Bhatnagar, A.; Patel, J. B.; Machado, M.; Boyd, S.; Reese, N.; Lutgring, J. D.; Lonsway, D.; Anderson, K.; Brown, A. C.; Elkins, C. A.; Rasheed, J. K.; Karlsson, M. Validation of aztreonam-avibactam susceptibility testing using digitally dispensed custom panels. *J. Clin. Microbiol.* **2020**, *58* (4), 1–9.
